# Characterizing rumen microbiota and CAZyme profile of Indian dromedary camel (*Camelus dromedarius*) in response to different roughages

**DOI:** 10.1038/s41598-021-88943-9

**Published:** 2021-04-30

**Authors:** Ankit T. Hinsu, Nilam J. Tulsani, Ketankumar J. Panchal, Ramesh J. Pandit, Basanti Jyotsana, Nishant A. Dafale, Niteen V. Patil, Hemant J. Purohit, Chaitanya G. Joshi, Subhash J. Jakhesara

**Affiliations:** 1 Department of Animal Biotechnology, College of Veterinary Science & A.H., Anand Agricultural University, Anand, 388001 India; 2 ICAR-National Research Centre On Camel, Bikaner, 334001 India; 3 ICAR-Central Arid Zone Research Institute, Jodhpur, 342003 India; 4 Environmental Biotechnology and Genomics Division, CSIR-National Environmental Engineering Research Institute, Nagpur, 440020 India; 5 Gujarat Biotechnology Research Centre, Gandhinagar, 382010 India

**Keywords:** Metagenomics, Microbial ecology, Microbiome

## Abstract

In dromedary camels, which are pseudo-ruminants, rumen or C1 section of stomach is the main compartment involved in fiber degradation, as in true ruminants. However, as camels are adapted to the harsh and scarce grazing conditions of desert, their ruminal microbiota makes an interesting target of study. The present study was undertaken to generate the rumen microbial profile of Indian camel using 16S rRNA amplicon and shotgun metagenomics. The camels were fed three diets differing in the source of roughage. The comparative metagenomic analysis revealed greater proportions of significant differences between two fractions of rumen content followed by diet associated differences. Significant differences were also observed in the rumen microbiota collected at different time-points of the feeding trial. However, fraction related differences were more highlighted as compared to diet dependent changes in microbial profile from shotgun metagenomics data. Further, 16 genera were identified as part of the core rumen microbiome of Indian camels. Moreover, glycoside hydrolases were observed to be the most abundant among all Carbohydrate-Active enzymes and were dominated by GH2, GH3, GH13 and GH43. In all, this study describes the camel rumen microbiota under different dietary conditions with focus on taxonomic, functional, and Carbohydrate-Active enzymes profiles.

## Introduction

Camels are characterized by the presence of one or more distinctive fatty deposits known as “humps” on their back. Fats stored in the humps are metabolized in the absence of food and water for longer periods^1^. It also helps camels to survive in harsh environments of arid, semi-arid, deserts and mountains. Camels are adapted to survive in wide temperature ranges, poor grazing conditions and scarcity of water. Furthermore, it is believed that certain physiological traits allow the camels to survive on a wide variety of vegetation available in the deserts including low-quality diet, salt-tolerant vegetation, and thorny plants^2,3^. However, the digestive system of camel differs from other herbivores like cattle and sheep (also known as true ruminant), as camels are pseudo-ruminants and have three-chambered stomach with no omasum^4^. Nonetheless, rumen is the main chamber responsible for fermentation of all ingested plant material in ruminants as well as camels. The rumen is a complex ecosystem containing a wide diversity of prokaryotic (bacteria and archaea) and eukaryotic (fungi and protozoa) micro-organisms interacting synergistically to ferment plant structural and non-structural carbohydrates and proteins^5^.

Conventional culture-based methods are the gold standard to study and isolate rumen microorganisms. However, molecular techniques like metagenomics have gained immense popularity with advancement in sequencing technologies^5,6^. Metagenomics involves direct study of microbiome by the means of sequencing its genetic material thereby bypassing the need of traditional culturing. Metagenomics has been extensively used to study microbiota of gut/rumen/caeca using various approaches^7–10^. 16S rRNA gene is present universally in all the prokaryotes making it an ideal target for diversity studies. Additionally, good resources in form of databases are also available for 16S rRNA gene. Therefore, the partial or full 16S rRNA gene sequencing has been a mainstay of sequence based bacteriome profiling for decades.

The studies involving the camel rumen microbiome are handful as compared to other ruminants. To name a few, previous studies on dromedary camel rumen microbiome employed 16S rRNA based approaches^8,11–13^ as well as shotgun metagenomics approach^14^. These studies have explored microbiota under different feeding conditions from free ranging and controlled environments. The present study was undertaken with the aim of characterizing the microbiota of camel rumen when fed with different roughage feeds namely Bajra, Jowar and Makai (Figure S1). These feeds have varying levels of lignocellulosic content and are used locally and traditionally as feed roughages. Two prominent Indian camel breeds were fed three different roughage and rumen content samples were collected across the period of 9 weeks. Further, 16S rRNA amplicon sequencing and shotgun metagenomics were used for phylogenetic and functional profile characterization of rumen microbial communities, respectively.

## Results

In total, 5.16 Gb of 16S amplicon sequencing data containing 10.2 million paired-end reads was generated from 120 samples. After all steps of DADA2 pipeline, 6.51 million clean paired-reads (63.75% of reads generated) with an average of 54,290 paired-reads per sample were assigned to 14,978 ASVs (Amplicon Sequence Variants) (Table S1). After filtering out ASVs present in 5 or less samples or having count of 5 or less, the remaining 4,794 ASVs were further analyzed using Phyloseq and other R packages.

### Diversity of communities

**Figure 1:**
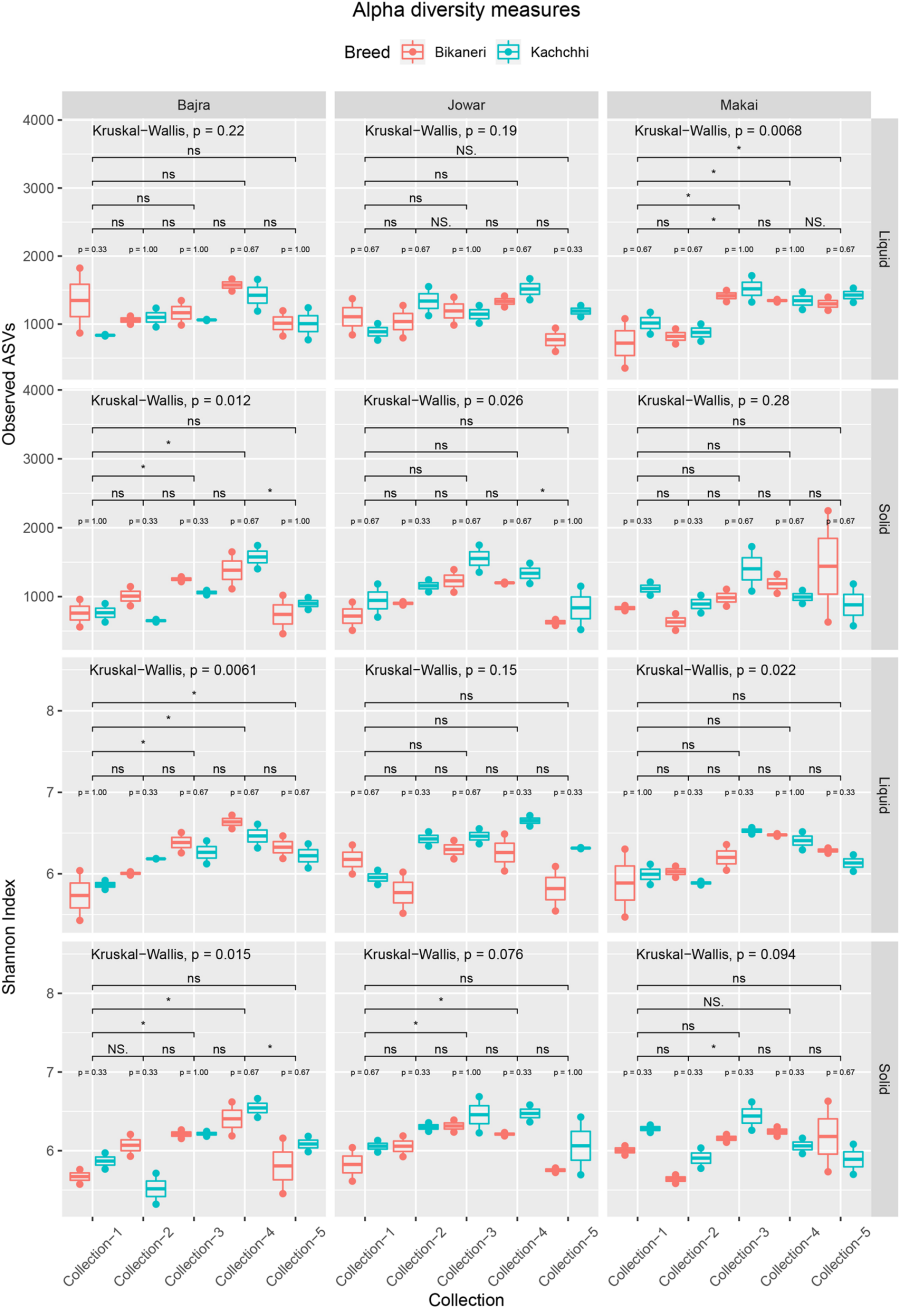
Alpha diversity measures (Observed ASVs, top and Shannon Index, bottom) distribution among all samples. Samples are colored by breed and separated based on feed and fraction. Wilcoxon test comparison between breeds are mentioned as “p = ” above the box-plots. Bars with p-value (*** < 0.001 < ** < 0.01 < * < 0.05; ns = not significant) mentioned above represents p-value from pairwise comparison of different Collections using Wilcoxon test. Kruskal–Wallis comparison among all the samples within same fraction and same feed is mentioned on the top of every facet.

Alpha diversity measures (Observed ASV and Shannon index) were calculated and compared to evaluate differences among the groups. Observed ASVs ranged from 356 to 2,250 with significant differences among liquid and solid fraction samples (Kruskal–Wallis, BH p-value = 0.026) and five collections (Kruskal–Wallis, BH p-value < 0.0001), while no significant differences observed between different feeds (Kruskal–Wallis, BH p-value = 0.83) and different breeds (Kruskal–Wallis, BH p-value = 0.38) (Fig. 1). Significant differences were also observed among collections within JS (JowarSolid) (Wilcoxon-test p-value = 0.036), BS (BajraSolid) (Wilcoxon-test p-value = 0.036) and ML (MakaiLiquid) (Wilcoxon-test p-value = 0.036) groups. On the other hand, Shannon diversity index ranged from 5.32 to 6.72 and differed significantly among collections within BL (Wilcoxon-test p-value = 0.037), BS (Wilcoxon-test p-value = 0.044) and ML (Wilcoxon-test p-value = 0.044) groups (Fig. 1). Significant differences (Wilcoxon-test p-value < 0.05) were also observed between Collection-4 and Collection-5 of BS (BajraSolid) and Collection-2 and Collection-3 of MS (MakaiSolid) groups. However, similar to Observed ASVs, significant differences were observed in Shannon index between different fractions (Kruskal–Wallis, BH p-value = 0.046) and collections (Kruskal–Wallis, BH p-value < 0.0001), but not among different feeds (Kruskal–Wallis, BH p-value = 0.83) and different breeds (Kruskal–Wallis, BH p-value = 0.2) as in Observed ASVs.

**Figure 2:**
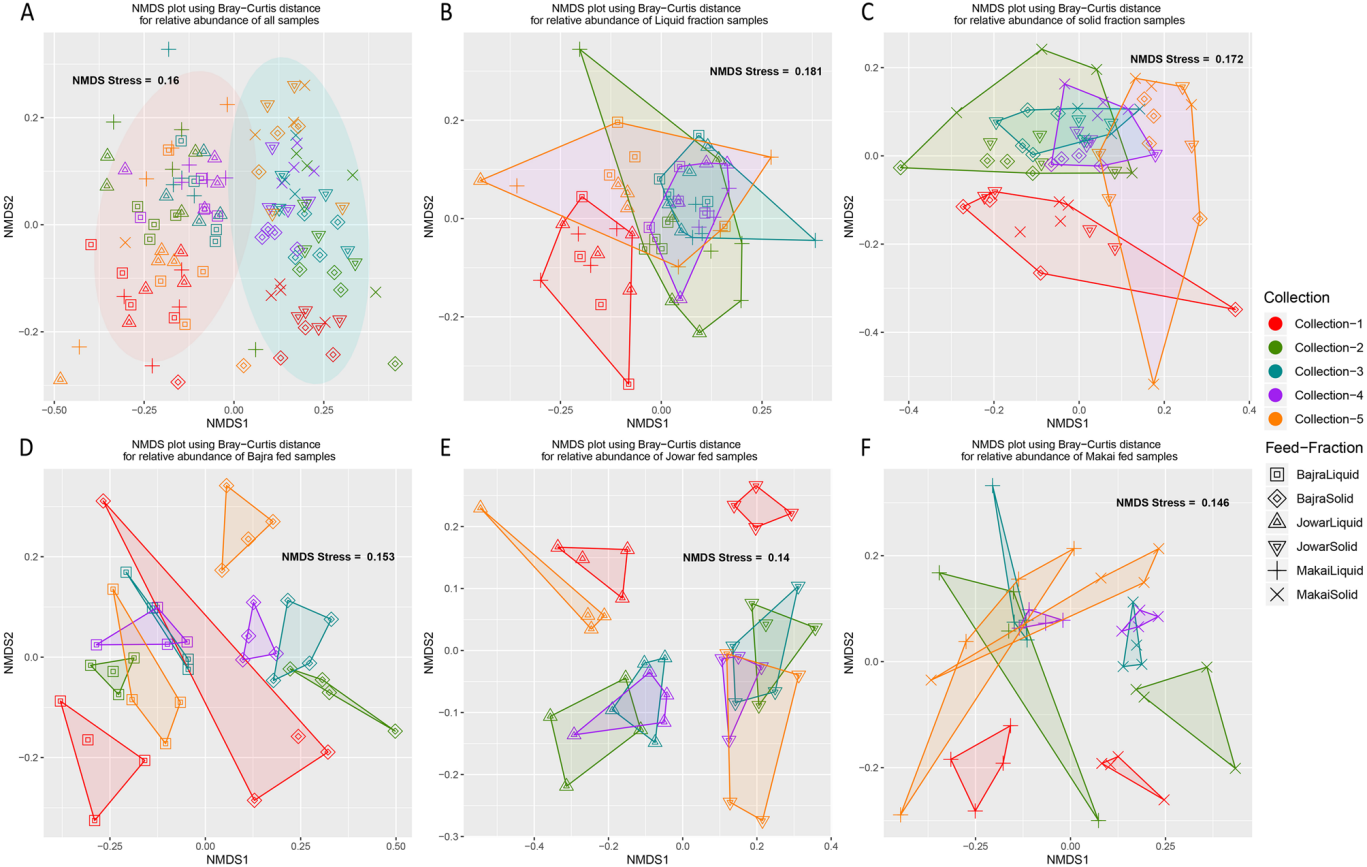
NMDS plots plotted from Bray–Curtis distances calculated from the relative abundances of (**A**) all samples, (**B**) samples from liquid fraction, (**C**) samples from solid fraction, (**D**) samples from Bajra fed animals, (**E**) Jowar fed animals and (**F**) Makai fed animals. All the plots are commonly colored by Collection and shaped by feed-fraction group.

Highly significant community level differences were observed between Fraction (PERMANOVA p-value < 0.001), Collection (PERMANOVA p-value < 0.001) and feed (PERMANOVA p-value < 0.001) based on the Bray–Curtis distances while less significant changes were contributed by breed (PERMANOVA p-value = 0.037) (Table 1). Structural similarities of these communities were visualized through NMDS (non-metric multidimensional screening) plot based on Bray–Curtis distance (Fig. 2). A clear separation of groups forming Liquid–Solid fractions and 5 collections were observed. Further, feed and collection were the most significant factors within liquid and solid fraction samples. Similarly, fraction and collection were the most significant factors within samples of each feed.

**Table 1:** Adonis (PERMANOVA) statistics applied on Bray–Curtis distance on relative abundance.

Group	Fraction	Feed	Breed	Collection
All samples	0.16687***	0.02930***	0.00993*	0.12292***
Liquid fraction	NA	0.04917**	0.01521ns	0.21033***
Solid fraction	NA	0.05176**	0.02283*	0.20835***
Bajra feed	0.18777***	NA	0.02751ns	0.18665***
Jowar feed	0.21741***	NA	0.03744*	0.17925***
Makai feed	0.15059***	NA	0.02413ns	0.20343***

Group column mentions the samples taken for respective calculations, while other columns are factors. Each value represents R^2^, p-value significance. *NA* Not applicable, *ns* not significant, ***< 0.001, **< 0.01, *< 0.05.

### Dietary effect on microbial taxa

**Figure 3:**
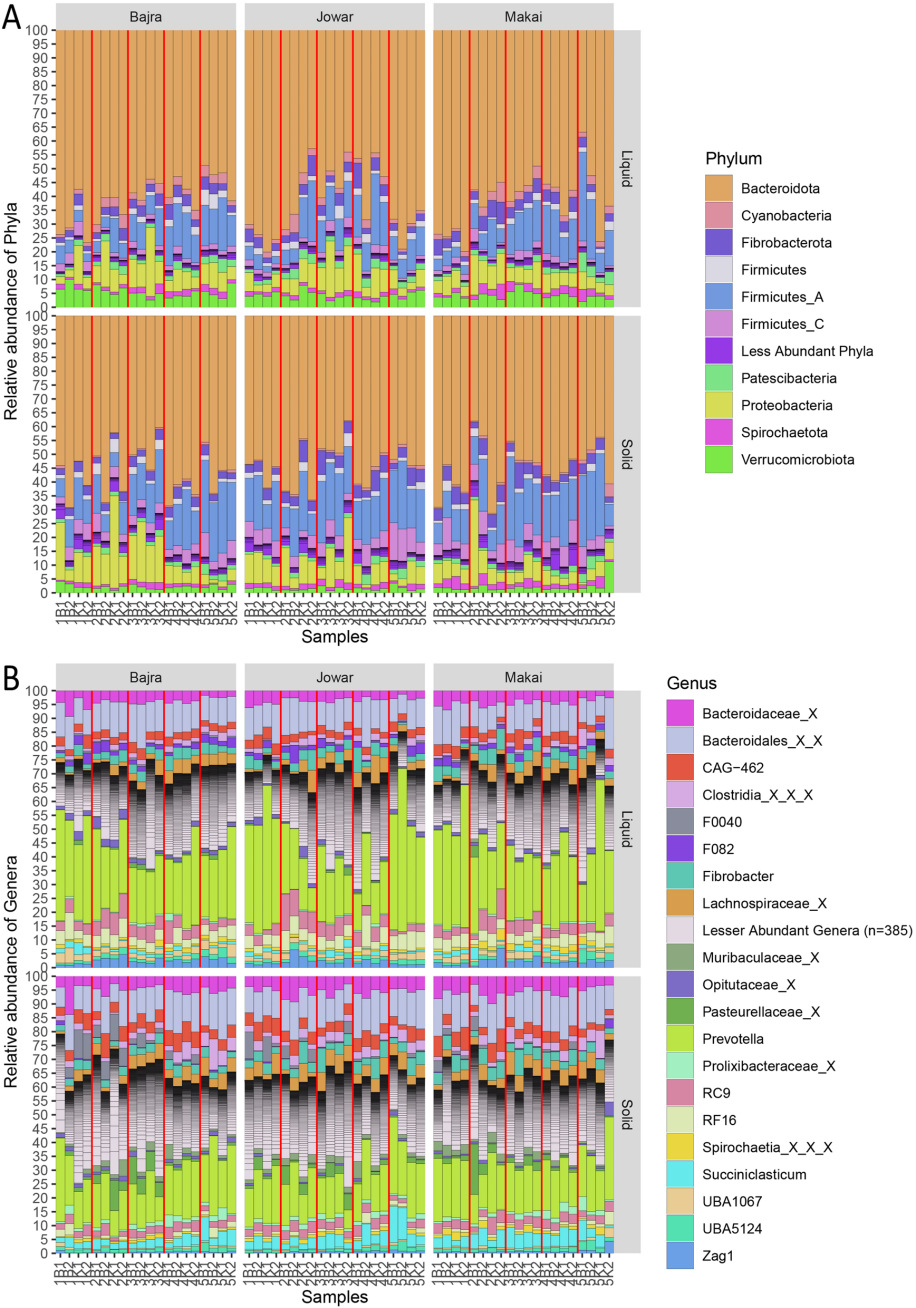
Bar plots showing diversity at (**A**). Phylum and (**B**). Genus level taxonomy. The samples are named and ordered as per Collection, Breed and animal number. Red vertical line differentiates different collections.

A total of 28 phyla were observed comprising 309 annotated genera, with 10 phyla having an average relative abundance greater than 1%. Bacteroidota (average rel. abundance: 58.2%), Firmicutes_A (13.4%), Proteobacteria (7.9%), Fibrobacterota (3.8%), Firmicutes_C (3.6%) and Verrucomicrobiota (3.3%) were some of the most abundant phyla (Fig. 3). While, *Prevotella* (phylum:Bacteroidota, 22.3%), *CAG-462* (Bacteroidota, 3.8%), *RC9* (Bacteroidota, 3.8%), *Fibrobacter* (Fibrobacterota, 3.7%), *Succiniclasticum* (Firmicutes, 2.8%), *RF16* (Bacteroidota, 2.8%), *Zag1* (Cyanobacteria, 1.5%), *UBA5124* (Patescibacteria, 1.4%), *UBA1067* (Verrucomicrobiota, 1.3%), *F0040* (Bacteroidota, 1.2%), *F082* (Bacteroidota, 1.1%) were the most abundant genera with an average relative abundance more than 1% (Fig. 3). Additionally, some unknown members of *Bacteroidaceae* (4.1%), *Lachnospiraceae* (3.8%), Pasteurellaceae (1.6%), *Opitutaceae* (1.1%), *Muribacullaceae* (1.0%), *Prolixibacteraceae* (1.0%) family; Bacteroidales (11.4%) order; and Clostridia (2.4%) class also had an average relative abundance more than 1%.

#### Fraction-wise comparison

Within liquid fraction, Bacteroidota phylum (mean ± sd, 61.32% ± 10.15%) was the most abundant across all samples, followed by Firmicutes_A (10.78% ± 6.03%), Proteobacteria (7.39% ± 4.38%), Verrucomicrobiota (4.65% ± 1.69%), Fibrobacterota (3.63% ± 1.56%) and others. Further, members of Bacteroidota phylum, *Prevotella* (27.31% ± 10.33%) and unknown members of Bacteroidales order (10.83% ± 2.49%) were the most abundant genera, followed by other genera like *RC9* (4.57% ± 2.37%), *RF16* (4.42% ± 1.79%), *Fibrobacter* (3.55% ± 1.55%) and others. Although, Bacteroidota (54.97% ± 8.15%) was the most abundant phylum in solid fraction, it was comparatively lower than liquid fraction. Further, other phyla like Firmicutes_A (16.11% ± 6.14%), Proteobacteria (8.46% ± 6.66%), Firmicutes_C (5.26% ± 2.4%) had comparatively higher abundance than liquid fraction samples. Compared to liquid fraction, most abundant genus *Prevotella* (17.31% ± 4.79%) decreased in solid fraction like its parent phylum Bacteroidota. However, other members of Bacteroidota phylum such as unknown genera from Bacteroidales order (11.95% ± 2.53%) and *Bacteroidaceae* family (5.45% ± 1.97%) were found to be increased in solid fraction. Additionally, other more abundant genera like *CAG-462* (4.49% ± 1.71%) and *Succiniclasticum* (4.29% ± 2.27%) also had higher abundance in solid fraction as compared to liquid fraction.

Between fractions, 17 out of 28 phyla differed significantly (Kruskal–Wallis, BH p-value < 0.05), which included highly abundant phyla like, Bacteroidota, Firmicutes_A, Firmicutes_C and Verrucomicrobiota (Table S2). In total, 243 genera differed significantly (Kruskal–Wallis, BH p-value < 0.05) between fractions (Table S2). These included all the abundant genera (average relative abundance > 1%) except *Fibrobacter*, *UBA5124* and an unknown genus from Spirochaetia class.

#### Feed-wise comparison

Firmicutes_I (Kruskal–Wallis, BH adjusted p-value = 0.031); and Campylobacterota (Kruskal–Wallis, BH p-value = 0.026) & Fibrobacterota (Kruskal–Wallis, BH p-value = 0.026) differed significantly among three feeds in liquid and solid fractions, respectively (Table S3). Additionally, 5 (Elusimicrobiota, Firmicutes_B, Firmicute_I, Riflebacteria and Verrucomicrobiota) and 8 (Actinobacteriota, Campylobacterota, Fibrobacterota, Planctomycetota, Riflebacteria, Spirochaetota, Synergistota and Verrucomicrobiota) phyla also differed significantly (Kruskal–Wallis, p-value < 0.05; not as per BH adjusted values) among feeds from liquid and solid fraction samples, respectively. Amongst observed genera, 14 and 1 genera (all of which were observed in lesser proportion) differed significantly (Kruskal–Wallis, BH p-value < 0.05) among feeds from liquid and solid fraction samples, respectively. While, 54 and 38 genera differed significantly (Kruskal–Wallis, p-value < 0.05) from liquid and solid fraction samples, respectively which included 5 genera with abundance > 1% (*F0040*, *Fibrobacter*, *Succiniclasticum* and the unknown members of *Opitutaceae* family and Spirochaetia class) (Table S3).

#### Collection-wise comparison

Amongst all collections, 14 phyla differed significantly (Kruskal–Wallis, BH p-value < 0.05) in both liquid and solid fraction samples including most abundant phyla Bacteroidota and Firmicutes_A (Table S4). Furthermore, 144 and 167 genera differed significantly (Kruskal–Wallis, BH p-value < 0.05) between collections in liquid and solid fractions, respectively (Table S4). Further, 5 phyla & 38 genera; and 9 phyla & 43 genera differed significantly (Wilcoxon-test, BH p-value < 0.05) between Collection-1 and Collection-5 in liquid and solid fractions, respectively (Table S5 and Table S6). Comparatively, more number of taxa differed significantly from Collection-1 in (Wilcoxon-test, p-value < 0.05) Collection-3 (15 phyla and 174 genera); and Collection-4 (13 phyla and 183 genera) compared to Collection-2 and Collection-5 (Table S7).

### The core rumen microbiome

**Figure 4:**
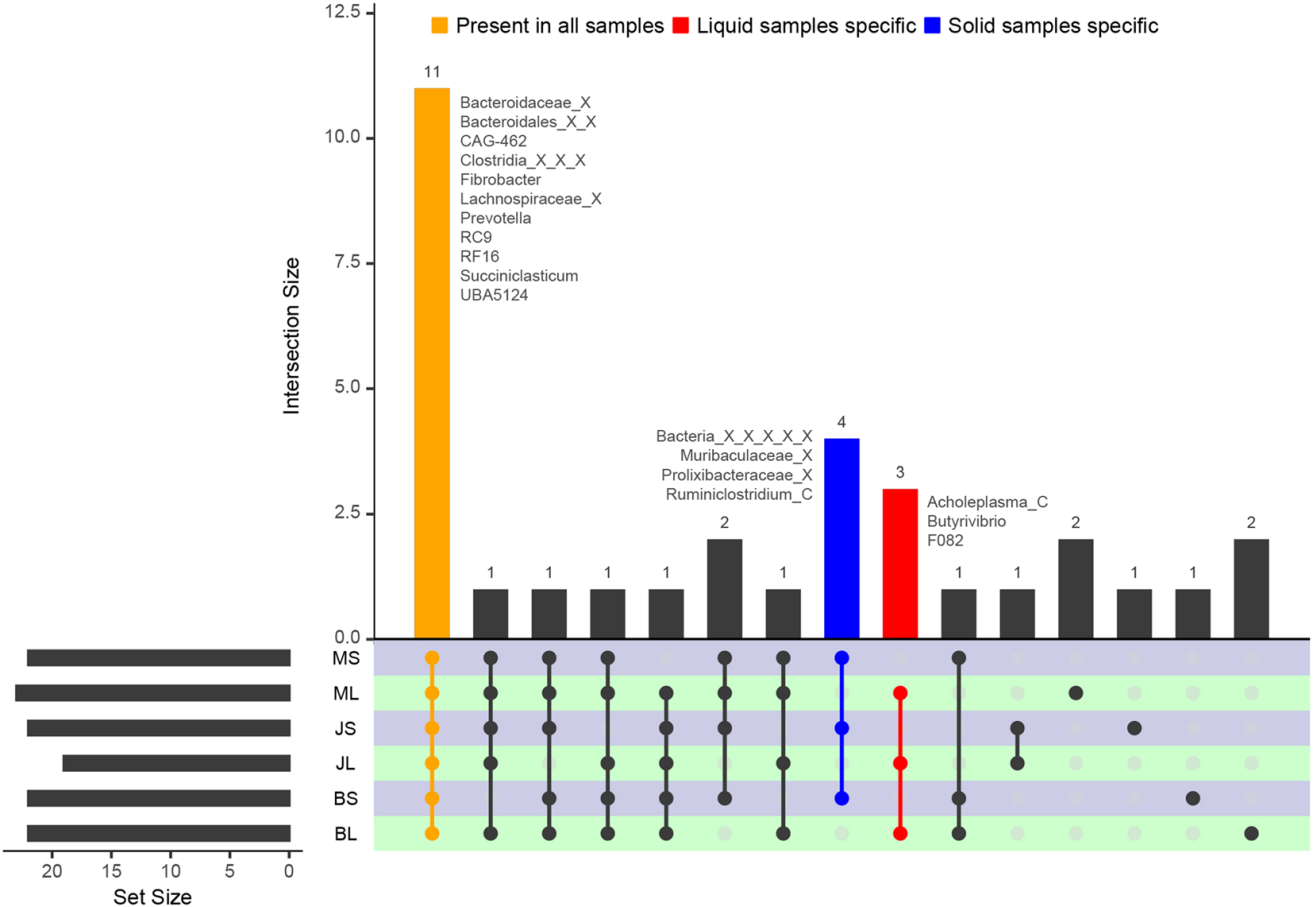
UpSet plot showing intersections among six groups of three feed and two fractions. Bars colored in Yellow, Blue and Red shows genus/taxa exclusively observed in all groups, Solid samples and Liquid samples, respectively. The names of taxa in colored bars are mentioned besides the bar.

Core microbiome (minimum abundance 0.5%, minimum prevalence > 50%) was identified in each feed-fraction group from the 63-day collection. With a total of 33 unique genera, 22, 22, 19, 22, 23 and 22 genera were identified as core microbiome for BL (BajraLiquid), BS (BajraSolid), JL (JowarLiquid), JS (JowarSolid), ML (MakaiLiquid) and MS (MakaiSolid) groups, respectively. Out of these, 11 genera with an average abundance > 1% were present in all the groups forming the core microbiome (Fig. 4). Additionally, 4 genera (*Ruminiclostridium_C*, an unclassified genus each from *Muribaculaceae* and *Prolixibacteraceae* family and unclassified Bacteria) were present only in solid group and 3 genera (*Acholeplasma*_C, *Butyrivibrio* and *F082*) were present only in liquid group. Further, 2, 1, 1 and 2 genera were detected exclusively in BL, BS, JS and ML groups, respectively.

### Shotgun metagenomics

Total 118.34 GB of shotgun metagenomic sequencing data (Table S8) from samples of the last two collections (n = 48) was analyzed using SqueezeMeta co-assembly pipeline. Co-assembly resulted in 4,277,503 contigs (> 200 bp) with 2.27 Gbp size. Additionally, 47.91%—77.36% reads per sample were mapped back to the assembly with an average of 65.69%. A total of 5,145,814 genes were predicted from all the contigs and annotated for COG and CAZymes. SqueezeMeta pipeline expresses gene abundance in the form of TPM (Transcripts per million)^15^. TPM is similar to RPKM (reads per kilobase per million reads) and represents the number of times a gene is observed per randomly sampled million genes.

#### Functional annotation

Around 53.87% of predicted ORFs were annotated by the COG database (46.9%-70.4% per sample). A total of 11,019 unique COGs were categorized in 60 different classes across all the samples. The NMDS plot based on Bray–Curtis distance of TPM of COG ids showed a clear separation between liquid and solid fractions (PERMANOVA R^2^:0.20360, p-value < 0.001) (Figure S2). Remarkably, no significant differences were observed among the feed type (PERMANOVA R^2^:0.02854, p-value = 0.663) and Breed (PERMANOVA R^2^:0.01490, p-value = 0.547), while less significant differences were observed between Collections (PERMANOVA R^2^:0.03847, p-value = 0.020).

**Figure 5:**
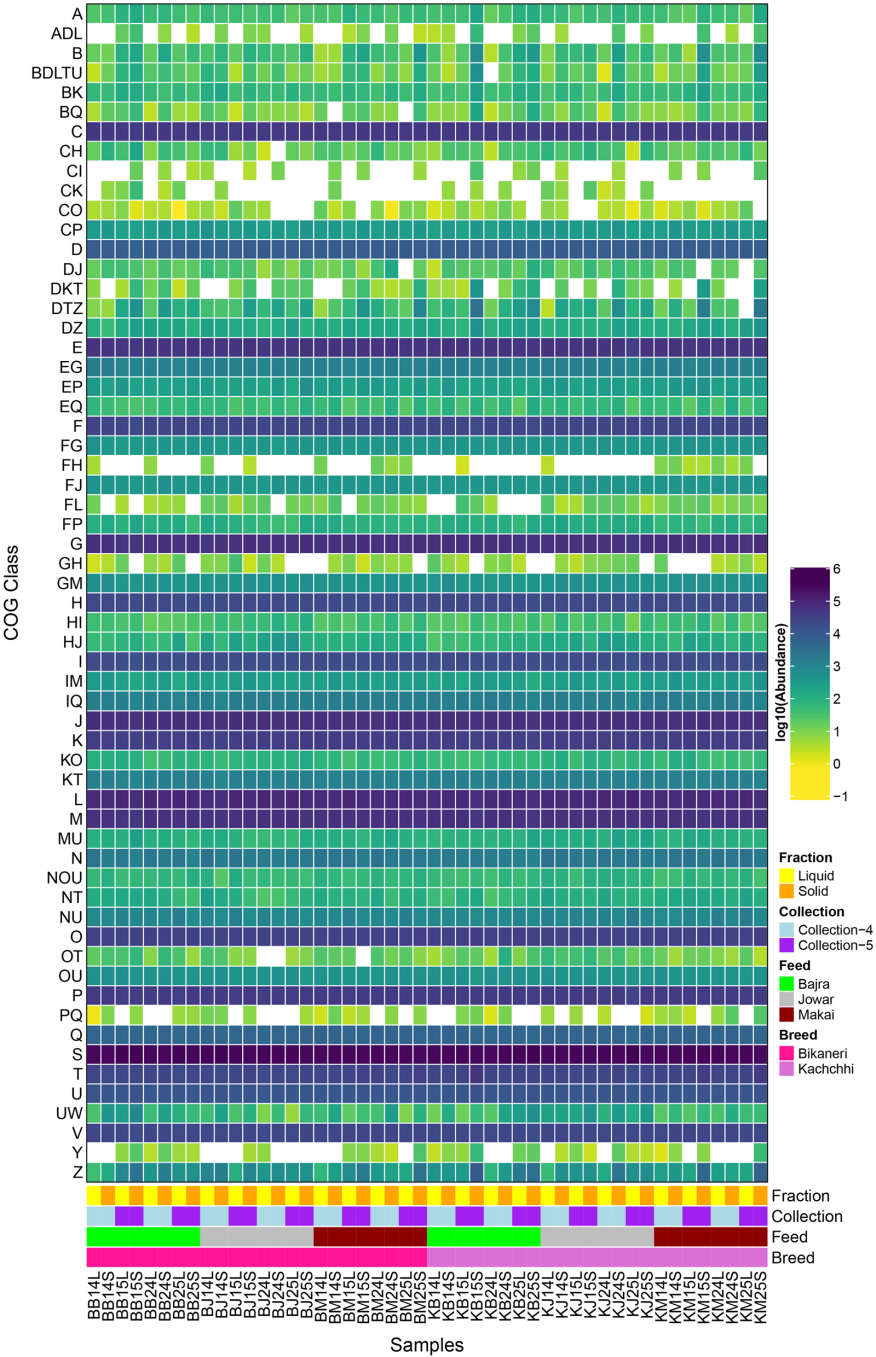
Heatmap representing the abundance of COG classes among all the samples of shotgun data. A to Z symbols represent COG categories and COGs presented by more than one COG class is giving by writing corresponding COG class code together. CELLULAR PROCESSES AND SIGNALING: [D] Cell cycle control, cell division, chromosome partitioning, [M] Cell wall/membrane/envelope biogenesis, [N] Cell motility, [O] Post-translational modification, protein turnover, and chaperones, [T] Signal transduction mechanisms, [U] Intracellular trafficking, secretion, and vesicular transport, [V] Defense mechanisms, [W] Extracellular structures, [Y] Nuclear structure, [Z] Cytoskeleton; INFORMATION STORAGE AND PROCESSING: [A] RNA processing and modification, [B] Chromatin structure and dynamics, [J] Translation, ribosomal structure and biogenesis, [K] Transcription, [L] Replication, recombination and repair; METABOLISM: [C] Energy production and conversion, [E] Amino acid transport and metabolism, [F] Nucleotide transport and metabolism, [G] Carbohydrate transport and metabolism, [H] Coenzyme transport and metabolism, [I] Lipid transport and metabolism, [P] Inorganic ion transport and metabolism, [Q] Secondary metabolites biosynthesis, transport, and catabolism; POORLY CHARACTERIZED: [R] General function prediction only, [S] Function unknown.

Further, functional annotation revealed 60 COG classes, which included 23 unique classes while, 32, 4 and 1 classes with a combination of 2, 3 and 5 COG classes, respectively (Fig. 5). As expected, most abundant COG class was S (Function unknown) followed by L (Replication, recombination and repair), J (Translation, ribosomal structure and biogenesis), G (Carbohydrate transport and metabolism), M (Cell wall/membrane/envelope biogenesis), E (Amino acid transport and metabolism), C (Energy production and conversion), P (Inorganic ion transport and metabolism), K (Transcription), O (Post-translational modification, protein turnover, and chaperones) and others. Further, significant differences (Kruskal–Wallis BH adjusted p-value < 0.05) were observed in 27, 7 and 6 classes between fractions, collections, and feeds, respectively while no COG class differed significantly between breeds. These included categories involved in Metabolism (Amino acid transport and metabolism [E]; Nucleotide transport and metabolism [F]; Carbohydrate transport and metabolism [G]; and Inorganic ion transport and metabolism [P]) and Cellular processing and Signaling (Cell cycle control, cell division, chromosome partitioning [D]; Cell motility [N]; Post-translational modification, protein turnover, and chaperones [O]; Intracellular trafficking, secretion, and vesicular transport [U]; Defense mechanisms[V]; and Cytoskeleton [Z]).

#### The carbohydrate‑active enzyme repertoire

The rumen microbiota breakdown and ferment lignocellulosic materials from feed into VFAs^16^. Hence, studying the genomic constituents of microbial communities for carbohydrate-active enzymes (CAZymes) will hint towards the repertoire of enzymes involved in feed degradation^16^. A total of 65,904 predicted ORFs were annotated to be coding for CAZymes as per HMM based prediction. A major proportion of these CAZymes were comprised of Glycoside hydrolases (GH, 54.51%) followed by Glycosyl transferases (GT, 25.26%), Carbohydrate esterases (CE, 11.91%), Carbohydrate binding molecules (CBM, 3.8%), Polysaccharide lyases (PL, 3.58%) and Auxiliary activities (AA, 0.07%), while rest were either annotated as Cohesin or S-layer homologous (SLH) or contained more than one CAZy class. Majority of these CAZymes coding-ORFs (~ 95%) were annotated as Bacteria. Further, these ORFs belonged mainly to Bacteroidota (60%) phylum followed by Firmicutes (14%) and Fibrobacteres (4%) with 13.3% ORFs remaining unclassified, while at genus level, *Prevotella* (23%), *Bacteroides* (4%), *Fibrobacter* (4%), *Ruminococcus* (1%) and *Butyrivibrio* (1%) were the most classified genera.

**Figure 6:**
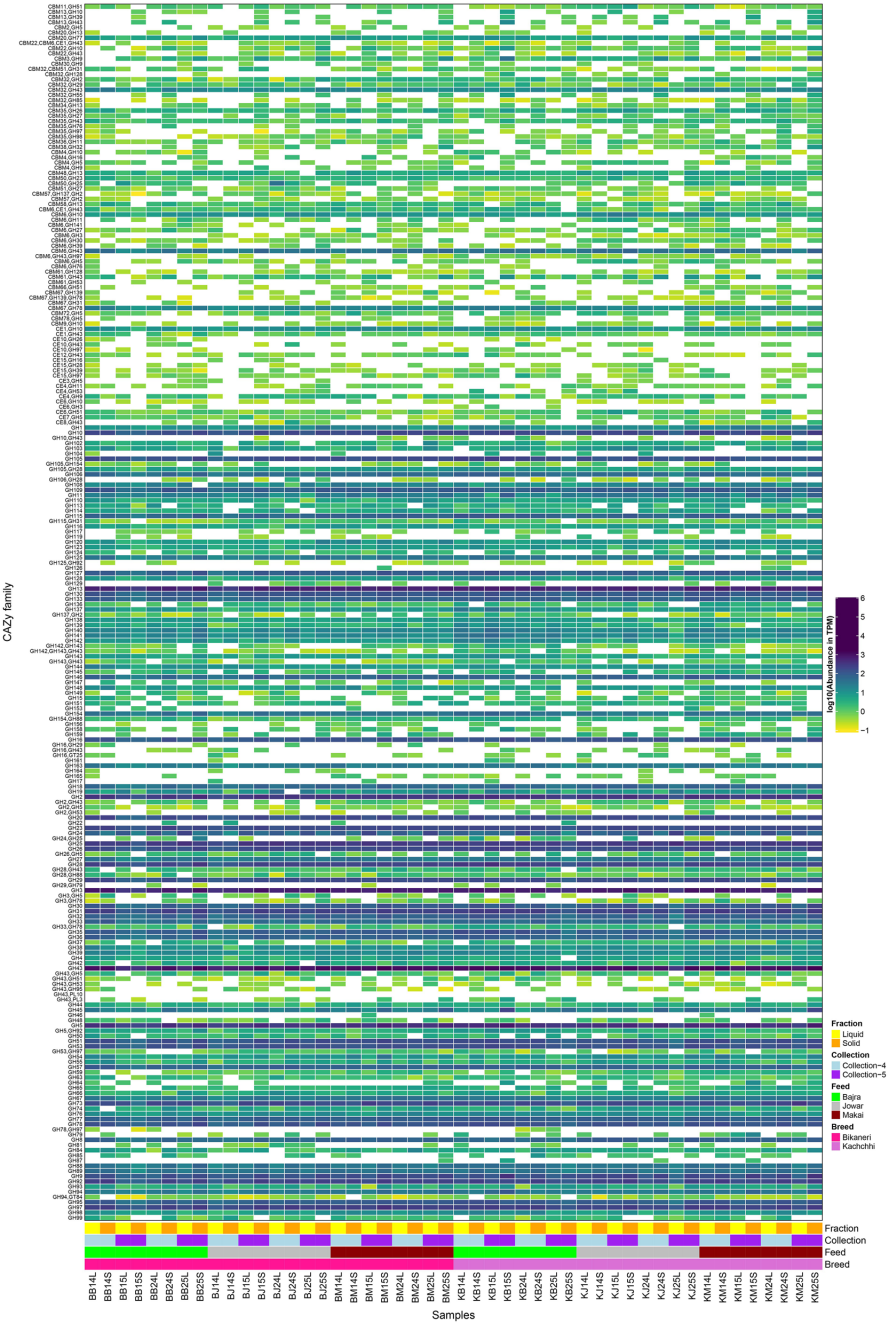
Heatmap showing distribution of all the CAZyme categories which were annotated to contain at least one GH family.

Around 233 unique CAZyme families (84 having GH + other families, 118 having single GH family and 31 having multiple GH families) containing GH were analyzed further (Fig. 6). GH43, GH13, GH2 and GH3 were the most abundant GH families. Most of these genes were encoded by phylum Bacteroidetes (GH2:87%, GH3:62%, GH13:53% and GH43:75%) with a major proportion of *Prevotella* followed by *Bacteroides* and little contributions from *Alistipes* genera. Other major contributing phyla included Firmicutes (GH2:4%, GH3:19%, GH13:24% and GH43:13%), Proteobacteria (GH3:1%, GH13:4% and GH43:0.3%) and Fibrobacteres (GH2:0.9%, GH3:0.7%, GH13:1% and GH43:4%) (Figure S3).

Further, 104, 1 and 15 families differed significantly (Kruskal–Wallis BH adjusted p-value < 0.05) between fractions, collections, and feeds, respectively (Figure S4). Among the most abundant families (TPM abundance > 100), families GH43, GH13, GH3, GH5, GH97, GH92, GH9, GH78, GH51, GH31, GH29, GH20, GH2, GH16, GH10 were significantly higher in solid fraction as compared to liquid fraction, while only few families such as GH24, GH25 and GH73 were more abundant in liquid fraction. Further, families GH65, GH1 and GH120 were significantly more abundant in Maize feed, while GH159, GH10 + CBM4 were more abundant in Jowar and GH5 + GH92 and GH139 were more abundant in Bajra feed.

## Discussion

Rumen ecosystem harbors a great diversity of microbes with wide variety of roles. It is speculated that a diverse set of microbes start fermenting incoming feed particles and then a different set of microbes starts acting on fermented feed^17,18^. The microbes adhered to feed particles are quite different from those present in the fluid. These differences are not only limited to the taxonomy of microbes but to their functions/metabolism as well^12,17,19^. In this study, significant differences were observed among liquid and solid fraction microbiota at both taxonomic and functional levels as reported in previous studies on camel rumen^12^. At a higher taxonomic level, the proportion of two major phyla Bacteroidota and Firmicutes differed between both the fractions. In accordance with the previous studies on rumen, Firmicutes (Firmicutes_A and Firmicutes_C) were comparatively higher in solid fraction as compared to liquid fraction and vice versa for Bacteroidota phylum^20,21^. Firmicutes phylum was split in multiple phyla according to the taxonomy of GTDB^22^. GTDB follows the standardized bacterial taxonomy based on genome phylogeny and hence, differs from the traditional taxonomy from NCBI or other databases. However, significant differences were observed only in Firmicutes_A and Firmicutes_C and not in Firmicutes phylum. The reason being that Firmicutes_A and Firmicutes_C phylum includes class Clostridia and Negativicutes, respectively which are commonly associated with fiber degradation and reported in higher abundances in rumen, justifying their higher abundance observed in Solid fraction^12,17,20^. The Firmicutes phylum includes class Bacilli having lesser abundance in anaerobic rumen environment and are not modulated by other parameters^17,20^.

At genus level, *Prevotella* was the most abundant genus from Bacteroidota phylum. Other abundant genera from Bacteroidota phylum included *CAG-462, RC9, RF16, F0040, F082* and some other taxa annotated at higher level. All these taxa belong to Bacteroidales order and were reconstructed from metagenomes. *Bacteroides*, a major genus from this order along with *Prevotella* are some of the most commonly observed genera in rumen of both ruminants and pseudo-ruminants and were reported in previous studies on bovine, sheep, goat, camel, alpaca^23–26^. The *RC9* and *RF16* genera were observed in higher abundance especially in liquid fraction. The previous studies on camel and moose rumen have also reported the higher abundances of *RC9*^12,27^ and *RF16* genera^25,28^, respectively.

*Fibrobacter* is yet another important member of the rumen community, reported in several studies. *Fibrobacter* is mainly associated with cellulolytic-fiber degradation and hence is an integral part of the rumen community. However, unlike previous studies in camel^8,12^ present study did not reported significant difference in its abundance between liquid and solid fractions but observed similar abundance levels. Previous studies on rumen have also observed similar abundance of *Fibrobacter* in cattle and buffalo rumen^20,25^ but lower abundance in other members of camelids^25^. Another genus *Succiniclasticum* known to ferment only succinate to propionate and not any other carbohydrates or amino acids^29^, which explains its significantly higher abundance in solid fraction compared to liquid fraction. Several bacteria from families *Opitutaceae* and *Muribacullaceae* were also observed in comparatively higher abundance in liquid and solid fractions, respectively. Members of *Opitutaceae* family have been isolated from soil, terrestrial environment and gut of ants and wood-feeding termites^30–32^. They have the ability of degrade lignocellulosic biomass and explains their presence in environments with plant biomass such as camel rumen. *Muribacullaceae* family was described very recently and the members of the family have been reported from mouse gut (for which it was named) and chicken caecum^33,34^. The members of this family are also shown to possess lignocellulosic degradation capabilities^33,34^.

From the functional point of view, around 33% of the annotated genes were annotated as “Functions Unknown” (COG Class-S). While higher (> 5% average) annotated COG class includes Class L (Replication, recombination and repair), Class J (Translation, ribosomal structure and biogenesis), Class G (Carbohydrate transport and metabolism), Class M (Cell wall/membrane/envelope biogenesis) and Class E (Amino acid transport and metabolism). The findings are in line with earlier studies on rumen, wherein a major proportion of genes involved in Genetic Information processing (Class L and J) followed by genes involved in Carbohydrate and Amino acid metabolism (Class G and E) were reported^14,17^. Previous studies have also described a higher degree of functional differences between fractions^17,20^.

Comparatively, lesser functional differences were observed between Collections (seven classes), and Feed (five classes), while no differences were observed between the breeds. While diet is one of the important factors influencing the shape of the rumen microbiome, few studies have observed that the taxonomic changes due to diet are more evident than functional changes^17,20,35,36^. Based on the results obtained in the study, it is speculated that the changes in the diet leads to the change in the abundance of the microbiota which gradually becomes stable under the influence of the same diet. Previous studies have shown that a period of 4–6 weeks can stabilize these diet related changes in rumen microbiota^37,38^. Incidentally, we did observe the greater number of significantly differentiating (Kruskal–Wallis, p-value < 0.05; not as per BH adjusted values) genera in Collection-2 (50) followed by Collection-4 (37), Collection-3 (33), Collection-5 (18), and Collection-1 (8), further substantiating the fact that indeed the most variation observed was immediately after diet change and decreased with time, whereas 0th day had the least feed-dependent variations as expected. This could also be the reason why lesser changes were observed between collections in the functional profile as they were based only on the last two collections.

The feeds included in present study were selected on the basis of their lignocellulosic content and therefore, we expected differences among the diet groups. However, we found less or no significant variations in taxa and diversity among the diet groups. The observed changes were also comparatively less pronounced as compared to similar experiments across multiple ruminants^25,39^ as well as compared to variations in roughage-concentrate proportion of same feed^40,41^. While the functions of rumen in camels and cattle are similar, there are some of the differences associated with the physiology of animal which might result in less pronounced differences among feed associated microbiota. It is also probable that camels being able to survive on a wide variety of plant-based diets available in scarce environments, change in the diet might have lesser impact on camel rumen microbiota as compared to true ruminants. We also speculate that including larger group of animals in further studies can provide more reliable findings confirming the effects of change in diet on rumen microbiota. Another point worth mentioning here is the probability of the introduction of sequencing biases due to the presence of reagent and laboratory contaminants affecting the analysis^42^. The results of this study are therefore to be interpreted with caution as no negative-controls (no-template controls) were included in the study using which such contaminants can be identified and removed^43^.

With respect to CAZYme profile, most of the previous studies have reported a high proportion of GH followed by GT and other classes of CAZymes similar to present study^14,28,44,45^. We also observed similar dominant organisms containing these CAZymes, i.e., members of Bacteroidetes, Firmicutes, Fibrobacter. However, unlike previous study on camel rumen, we didn't observe higher contributions from Spirochaetes (0.6% in our study compared to 4% in other study)^14^. In line with previous studies on rumen^14,28,45^, we observed comparable proportions of GH families acting as cellulases (GH5, GH9, GH88, GH95), hemicellulases (GH8, GH10, GH11, GH23, GH28, GH53), debranching enzymes (GH23, GH33, GH51, GH54, GH67, GH77, GH78, GH84, GH103, GH127) and oligosaccharide degrading enzymes (GH1, GH2, GH3, GH13, GH18, GH20, GH27, GH29, GH31, GH32, GH35, GH38, GH39, GH42, GH43, GH57, GH92, GH94, GH97, GH130). Also consistent in this study was the pattern of more abundant GH families. Amongst all, GH3 (β-Glucosidases), GH13 (α-Amylases), GH43 (arabino/xylosidases) and GH2 (β-Galactosidases) were the most dominant families observed as in other studies of rumen of camel^14^, cattle^18,45,46^, buffalo^47^ and moose^28^. The members of Bacteroidetes and Firmicutes were the major contributors for these four GH families, especially GH2, while more than 1% ORFs in GH13 and GH43 were coded by Fibrobacteres; and by Proteobacteria in GH3 and GH13. The contributions from Eukaryotes were also observed in GH3 (0.9%; 0.3% by *Neocallimastigaceae*, rest unclassified), GH43 (0.03%; entirely by *Neocallimastigaceae*) and GH13 (2%; 0.03% by *Neocallimastigaceae family* and 0.3% by *Eudiplodinium* genus). *Eudiplodinium* genus is a group of rumen ciliates belonging to family *ophryoscolecids* and have been linked with their cellulolytic and amylolytic activities^48,49^.

## Materials and methods

### Experimental design and sample collection

To access the dietary impact on the camel rumen microbiome, Kachchhi (K) and Bikaneri (B) breeds of camels were fed with three different diets, Bajra (B) (*Pennisetum glaucum*, pearl millet), Jowar (J) (*Sorghum bicolor*, sorghum) and Makai (M) (*Zea mays*, maize). The experimental animals were housed at the National Research Centre on Camel (NRCC), Bikaner, Rajasthan and provided ad libitum feed consumption and free access to drinking water. Twelve animals were divided into three groups (four animals in each group; two animals each of Bikaneri and Kachchhi breed) for 63 days (Figure S1). Prior to the experiment, all the animals were maintained on the same diet based on Guar (cluster bean, *Cyamopsis tetragonoloba*), different from the experimental diets. Rumen liquor samples were collected using probang as mentioned earlier^50^ under mild sedation. The samples were collected at 0 day before starting the feeding trial and subsequent collections were made on 10th, 21st, 42nd and 63rd days of experiment. We decided to collect the samples on every 21 days (21st, 42nd and 63rd) to cover the period of feed adaptation^37,38^ and intermediary collection during initial week on 10th day. Collected rumen content was filtered through four-layered sterile muslin cloth to separate the liquid and solid fractions to be collected in 2 ml cryovials prefilled with Qiagen RNAprotect Bacteria reagent (Qiagen, Germany) at an approximate 1:1 ratio. Samples were immediately stored at − 20 °C in a portable freezer and transported to the laboratory where these samples were stored at − 80 °C until further processing.

### Extraction of metagenomic DNA

Metagenomic DNA was isolated from liquid and solid fractions of rumen samples using QIAamp Fast DNA stool Mini Kit (Qiagen, Germany) following the manufacturer’s instructions with minor modifications. Briefly, liquid samples were subjected to bead beating in Qiagen TissueLyser for 30 s at 25 Hz and subsequently processed for lysis as per manufacturer’s instructions. Rumen solid samples were vortexed for 20 min to completely dissociate bacteria attached with feed particles followed by centrifugation at 2600 *g* for 30 s to separate solid particles. Approximately, 600 μl of supernatant was processed from the previous step for DNA isolation as recommended by the kit manufacturer. Quantity and quality of metagenomic DNA was assessed using a Qubit 3.0 fluorometer (ThermoFisher scientific, MA) and agarose gel electrophoresis, respectively.

### Library preparation and sequencing

V3-V4 hypervariable region of 16S rRNA gene was amplified using universal primer pair, 341F and 785R^51^ and library was prepared according to Illumina 16S Metagenomics library preparation guide (Illumina, USA). The final library size and concentration was checked using Agilent Bioanalyzer DNA 1000 chip (Agilent, USA) and Qubit fluorometer (Invitrogen, USA), respectively. Four sequencing runs were carried out using prepared libraries on Illumina MiSeq sequencer employing 2 × 250 v2 chemistry.

Shotgun metagenomic libraries were prepared from samples of collection 4 and 5 (n = 24). Libraries were prepared from 1 ng of metagenomic DNA with Nextera XT DNA Library Prep Kit (Illumina, USA) using the manufacturer’s protocol. Prepared libraries were quantified using Qubit 3.0 and checked for size on Agilent Bioanalyzer 2100 using DNA HS kit. Five sequencing runs were performed on Illumina MiSeq using 2 × 250 v2 sequencing chemistry to sequence all the metagenomic libraries.

### Data analysis

The raw data of amplicon sequencing was manually curated and quality filtered (average qual score < Q30 and trimming last 10 nucleotides from R2 reads) using Prinseq-lite Perl script^52^. The quality filtered data was then imported in the R v3.6.1 environment and analyzed with the DADA2 package v1.14.0^53^. As per DADA2 pipeline for 16S data (https://benjjneb.github.io/dada2/tutorial.html) and big data pipeline (https://benjjneb.github.io/dada2/bigdata.html), data from four runs was analyzed separately and then merged at a later stage. Briefly, the steps followed were quality check, trimming (primers were trimmed from both pairs) and filtering (no Ns and no PhiX), and sequence variants were inferred by estimating error rates and denoising. Sequence variants were merged across paired data and then data from all the runs were merged to construct the amplicon sequence variant (ASV) table followed by chimera/bimera removal and taxonomy assignment. GTDBr89 (Genome Taxonomy Database) database was used to assign taxonomy to the ASVs^22^. The ASV table, assigned taxonomy and related metadata were combined to create a phyloseq object using the Phyloseq R package v1.30.0^54^. Further, downstream analysis was done with the phyloseq object and using other R packages including microbiome v1.8.0^55^, vegan v2.5.6^56^, ggplot2 v3.2.1^57^, ggpubr v0.2.4^58^, UpSetR v1.4.0^59^. Observed ASVs and Shannon diversity were calculated and compared among groups. Further, between sample/groups comparison was done based on Bray–Curtis distance and visualized by plotting Non-metric multidimensional scaling (NMDS) plot followed by group level comparisons using PERMANOVA test. The Phylum and Genus level taxonomy was compared between groups to identify group specific differences. All the comparisons of diversity indices and taxa abundance across different groups were done using non-parametric Kruskal–Wallis (for multi-group comparisons) and Wilcoxon tests (for two-group comparison). The p-values were adjusted by Benjamini–Hochberg correction and have been mentioned accordingly throughout the manuscript. All the statistical testing between multiple groups were done using the R packages ggpubr and vegan.

The raw reads obtained from shotgun metagenomics were curated using Prinseq-lite Perl script with following parameters: minimum length = 50, length trimmed to = 190 (to remove G-biased tails; one of the runs with very poor tail-quality was trimmed to 150 nucleotides), and minimum average quality = 30. The quality filtered reads were analyzed using SqueezeMeta employing a co-assembly pipeline^60^. Within the pipeline, assembly was done using MetaSpades^61^, ORF prediction using MetaProdigal^62^, taxonomy assignment using Diamond^63^ against NCBI RefSeq database and functional prediction using Diamond/HMM against COG database^64^. Further, predicted ORFs were annotated for Carbohydrate Active Enzymes using HMMer based approach within dbCAN2^65,66^.

### Ethical permission

The work described in this article was carried out with prior ethical approval of the institutional animal ethics committee of the National Research Center on Camel, Bikaner, Rajasthan (NRCC/PSME/6(141)2000-Tech/). All procedures performed in studies involving animals were in accordance with the ethical standards of the institution or practice at which the studies were conducted. The work included non-invasive sample collection and no animals were harmed during the experiment. The study was carried out in compliance with the ARRIVE guidelines.

## Conclusion

In all, we report an extensive overview of camel rumen microbiota under influence of different diets. We observed the differences among three different feed roughages although the differences were not as much prominent as those reported in true ruminants. The study also tracked the microbiota diversity changes through time-points. We observed the highest number of significantly differentiating taxa in Collection-3 (21st day) with respect to Collection-1 (0 day). This points to the fact that on introduction of a new diet, microbiota starts changing slowly and more prominently during the third to sixth week and reaches a stable level thereafter. This was also observed in case of lesser functional differences between Collection-4 and Collection-5. However, the highest degree of variations were observed between two fractions of rumen content similar to that of previous studies of similar nature. We also observed a higher proportion of GH2, GH3, GH13 and GH43 CAZy families prominently involved in biomass degradation and reported in several rumen microbiota. Overall, this study presents important insights into camel rumen microbiome which can serve as critical information to increase feed digestibility in camels through selective enrichment of rumen microbiota.

## Supplementary Information

Supplementary Information

## Data Availability

All the raw sequencing data is submitted in NCBI under BioProject PRJNA603266 and available from SRA under accessions SRR13178665 to SRR13178784 for 16S data and SRR13205818 to SRR13205865 for Shotgun data. The R script used for analysis is available from github.com/ankit4035/camelrumenproject (https://doi.org/10.5281/zenodo.4308948) for reproduction of the entire work.
