# Supplementary material for: Characterizing rumen microbiota and CAZyme profile of Indian dromedary camel (Camelus dromedarius) in response to different roughages
Source: Sci Rep. 2021 Apr 30;11:9400. doi: 10.1038/s41598-021-88943-9 (PMC8087840; doi:10.1038/s41598-021-88943-9)
Supplement: Supplementary file 1 — Supplementary Information [file 41598_2021_88943_MOESM1_ESM.pdf]

Characterizing rumen microbiota and CAZyme profile of Indian dromedary camel (*Camelus dromedarius*) in response to different roughages

Ankit T. Hinsu[1], Nilam J. Tulsani[1], Ketankumar J. Panchal[1], Ramesh J. Pandit[1], Basanti Jyotsana[2], Nishant A. Dafale[3], Niteen V. Patil[2,3], Hemant J. Purohit[4], Chaitanya G. Joshi[1,5], Subhash J. Jakhesara[1#]

1 Department of Animal Biotechnology, College of Veterinary Science & A.H., Anand Agricultural University, Anand - 388001, India
2 ICAR-National Research Centre on Camel, Bikaner - 334001, India
3 ICAR-Central Arid Zone Research Institute, Jodhpur – 342003, India
4 Environmental Biotechnology and Genomics Division, CSIR-National Environmental Engineering Research Institute, Nagpur - 440020, India
5 Gujarat Biotechnology Research Centre, Gandhinagar - 382010, India

Ankit T. Hinsu: ankit4035hinsu@gmail.com
Nilam J. Tulsani: njtulsani@gmail.com
Ketankumar J. Panchal: ketan9589@gmail.com
Ramesh J. Pandit: panditrameshj@gmail.com
Basanti Jyotsana: bjyotsana@gmail.com
Nishant Dafale: na_dafale@neeri.res.in
Niteen V. Patil: nvpatil61@gmail.com
Hemant J. Purohit: hj_purohit@neeri.res.in
Chaitanya G. Joshi: cgjoshi@aau.in
Subhash J. Jakhesara: drsubhash81@gmail.com

# Address for Correspondence:
Subhash J. Jakhesara
Department of Animal Biotechnology,
College of Veterinary Science & A.H.,
Anand Agricultural University, Anand - 388001, India
E-mail: drsubhash81@gmail.com

Supplementary Information

Supplementary figures

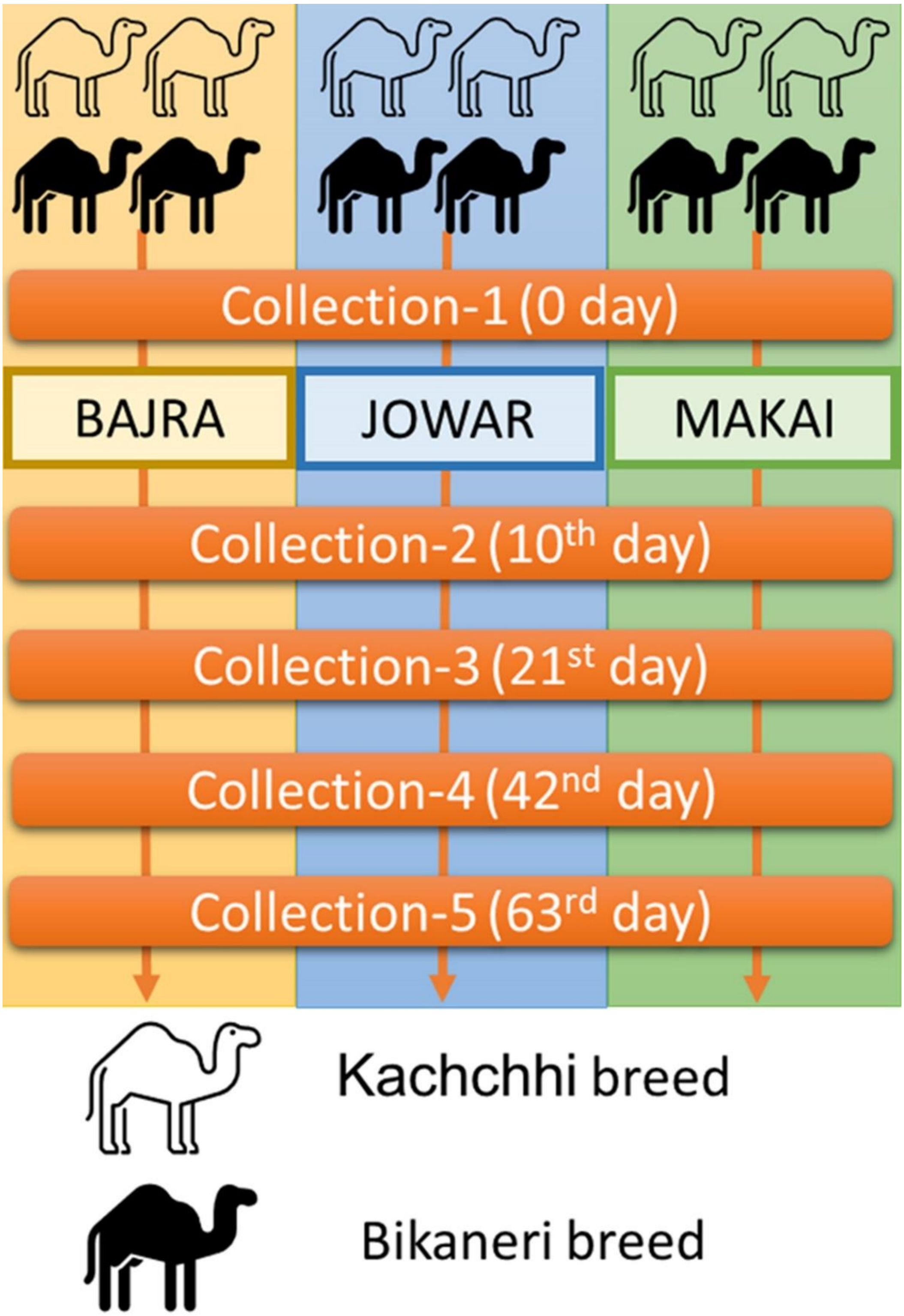

Figure S1: Schematics of experimental design.

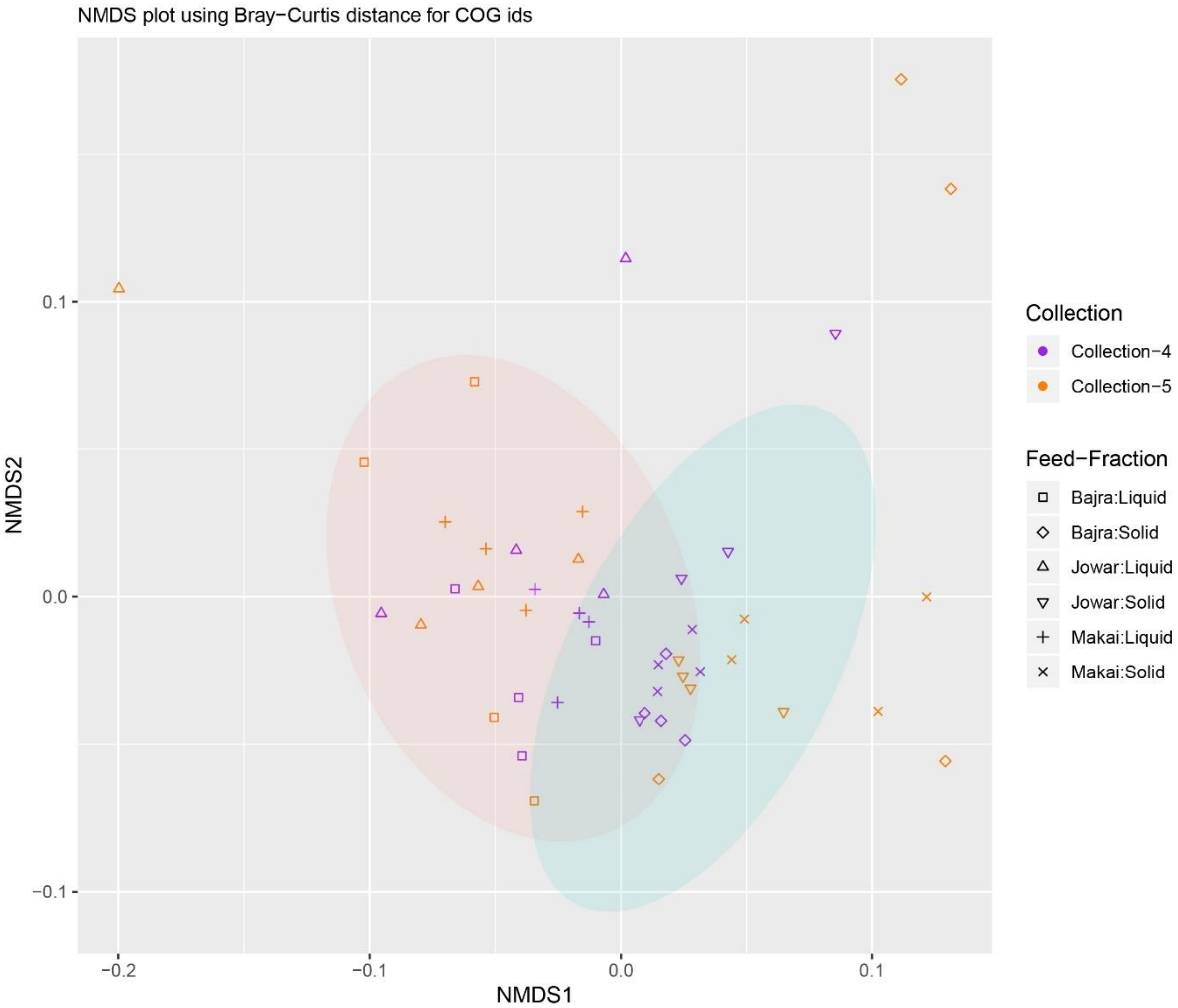

Figure S2: NMDS plot prepared using Bray-Curtis distance of all the COG ids. Plot is colored by Collections and shaped by feed-fraction group. The ellipses represent liquid and solid fractions.

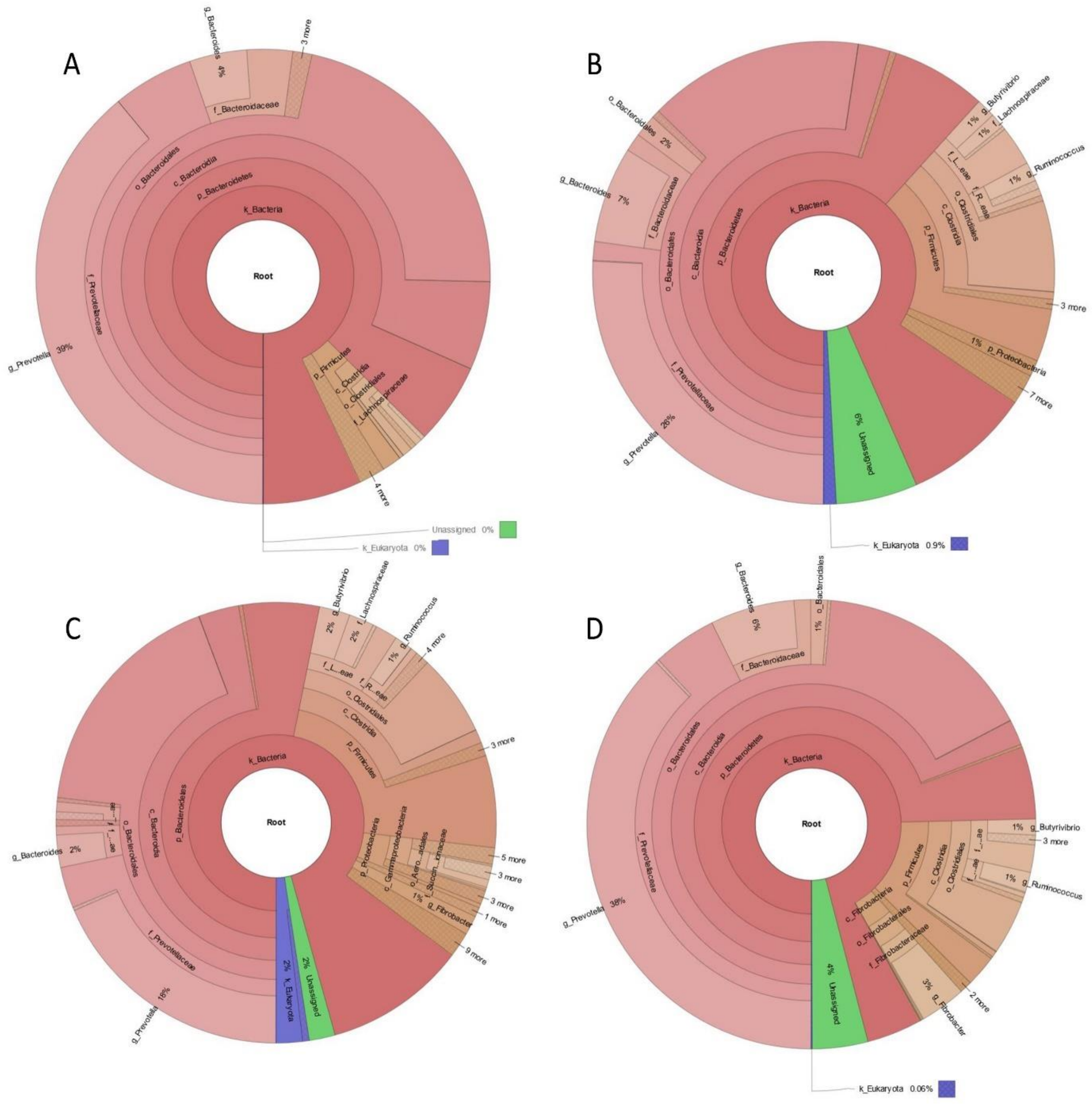

Figure S3: Krona chart plotting taxonomy of the genes coding for A. GH2, B. GH3, C. GH13 and D. GH43 families.

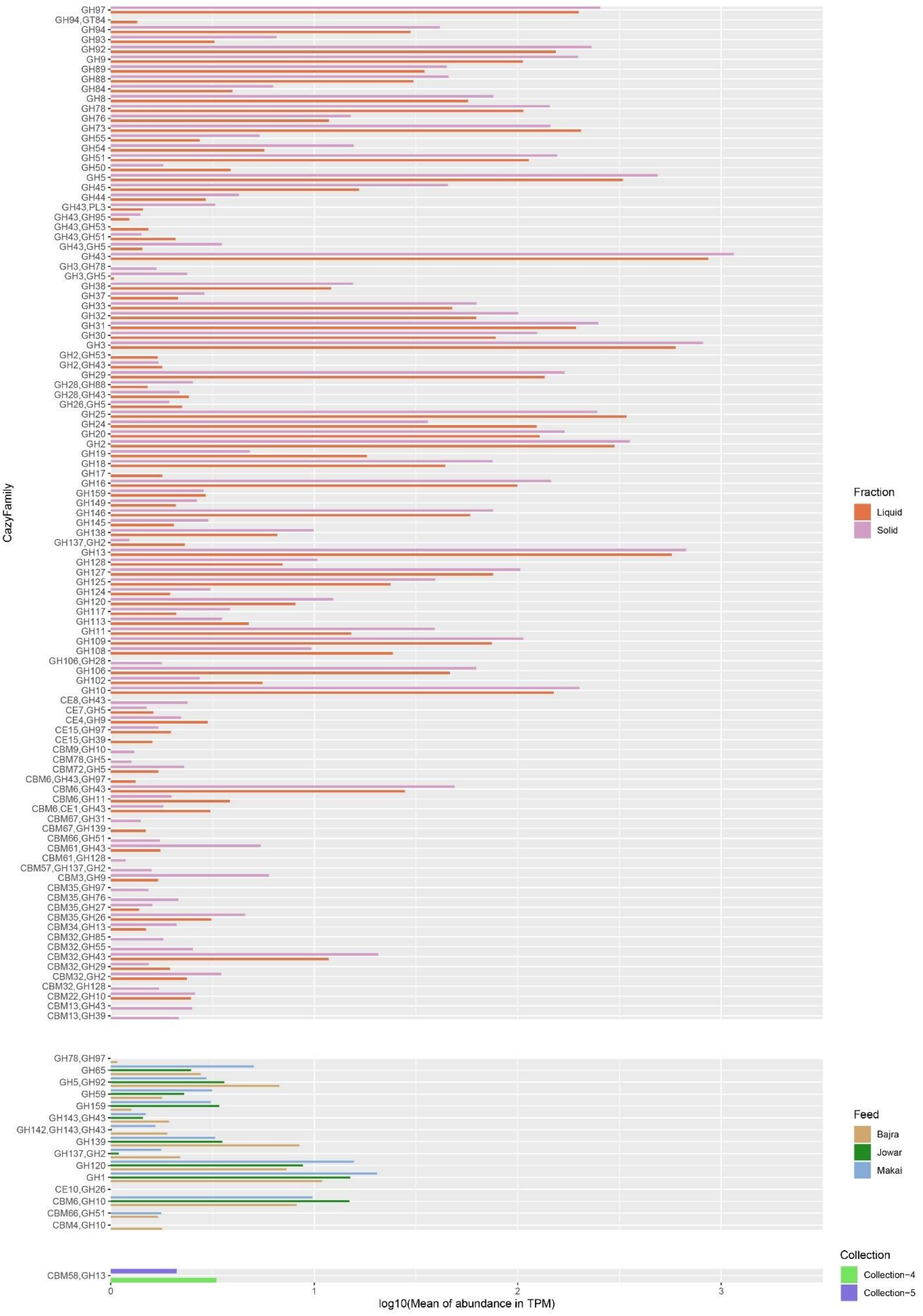

Figure S4: Bar chart showing the abundances of at least one GH-containing CAZyme categories significantly differing between fractions (top), feeds (middle) and collection (bottom) as per Kruskal-Wallis test.

Supplementary tables

Table S1: Statistics of 16S rRNA gene sequences generated and analyzed throughout the study. The mentioned numbers represents number of paired-reads.

Sample name	Breed	Feed	Collection	Collection day	Fraction	Raw reads	Prinseq-QC	DADA2-QC	Denoised-Forward	Denoised-Reverse	Merged	Non-chimeric / Analysed
**BB11L**	Bikaneri	Bajra	Collection-1	0 day	Liquid	413,273	325,098	325,097	323,104	322,884	314,979	299,705
**BB11S**	Bikaneri	Bajra	Collection-1	0 day	Solid	67,666	46,660	46,660	44,818	44,112	36,769	36,133
**BB12L**	Bikaneri	Bajra	Collection-2	10 day	Liquid	52,397	40,774	40,774	39,682	39,784	37,570	36,710
**BB12S**	Bikaneri	Bajra	Collection-2	10 day	Solid	100,804	67,963	67,963	66,167	65,598	58,685	57,774
**BB13L**	Bikaneri	Bajra	Collection-3	21 day	Liquid	75,478	55,760	55,759	53,744	53,832	48,215	47,461
**BB13S**	Bikaneri	Bajra	Collection-3	21 day	Solid	87,969	65,411	65,411	63,723	63,895	58,472	57,639
**BB14L**	Bikaneri	Bajra	Collection-4	42 day	Liquid	95,827	72,444	72,443	70,567	70,566	64,633	63,736
**BB14S**	Bikaneri	Bajra	Collection-4	42 day	Solid	67,762	50,194	50,194	48,612	48,526	43,355	42,692
**BB15L**	Bikaneri	Bajra	Collection-5	63 day	Liquid	57,735	47,638	47,636	45,779	44,929	37,037	36,254
**BB15S**	Bikaneri	Bajra	Collection-5	63 day	Solid	69,833	45,811	45,811	44,312	44,015	39,133	38,641
**BB21L**	Bikaneri	Bajra	Collection-1	0 day	Liquid	77,707	58,549	58,549	57,580	57,407	55,100	54,011
**BB21S**	Bikaneri	Bajra	Collection-1	0 day	Solid	69,195	51,400	51,400	50,186	50,269	47,802	47,357
**BB22L**	Bikaneri	Bajra	Collection-2	10 day	Liquid	69,073	54,354	54,354	53,076	52,956	50,204	49,236
**BB22S**	Bikaneri	Bajra	Collection-2	10 day	Solid	41,879	29,814	29,814	28,872	28,890	27,067	26,876
**BB23L**	Bikaneri	Bajra	Collection-3	21 day	Liquid	43,951	32,764	32,764	31,172	31,380	27,744	27,292
**BB23S**	Bikaneri	Bajra	Collection-3	21 day	Solid	98,120	73,093	73,093	71,438	70,862	63,925	62,925
**BB24L**	Bikaneri	Bajra	Collection-4	42 day	Liquid	104,528	78,067	78,066	76,075	76,147	69,579	68,583
**BB24S**	Bikaneri	Bajra	Collection-4	42 day	Solid	131,753	98,915	98,915	96,970	96,422	88,128	86,564
**BB25L**	Bikaneri	Bajra	Collection-5	63 day	Liquid	69,531	48,903	48,903	47,118	47,071	42,197	41,308
**BB25S**	Bikaneri	Bajra	Collection-5	63 day	Solid	46,016	36,495	36,494	35,047	33,477	26,264	26,017
**BJ11L**	Bikaneri	Jowar	Collection-1	0 day	Liquid	106,569	80,156	80,156	78,749	78,838	75,598	73,374
**BJ11S**	Bikaneri	Jowar	Collection-1	0 day	Solid	99,895	80,821	80,821	78,857	77,145	67,854	66,782
**BJ12L**	Bikaneri	Jowar	Collection-2	10 day	Liquid	44,811	34,879	34,879	33,886	34,029	32,174	31,749

**BJ12S**	Bikaneri	Jowar	Collection-2	10 day	Solid	64,055	42,711	42,706	41,549	41,570	39,187	38,411
**BJ13L**	Bikaneri	Jowar	Collection-3	21 day	Liquid	51,700	38,905	38,905	37,292	37,081	33,284	32,953
**BJ13S**	Bikaneri	Jowar	Collection-3	21 day	Solid	99,120	73,284	73,283	71,634	71,471	65,793	64,716
**BJ14L**	Bikaneri	Jowar	Collection-4	42 day	Liquid	95,173	68,947	68,946	67,170	67,016	61,284	60,673
**BJ14S**	Bikaneri	Jowar	Collection-4	42 day	Solid	84,407	61,382	61,382	59,681	59,744	54,567	53,673
**BJ15L**	Bikaneri	Jowar	Collection-5	63 day	Liquid	61,465	42,013	42,013	40,552	40,253	36,299	34,911
**BJ15S**	Bikaneri	Jowar	Collection-5	63 day	Solid	65,676	52,764	52,764	51,127	49,925	43,029	42,366
**BJ21L**	Bikaneri	Jowar	Collection-1	0 day	Liquid	42,882	32,621	32,621	31,626	31,655	29,627	28,925
**BJ21S**	Bikaneri	Jowar	Collection-1	0 day	Solid	71,258	48,035	48,035	46,194	41,511	33,258	32,373
**BJ22L**	Bikaneri	Jowar	Collection-2	10 day	Liquid	104,369	82,523	82,523	81,013	81,192	77,589	75,381
**BJ22S**	Bikaneri	Jowar	Collection-2	10 day	Solid	66,193	44,361	44,360	43,081	42,280	37,198	36,491
**BJ23L**	Bikaneri	Jowar	Collection-3	21 day	Liquid	93,269	70,363	70,363	68,605	68,338	62,488	61,509
**BJ23S**	Bikaneri	Jowar	Collection-3	21 day	Solid	70,094	48,776	48,776	47,365	47,462	40,864	39,987
**BJ24L**	Bikaneri	Jowar	Collection-4	42 day	Liquid	102,808	79,998	79,982	78,336	78,223	72,762	71,579
**BJ24S**	Bikaneri	Jowar	Collection-4	42 day	Solid	102,560	75,955	75,955	74,551	73,673	67,236	65,728
**BJ25L**	Bikaneri	Jowar	Collection-5	63 day	Liquid	59,893	41,847	41,847	40,720	40,689	37,121	34,824
**BJ25S**	Bikaneri	Jowar	Collection-5	63 day	Solid	39,181	33,156	33,155	31,741	30,947	26,027	25,668
**BM11L**	Bikaneri	Makai	Collection-1	0 day	Liquid	56,683	43,975	43,975	42,816	43,003	40,743	39,656
**BM11S**	Bikaneri	Makai	Collection-1	0 day	Solid	41,562	29,453	29,453	28,275	28,333	26,099	25,750
**BM12L**	Bikaneri	Makai	Collection-2	10 day	Liquid	23,491	17,638	17,633	16,800	16,821	15,180	14,818
**BM12S**	Bikaneri	Makai	Collection-2	10 day	Solid	71,806	56,960	56,959	54,878	54,037	46,518	44,552
**BM13L**	Bikaneri	Makai	Collection-3	21 day	Liquid	101,075	74,429	74,428	72,790	72,652	67,567	66,590
**BM13S**	Bikaneri	Makai	Collection-3	21 day	Solid	82,009	42,755	42,753	41,433	41,099	36,596	35,686
**BM14L**	Bikaneri	Makai	Collection-4	42 day	Liquid	80,974	60,282	60,282	58,585	58,473	53,330	52,561
**BM14S**	Bikaneri	Makai	Collection-4	42 day	Solid	104,975	77,777	77,777	76,111	75,746	68,278	67,166
**BM15L**	Bikaneri	Makai	Collection-5	63 day	Liquid	129,428	87,357	87,357	85,413	85,285	79,080	78,077
**BM15S**	Bikaneri	Makai	Collection-5	63 day	Solid	64,716	52,295	52,295	50,682	48,422	40,688	40,028

**BM21L**	Bikaneri	Makai	Collection-1	0 day	Liquid	24,370	19,626	19,624	18,313	17,789	14,037	13,627
**BM21S**	Bikaneri	Makai	Collection-1	0 day	Solid	41,228	30,007	30,007	29,110	29,145	27,489	27,232
**BM22L**	Bikaneri	Makai	Collection-2	10 day	Liquid	44,100	33,654	33,654	32,531	32,593	30,347	29,802
**BM22S**	Bikaneri	Makai	Collection-2	10 day	Solid	57,826	41,039	41,036	39,333	36,188	29,346	28,913
**BM23L**	Bikaneri	Makai	Collection-3	21 day	Liquid	246,775	174,787	174,787	172,856	172,190	165,723	162,141
**BM23S**	Bikaneri	Makai	Collection-3	21 day	Solid	65,053	48,027	48,027	46,740	46,577	42,371	41,749
**BM24L**	Bikaneri	Makai	Collection-4	42 day	Liquid	83,661	62,040	62,040	60,321	60,155	54,963	54,324
**BM24S**	Bikaneri	Makai	Collection-4	42 day	Solid	65,291	48,931	48,930	47,668	47,402	42,995	42,473
**BM25L**	Bikaneri	Makai	Collection-5	63 day	Liquid	96,040	66,156	66,155	64,580	64,055	58,467	57,666
**BM25S**	Bikaneri	Makai	Collection-5	63 day	Solid	390,516	269,453	269,452	264,843	263,357	242,529	237,593
**KB11L**	Kachchhi	Bajra	Collection-1	0 day	Liquid	39,574	28,119	28,106	27,149	27,189	25,381	24,765
**KB11S**	Kachchhi	Bajra	Collection-1	0 day	Solid	63,547	49,304	49,304	47,593	45,780	38,920	38,270
**KB12L**	Kachchhi	Bajra	Collection-2	10 day	Liquid	52,360	36,910	36,910	35,651	35,487	31,850	31,432
**KB12S**	Kachchhi	Bajra	Collection-2	10 day	Solid	38,071	27,496	27,496	26,705	26,718	25,351	24,970
**KB13L**	Kachchhi	Bajra	Collection-3	21 day	Liquid	61,154	46,025	46,025	44,556	44,471	40,047	39,551
**KB13S**	Kachchhi	Bajra	Collection-3	21 day	Solid	61,942	45,821	45,809	44,392	44,267	39,997	39,057
**KB14L**	Kachchhi	Bajra	Collection-4	42 day	Liquid	135,290	98,221	98,221	95,878	95,742	88,689	87,388
**KB14S**	Kachchhi	Bajra	Collection-4	42 day	Solid	108,447	81,413	81,413	79,649	78,758	71,233	69,822
**KB15L**	Kachchhi	Bajra	Collection-5	63 day	Liquid	100,551	67,372	67,370	65,687	65,911	60,427	59,410
**KB15S**	Kachchhi	Bajra	Collection-5	63 day	Solid	47,029	33,582	33,582	32,346	32,053	28,385	27,992
**KB21L**	Kachchhi	Bajra	Collection-1	0 day	Liquid	44,530	34,636	34,636	33,580	33,643	31,765	30,544
**KB21S**	Kachchhi	Bajra	Collection-1	0 day	Solid	47,858	35,499	35,499	34,329	34,533	32,305	31,798
**KB22L**	Kachchhi	Bajra	Collection-2	10 day	Liquid	76,588	59,752	59,752	58,319	58,447	55,489	54,165
**KB22S**	Kachchhi	Bajra	Collection-2	10 day	Solid	76,936	58,807	58,807	56,806	54,709	46,096	45,337
**KB23L**	Kachchhi	Bajra	Collection-3	21 day	Liquid	47,153	34,832	34,832	33,433	33,508	30,113	29,754
**KB23S**	Kachchhi	Bajra	Collection-3	21 day	Solid	71,617	46,644	46,644	45,160	44,962	40,467	39,543
**KB24L**	Kachchhi	Bajra	Collection-4	42 day	Liquid	85,903	66,713	66,713	65,246	64,963	59,983	58,610

**KB24S**	Kachchhi	Bajra	Collection-4	42 day	Solid	152,795	113,630	113,630	111,762	111,446	102,539	100,877
**KB25L**	Kachchhi	Bajra	Collection-5	63 day	Liquid	75,002	58,220	58,220	56,395	54,628	46,173	45,009
**KB25S**	Kachchhi	Bajra	Collection-5	63 day	Solid	74,503	50,134	50,134	48,661	48,355	43,736	43,159
**KJ11L**	Kachchhi	Jowar	Collection-1	0 day	Liquid	40,365	30,222	30,222	29,315	29,379	27,684	26,647
**KJ11S**	Kachchhi	Jowar	Collection-1	0 day	Solid	78,668	59,580	59,580	58,269	58,213	55,500	54,454
**KJ12L**	Kachchhi	Jowar	Collection-2	10 day	Liquid	135,191	92,914	92,914	89,779	89,584	77,322	75,196
**KJ12S**	Kachchhi	Jowar	Collection-2	10 day	Solid	73,343	48,569	48,569	47,148	46,671	41,589	40,647
**KJ13L**	Kachchhi	Jowar	Collection-3	21 day	Liquid	51,211	35,902	35,902	34,456	34,437	30,253	29,781
**KJ13S**	Kachchhi	Jowar	Collection-3	21 day	Solid	149,904	111,259	111,259	109,332	108,983	99,865	97,600
**KJ14L**	Kachchhi	Jowar	Collection-4	42 day	Liquid	81,565	61,109	61,108	59,204	59,265	53,511	52,900
**KJ14S**	Kachchhi	Jowar	Collection-4	42 day	Solid	75,209	53,733	53,733	52,118	52,079	46,389	45,555
**KJ15L**	Kachchhi	Jowar	Collection-5	63 day	Liquid	110,368	79,084	79,083	77,208	77,000	70,723	68,106
**KJ15S**	Kachchhi	Jowar	Collection-5	63 day	Solid	50,843	38,113	38,112	36,680	34,707	28,060	27,491
**KJ21L**	Kachchhi	Jowar	Collection-1	0 day	Liquid	70,500	54,596	54,595	53,414	53,528	51,031	48,780
**KJ21S**	Kachchhi	Jowar	Collection-1	0 day	Solid	59,033	46,192	46,181	44,388	42,781	34,913	34,341
**KJ22L**	Kachchhi	Jowar	Collection-2	10 day	Liquid	75,696	50,744	50,744	49,293	49,137	44,289	43,695
**KJ22S**	Kachchhi	Jowar	Collection-2	10 day	Solid	83,589	59,464	59,463	57,942	58,128	54,623	53,706
**KJ23L**	Kachchhi	Jowar	Collection-3	21 day	Liquid	71,118	50,898	50,880	48,783	48,857	42,770	41,935
**KJ23S**	Kachchhi	Jowar	Collection-3	21 day	Solid	115,293	81,358	81,357	79,427	79,426	73,315	72,104
**KJ24L**	Kachchhi	Jowar	Collection-4	42 day	Liquid	109,875	83,660	83,660	81,586	81,632	74,823	73,885
**KJ24S**	Kachchhi	Jowar	Collection-4	42 day	Solid	97,759	71,602	71,587	69,622	69,558	61,733	60,804
**KJ25L**	Kachchhi	Jowar	Collection-5	63 day	Liquid	71,869	50,366	50,366	48,864	48,590	44,167	42,941
**KJ25S**	Kachchhi	Jowar	Collection-5	63 day	Solid	82,585	54,993	54,993	53,471	52,539	46,288	45,595
**KM11L**	Kachchhi	Makai	Collection-1	0 day	Liquid	96,044	69,672	69,672	68,417	68,574	65,871	62,566
**KM11S**	Kachchhi	Makai	Collection-1	0 day	Solid	75,671	54,777	54,777	53,017	53,134	49,496	48,583
**KM12L**	Kachchhi	Makai	Collection-2	10 day	Liquid	66,077	55,058	55,058	53,051	52,969	44,872	44,302
**KM12S**	Kachchhi	Makai	Collection-2	10 day	Solid	41,769	29,765	29,765	28,780	28,918	27,061	26,629

**KM13L**	Kachchhi	Makai	Collection-3	21 day	Liquid	155,771	118,307	118,307	116,560	116,114	109,229	107,692
**KM13S**	Kachchhi	Makai	Collection-3	21 day	Solid	60,213	44,423	44,423	43,094	42,903	39,123	38,360
**KM14L**	Kachchhi	Makai	Collection-4	42 day	Liquid	79,606	60,947	60,947	59,334	59,191	54,804	53,739
**KM14S**	Kachchhi	Makai	Collection-4	42 day	Solid	76,010	57,918	57,918	56,392	56,202	51,197	50,026
**KM15L**	Kachchhi	Makai	Collection-5	63 day	Liquid	341,340	247,326	247,324	244,978	244,593	233,110	212,082
**KM15S**	Kachchhi	Makai	Collection-5	63 day	Solid	101,273	67,796	67,795	65,860	65,724	58,786	57,917
**KM21L**	Kachchhi	Makai	Collection-1	0 day	Liquid	48,505	38,473	38,473	37,334	37,462	35,193	34,033
**KM21S**	Kachchhi	Makai	Collection-1	0 day	Solid	52,055	38,137	38,137	36,929	36,874	34,404	33,873
**KM22L**	Kachchhi	Makai	Collection-2	10 day	Liquid	70,777	50,894	50,894	49,654	49,707	47,070	45,889
**KM22S**	Kachchhi	Makai	Collection-2	10 day	Solid	56,324	40,982	40,982	39,735	39,920	37,414	36,688
**KM23L**	Kachchhi	Makai	Collection-3	21 day	Liquid	76,970	57,080	57,080	55,350	55,138	50,104	49,366
**KM23S**	Kachchhi	Makai	Collection-3	21 day	Solid	150,942	112,724	112,724	110,611	110,250	99,232	96,909
**KM24L**	Kachchhi	Makai	Collection-4	42 day	Liquid	107,004	80,321	80,321	78,753	78,843	73,327	72,201
**KM24S**	Kachchhi	Makai	Collection-4	42 day	Solid	62,002	41,637	41,636	40,481	39,997	36,053	35,598
**KM25L**	Kachchhi	Makai	Collection-5	63 day	Liquid	119,333	84,737	84,736	82,971	82,888	76,960	75,448
**KM25S**	Kachchhi	Makai	Collection-5	63 day	Solid	62,073	47,396	47,369	45,643	43,715	36,637	35,968

Table S2: Fraction-wise comparison of taxa at Phylum and Genus level. BH = Benjamini-Hochberg. Taxa which are non-significant and had less abundance (Combined rel. abu. < 0.01) are not mentioned in the table. Suffix "_X" in genus name suggests that taxa was assigned at higher taxonomic levels than genus. Multiple suffixes are kept identifying exact level of assignment. % mean relative abundance is mentioned as mean ± standard deviation.

Taxa	% mean rel. abu.-Liquid	% mean rel. abu.-Solid	Kruskal-Wallis p-value	Kruskal-Wallis BH adjusted p-value
**Phylum**				
Acidobacteriota	0±0	0.004±0.02	0.0010	0.0018
Actinobacteriota	0.042±0.06	0.27±0.44	0.0000	0.0000
Bacteroidota	61.325±10.15	54.976±8.15	0.0006	0.0013
Bdellovibrionota	0.004±0.01	0.002±0	0.1317	0.1700
Campylobacterota	0.07±0.11	0.07±0.06	0.3772	0.4400
Chloroflexota	0.054±0.05	0.098±0.08	0.0003	0.0007
Cyanobacteria	2.485±1.29	0.752±0.61	0.0000	0.0000
Desulfobacterota	0.008±0.01	0.005±0.01	0.0078	0.0130
Desulfobacterota_A	0.02±0.02	0.096±0.06	0.0000	0.0000
Desulfuromonadota	0.002±0	0.013±0.02	0.0000	0.0000
Elusimicrobiota	0.495±0.3	0.347±0.24	0.0001	0.0002
Fibrobacterota	3.634±1.56	3.934±1.67	0.5780	0.6200
Firmicutes	2.021±1.02	1.813±1	0.1877	0.2300
Firmicutes_A	10.78±6.03	16.106±6.14	0.0000	0.0000
Firmicutes_B	0.003±0	0.033±0.06	0.0000	0.0000
Firmicutes_C	1.906±1.01	5.256±2.4	0.0000	0.0000
Firmicutes_I	0.005±0.01	0.004±0.01	0.6190	0.6400
Firmicutes_K	0.002±0.01	0.001±0	0.0752	0.1100
Fusobacteriota	0.221±0.24	1.198±1.38	0.0000	0.0000
Gemmatimonadota	0.001±0	0.002±0.01	0.1608	0.2000
Myxococcota	0.022±0.02	0.028±0.02	0.1067	0.1500
Patescibacteria	2.221±1.12	1.695±0.81	0.0012	0.0020
Planctomycetota	0.175±0.16	0.245±0.21	0.0360	0.0550
Proteobacteria	7.394±4.38	8.455±6.66	0.7290	0.7300
Riflebacteria	0.211±0.09	0.118±0.08	0.0000	0.0000
Spirochaetota	1.849±0.99	1.73±0.92	0.5461	0.6100
Synergistota	0.076±0.08	0.353±0.22	0.0000	0.0000
Unclassified Phylum	0.324±0.14	0.455±0.22	0.0009	0.0018
Verrucomicrobiota	4.652±1.69	1.943±1.42	0.0000	0.0000
**Genus**				
4C28d-15_X_X	0.066±0.08	0.019±0.04	0.0000	0.0000
992a	0.01±0.01	0.009±0.01	0.4700	0.5400
ABY1_X_X_X	0.001±0	0±0	0.0005	0.0015
Acetivibrionaceae_X	0.064±0.13	0.106±0.09	0.0000	0.0000
Acholeplasma_C	0.512±0.31	0.273±0.14	0.0000	0.0000
Acholeplasma_D	0.026±0.02	0.006±0.01	0.0000	0.0000

Acholeplasmatales_X_X	0.068±0.1	0.02±0.02	0.0003	0.0008
Achromobacter	0.016±0.04	0.002±0.01	0.0009	0.0022
Acidovorax_E	0.005±0.01	0±0	0.0005	0.0015
Acinetobacter	0.043±0.08	0.041±0.13	0.7147	0.7500
Actinobacillus	0.128±0.14	1.007±1.39	0.0000	0.0000
Actinobacillus_A	0.006±0.01	0.013±0.02	0.1158	0.1700
Actinomyces	0.001±0.01	0.022±0.05	0.0002	0.0005
Actinomycetaceae_X	0.002±0.01	0.013±0.02	0.0073	0.0150
Acutalibacteraceae_X	0.048±0.04	0.029±0.03	0.0068	0.0140
Aerococcaceae_X	0.009±0.02	0.15±0.18	0.0000	0.0000
Aerococcus	0.001±0.01	0.006±0.02	0.0031	0.0068
Agarilytica	0.007±0.02	0.002±0.01	0.0273	0.0460
Agitococcus	0.176±0.27	0.113±0.17	0.4663	0.5400
Akkermansia	0.005±0.02	0.015±0.07	0.4313	0.5000
Alcanivorax_A	0.05±0.1	0.007±0.02	0.0000	0.0002
Algoriphagus	0.001±0.01	0±0	0.0480	0.0760
Aliarcobacter	0.017±0.09	0±0	0.0036	0.0075
Alishewanella	0.321±0.71	0.123±0.25	0.0269	0.0450
Alphaproteobacteria_X_X_X	0.42±0.29	0.072±0.12	0.0000	0.0000
Anaerofilum	0.011±0.01	0.071±0.05	0.0000	0.0000
Anaerofustis	0±0	0.006±0.01	0.0000	0.0000
Anaerorhabdus	0.001±0	0.004±0.01	0.0290	0.0480
Anaerotignum	0.001±0	0.007±0.01	0.0000	0.0000
Anaerovibrio	0.008±0.01	0.005±0.01	0.1564	0.2100
Anaerovoracaceae_X	0.006±0.01	0.022±0.03	0.0057	0.0120
Aquabacterium	0.019±0.04	0.018±0.03	0.9581	0.9700
Arabia	0±0	0.002±0	0.0003	0.0008
Arenimonas	0.046±0.14	0.002±0.01	0.0000	0.0000
Aromatoleum	0.008±0.03	0.002±0.01	0.2339	0.3000
Bacilli_X_X_X	0.092±0.07	0.029±0.03	0.0000	0.0000
Bacillus	0.002±0.02	0.013±0.07	0.0558	0.0860
Bacillus_W	0.018±0.08	0.002±0.01	0.0913	0.1300
Bact-08	0.07±0.06	0.07±0.08	0.3094	0.3800
Bacteria_X_X_X_X_X	0.324±0.14	0.455±0.22	0.0009	0.0023
Bacteroidaceae_X	3.954±1.41	5.453±1.97	0.0000	0.0000
Bacteroidales_X_X	10.835±2.49	11.947±2.53	0.0051	0.0110
Bacteroides	0.04±0.04	0.23±0.3	0.0001	0.0003
Bacteroidia_X_X_X	0.432±0.21	0.535±0.23	0.0055	0.0110
Beijerinckiaceae_X	0.005±0.03	0.023±0.13	0.2397	0.3100
Bibersteinia	0.201±0.2	0.157±0.17	0.2112	0.2800
Blastomonas	0.005±0.01	0±0	0.0006	0.0017
Blautia_A	0.002±0.01	0.113±0.22	0.0000	0.0000
Bosea	0.002±0.01	0±0	0.0036	0.0075
Brevundimonas	0.052±0.09	0.035±0.06	0.4917	0.5600

Burkholderiaceae_X	0.084±0.09	0.021±0.04	0.0000	0.0000
Butyrivibrio	0.507±0.5	0.518±0.4	0.5219	0.5900
C941	0.15±0.16	0.894±0.53	0.0000	0.0000
CAG-180	0.003±0.01	0.007±0.01	0.0171	0.0300
CAG-312	0.009±0.02	0.002±0.01	0.0127	0.0230
CAG-313	0.059±0.04	0.018±0.02	0.0000	0.0000
CAG-354	0.574±0.42	0.684±0.4	0.0900	0.1300
CAG-462	3.052±0.93	4.487±1.63	0.0000	0.0000
CAG-465	0.009±0.02	0.003±0.02	0.0357	0.0590
CAG-475	0.008±0.01	0.002±0	0.0000	0.0000
CAG-495	0.587±0.39	0.222±0.14	0.0000	0.0000
CAG-826_X	0.346±0.25	0.131±0.12	0.0000	0.0000
CAG-873	0.003±0.01	0.021±0.05	0.0447	0.0720
CAG-878	0.113±0.09	0.06±0.05	0.0020	0.0048
Campylobacter	0.015±0.02	0.018±0.03	0.7656	0.8000
Campylobacter_A	0.033±0.04	0.04±0.06	0.5639	0.6300
Campylobacteraceae_X	0.005±0.01	0.009±0.02	0.2008	0.2700
Caviibacter	0.005±0.01	0.015±0.03	0.0438	0.0710
Cellvibrio	0.033±0.05	0.012±0.03	0.0031	0.0068
Cellvibrionaceae_X	0.004±0.02	0.002±0.01	0.0374	0.0610
Chishuiella	0.007±0.01	0.009±0.02	0.8637	0.8900
Christensenellales_X_X	0.001±0	0.023±0.03	0.0000	0.0000
Chryseobacterium	0.032±0.06	0.009±0.03	0.0155	0.0280
Cloacibacterium	0.02±0.03	0.092±0.14	0.0000	0.0000
Clostridia_X_X_X	1.71±0.93	3.172±1.86	0.0000	0.0000
Clostridium	0.013±0.04	0.025±0.05	0.0194	0.0330
Clostridium_A	0±0	0.01±0.01	0.0000	0.0000
Clostridium_M	0.005±0.01	0±0	0.0067	0.0130
Clostridium_N	0.023±0.04	0.006±0.02	0.0031	0.0068
Comamonas	0.018±0.03	0.004±0.01	0.0027	0.0062
Corynebacterium	0±0	0.006±0.02	0.0259	0.0440
CP2B	0±0	0.002±0.01	0.0077	0.0150
Denitrobacterium	0±0	0.002±0	0.0008	0.0021
Dermatophilaceae_X	0.009±0.03	0.031±0.05	0.0002	0.0007
Desulfobacterota_X_X_X_X	0.008±0.01	0.005±0.01	0.0078	0.0150
Desulfovibrio	0±0	0.005±0.02	0.0079	0.0150
Desulfovibrionaceae_X	0.005±0.01	0.046±0.03	0.0000	0.0000
Devosia	0.007±0.02	0.01±0.02	0.4677	0.5400
Dialister_A	0.001±0	0.004±0.01	0.0031	0.0068
Dichelobacter	0.049±0.05	0.06±0.06	0.3062	0.3800
Dietzia	0.001±0	0.006±0.01	0.0061	0.0120
Dongia	0.017±0.03	0.003±0.01	0.0001	0.0005
Dorea	0±0	0.001±0	0.0036	0.0075
DTU053	0.014±0.02	0.092±0.08	0.0000	0.0000

Echinicola	0.012±0.02	0.01±0.02	0.2104	0.2800
Eggerthellaceae_X	0.001±0	0.016±0.02	0.0000	0.0000
Emergencia	0±0	0.004±0.01	0.0017	0.0042
Empedobacter	0.012±0.02	0.018±0.03	0.5786	0.6400
Endomicrobium	0.011±0.02	0.008±0.02	0.3334	0.4000
Endomicrobium_A	0.104±0.09	0.159±0.11	0.0032	0.0070
Enterobacterales_X_X	0.173±0.22	0.07±0.08	0.0000	0.0000
Enterobacteriaceae_X	0.418±0.38	0.311±0.23	0.3395	0.4100
Enterococcaceae_X	0.006±0.02	0.022±0.03	0.0000	0.0001
Enterococcus	0.015±0.04	0.022±0.05	0.9975	1.0000
Erysipelothrix	0.029±0.03	0.078±0.09	0.0000	0.0001
Erysipelotrichaceae_X	0.005±0.01	0.017±0.02	0.0000	0.0000
Erysipelotrichales_X_X	0.001±0	0±0	0.0144	0.0260
Eubacterium_C	0.006±0.01	0±0	0.0010	0.0024
Eubacterium_E	0.065±0.05	0.078±0.07	0.4995	0.5700
Eubacterium_F	0.146±0.12	0.25±0.15	0.0000	0.0000
Eubacterium_Q	0.091±0.09	0.146±0.12	0.0164	0.0290
Eubacterium_S	0.021±0.02	0.057±0.04	0.0000	0.0000
F0040	0.278±0.26	2.172±2.86	0.0000	0.0000
F0058	0.002±0.01	0.024±0.03	0.0000	0.0000
F0422	0.025±0.07	0.406±0.77	0.0000	0.0000
F0428	0.026±0.02	0.115±0.07	0.0000	0.0000
F082	1.972±1.14	0.263±0.44	0.0000	0.0000
FD2005	0.209±0.19	0.195±0.13	0.9540	0.9700
Fermentimonas	0.003±0.01	0.03±0.06	0.0009	0.0022
Fibrobacter	3.553±1.55	3.92±1.67	0.3529	0.4300
Fibrobacter_A	0.081±0.07	0.014±0.02	0.0000	0.0000
Firm-04	0.008±0.01	0.091±0.07	0.0000	0.0000
Firm-16	0.004±0.01	0.02±0.04	0.0005	0.0013
Firmicutes_A_X_X_X_X	0.004±0.01	0.014±0.01	0.0000	0.0000
Firmicutes_B_X_X_X_X	0.003±0	0.033±0.06	0.0000	0.0000
Flavobacteriaceae_X	0.294±0.27	0.161±0.14	0.0012	0.0029
Flavobacteriales_X_X	0.044±0.05	0.07±0.06	0.0003	0.0008
Flavobacterium	0.116±0.23	0.023±0.05	0.0002	0.0005
Flavobacterium_A	0.07±0.15	0.01±0.02	0.0008	0.0022
Flexilinea	0.054±0.05	0.098±0.08	0.0003	0.0009
Fodinicurvata	0.003±0.01	0±0	0.0203	0.0350
Frateuria	0.002±0.01	0±0	0.0036	0.0075
Fusicatenibacter	0.037±0.04	0.02±0.03	0.0334	0.0550
Fusobacterium_A	0.075±0.09	0.459±0.56	0.0000	0.0000
Fusobacterium_C	0.123±0.13	0.705±0.95	0.0000	0.0000
Gammaproteobacteria_X_X_X	0.364±0.34	0.155±0.12	0.0000	0.0000
Gastranaerophilaceae_X	0.018±0.03	0.004±0.01	0.0030	0.0068
GCA-900066135	0±0	0.002±0.01	0.0124	0.0230

GCA-900066905	0.004±0.01	0.013±0.02	0.0116	0.0220
GCA-900066995	0.05±0.15	0.112±0.2	0.0095	0.0180
Gemmobacter_A	0.014±0.05	0.006±0.02	0.1535	0.2100
Geothermobacter	0.002±0	0.013±0.02	0.0000	0.0000
GN02-873	0.011±0.02	0.014±0.03	0.5253	0.5900
GWE2-31-10	0.011±0.02	0.007±0.01	0.0621	0.0950
GWF2-44-16	0.005±0.01	0±0	0.0000	0.0000
Helcococcaceae_X	0±0	0.009±0.02	0.0000	0.0001
Helcococcus	0.005±0.01	0.015±0.02	0.0007	0.0019
Hepatobacter	0.034±0.08	0.003±0.01	0.0000	0.0001
Hungatella_A	0.488±0.42	0.431±0.35	0.5637	0.6300
Inquilinus	0.006±0.04	0.1±0.6	0.2295	0.3000
Kapabacteriaceae_X	0.002±0.01	0.007±0.01	0.0034	0.0073
Kingella	0.068±0.09	0.584±0.93	0.0000	0.0000
Kiritimatiellae_X_X_X	0.476±0.21	0.156±0.14	0.0000	0.0000
KLE1796	0±0	0.004±0.01	0.0001	0.0004
Kocuria	0.005±0.04	0.076±0.43	0.0940	0.1400
Lachnoanaerobaculum	0.012±0.02	0.073±0.07	0.0000	0.0000
Lachnospira	0.013±0.03	0.011±0.02	0.7768	0.8100
Lachnospiraceae_X	3.273±2.03	4.263±1.78	0.0020	0.0047
Lachnospirales_X_X	0.06±0.05	0.097±0.05	0.0000	0.0001
Lactobacillales_X_X	0.001±0	0.016±0.03	0.0001	0.0002
Lactococcus	0.007±0.01	0.021±0.04	0.0524	0.0820
Lactonifactor	0.004±0.01	0.01±0.02	0.0128	0.0230
Lancefieldella	0±0	0.007±0.01	0.0000	0.0002
Lawsonibacter	0.003±0.01	0±0	0.0036	0.0075
Leaf454	0.101±0.14	0.129±0.21	0.3073	0.3800
Lenti-01	0.117±0.11	0.095±0.09	0.2669	0.3400
Lentimicrobium	0.663±0.29	0.439±0.18	0.0000	0.0000
Leptotrichiaceae_X	0.001±0	0.006±0.01	0.0100	0.0190
Leuconostoc	0.01±0.02	0.041±0.09	0.2010	0.2700
Lysobacter	0.002±0.01	0.015±0.08	0.2449	0.3100
Mailhella	0.014±0.02	0.045±0.03	0.0000	0.0000
Mannheimia	0.022±0.04	0.249±0.41	0.0000	0.0000
Massilia_B	0.067±0.2	0.006±0.03	0.2935	0.3600
Massilibacteroides	0.114±0.09	0.165±0.17	0.3778	0.4500
Metamycoplasma	0.002±0.01	0.004±0.01	0.0116	0.0220
Monoglobales_X_X	0.005±0.01	0±0	0.0010	0.0025
Monoglobus	0.07±0.06	0.052±0.03	0.1488	0.2000
Moraxella	0.556±0.9	1.183±1.6	0.0001	0.0003
Moraxella_A	0.001±0	0.004±0.01	0.0381	0.0620
Moraxella_C	0±0	0.047±0.14	0.0003	0.0008
Moraxellaceae_X	0.11±0.13	0.055±0.07	0.0030	0.0068
Mucilaginibacter	0.003±0.01	0±0	0.0021	0.0048

Muribaculaceae_X	0.201±0.2	1.887±1.3	0.0000	0.0000
Ndongobacter	0±0	0.005±0.01	0.0009	0.0023
Negativicutes_X_X_X	0.107±0.06	0.039±0.04	0.0000	0.0000
Neisseria_B	0.004±0.01	0.01±0.02	0.0098	0.0190
Neisseriaceae_X	0.036±0.04	0.111±0.09	0.0000	0.0000
Neorhizobium	0.018±0.06	0.09±0.48	0.7486	0.7800
Niveispirillum	0.007±0.02	0±0	0.0127	0.0230
OEMR01	0.001±0	0.034±0.07	0.0000	0.0000
OLB17	0±0	0.004±0.02	0.0010	0.0025
Olegusella	0±0	0.007±0.02	0.0074	0.0150
Olsenella	0.011±0.02	0.059±0.07	0.0000	0.0002
Opitutaceae_X	1.295±0.91	0.827±0.74	0.0005	0.0014
Opitutales_X_X	0.001±0	0±0	0.0495	0.0780
Opitutus	0.006±0.02	0±0	0.0010	0.0025
Oribacterium	0.021±0.03	0.004±0.01	0.0000	0.0000
Orrella	0.012±0.03	0.002±0.01	0.0020	0.0047
Oscillibacter	0.015±0.02	0.029±0.04	0.0271	0.0460
Oscillospiraceae_X	0.57±0.57	0.791±0.49	0.0006	0.0016
Oscillospirales_X_X	0.257±0.15	0.211±0.11	0.1267	0.1800
Paeniclostridium	0±0	0.003±0.01	0.0001	0.0003
PALSA-1355	0.008±0.01	0.01±0.02	0.5481	0.6100
Paludibacteraceae_X	0.26±0.23	0.075±0.11	0.0000	0.0000
Paramesorhizobium	0.004±0.01	0.02±0.1	0.7171	0.7500
Paramuribaculum	0.003±0.01	0.013±0.02	0.0008	0.0022
Parapedobacter	0.003±0.01	0.001±0.01	0.0031	0.0068
Paraprevotella	0.209±0.19	0.27±0.21	0.0735	0.1100
Parvibaculum	0.096±0.18	0.067±0.1	0.4001	0.4700
Parvimonas	0.006±0.01	0.037±0.05	0.0000	0.0000
Pasteurellaceae_X	0.599±0.72	2.502±3.09	0.0000	0.0000
Pauljensenia	0±0	0.012±0.03	0.0001	0.0003
Pedosphaeraceae_X	0.186±0.17	0.131±0.12	0.1627	0.2200
PeH17	0.111±0.09	0.384±0.19	0.0000	0.0000
Peptostreptococcaceae_X	0.002±0	0.042±0.06	0.0000	0.0000
Peptostreptococcales_X_X	0.039±0.06	0.17±0.17	0.0000	0.0000
Phycorickettsia	0.001±0	0±0	0.0135	0.0250
Pigmentiphaga	0.048±0.15	0.002±0.01	0.0000	0.0000
Pirellulaceae_X	0.001±0	0.002±0.01	0.0401	0.0650
Pirellulales_X_X	0.133±0.15	0.186±0.2	0.1868	0.2500
Planctomycetes_X_X_X	0.01±0.02	0.011±0.02	0.6729	0.7300
Planctomycetota_X_X_X_X	0.023±0.04	0.032±0.04	0.0096	0.0180
Porphyromonas	0.342±0.37	1.136±1.14	0.0000	0.0002
Prevotella	27.316±10.33	17.309±4.79	0.0000	0.0000
Prevotellamassilia	0.198±0.09	0.263±0.14	0.0184	0.0320
Prolixibacteraceae_X	0.506±0.4	1.589±0.98	0.0000	0.0000

Prosthecobacter	0.018±0.06	0.014±0.04	0.7067	0.7500
Proteiniclasticum	0.021±0.04	0.038±0.07	0.1204	0.1700
Proteobacteria_X_X_X_X	0.235±0.23	0.119±0.16	0.0000	0.0001
Pseudaminobacter	0.004±0.01	0.015±0.03	0.0031	0.0068
Pseudomonadaceae_X	0.092±0.29	0.055±0.26	0.0148	0.0260
Pseudomonas_A	0.203±0.32	0.082±0.12	0.0199	0.0340
Pseudomonas_D	0.051±0.14	0.001±0	0.0000	0.0000
Pseudomonas_E	0.039±0.07	0.001±0	0.0000	0.0000
Pseudomonas_M	0.137±0.24	0.02±0.07	0.0001	0.0002
Pygmaiobacter	0.007±0.01	0.048±0.09	0.0000	0.0000
Pyramidobacter	0.067±0.07	0.331±0.2	0.0000	0.0000
RC9	4.568±2.37	2.979±0.91	0.0000	0.0000
RF16	4.421±1.79	1.172±0.91	0.0000	0.0000
Rhizobiaceae_X	0.009±0.04	0.059±0.32	0.5035	0.5700
Rhizobiales_X_X	0.01±0.02	0.003±0.01	0.0012	0.0029
Rhodospirillaceae_X	0.011±0.02	0.001±0	0.0000	0.0001
Roseomonas	0.008±0.01	0±0	0.0000	0.0001
RUG131	0.225±0.15	0.09±0.05	0.0000	0.0000
RUG163	0.452±0.42	0.483±0.32	0.1431	0.2000
RUG350	0.155±0.09	0.105±0.21	0.0000	0.0000
Ruminiclostridium	0.192±0.17	0.143±0.11	0.2796	0.3500
Ruminiclostridium_C	0.364±0.63	0.742±0.48	0.0000	0.0000
Ruminiclostridium_D	0.017±0.01	0.005±0.01	0.0000	0.0000
Ruminiclostridium_F	0.012±0.02	0.005±0.02	0.0161	0.0290
Ruminococcaceae_X	0.102±0.12	0.313±0.21	0.0000	0.0000
Ruminococcus	0.077±0.08	0.192±0.23	0.0010	0.0025
Ruminococcus_C	0.047±0.05	0.078±0.08	0.0141	0.0260
Ruminococcus_D	0.359±0.3	0.483±0.35	0.0450	0.0720
Ruminococcus_F	0.075±0.07	0.219±0.19	0.0000	0.0000
Saccharicrinis	0.009±0.02	0.01±0.02	0.9246	0.9400
Saccharimonadaceae_X	0.169±0.13	0.089±0.07	0.0006	0.0017
Saccharimonadales_X_X	0.052±0.06	0.007±0.02	0.0000	0.0000
Saccharofermentans	0.158±0.12	0.316±0.21	0.0000	0.0000
Schwartzia	0.359±0.2	0.161±0.11	0.0000	0.0000
Selenomonadaceae_X	0.129±0.12	0.032±0.04	0.0000	0.0000
Selenomonas_B	0.016±0.02	0.006±0.02	0.0176	0.0310
Serratia	0.008±0.02	0.002±0.01	0.0334	0.0550
Shinella	0.012±0.07	0.011±0.02	0.0107	0.0200
Simonsiella	0.025±0.05	0.078±0.12	0.0004	0.0012
Sneathia	0.017±0.06	0.006±0.02	0.7033	0.7500
Soleaferrea	0.059±0.08	0.107±0.12	0.0490	0.0780
Sphaerochaeta	0.03±0.04	0.004±0.01	0.0000	0.0000
Sphaerochaeta_A	0.021±0.02	0.014±0.02	0.0229	0.0390
Sphaerochaetaceae_X	0.155±0.14	0.128±0.09	0.6404	0.7000

Sphingobacterium	0.1±0.14	0.011±0.02	0.0001	0.0004
Sphingomonas	0.033±0.05	0.01±0.02	0.0122	0.0230
Sphingopyxis_A	0.006±0.02	0.019±0.08	0.4917	0.5600
Spirochaetia_X_X_X	1.176±0.73	1.07±0.63	0.5152	0.5800
Spirochaetota_X_X_X_X	0.019±0.02	0.038±0.03	0.0016	0.0038
Stenotrophomonas	0.834±1.34	0.093±0.14	0.0001	0.0002
Streptococcus	0.092±0.23	0.571±0.82	0.0000	0.0000
Succiniclasticum	1.231±0.85	4.292±2.28	0.0000	0.0000
Succinimonas	0.23±0.18	0.116±0.14	0.0000	0.0000
Succinivibrio	0.275±0.24	0.046±0.04	0.0000	0.0000
Synergistales_X_X	0.008±0.01	0.01±0.01	0.2352	0.3000
Synergistes	0±0	0.005±0.01	0.0000	0.0000
SZUA-359	0.411±0.36	0.24±0.29	0.0017	0.0041
Tannerella	0.001±0	0.005±0.01	0.0098	0.0190
Tannerellaceae_X	0.021±0.02	0.038±0.04	0.0440	0.0710
TF01-11	0.012±0.02	0.052±0.08	0.0000	0.0000
Tissierellales_X_X	0.055±0.05	0.166±0.09	0.0000	0.0000
Treponema_A	0.001±0.01	0.009±0.04	0.6677	0.7200
Treponema_B	0.005±0.01	0.033±0.06	0.0003	0.0008
Treponema_C	0.01±0.02	0.011±0.02	0.6526	0.7100
Treponema_D	0.093±0.05	0.071±0.05	0.0049	0.0100
Treponemataceae_X	0.196±0.13	0.216±0.15	0.4934	0.5600
Treponematales_X_X	0.071±0.07	0.127±0.11	0.0002	0.0006
UBA1020	0.005±0.02	0.001±0	0.0160	0.0280
UBA1033	0.037±0.13	0.053±0.11	0.0347	0.0570
UBA1067	2.011±0.84	0.539±0.45	0.0000	0.0000
UBA1174	0.077±0.1	0.036±0.12	0.0000	0.0000
UBA1191	0.004±0.01	0.023±0.03	0.0000	0.0000
UBA1258	0.189±0.16	0.266±0.15	0.0032	0.0070
UBA1361	0.09±0.07	0.078±0.05	0.8193	0.8400
UBA1394	0.004±0.01	0.026±0.03	0.0000	0.0000
UBA1436	0.304±0.22	0.144±0.12	0.0000	0.0000
UBA1532	0.02±0.02	0.025±0.02	0.1180	0.1700
UBA1547	0.004±0.01	0±0	0.0144	0.0260
UBA1711	0.348±0.22	0.208±0.14	0.0000	0.0001
UBA1829	0.434±0.43	0.139±0.17	0.0000	0.0000
UBA2450	0.068±0.14	0.029±0.04	0.0002	0.0006
UBA3206	0.133±0.12	0.025±0.03	0.0000	0.0000
UBA4179	0.056±0.11	0.038±0.06	0.4116	0.4800
UBA5124	1.514±0.78	1.332±0.69	0.1306	0.1900
UBA5194	0.005±0.01	0.009±0.02	0.2602	0.3300
UBA5946	0.007±0.02	0±0	0.0019	0.0045
UBA636	0.002±0	0.008±0.02	0.0189	0.0330
UBA6984	0.021±0.02	0±0	0.0000	0.0000

UBA7182	0.042±0.16	0.008±0.02	0.9387	0.9600
UBA733	0.081±0.06	0.009±0.01	0.0000	0.0000
UBA7862	0.081±0.06	0.019±0.02	0.0000	0.0000
UBA8416	0.049±0.03	0.014±0.02	0.0000	0.0000
UBA8525	0.061±0.07	0.003±0.01	0.0000	0.0000
UBA8953	0.211±0.09	0.118±0.08	0.0000	0.0000
UBA932_X	0.071±0.07	0.114±0.11	0.0846	0.1300
UBA9983_A_X_X	0.04±0.12	0.004±0.01	0.0084	0.0160
V5-8f	0±0	0.013±0.04	0.0037	0.0078
Varibaculum_A	0±0	0.004±0.01	0.0008	0.0022
Veillonella	0.017±0.06	0.095±0.21	0.0000	0.0000
Veillonella_A	0.013±0.04	0.215±0.44	0.0000	0.0000
Verrucomicrobiota_X_X_X_X	0.002±0.01	0±0	0.0036	0.0075
Victivallis	0.037±0.03	0.009±0.02	0.0000	0.0000
Williamwhitmania	0.028±0.03	0.191±0.22	0.0000	0.0000
Xanthomonas_A	0.006±0.01	0±0	0.0003	0.0008
Xanthomonas_B	0.041±0.06	0.013±0.02	0.0039	0.0082
Zag1	2.358±1.25	0.616±0.56	0.0000	0.0000
Zag111	0.086±0.06	0.13±0.12	0.0746	0.1100

Table S3: Feed-wise (among feed) comparison of taxa at Phylum and Genus level within Liquid fraction and Solid fraction samples. Taxa which are non-significant and had less abundance (Combined rel. abu. < 0.01) are not mentioned in the table. Suffix "_X" in genus name suggests that taxa was assigned at higher taxonomic levels than genus. Multiple suffixes are kept identifying exact level of assignment. % mean relative abundance is mentioned as mean ± standard deviation.

	Liquid fraction					Solid fraction				
Taxa	% mean rel. abu. - Bajra	% mean rel. abu. - Jowar	% mean rel. abu. - Makai	Kruskal-Wallis p-value	Kruskal-Wallis BH adjusted p-value	% mean rel. abu. - Bajra	% mean rel. abu. - Jowar	% mean rel. abu. - Makai	Kruskal-Wallis p-value	Kruskal-Wallis BH adjusted p-value
Phylum										
Acidobacteriota	0±0	0±0	0±0	NA	NA	0.004±0.01	0.007±0.02	0.001±0	0.8487	0.9300
Actinobacteriota	0.046±0.06	0.042±0.04	0.037±0.07	0.3870	0.5700	0.359±0.72	0.307±0.16	0.143±0.14	0.0086	0.0590
Bacteroidota	60.781±7.76	61.917±12.31	61.276±10.37	0.9151	0.9200	56.417±8.59	53.863±7.46	54.648±8.55	0.5428	0.7500
Bdellovibrionota	0.001±0	0.006±0.01	0.005±0.01	0.2728	0.5500	0.001±0	0.003±0.01	0.002±0	0.5291	0.7500
Campylobacterota	0.075±0.06	0.066±0.07	0.07±0.17	0.1078	0.3800	0.108±0.07	0.062±0.05	0.038±0.04	0.0013	0.0260
Chloroflexota	0.044±0.03	0.055±0.04	0.062±0.07	0.8319	0.8700	0.078±0.07	0.096±0.08	0.119±0.08	0.1685	0.3800
Cyanobacteria	2.666±1.12	2.302±1.34	2.487±1.44	0.3513	0.5600	0.561±0.28	0.768±0.39	0.926±0.92	0.1370	0.3300
Desulfobacterota	0.009±0.01	0.008±0.01	0.008±0.01	0.8414	0.8700	0.005±0.01	0.004±0.01	0.005±0.01	0.8964	0.9300
Desulfobacterota_A	0.024±0.02	0.019±0.03	0.017±0.02	0.1367	0.4300	0.089±0.05	0.1±0.07	0.098±0.06	0.7054	0.8500
Desulfuromonadota	0.002±0	0.001±0	0.002±0	0.3565	0.5600	0.016±0.02	0.008±0.01	0.013±0.02	0.6399	0.8100
Elusimicrobiota	0.577±0.3	0.548±0.36	0.361±0.17	0.0223	0.1200	0.342±0.22	0.284±0.09	0.415±0.33	0.5434	0.7500
Fibrobacterota	3.308±1.05	3.576±1.9	4.018±1.61	0.2253	0.5300	2.932±0.73	4.39±1.92	4.479±1.69	0.0018	0.0260
Firmicutes	2.049±1.38	2.218±0.87	1.797±0.69	0.2695	0.5500	1.945±1.16	1.877±0.99	1.616±0.85	0.6136	0.8100
Firmicutes_A	9.845±4.89	10.703±6.05	11.792±7.09	0.7013	0.8500	14.565±6.22	17.089±4.93	16.665±7.07	0.2890	0.5600
Firmicutes_B	0.006±0.01	0.001±0	0.001±0	0.0068	0.0960	0.039±0.04	0.026±0.03	0.035±0.09	0.3769	0.6400
Firmicutes_C	2.049±1.11	1.818±0.94	1.85±1.01	0.7532	0.8500	4.344±1.64	5.718±2.86	5.705±2.4	0.1107	0.2900
Firmicutes_I	0.005±0.01	0±0	0.011±0.02	0.0011	0.0310	0.005±0.02	0.001±0	0.007±0.01	0.2683	0.5600
Firmicutes_K	0.003±0.01	0.001±0	0.001±0.01	0.3626	0.5600	0±0	0.001±0	0.001±0	0.9989	1.0000
Fusobacteriota	0.259±0.21	0.252±0.32	0.152±0.18	0.2180	0.5300	1.25±1.29	1.136±1.19	1.209±1.7	0.8790	0.9300
Gemmatimonadota	0.001±0	0.002±0	0±0	0.5063	0.6800	0.002±0.01	0.003±0	0.002±0	0.3084	0.5600
Myxococcota	0.021±0.02	0.023±0.02	0.021±0.03	0.4061	0.5700	0.019±0.02	0.037±0.03	0.027±0.02	0.0808	0.2300
Patescibacteria	2.151±0.96	2.385±0.81	2.128±1.51	0.2209	0.5300	1.691±0.67	1.68±0.9	1.716±0.88	0.8903	0.9300
Planctomycetota	0.165±0.15	0.114±0.1	0.247±0.2	0.0521	0.2400	0.192±0.13	0.176±0.15	0.368±0.27	0.0143	0.0590
Proteobacteria	8.024±5.35	7.684±4.7	6.473±2.75	0.7606	0.8500	10.76±8.02	8.206±5.03	6.399±6.15	0.0687	0.2200

Riflebacteria	0.205±0.09	0.254±0.07	0.174±0.11	0.0163	0.1100	0.083±0.06	0.136±0.09	0.136±0.08	0.0261	0.0950
Spirochaetota	1.721±0.77	1.458±0.38	2.368±1.36	0.0694	0.2800	1.29±0.56	1.669±0.69	2.23±1.18	0.0103	0.0590
Synergistota	0.06±0.05	0.076±0.08	0.091±0.1	0.6216	0.7900	0.25±0.1	0.419±0.18	0.39±0.29	0.0080	0.0590
Unclassified Phylum	0.32±0.14	0.288±0.13	0.365±0.16	0.3436	0.5600	0.471±0.28	0.418±0.2	0.477±0.17	0.4440	0.7200
Verrucomicrobiota	5.585±1.89	4.183±1.44	4.186±1.35	0.0142	0.1100	2.18±0.82	1.517±0.66	2.13±2.2	0.0124	0.0590
**Genus**										
32-67-11	0.002±0.01	0.005±0.01	0.002±0.01	0.6855	0.9100	0±0	0.001±0	0.002±0.01	0.3555	0.8100
4C28d-15_X_X	0.046±0.06	0.11±0.1	0.042±0.04	0.0153	0.1800	0.023±0.06	0.022±0.02	0.011±0.01	0.1299	0.6200
992a	0.01±0.01	0.009±0.01	0.01±0.01	0.8197	0.9500	0.007±0.02	0.014±0.02	0.006±0.01	0.0290	0.4000
Acetivibrionaceae_X	0.074±0.17	0.044±0.06	0.074±0.14	0.8839	0.9700	0.07±0.05	0.091±0.06	0.158±0.13	0.0190	0.3900
Acetobacteraceae_X	0.004±0.01	0±0	0.015±0.02	0.0065	0.1100	0.005±0.01	0.001±0	0.003±0.01	0.4806	0.8800
Acholeplasma_C	0.544±0.39	0.525±0.3	0.466±0.21	0.9784	1.0000	0.251±0.1	0.333±0.16	0.235±0.14	0.0709	0.5500
Acholeplasma_D	0.029±0.03	0.028±0.02	0.021±0.02	0.5043	0.8700	0.002±0.01	0.008±0.01	0.007±0.01	0.1884	0.7500
Acholeplasmatales_X_X	0.079±0.12	0.081±0.12	0.044±0.05	0.6875	0.9100	0.025±0.03	0.015±0.02	0.02±0.02	0.7064	0.9500
Achromobacter	0.008±0.02	0.022±0.04	0.017±0.04	0.3497	0.7100	0±0	0.004±0.01	0.001±0.01	0.1628	0.7000
Acidovorax_E	0.002±0.01	0.006±0.02	0.007±0.01	0.0828	0.5300	0±0	0±0	0±0	NA	NA
Acinetobacter	0.034±0.08	0.001±0	0.095±0.09	0.0000	0.0046	0.068±0.22	0.017±0.04	0.037±0.04	0.1462	0.6800
Actinobacillus	0.18±0.17	0.101±0.08	0.103±0.15	0.0513	0.3700	1.734±2	0.787±0.85	0.501±0.63	0.0241	0.4000
Actinobacillus_A	0.009±0.02	0.004±0.01	0.007±0.01	0.7660	0.9500	0.011±0.02	0.01±0.02	0.017±0.03	0.9764	1.0000
Actinomyces	0.003±0.01	0.001±0	0±0	0.4061	0.7600	0.032±0.06	0.02±0.04	0.014±0.04	0.1871	0.7500
Actinomycetaceae_X	0.002±0.01	0.003±0.01	0.002±0	0.4533	0.8000	0.015±0.03	0.02±0.03	0.005±0.01	0.0209	0.3900
Acutalibacteraceae_X	0.048±0.03	0.044±0.05	0.051±0.05	0.5891	0.8800	0.031±0.03	0.043±0.04	0.012±0.02	0.0136	0.3800
Aerococcaceae_X	0.01±0.02	0.005±0.01	0.012±0.03	0.7641	0.9500	0.122±0.15	0.204±0.24	0.124±0.13	0.5535	0.9000
Aerococcus	0.003±0.01	0±0	0±0	0.1308	0.6000	0.006±0.02	0.012±0.02	0.001±0	0.1575	0.7000
Agarilytica	0.006±0.02	0.014±0.03	0.001±0	0.1126	0.6000	0±0	0.004±0.01	0.003±0.01	0.3675	0.8100
Agitococcus	0.257±0.32	0.207±0.26	0.064±0.17	0.0024	0.0530	0.135±0.19	0.151±0.21	0.054±0.06	0.4446	0.8500
Akkermansia	0.01±0.03	0.005±0.01	0.001±0	0.5103	0.8800	0.03±0.11	0.013±0.03	0.003±0.01	0.8430	0.9600
Alcanivorax_A	0.063±0.15	0.042±0.05	0.044±0.08	0.5655	0.8800	0.009±0.01	0.003±0.01	0.01±0.02	0.6084	0.9000
Aliarcobacter	0.011±0.03	0.006±0.02	0.033±0.15	0.8660	0.9700	0±0	0±0	0±0	NA	NA
Alishewanella	0.375±0.89	0.49±0.83	0.098±0.08	0.7202	0.9400	0.113±0.25	0.172±0.33	0.082±0.1	0.6211	0.9100
Alphaproteobacteria_X_X_X	0.446±0.33	0.495±0.3	0.318±0.22	0.0865	0.5400	0.057±0.04	0.063±0.05	0.096±0.21	0.9246	0.9900

Anaerofilum	0.01±0.01	0.01±0.01	0.015±0.02	0.6678	0.9000	0.062±0.04	0.07±0.04	0.08±0.06	0.7276	0.9500
Anaerofustis	0.001±0	0±0	0±0	0.7686	0.9500	0.004±0.01	0.007±0.01	0.007±0.01	0.3043	0.8100
Anaerorhabdus	0.001±0	0±0	0.001±0	0.6107	0.8800	0.006±0.01	0.003±0.01	0.002±0	0.3347	0.8100
Anaerosporobacter	0±0	0±0	0.001±0	0.1308	0.6000	0.003±0.01	0.001±0	0.011±0.03	0.4935	0.8800
Anaerotignum	0.001±0	0±0	0.001±0	0.7713	0.9500	0.004±0.01	0.008±0.01	0.008±0.01	0.2192	0.7600
Anaerovibrio	0.005±0.01	0.008±0.01	0.01±0.01	0.5188	0.8800	0.004±0.01	0.007±0.01	0.003±0.01	0.4103	0.8400
Anaerovoracaceae_X	0.008±0.01	0.007±0.01	0.004±0.01	0.4446	0.8000	0.027±0.04	0.025±0.03	0.014±0.02	0.5378	0.9000
Aquabacterium	0.018±0.04	0.024±0.03	0.014±0.04	0.6386	0.8900	0.014±0.02	0.021±0.04	0.017±0.03	0.8843	0.9700
Arenimonas	0.07±0.21	0.031±0.07	0.038±0.1	0.5852	0.8800	0.001±0	0.002±0.01	0.002±0.01	0.4806	0.8800
Aromatoleum	0.008±0.02	0.01±0.03	0.007±0.02	0.6691	0.9000	0.002±0	0±0	0.004±0.01	0.5779	0.9000
Bacilli_X_X_X	0.091±0.06	0.109±0.08	0.078±0.06	0.3229	0.7100	0.023±0.02	0.036±0.03	0.027±0.02	0.3434	0.8100
Bacillus	0±0	0±0	0.006±0.03	0.3679	0.7100	0.031±0.13	0.008±0.02	0±0	0.1953	0.7500
Bacillus_AD	0.001±0	0±0	0±0	0.6012	0.8800	0.015±0.06	0.01±0.04	0.001±0	0.9922	1.0000
Bacillus_W	0.01±0.04	0.037±0.12	0.008±0.03	0.7863	0.9500	0.005±0.02	0±0	0±0	0.3679	0.8100
Bact-08	0.065±0.05	0.052±0.04	0.092±0.08	0.0993	0.5900	0.068±0.09	0.056±0.08	0.085±0.07	0.2428	0.7600
Bacteria_X_X_X_X_X	0.32±0.14	0.288±0.13	0.365±0.16	0.3436	0.7100	0.471±0.28	0.418±0.2	0.477±0.17	0.4440	0.8500
Bacteroidaceae_X	4.063±1.71	3.528±1.13	4.271±1.3	0.2032	0.7100	5.151±2.39	5.607±1.73	5.602±1.8	0.3361	0.8100
Bacteroidales_X_X	11.087±1.75	10.962±2.48	10.454±3.12	0.3832	0.7200	11.692±2.93	11.76±2.35	12.388±2.32	0.5428	0.9000
Bacteroides	0.057±0.05	0.039±0.05	0.023±0.03	0.0136	0.1800	0.325±0.29	0.21±0.28	0.156±0.3	0.1231	0.6200
Bacteroidia_X_X_X	0.384±0.14	0.477±0.24	0.435±0.22	0.6019	0.8800	0.498±0.21	0.559±0.2	0.547±0.26	0.6248	0.9100
Beijerinckiaceae_X	0±0	0.003±0.01	0.012±0.05	0.6012	0.8800	0.053±0.22	0.015±0.04	0±0	0.2282	0.7600
Bibersteinia	0.222±0.17	0.23±0.22	0.15±0.21	0.1659	0.6700	0.17±0.19	0.17±0.18	0.132±0.16	0.8138	0.9500
Blastomonas	0.003±0.01	0±0	0.013±0.01	0.0001	0.0093	0±0	0±0	0.001±0	0.3679	0.8100
Blautia_A	0.002±0.01	0±0	0.004±0.01	0.1298	0.6000	0.128±0.25	0.116±0.23	0.096±0.18	0.7940	0.9500
Brachymonas	0.001±0	0.001±0	0.004±0.01	0.4138	0.7600	0.008±0.02	0.005±0.01	0.005±0.02	0.7273	0.9500
Brevundimonas	0.052±0.1	0.049±0.09	0.054±0.1	0.8514	0.9600	0.038±0.08	0.028±0.03	0.04±0.07	0.7743	0.9500
BRH-c57	0.005±0.01	0.005±0.02	0.003±0.01	0.8769	0.9700	0±0	0±0	0.001±0	0.3555	0.8100
Burkholderiaceae_X	0.077±0.09	0.078±0.1	0.096±0.09	0.6134	0.8800	0.011±0.02	0.026±0.05	0.024±0.05	0.3945	0.8300
Butyrivibrio	0.416±0.39	0.515±0.51	0.589±0.6	0.6313	0.8900	0.365±0.31	0.558±0.38	0.631±0.46	0.1253	0.6200
C941	0.113±0.1	0.124±0.09	0.212±0.23	0.2535	0.7100	0.772±0.39	0.937±0.5	0.972±0.68	0.7256	0.9500
CAG-180	0±0	0.006±0.01	0.004±0.01	0.0124	0.1800	0.008±0.01	0.007±0.01	0.006±0.01	0.7110	0.9500

CAG-312	0.008±0.02	0.017±0.03	0.003±0	0.3044	0.7100	0.002±0.01	0.004±0.01	0.001±0	0.1940	0.7500
CAG-313	0.055±0.03	0.068±0.04	0.053±0.04	0.2638	0.7100	0.014±0.02	0.019±0.02	0.02±0.02	0.7231	0.9500
CAG-354	0.577±0.4	0.638±0.44	0.507±0.44	0.6114	0.8800	0.558±0.31	0.879±0.4	0.613±0.41	0.0276	0.4000
CAG-462	2.939±0.69	2.858±0.83	3.359±1.15	0.2375	0.7100	4.747±1.61	4.46±1.28	4.254±1.98	0.3754	0.8100
CAG-465	0.009±0.02	0.008±0.02	0.011±0.02	0.9230	0.9800	0.002±0	0.006±0.03	0.002±0	0.6972	0.9500
CAG-475	0.006±0.01	0.012±0.01	0.007±0.01	0.3660	0.7100	0.001±0	0.002±0	0.002±0	0.6585	0.9200
CAG-495	0.647±0.32	0.675±0.53	0.44±0.26	0.0663	0.4600	0.198±0.1	0.256±0.15	0.212±0.16	0.3020	0.8100
CAG-826_X	0.32±0.22	0.401±0.27	0.316±0.26	0.4538	0.8000	0.152±0.13	0.131±0.12	0.11±0.1	0.4892	0.8800
CAG-873	0.003±0.01	0.001±0	0.004±0.01	0.5687	0.8800	0.027±0.07	0.015±0.03	0.022±0.06	0.9547	0.9900
CAG-878	0.104±0.09	0.12±0.1	0.116±0.09	0.8605	0.9600	0.047±0.04	0.069±0.05	0.063±0.06	0.3937	0.8300
Campylobacter	0.012±0.02	0.019±0.02	0.013±0.03	0.3643	0.7100	0.022±0.03	0.018±0.03	0.014±0.02	0.9564	0.9900
Campylobacter_A	0.042±0.04	0.034±0.05	0.023±0.03	0.2657	0.7100	0.072±0.08	0.031±0.04	0.016±0.02	0.0942	0.5800
Campylobacter_B	0.001±0	0.001±0	0±0	0.7718	0.9500	0.004±0.01	0.004±0.01	0.001±0	0.2251	0.7600
Campylobacteraceae_X	0.008±0.02	0.005±0.01	0±0	0.1830	0.7100	0.01±0.02	0.01±0.02	0.007±0.01	0.9310	0.9900
Caviibacter	0.007±0.01	0.003±0.01	0.006±0.02	0.3039	0.7100	0.012±0.03	0.011±0.03	0.021±0.04	0.8550	0.9600
Cellvibrio	0.029±0.04	0.051±0.07	0.019±0.04	0.5734	0.8800	0.007±0.01	0.022±0.05	0.008±0.01	0.4877	0.8800
Cellvibrionaceae_X	0.002±0.01	0.009±0.03	0.001±0	0.8749	0.9700	0±0	0.003±0.01	0.002±0.01	0.7718	0.9500
Chishuiella	0.006±0.01	0.001±0	0.014±0.02	0.0016	0.0490	0.003±0.01	0±0	0.022±0.02	0.0001	0.0330
Christensenellales_X_X	0.001±0	0.002±0	0.001±0	0.3595	0.7100	0.026±0.03	0.023±0.03	0.02±0.03	0.5752	0.9000
Chryseobacterium	0.025±0.07	0.003±0.01	0.067±0.07	0.0001	0.0098	0.013±0.03	0±0	0.014±0.03	0.1787	0.7400
Chryseobacterium_D	0.009±0.02	0.003±0.01	0±0	0.2802	0.7100	0.003±0.01	0.001±0	0±0	0.1845	0.7500
Cloacibacterium	0.026±0.03	0.017±0.02	0.016±0.02	0.4486	0.8000	0.12±0.21	0.086±0.09	0.069±0.09	0.4602	0.8700
Clostridia_X_X_X	1.653±0.64	1.713±0.82	1.764±1.27	0.9053	0.9800	3.232±2.41	3.109±0.89	3.177±2.04	0.5531	0.9000
Clostridium	0.022±0.05	0.004±0.01	0.013±0.04	0.1932	0.7100	0.025±0.04	0.027±0.05	0.024±0.07	0.2408	0.7600
Clostridium_A	0±0	0±0	0±0	0.6012	0.8800	0.005±0.01	0.011±0.02	0.013±0.02	0.2130	0.7600
Clostridium_M	0.008±0.02	0.004±0.01	0.001±0.01	0.2998	0.7100	0±0	0±0	0±0	NA	NA
Clostridium_N	0.037±0.06	0.027±0.03	0.004±0.01	0.0239	0.2300	0.013±0.03	0.005±0.01	0.001±0	0.2019	0.7600
Comamonas	0.013±0.03	0.001±0	0.039±0.04	0.0001	0.0093	0±0	0.003±0.01	0.008±0.01	0.0093	0.3300
Corynebacterium	0±0	0±0	0±0	0.3679	0.7100	0.01±0.03	0.002±0.01	0.006±0.02	0.5574	0.9000
Cytophagales_X_X	0.013±0.03	0.001±0	0.002±0	0.5643	0.8800	0.005±0.02	0.003±0	0±0	0.1193	0.6200
Dermatophilaceae_X	0.018±0.04	0.005±0.01	0.005±0.02	0.1263	0.6000	0.032±0.05	0.036±0.06	0.025±0.05	0.6361	0.9100

Desulfobacterota_X_X_X_X	0.009±0.01	0.008±0.01	0.008±0.01	0.8414	0.9500	0.005±0.01	0.004±0.01	0.005±0.01	0.8964	0.9700
Desulfovibrio	0.001±0	0±0	0±0	0.1308	0.6000	0.007±0.02	0.001±0	0.008±0.02	0.4177	0.8400
Desulfovibrionaceae_X	0.004±0.01	0.007±0.01	0.005±0.01	0.9839	1.0000	0.046±0.03	0.044±0.03	0.046±0.04	0.8943	0.9700
Devosia	0.003±0.01	0.01±0.01	0.007±0.03	0.0478	0.3500	0.013±0.02	0.012±0.02	0.006±0.02	0.4078	0.8400
Dialister_A	0±0	0±0	0.001±0	0.6010	0.8800	0.005±0.01	0.003±0	0.005±0.01	0.8551	0.9600
Dichelobacter	0.056±0.05	0.055±0.05	0.035±0.04	0.3643	0.7100	0.056±0.06	0.071±0.07	0.052±0.05	0.6937	0.9500
Dietzia	0±0	0.001±0	0.001±0.01	0.9989	1.0000	0.007±0.02	0.008±0.02	0.003±0.01	0.4896	0.8800
Dongia	0.017±0.03	0.025±0.04	0.009±0.01	0.7574	0.9500	0.002±0.01	0.003±0.01	0.003±0.01	0.5905	0.9000
DTU053	0.015±0.02	0.011±0.02	0.014±0.02	0.7309	0.9500	0.109±0.09	0.105±0.07	0.063±0.07	0.0845	0.5600
Duncaniella	0.001±0	0.002±0.01	0.001±0.01	0.9958	1.0000	0±0	0.014±0.04	0.005±0.01	0.1126	0.6200
Dysgonomonadaceae_X	0.004±0.01	0.011±0.05	0.004±0.01	0.7232	0.9400	0.003±0.01	0±0	0.008±0.03	0.3492	0.8100
Echinicola	0.012±0.02	0.015±0.02	0.011±0.01	0.8220	0.9500	0.008±0.01	0.013±0.02	0.009±0.01	0.9881	1.0000
Eggerthellaceae_X	0±0	0.001±0	0.002±0	0.3677	0.7100	0.013±0.02	0.021±0.03	0.013±0.02	0.6975	0.9500
Elizabethkingia	0.002±0	0.004±0.01	0.003±0.01	0.9172	0.9800	0±0	0±0	0.002±0	0.3718	0.8100
Emergencia	0.001±0	0±0	0±0	0.3555	0.7100	0.004±0.01	0.006±0.01	0.001±0	0.2440	0.7600
Empedobacter	0.012±0.03	0.001±0	0.023±0.02	0.0003	0.0140	0.011±0.02	0.005±0.02	0.037±0.04	0.0077	0.3300
Endomicrobium	0.023±0.04	0.004±0.01	0.006±0.01	0.3158	0.7100	0.008±0.02	0.005±0.01	0.012±0.04	0.5905	0.9000
Endomicrobium_A	0.111±0.07	0.092±0.11	0.109±0.09	0.2487	0.7100	0.176±0.12	0.13±0.11	0.169±0.12	0.3489	0.8100
Enterobacterales_X_X	0.105±0.05	0.173±0.13	0.241±0.34	0.3007	0.7100	0.054±0.05	0.078±0.1	0.08±0.09	0.7665	0.9500
Enterobacteriaceae_X	0.415±0.41	0.42±0.33	0.419±0.42	0.9306	0.9900	0.303±0.25	0.353±0.22	0.276±0.23	0.4289	0.8500
Enterococcaceae_X	0.001±0	0.006±0.01	0.009±0.03	0.6300	0.8900	0.022±0.03	0.023±0.03	0.021±0.03	0.8952	0.9700
Enterococcus	0.008±0.03	0.002±0.01	0.035±0.05	0.0001	0.0098	0.019±0.05	0.008±0.03	0.039±0.06	0.0030	0.2900
Erysipelothrix	0.022±0.02	0.042±0.05	0.022±0.02	0.4163	0.7700	0.057±0.05	0.106±0.12	0.07±0.08	0.4873	0.8800
Erysipelotrichaceae_X	0.005±0.01	0.005±0.01	0.005±0.01	0.5558	0.8800	0.019±0.02	0.016±0.02	0.017±0.02	0.6007	0.9000
Eubacterium_C	0.008±0.01	0.006±0.02	0.003±0.01	0.1612	0.6600	0±0	0±0	0.001±0	0.1308	0.6200
Eubacterium_E	0.061±0.04	0.062±0.06	0.074±0.06	0.7758	0.9500	0.048±0.04	0.088±0.08	0.098±0.07	0.0504	0.5100
Eubacterium_F	0.115±0.06	0.177±0.15	0.146±0.13	0.5718	0.8800	0.258±0.17	0.251±0.13	0.239±0.14	0.9708	1.0000
Eubacterium_Q	0.088±0.09	0.063±0.06	0.123±0.1	0.0479	0.3500	0.122±0.13	0.119±0.08	0.196±0.14	0.0726	0.5500
Eubacterium_S	0.019±0.02	0.018±0.02	0.027±0.03	0.6642	0.9000	0.047±0.04	0.049±0.04	0.076±0.05	0.0643	0.5500
F0040	0.402±0.27	0.223±0.23	0.209±0.24	0.0164	0.1800	3.39±3.46	2.049±2.92	1.077±1.41	0.1489	0.6800
F0058	0.003±0.01	0.003±0.01	0.001±0	0.5998	0.8800	0.028±0.03	0.029±0.04	0.016±0.03	0.1431	0.6700

F0422	0.057±0.12	0.007±0.02	0.01±0.02	0.0281	0.2600	0.448±0.56	0.359±0.56	0.412±1.1	0.3616	0.8100
F0428	0.028±0.02	0.018±0.02	0.031±0.03	0.2762	0.7100	0.098±0.07	0.13±0.08	0.118±0.07	0.2087	0.7600
F082	2.144±1.11	2.006±1.47	1.767±0.75	0.6685	0.9000	0.309±0.32	0.177±0.12	0.303±0.68	0.0929	0.5800
FD2005	0.144±0.11	0.22±0.19	0.263±0.25	0.1494	0.6300	0.14±0.13	0.255±0.15	0.189±0.09	0.0107	0.3500
Fermentimonas	0.004±0.01	0.004±0.01	0.002±0	0.5518	0.8800	0.056±0.08	0.029±0.05	0.005±0.01	0.0406	0.4700
Fibrobacter	3.211±1.02	3.524±1.91	3.924±1.57	0.2305	0.7100	2.922±0.73	4.383±1.92	4.455±1.7	0.0022	0.2800
Fibrobacter_A	0.097±0.09	0.052±0.04	0.094±0.06	0.0384	0.3200	0.01±0.01	0.007±0.01	0.025±0.03	0.0298	0.4000
Firm-04	0.009±0.01	0.007±0.01	0.008±0.01	0.9012	0.9700	0.086±0.05	0.1±0.08	0.088±0.06	0.9447	0.9900
Firm-16	0.003±0.01	0.004±0.01	0.005±0.01	0.4131	0.7600	0.015±0.02	0.013±0.02	0.033±0.06	0.7846	0.9500
Firmicutes_A_X_X_X_X	0.005±0.01	0.002±0.01	0.004±0.01	0.1518	0.6300	0.013±0.01	0.017±0.02	0.012±0.01	0.6453	0.9100
Firmicutes_B_X_X_X_X	0.006±0.01	0.001±0	0.001±0	0.0068	0.1100	0.039±0.04	0.026±0.03	0.035±0.09	0.3769	0.8100
Flavobacteriaceae_X	0.341±0.26	0.327±0.21	0.212±0.33	0.0023	0.0530	0.15±0.12	0.203±0.16	0.131±0.14	0.1229	0.6200
Flavobacteriales_X_X	0.039±0.02	0.046±0.04	0.046±0.07	0.5603	0.8800	0.06±0.04	0.076±0.08	0.072±0.06	0.7413	0.9500
Flavobacterium	0.104±0.2	0.185±0.34	0.058±0.08	0.9523	1.0000	0.012±0.02	0.034±0.07	0.022±0.05	0.4868	0.8800
Flavobacterium_A	0.069±0.14	0.107±0.2	0.033±0.04	0.6991	0.9200	0.008±0.01	0.014±0.03	0.009±0.01	0.9704	1.0000
Flexilinea	0.044±0.03	0.055±0.04	0.062±0.07	0.8319	0.9500	0.078±0.07	0.096±0.08	0.119±0.08	0.1685	0.7200
Fodinicurvata	0.003±0.01	0.003±0.01	0.002±0.01	0.7582	0.9500	0.001±0	0±0	0.001±0	0.3675	0.8100
Frateuria	0.001±0	0±0	0.005±0.01	0.0183	0.1900	0±0	0±0	0±0	NA	NA
Fusicatenibacter	0.027±0.03	0.04±0.04	0.043±0.05	0.5402	0.8800	0.019±0.03	0.021±0.03	0.021±0.02	0.8238	0.9500
Fusobacterium	0±0	0.001±0	0±0	0.9989	1.0000	0.009±0.02	0.006±0.03	0.011±0.04	0.3543	0.8100
Fusobacterium_A	0.081±0.07	0.083±0.11	0.06±0.1	0.3246	0.7100	0.581±0.63	0.43±0.53	0.367±0.51	0.5844	0.9000
Fusobacterium_C	0.146±0.12	0.139±0.17	0.082±0.09	0.2032	0.7100	0.644±0.77	0.67±0.79	0.8±1.26	0.8885	0.9700
Gammaproteobacteria_X_X_X	0.38±0.22	0.385±0.39	0.326±0.41	0.2886	0.7100	0.161±0.1	0.179±0.13	0.124±0.12	0.1954	0.7500
Gastranaerophilaceae_X	0.018±0.03	0.026±0.05	0.01±0.02	0.6573	0.9000	0.002±0.01	0.006±0.01	0.005±0.01	0.3870	0.8200
GCA-900066495	0.001±0	0.002±0	0±0	0.1495	0.6300	0.006±0.01	0.006±0.01	0.003±0.01	0.6534	0.9100
GCA-900066905	0.005±0.01	0.004±0.01	0.004±0.01	0.9794	1.0000	0.009±0.01	0.016±0.02	0.015±0.03	0.7445	0.9500
GCA-900066995	0.025±0.03	0.08±0.26	0.046±0.08	0.4839	0.8500	0.07±0.1	0.098±0.1	0.167±0.3	0.3735	0.8100
Gemella_A	0.002±0.01	0.002±0	0.002±0	0.9720	1.0000	0.005±0.01	0.006±0.01	0.003±0	0.8552	0.9600
Gemmobacter_A	0.007±0.02	0.013±0.05	0.022±0.06	0.2269	0.7100	0.004±0.02	0.004±0.02	0.01±0.03	0.3514	0.8100
Geothermobacter	0.002±0	0.001±0	0.002±0	0.3565	0.7100	0.016±0.02	0.008±0.01	0.013±0.02	0.6399	0.9100
Gluconobacter	0.004±0.01	0±0	0.007±0.01	0.0650	0.4600	0.001±0	0±0	0.008±0.01	0.0058	0.3100

Glutamicibacter	0.002±0.01	0±0	0.003±0.01	0.2282	0.7100	0.003±0.02	0±0	0.011±0.02	0.0087	0.3300
GN02-873	0.011±0.02	0.013±0.02	0.011±0.03	0.1282	0.6000	0.012±0.02	0.018±0.04	0.01±0.02	0.4638	0.8800
GWE2-31-10	0.006±0.01	0.018±0.02	0.009±0.02	0.0175	0.1900	0.005±0.01	0.008±0.01	0.006±0.01	0.6930	0.9500
GWF2-44-16	0.004±0.01	0.006±0.02	0.004±0.01	0.8555	0.9600	0±0	0±0	0±0	NA	NA
Helcococcaceae_X	0±0	0±0	0±0	0.3679	0.7100	0.01±0.02	0.01±0.02	0.006±0.01	0.7732	0.9500
Helcococcus	0.005±0.01	0.006±0.01	0.003±0.01	0.4545	0.8000	0.018±0.02	0.013±0.02	0.012±0.02	0.7867	0.9500
Hepatobacter	0.028±0.07	0.053±0.1	0.019±0.04	0.8992	0.9700	0.001±0	0.004±0.01	0.004±0.01	0.3570	0.8100
Humitalea	0±0	0.001±0	0.001±0.01	0.3489	0.7100	0.024±0.11	0.003±0.01	0±0	0.3675	0.8100
Hungatella_A	0.435±0.37	0.457±0.42	0.571±0.46	0.5370	0.8800	0.319±0.25	0.439±0.31	0.534±0.43	0.2260	0.7600
Inquilinus	0±0	0±0	0.016±0.07	0.6012	0.8800	0.235±1.02	0.065±0.17	0±0	0.2235	0.7600
Kapabacteriaceae_X	0.003±0.01	0.001±0	0.001±0.01	0.6542	0.9000	0.009±0.01	0.006±0.01	0.007±0.01	0.5558	0.9000
Kingella	0.098±0.11	0.073±0.07	0.033±0.07	0.0021	0.0520	1.039±1.41	0.461±0.5	0.252±0.35	0.0532	0.5200
Kiritimatiellae_X_X_X	0.525±0.24	0.439±0.17	0.464±0.21	0.5112	0.8800	0.135±0.12	0.141±0.12	0.191±0.18	0.3633	0.8100
KLE1796	0±0	0±0	0±0	0.1308	0.6000	0.002±0	0.009±0.01	0.002±0	0.0453	0.4800
Kocuria	0±0	0±0	0.015±0.07	0.3679	0.7100	0.173±0.74	0.054±0.14	0±0	0.2282	0.7600
Lachnoanaerobaculum	0.007±0.01	0.013±0.03	0.016±0.02	0.2632	0.7100	0.054±0.06	0.108±0.08	0.056±0.05	0.0287	0.4000
Lachnospira	0.01±0.02	0.017±0.04	0.014±0.03	0.9469	1.0000	0.013±0.02	0.007±0.01	0.012±0.03	0.8752	0.9600
Lachnospiraceae_X	2.964±1.64	3.341±2.5	3.515±1.93	0.6505	0.9000	3.637±1.67	4.807±1.81	4.343±1.76	0.0920	0.5800
Lachnospirales_X_X	0.061±0.04	0.066±0.05	0.052±0.05	0.3663	0.7100	0.092±0.06	0.104±0.05	0.095±0.06	0.6516	0.9100
Lactobacillales_X_X	0.002±0	0.002±0.01	0±0	0.1257	0.6000	0.021±0.04	0.019±0.03	0.007±0.02	0.0608	0.5500
Lactococcus	0.004±0.01	0.001±0	0.016±0.02	0.0001	0.0093	0.013±0.03	0.009±0.03	0.041±0.06	0.0120	0.3600
Lactonifactor	0±0	0.005±0.01	0.007±0.02	0.2186	0.7100	0.008±0.02	0.009±0.02	0.013±0.02	0.7260	0.9500
Lancefieldella	0±0	0.001±0	0±0	0.3679	0.7100	0.004±0.01	0.012±0.02	0.004±0.01	0.0798	0.5600
Leaf454	0.103±0.13	0.088±0.09	0.111±0.2	0.9604	1.0000	0.16±0.24	0.079±0.09	0.146±0.27	0.4429	0.8500
Lenti-01	0.123±0.09	0.153±0.15	0.075±0.07	0.0478	0.3500	0.092±0.09	0.101±0.08	0.093±0.09	0.7724	0.9500
Lentimicrobium	0.708±0.31	0.59±0.31	0.689±0.24	0.3452	0.7100	0.451±0.23	0.422±0.16	0.445±0.15	0.8652	0.9600
Leptotrichiaceae_X	0.001±0	0.001±0	0.001±0	0.8242	0.9500	0.003±0.01	0.008±0.02	0.005±0.01	0.3861	0.8200
Leuconostoc	0.008±0.02	0.002±0	0.022±0.03	0.0021	0.0520	0.029±0.07	0.015±0.06	0.077±0.11	0.0191	0.3900
Lysobacter	0±0	0.003±0.02	0.003±0.01	0.6012	0.8800	0.033±0.14	0.012±0.03	0±0	0.2235	0.7600
Mailhella	0.019±0.02	0.013±0.02	0.011±0.01	0.2479	0.7100	0.036±0.03	0.054±0.05	0.044±0.02	0.2433	0.7600
Mannheimia	0.038±0.05	0.019±0.03	0.008±0.01	0.0431	0.3400	0.457±0.6	0.211±0.26	0.08±0.1	0.0699	0.5500

Marinilabiliaceae_X	0.001±0	0.002±0.01	0.002±0.01	0.8659	0.9700	0.004±0.01	0.004±0.01	0.006±0.02	0.9133	0.9900
Massilia_B	0.108±0.27	0.07±0.22	0.022±0.06	0.6734	0.9000	0.001±0.01	0.013±0.04	0.004±0.01	0.6074	0.9000
Massilibacteroides	0.125±0.08	0.134±0.11	0.082±0.07	0.1079	0.5900	0.196±0.21	0.175±0.19	0.123±0.11	0.7626	0.9500
Metamycoplasma	0.002±0.01	0.004±0.01	0±0	0.3194	0.7100	0.003±0.01	0.007±0.01	0.003±0.01	0.0791	0.5600
Methylobacterium	0.001±0	0±0	0.002±0.01	0.3675	0.7100	0.009±0.04	0.001±0	0.002±0	0.3408	0.8100
Micrococcaceae_X	0±0	0.002±0.01	0±0	0.3430	0.7100	0±0	0.01±0.04	0±0	0.3430	0.8100
Monoglobales_X_X	0.002±0.01	0.005±0.02	0.007±0.02	0.8738	0.9700	0±0	0±0	0±0	NA	NA
Monoglobus	0.069±0.06	0.06±0.04	0.081±0.08	0.9219	0.9800	0.051±0.03	0.05±0.04	0.055±0.03	0.3995	0.8400
Moraxella	0.848±1.33	0.518±0.65	0.303±0.45	0.0311	0.2800	1.286±1.13	1.496±2.32	0.768±1.01	0.1591	0.7000
Moraxella_A	0.001±0.01	0.002±0.01	0.001±0	0.8242	0.9500	0.004±0.01	0.005±0.01	0.004±0.01	0.5890	0.9000
Moraxella_C	0±0	0±0	0±0	NA	NA	0.045±0.14	0.074±0.18	0.02±0.07	0.6300	0.9100
Moraxellaceae_X	0.082±0.07	0.147±0.17	0.1±0.11	0.5799	0.8800	0.063±0.08	0.046±0.07	0.056±0.06	0.5514	0.9000
Muribaculaceae_X	0.191±0.18	0.184±0.14	0.228±0.27	0.9679	1.0000	1.9±1.46	2.043±1.15	1.72±1.32	0.5017	0.8900
Ndongobacter	0.001±0	0±0	0±0	0.3430	0.7100	0.004±0.01	0.006±0.01	0.004±0.01	0.9367	0.9900
Negativicutes_X_X_X	0.108±0.05	0.114±0.08	0.099±0.06	0.8172	0.9500	0.039±0.03	0.038±0.03	0.04±0.05	0.7631	0.9500
Neisseria_B	0.002±0	0.003±0.01	0.006±0.02	0.8090	0.9500	0.018±0.03	0.01±0.02	0.002±0	0.0657	0.5500
Neisseriaceae_X	0.045±0.05	0.04±0.03	0.023±0.02	0.1429	0.6300	0.127±0.1	0.132±0.07	0.075±0.08	0.0232	0.4000
Neorhizobium	0.007±0.02	0.002±0.01	0.045±0.09	0.0008	0.0250	0.2±0.83	0.055±0.14	0.014±0.02	0.6243	0.9100
Niveispirillum	0.005±0.01	0.008±0.03	0.007±0.02	0.5449	0.8800	0±0	0±0	0.001±0	0.7476	0.9500
NS-102	0.001±0.01	0.002±0	0.009±0.03	0.2483	0.7100	0.005±0.01	0.008±0.01	0.002±0.01	0.1919	0.7500
OEMR01	0.002±0	0.002±0	0±0	0.1050	0.5900	0.042±0.1	0.04±0.06	0.02±0.03	0.2981	0.8100
OLB17	0±0	0±0	0±0	NA	NA	0.004±0.01	0.007±0.02	0.001±0	0.8487	0.9600
Olegusella	0.001±0	0±0	0±0	0.3679	0.7100	0.006±0.02	0.01±0.02	0.006±0.02	0.7158	0.9500
Olsenella	0.011±0.01	0.019±0.02	0.004±0.01	0.0051	0.0950	0.041±0.07	0.087±0.09	0.047±0.05	0.0978	0.6000
Opitutaceae_X	1.788±1	1.101±0.95	0.994±0.49	0.0097	0.1500	1.108±0.51	0.532±0.44	0.842±1.03	0.0011	0.2100
Opitutus	0.005±0.02	0.014±0.03	0±0	0.0785	0.5200	0±0	0±0	0±0	NA	NA
Oribacterium	0.015±0.02	0.021±0.03	0.027±0.02	0.2480	0.7100	0.002±0.01	0.006±0.01	0.005±0.01	0.5525	0.9000
Orrella	0.016±0.03	0.011±0.02	0.009±0.02	0.8445	0.9500	0.001±0	0±0	0.005±0.01	0.2083	0.7600
Oscillibacter	0.017±0.02	0.013±0.02	0.015±0.02	0.9770	1.0000	0.023±0.02	0.017±0.02	0.047±0.06	0.2850	0.8100
Oscillospiraceae_X	0.441±0.34	0.461±0.27	0.807±0.86	0.3787	0.7200	0.743±0.51	0.8±0.36	0.829±0.58	0.7529	0.9500
Oscillospirales_X_X	0.291±0.16	0.251±0.13	0.23±0.15	0.4138	0.7600	0.187±0.09	0.234±0.13	0.212±0.12	0.5691	0.9000

Paenibacillus_J	0.003±0.01	0±0	0.003±0.01	0.1257	0.6000	0.005±0.02	0±0	0.004±0.01	0.1216	0.6200
PALSA-1355	0.013±0.02	0.003±0.01	0.007±0.01	0.1344	0.6000	0.008±0.01	0.005±0.01	0.016±0.03	0.4289	0.8500
Paludibacteraceae_X	0.311±0.32	0.276±0.18	0.192±0.14	0.3274	0.7100	0.07±0.06	0.063±0.06	0.091±0.17	0.8210	0.9500
Paramesorhizobium	0.003±0.01	0±0	0.01±0.01	0.0056	0.0980	0.043±0.17	0.015±0.04	0.003±0.01	0.7968	0.9500
Paramuribaculum	0±0	0.001±0	0.009±0.02	0.0052	0.0950	0.007±0.01	0.012±0.02	0.018±0.02	0.0998	0.6000
Parapedobacter	0.003±0.01	0.006±0.01	0.001±0	0.7786	0.9500	0.004±0.02	0±0	0±0	0.1308	0.6200
Paraprevotella	0.24±0.28	0.191±0.12	0.196±0.13	0.8395	0.9500	0.294±0.24	0.237±0.22	0.28±0.19	0.6412	0.9100
Parvibaculum	0.077±0.09	0.157±0.3	0.055±0.06	0.8053	0.9500	0.055±0.08	0.085±0.14	0.06±0.07	0.7815	0.9500
Parvimonas	0.005±0.01	0.006±0.01	0.006±0.02	0.8191	0.9500	0.039±0.05	0.046±0.05	0.025±0.04	0.4493	0.8600
Pasteurellaceae_X	0.687±0.62	0.545±0.63	0.564±0.89	0.5596	0.8800	2.872±3.22	2.243±1.95	2.391±3.9	0.7678	0.9500
Pauljensenia	0±0	0±0	0±0	NA	NA	0.016±0.04	0.015±0.03	0.005±0.01	0.8063	0.9500
Pedosphaeraceae_X	0.239±0.19	0.166±0.15	0.154±0.18	0.1439	0.6300	0.158±0.15	0.139±0.12	0.097±0.07	0.3132	0.8100
PeH17	0.11±0.07	0.107±0.06	0.118±0.13	0.6138	0.8800	0.371±0.18	0.374±0.23	0.408±0.15	0.2909	0.8100
Peptostreptococcaceae_X	0.002±0	0.002±0	0.002±0.01	0.9983	1.0000	0.055±0.07	0.036±0.04	0.036±0.06	0.5941	0.9000
Peptostreptococcales_X_X	0.045±0.04	0.035±0.04	0.037±0.09	0.0833	0.5300	0.201±0.2	0.157±0.15	0.152±0.17	0.8171	0.9500
Pigmentiphaga	0.058±0.22	0.05±0.11	0.035±0.09	0.1092	0.5900	0±0	0±0	0.005±0.02	0.6012	0.9000
Pirellulales_X_X	0.107±0.13	0.079±0.08	0.212±0.2	0.1134	0.6000	0.133±0.12	0.121±0.16	0.304±0.26	0.0063	0.3100
Planctomycetes_X_X_X	0.012±0.02	0.01±0.01	0.006±0.02	0.2276	0.7100	0.011±0.02	0.007±0.02	0.016±0.02	0.4424	0.8500
Planctomycetota_X_X_X_X	0.031±0.05	0.02±0.05	0.018±0.03	0.2033	0.7100	0.033±0.04	0.038±0.03	0.026±0.04	0.2202	0.7600
Porphyromonas	0.488±0.43	0.354±0.39	0.183±0.21	0.0136	0.1800	1.401±1.23	1.158±1.27	0.849±0.86	0.5870	0.9000
Prevotella	25.936±6.78	27.843±13.35	28.168±10.21	0.8124	0.9500	17.529±5.51	16.553±4.52	17.845±4.41	0.6693	0.9300
Prevotellamassilia	0.177±0.07	0.175±0.09	0.24±0.1	0.1269	0.6000	0.25±0.13	0.315±0.16	0.225±0.1	0.2276	0.7600
Prolixibacteraceae_X	0.603±0.55	0.42±0.26	0.497±0.31	0.5134	0.8800	1.655±1.14	1.413±0.51	1.698±1.17	0.9820	1.0000
Prosthecobacter	0.007±0.01	0.016±0.05	0.03±0.1	0.6419	0.8900	0.006±0.01	0.015±0.03	0.02±0.06	0.9418	0.9900
Proteiniclasticum	0.011±0.02	0.025±0.05	0.027±0.06	0.8709	0.9700	0.029±0.05	0.049±0.09	0.035±0.06	0.8244	0.9500
Proteobacteria_X_X_X_X	0.181±0.12	0.203±0.18	0.321±0.32	0.1701	0.6800	0.123±0.25	0.088±0.05	0.146±0.11	0.0718	0.5500
Pseudaminobacter	0.002±0.01	0.004±0.01	0.006±0.02	0.5472	0.8800	0.017±0.03	0.01±0.02	0.017±0.03	0.8338	0.9600
Pseudomonadaceae_X	0.119±0.38	0.114±0.32	0.044±0.05	0.7097	0.9300	0.113±0.44	0.048±0.08	0.004±0.01	0.0657	0.5500
Pseudomonadales_X_X	0.006±0.01	0.011±0.02	0.003±0.01	0.4197	0.7700	0.003±0.01	0.004±0.01	0.003±0.01	0.8723	0.9600
Pseudomonas_A	0.228±0.4	0.234±0.34	0.147±0.17	0.9837	1.0000	0.073±0.09	0.091±0.18	0.081±0.07	0.5640	0.9000
Pseudomonas_B	0.006±0.01	0.003±0.01	0.005±0.01	0.6960	0.9200	0.003±0.01	0.001±0	0.003±0.01	0.4726	0.8800

Pseudomonas_D	0.073±0.19	0.061±0.15	0.021±0.05	0.3181	0.7100	0.001±0	0.001±0	0.001±0.01	0.7880	0.9500
Pseudomonas_E	0.037±0.08	0.012±0.04	0.069±0.07	0.0017	0.0490	0±0	0±0	0.002±0.01	0.1308	0.6200
Pseudomonas_M	0.134±0.26	0.091±0.23	0.185±0.21	0.0332	0.2800	0.031±0.12	0.016±0.03	0.014±0.03	0.7204	0.9500
Pygmaiobacter	0.007±0.01	0.01±0.01	0.004±0.01	0.2287	0.7100	0.064±0.11	0.047±0.11	0.033±0.05	0.5000	0.8900
Pyramidobacter	0.054±0.05	0.072±0.08	0.075±0.09	0.7393	0.9500	0.234±0.1	0.406±0.18	0.352±0.27	0.0057	0.3100
RC9	4.733±1.98	4.856±2.87	4.115±2.21	0.4493	0.8000	2.703±0.91	3.043±0.78	3.191±1.01	0.3010	0.8100
RF16	4.333±1.79	5.045±2.09	3.884±1.27	0.1490	0.6300	1.311±1.08	1.09±0.74	1.116±0.91	0.9641	1.0000
Rhizobiaceae_X	0.001±0.01	0.001±0	0.025±0.06	0.0026	0.0530	0.128±0.55	0.04±0.1	0.009±0.02	0.8349	0.9600
Rhizobiales_X_X	0.009±0.01	0.012±0.02	0.01±0.02	0.7493	0.9500	0.002±0.01	0.005±0.01	0.002±0.01	0.1292	0.6200
Rhodospirillaceae_X	0.006±0.01	0.008±0.01	0.021±0.03	0.1621	0.6600	0±0	0.001±0	0.001±0	0.8068	0.9500
Riemerella	0.002±0	0.002±0.01	0.004±0.01	0.8994	0.9700	0.005±0.01	0.005±0.01	0.006±0.01	0.9363	0.9900
Roseomonas	0.006±0.01	0.001±0	0.018±0.02	0.0003	0.0140	0±0	0±0	0±0	0.3679	0.8100
Rothia	0.004±0.01	0.004±0.01	0.003±0.01	0.9987	1.0000	0.002±0.01	0.007±0.02	0±0	0.1545	0.7000
RUG131	0.207±0.13	0.284±0.19	0.185±0.12	0.1087	0.5900	0.074±0.03	0.108±0.07	0.087±0.05	0.2366	0.7600
RUG163	0.418±0.38	0.257±0.18	0.682±0.53	0.0148	0.1800	0.465±0.36	0.448±0.3	0.537±0.29	0.4695	0.8800
RUG350	0.156±0.08	0.168±0.1	0.141±0.09	0.4705	0.8300	0.139±0.34	0.095±0.1	0.08±0.1	0.8578	0.9600
Ruminiclostridium	0.164±0.13	0.198±0.17	0.215±0.21	0.8132	0.9500	0.124±0.09	0.146±0.12	0.159±0.13	0.8082	0.9500
Ruminiclostridium_C	0.235±0.15	0.314±0.23	0.544±1.06	0.3050	0.7100	0.574±0.35	0.8±0.3	0.85±0.68	0.0365	0.4600
Ruminiclostridium_D	0.022±0.02	0.015±0.01	0.015±0.01	0.3489	0.7100	0.007±0.01	0.002±0	0.006±0.01	0.2355	0.7600
Ruminiclostridium_F	0.016±0.03	0.015±0.03	0.005±0.01	0.3740	0.7100	0.005±0.02	0.001±0.01	0.009±0.03	0.7897	0.9500
Ruminococcaceae_X	0.097±0.08	0.12±0.16	0.088±0.12	0.2222	0.7100	0.314±0.23	0.326±0.23	0.299±0.18	0.9722	1.0000
Ruminococcus	0.062±0.06	0.052±0.07	0.118±0.09	0.0160	0.1800	0.145±0.15	0.206±0.3	0.224±0.21	0.4105	0.8400
Ruminococcus_C	0.053±0.05	0.046±0.05	0.043±0.05	0.7633	0.9500	0.082±0.1	0.083±0.07	0.068±0.05	0.6461	0.9100
Ruminococcus_D	0.301±0.25	0.286±0.18	0.491±0.39	0.2117	0.7100	0.389±0.31	0.554±0.41	0.504±0.32	0.3655	0.8100
Ruminococcus_F	0.089±0.06	0.059±0.06	0.077±0.08	0.3032	0.7100	0.201±0.17	0.21±0.15	0.246±0.24	0.9392	0.9900
Saccharibacillus	0.001±0.01	0±0	0.007±0.01	0.0027	0.0530	0±0	0.001±0	0.002±0.01	0.2083	0.7600
Saccharicrinis	0.004±0.01	0.016±0.03	0.007±0.01	0.3689	0.7100	0.006±0.01	0.014±0.03	0.011±0.03	0.9754	1.0000
Saccharimonadaceae_X	0.141±0.14	0.23±0.11	0.135±0.12	0.0128	0.1800	0.08±0.07	0.096±0.07	0.091±0.07	0.5425	0.9000
Saccharimonadales_X_X	0.052±0.07	0.057±0.06	0.048±0.06	0.8217	0.9500	0.004±0.01	0.003±0.01	0.014±0.02	0.0299	0.4000
Saccharofermentans	0.127±0.07	0.145±0.11	0.2±0.15	0.2756	0.7100	0.287±0.16	0.268±0.08	0.392±0.31	0.4065	0.8400
Schwartzia	0.401±0.25	0.336±0.18	0.34±0.14	0.8037	0.9500	0.133±0.11	0.164±0.11	0.185±0.11	0.1032	0.6100

Selenomonadaceae_X	0.117±0.09	0.197±0.16	0.072±0.07	0.0138	0.1800	0.024±0.03	0.049±0.05	0.022±0.03	0.1606	0.7000
Selenomonas_B	0.02±0.03	0.021±0.03	0.006±0.01	0.1055	0.5900	0.01±0.03	0.007±0.01	0.001±0	0.0836	0.5600
Serratia	0.007±0.02	0±0	0.017±0.02	0.0003	0.0140	0±0	0±0	0.006±0.01	0.0184	0.3900
Shinella	0.002±0.01	0.004±0.01	0.029±0.13	0.8389	0.9500	0.011±0.02	0.009±0.02	0.013±0.03	0.8723	0.9600
Simonsiella	0.034±0.07	0.029±0.05	0.013±0.02	0.9161	0.9800	0.08±0.08	0.12±0.18	0.033±0.05	0.0703	0.5500
Sneathia	0.023±0.08	0.026±0.07	0.003±0.01	0.3271	0.7100	0.002±0.01	0.011±0.03	0.004±0.01	0.8424	0.9600
Soleaferrea	0.066±0.11	0.043±0.04	0.068±0.06	0.3493	0.7100	0.114±0.13	0.133±0.13	0.074±0.11	0.2670	0.8100
Sphaerochaeta	0.03±0.04	0.032±0.04	0.028±0.03	0.9843	1.0000	0.004±0.01	0.003±0.01	0.004±0.01	0.9015	0.9800
Sphaerochaeta_A	0.022±0.02	0.025±0.02	0.014±0.01	0.2342	0.7100	0.019±0.02	0.009±0.01	0.013±0.02	0.2542	0.7900
Sphaerochaetaceae_X	0.162±0.13	0.104±0.07	0.198±0.18	0.2020	0.7100	0.113±0.05	0.095±0.06	0.175±0.13	0.0424	0.4700
Sphingobacterium	0.088±0.15	0.061±0.12	0.153±0.14	0.0399	0.3300	0.005±0.01	0.003±0.01	0.026±0.03	0.0037	0.2900
Sphingomonas	0.018±0.04	0.01±0.01	0.072±0.06	0.0002	0.0110	0.009±0.02	0.007±0.01	0.013±0.02	0.5216	0.9000
Sphingopyxis_A	0.003±0.01	0.004±0.01	0.011±0.03	0.6065	0.8800	0.03±0.12	0.016±0.04	0.01±0.03	0.5742	0.9000
Spirochaetia_X_X_X	1.035±0.53	0.873±0.27	1.618±0.98	0.0200	0.2000	0.762±0.37	1.054±0.47	1.393±0.83	0.0193	0.3900
Spirochaetota_X_X_X_X	0.017±0.02	0.026±0.02	0.013±0.02	0.1869	0.7100	0.022±0.02	0.052±0.04	0.039±0.03	0.0210	0.3900
Stenotrophomonas	0.965±1.8	0.773±1.32	0.764±0.77	0.6374	0.8900	0.061±0.07	0.069±0.1	0.15±0.19	0.0364	0.4600
Streptococcus	0.191±0.37	0.055±0.06	0.03±0.06	0.0439	0.3400	0.754±1.05	0.505±0.5	0.454±0.81	0.4379	0.8500
Succiniclasticum	1.276±0.95	1.118±0.7	1.297±0.91	0.8988	0.9700	3.361±1.69	4.755±2.94	4.761±1.79	0.0145	0.3800
Succinimonas	0.19±0.08	0.286±0.22	0.212±0.2	0.2266	0.7100	0.117±0.13	0.131±0.13	0.101±0.16	0.3094	0.8100
Succinivibrio	0.198±0.13	0.314±0.31	0.313±0.25	0.2129	0.7100	0.03±0.03	0.057±0.05	0.051±0.04	0.1104	0.6200
Syner-01	0.002±0.01	0±0	0.001±0	0.3675	0.7100	0±0	0.001±0	0.024±0.1	0.1092	0.6200
Synergistales_X_X	0.004±0.01	0.004±0.01	0.015±0.02	0.0329	0.2800	0.011±0.01	0.007±0.01	0.011±0.01	0.6249	0.9100
Synergistes	0±0	0±0	0±0	0.3679	0.7100	0.006±0.01	0.005±0.01	0.004±0.01	0.5248	0.9000
SZUA-359	0.341±0.24	0.528±0.4	0.365±0.4	0.2385	0.7100	0.218±0.13	0.332±0.44	0.169±0.16	0.1979	0.7500
Tannerella	0.001±0	0.001±0.01	0±0	0.4424	0.8000	0.005±0.01	0.008±0.01	0.003±0.01	0.6884	0.9500
Tannerellaceae_X	0.027±0.02	0.018±0.02	0.018±0.03	0.0803	0.5200	0.043±0.05	0.044±0.04	0.027±0.03	0.6025	0.9000
TF01-11	0.019±0.03	0.013±0.02	0.004±0.01	0.1249	0.6000	0.082±0.11	0.04±0.05	0.034±0.04	0.0856	0.5600
Tissierellales_X_X	0.042±0.03	0.062±0.05	0.062±0.06	0.5518	0.8800	0.156±0.08	0.168±0.07	0.174±0.12	0.5739	0.9000
Treponema_A	0±0	0.003±0.01	0±0	0.0699	0.4800	0.01±0.04	0.004±0.01	0.013±0.05	0.6332	0.9100
Treponema_B	0.007±0.01	0.006±0.01	0.003±0.01	0.5856	0.8800	0.034±0.05	0.03±0.04	0.033±0.09	0.4047	0.8400
Treponema_C	0.011±0.02	0.009±0.01	0.011±0.02	0.9758	1.0000	0.014±0.01	0.006±0.01	0.014±0.02	0.1768	0.7400

Treponema_D	0.091±0.05	0.106±0.04	0.084±0.07	0.1030	0.5900	0.048±0.03	0.09±0.06	0.074±0.06	0.0405	0.4700
Treponemataceae_X	0.193±0.13	0.146±0.06	0.248±0.16	0.0907	0.5600	0.153±0.09	0.211±0.1	0.284±0.2	0.0607	0.5500
Treponematales_X_X	0.063±0.04	0.06±0.03	0.092±0.11	0.7071	0.9300	0.102±0.08	0.105±0.08	0.174±0.14	0.0700	0.5500
UBA1020	0.008±0.03	0.006±0.01	0.001±0	0.1450	0.6300	0.001±0.01	0±0	0.001±0	0.6012	0.9000
UBA1033	0.058±0.21	0.036±0.06	0.017±0.03	0.3306	0.7100	0.064±0.15	0.046±0.09	0.05±0.09	0.3627	0.8100
UBA1067	2.339±1.14	1.861±0.61	1.833±0.57	0.4102	0.7600	0.515±0.3	0.442±0.17	0.66±0.7	0.3405	0.8100
UBA1174	0.096±0.14	0.089±0.09	0.045±0.05	0.1055	0.5900	0.037±0.09	0.014±0.02	0.058±0.2	0.7230	0.9500
UBA1191	0.003±0.01	0.005±0.01	0.005±0.02	0.6212	0.8900	0.023±0.02	0.02±0.03	0.027±0.03	0.5315	0.9000
UBA1258	0.165±0.12	0.233±0.21	0.17±0.15	0.6392	0.8900	0.229±0.13	0.305±0.16	0.264±0.16	0.4864	0.8800
UBA1361	0.081±0.06	0.113±0.07	0.076±0.07	0.2246	0.7100	0.078±0.03	0.081±0.05	0.075±0.05	0.8599	0.9600
UBA1394	0.004±0.01	0.002±0	0.006±0.01	0.3491	0.7100	0.024±0.03	0.02±0.02	0.034±0.04	0.9207	0.9900
UBA1436	0.347±0.22	0.363±0.26	0.2±0.13	0.0274	0.2600	0.121±0.11	0.135±0.09	0.176±0.15	0.4272	0.8500
UBA1532	0.018±0.02	0.022±0.02	0.019±0.03	0.2258	0.7100	0.018±0.02	0.034±0.03	0.023±0.02	0.0857	0.5600
UBA1547	0.003±0.01	0.004±0.01	0.004±0.01	0.6332	0.8900	0±0	0±0	0.001±0	0.3679	0.8100
UBA1711	0.304±0.14	0.347±0.21	0.393±0.3	0.7327	0.9500	0.204±0.16	0.202±0.15	0.217±0.13	0.7869	0.9500
UBA1829	0.45±0.26	0.315±0.2	0.537±0.67	0.2401	0.7100	0.111±0.07	0.107±0.05	0.198±0.28	0.5232	0.9000
UBA2450	0.027±0.03	0.125±0.24	0.052±0.05	0.0005	0.0180	0.043±0.07	0.022±0.02	0.023±0.02	0.9285	0.9900
UBA3206	0.164±0.14	0.084±0.05	0.151±0.14	0.2058	0.7100	0.03±0.02	0.016±0.02	0.031±0.03	0.0804	0.5600
UBA4179	0.047±0.07	0.048±0.08	0.072±0.16	0.9498	1.0000	0.048±0.06	0.023±0.04	0.043±0.08	0.4436	0.8500
UBA4658	0.001±0	0±0	0.001±0.01	0.9989	1.0000	0.003±0.01	0.004±0.02	0.005±0.02	0.8080	0.9500
UBA5124	1.562±0.76	1.462±0.51	1.519±1.01	0.7966	0.9500	1.368±0.58	1.208±0.73	1.421±0.77	0.3836	0.8200
UBA5194	0.007±0.02	0.007±0.01	0.001±0	0.1997	0.7100	0.006±0.01	0.014±0.02	0.006±0.01	0.2319	0.7600
UBA5946	0.008±0.02	0.007±0.02	0.006±0.02	0.7830	0.9500	0±0	0±0	0±0	NA	NA
UBA636	0.003±0.01	0.002±0	0.001±0	0.6734	0.9000	0.005±0.01	0.007±0.02	0.012±0.04	0.9230	0.9900
UBA6984	0.022±0.03	0.014±0.02	0.027±0.02	0.2313	0.7100	0±0	0±0	0±0	NA	NA
UBA7182	0.093±0.27	0.026±0.06	0.008±0.03	0.2139	0.7100	0.008±0.02	0.006±0.01	0.011±0.04	0.7733	0.9500
UBA733	0.071±0.07	0.105±0.06	0.066±0.06	0.0294	0.2700	0.006±0.01	0.012±0.02	0.011±0.02	0.8140	0.9500
UBA7862	0.085±0.06	0.086±0.07	0.071±0.05	0.8856	0.9700	0.016±0.02	0.02±0.01	0.02±0.03	0.5064	0.8900
UBA8416	0.053±0.04	0.046±0.03	0.048±0.03	0.7652	0.9500	0.014±0.02	0.013±0.02	0.014±0.01	0.8031	0.9500
UBA8525	0.084±0.1	0.05±0.05	0.048±0.05	0.6569	0.9000	0.002±0.01	0.001±0	0.007±0.02	0.9922	1.0000
UBA8953	0.205±0.09	0.254±0.07	0.174±0.11	0.0163	0.1800	0.083±0.06	0.136±0.09	0.136±0.08	0.0261	0.4000

UBA932_X	0.065±0.06	0.055±0.06	0.093±0.09	0.2901	0.7100	0.116±0.1	0.122±0.13	0.104±0.11	0.8722	0.9600
UBA9983_A_X_X	0.018±0.04	0.068±0.18	0.034±0.09	0.8063	0.9500	0.001±0	0.007±0.02	0.004±0.01	0.6803	0.9400
V5-8f	0±0	0.001±0	0±0	0.6012	0.8800	0.015±0.04	0.018±0.06	0.005±0.01	0.9916	1.0000
Vampirovibrionia_X_X_X	0.002±0.01	0.003±0.01	0±0	0.3555	0.7100	0.002±0.01	0.001±0	0.004±0.02	0.8080	0.9500
Varibaculum_A	0.001±0	0±0	0.001±0	0.3489	0.7100	0.004±0.01	0.008±0.01	0.001±0	0.0385	0.4700
Veillonella	0.039±0.11	0.004±0.01	0.006±0.01	0.3696	0.7100	0.062±0.08	0.114±0.27	0.11±0.23	0.9597	1.0000
Veillonella_A	0.022±0.06	0.009±0.01	0.008±0.02	0.8433	0.9500	0.258±0.52	0.222±0.45	0.164±0.36	0.9240	0.9900
Victivallis	0.032±0.03	0.039±0.03	0.039±0.04	0.3711	0.7100	0.008±0.01	0.009±0.01	0.01±0.03	0.7174	0.9500
Vitiosangium	0.002±0.01	0.001±0	0.003±0	0.4975	0.8700	0.001±0	0.003±0	0.004±0.01	0.2852	0.8100
Weeksellaceae_X	0.001±0	0.001±0	0.001±0	0.9994	1.0000	0.001±0	0.008±0.02	0.002±0	0.0845	0.5600
Weissella	0.001±0	0±0	0.001±0	0.2368	0.7100	0.004±0.01	0±0	0.007±0.02	0.3401	0.8100
Williamwhitmania	0.038±0.04	0.018±0.03	0.027±0.03	0.1334	0.6000	0.262±0.24	0.102±0.13	0.21±0.25	0.0450	0.4800
Xanthomonadaceae_X	0.002±0.01	0.008±0.02	0.01±0.04	0.2325	0.7100	0.004±0.01	0.001±0	0.003±0.01	0.8496	0.9600
Xanthomonadales_X_X	0.001±0	0.002±0.01	0.001±0	0.7552	0.9500	0±0	0.004±0.01	0.005±0.01	0.3401	0.8100
Xanthomonas_A	0.006±0.02	0.001±0	0.012±0.02	0.0177	0.1900	0±0	0±0	0±0	NA	NA
Xanthomonas_B	0.044±0.08	0.051±0.05	0.028±0.05	0.2618	0.7100	0.016±0.03	0.009±0.02	0.013±0.02	0.5124	0.9000
XYC2-FULL-35-21	0.002±0	0.001±0	0.001±0	0.0771	0.5200	0.003±0.01	0.002±0	0.018±0.06	0.9548	0.9900
Zag1	2.528±1.07	2.175±1.3	2.37±1.4	0.2986	0.7100	0.481±0.26	0.573±0.29	0.793±0.88	0.2616	0.8100
Zag111	0.096±0.07	0.083±0.05	0.08±0.06	0.8297	0.9500	0.077±0.05	0.188±0.16	0.124±0.09	0.0492	0.5100

Table S4: Collection-wise (among collection) comparison of taxa at Phylum and Genus level within Liquid fraction and Solid fraction samples. Taxa which are non-significant and had less abundance (Combined rel. abu. < 0.01) are not mentioned in the table. Suffix "_X" in genus name suggests that taxa was assigned at higher taxonomic levels than genus. Multiple suffixes are kept identifying exact level of assignment. % mean relative abundance is mentioned as mean ± standard deviation.

	Liquid fraction							Solid fraction						
**Taxa**	**% mean rel. abu. - Collection1**	**% mean rel. abu. - Collection2**	**% mean rel. abu. - Collection3**	**% mean rel. abu. - Collection4**	**% mean rel. abu. - Collection5**	**Kruskal-Wallis p-value**	**Kruskal-Wallis BH adjusted p-value**	**% mean rel. abu. - Collection1**	**% mean rel. abu. - Collection2**	**% mean rel. abu. - Collection3**	**% mean rel. abu. - Collection4**	**% mean rel. abu. - Collection5**	**Kruskal-Wallis p-value**	**Kruskal-Wallis BH adjusted p-value**
**Phylum**														
Acidobacteriota	0±0	0±0	0±0	0±0	0±0	NA	NA	0.009±0.03	0.005±0.01	0.004±0.01	0.002±0.01	0±0	0.6469	0.6900
Actinobacteriota	0.034±0.09	0.047±0.05	0.039±0.04	0.038±0.04	0.05±0.08	0.1672	0.2100	0.538±0.89	0.228±0.21	0.279±0.16	0.106±0.09	0.197±0.17	0.0342	0.0620
Bacteroidota	73.195±5.84	60.388±8.25	55.565±5.39	57.027±7.52	60.448±12.36	0.0002	0.0009	58.87±6.34	56.233±11.72	48.371±5.18	58.716±5.33	52.692±6.05	0.0037	0.0120
Bdellovibrionota	0±0	0.009±0.01	0.004±0.01	0.006±0.01	0.002±0	0.3569	0.3700	0±0	0.002±0	0.001±0	0.004±0.01	0.001±0	0.2850	0.4300
Campylobacterota	0.026±0.03	0.041±0.05	0.152±0.21	0.067±0.05	0.064±0.05	0.0166	0.0330	0.065±0.06	0.095±0.08	0.079±0.06	0.061±0.06	0.048±0.04	0.4508	0.5200
Chloroflexota	0.024±0.03	0.057±0.06	0.073±0.06	0.058±0.03	0.056±0.05	0.0601	0.0930	0.049±0.04	0.072±0.05	0.153±0.09	0.077±0.03	0.137±0.1	0.0148	0.0330
Cyanobacteria	1.392±0.44	3.718±1.84	2.625±0.89	2.41±0.59	2.28±1.1	0.0001	0.0009	0.461±0.27	0.517±0.27	0.703±0.22	0.803±0.26	1.275±1.13	0.0009	0.0043
Desulfobacterota	0.005±0.01	0.01±0.01	0.013±0.01	0.009±0.01	0.004±0.01	0.1176	0.1600	0.004±0.01	0.005±0.01	0.006±0.01	0.006±0.01	0.001±0	0.3620	0.4400
Desulfobacterota_A	0.015±0.02	0.007±0.01	0.042±0.04	0.031±0.02	0.006±0.01	0.0002	0.0009	0.074±0.08	0.107±0.06	0.117±0.06	0.096±0.05	0.086±0.04	0.3283	0.4300
Desulfuromonadota	0.001±0	0±0	0.004±0.01	0.002±0	0.001±0	0.0315	0.0570	0.013±0.02	0.013±0.02	0.012±0.01	0.019±0.02	0.006±0.01	0.3245	0.4300
Elusimicrobiota	0.393±0.34	0.815±0.35	0.402±0.17	0.442±0.21	0.425±0.14	0.0008	0.0027	0.476±0.23	0.346±0.17	0.218±0.07	0.268±0.1	0.428±0.41	0.0003	0.0020
Fibrobacterota	3.109±1.32	4.277±1.94	3.745±0.74	4.445±1.77	2.592±1.09	0.0119	0.0260	3.697±1.18	3.943±1.82	4.43±2.25	4.217±1.68	3.381±1.28	0.6754	0.7000
Firmicutes	1.505±0.43	1.398±0.66	2.157±1.09	2.327±0.59	2.719±1.44	0.0015	0.0047	1.914±0.73	2.058±1.44	2.368±1.01	1.378±0.46	1.344±0.82	0.0178	0.0370
Firmicutes_A	4.166±1.3	8.815±5.68	11.89±2.44	15.03±4.36	13.999±7.2	0.0000	0.0001	12.186±4.82	11.761±5.36	18.993±4.65	16.006±3.59	21.585±6.01	0.0002	0.0019
Firmicutes_B	0.006±0.01	0.003±0.01	0.001±0	0.001±0	0.003±0.01	0.1210	0.1600	0.095±0.1	0.017±0.02	0.03±0.03	0.018±0.02	0.006±0.01	0.0001	0.0015
Firmicutes_C	2.489±1.14	1.313±1.15	2.374±0.84	2.023±0.74	1.33±0.49	0.0002	0.0009	5.42±2.28	4.536±1.96	4.712±1.49	5.098±1.93	6.513±3.66	0.5417	0.6000
Firmicutes_I	0±0	0.002±0	0.002±0	0.009±0.02	0.012±0.02	0.2522	0.2900	0±0	0.003±0.01	0.008±0.02	0.002±0.01	0.007±0.01	0.3550	0.4400
Firmicutes_K	0.001±0	0±0	0.006±0.01	0.001±0	0.001±0	0.1236	0.1600	0.001±0	0±0	0.001±0.01	0±0	0±0	0.7224	0.7200
Fusobacteriota	0.106±0.09	0.256±0.28	0.319±0.34	0.257±0.26	0.167±0.14	0.3665	0.3700	1.262±1.25	1.679±1.22	1.248±1.08	1.617±2.12	0.187±0.2	0.0017	0.0061
Gemmatimonadota	0±0	0.001±0	0.001±0	0.003±0.01	0±0	0.3228	0.3500	0.001±0	0.005±0.01	0.001±0	0.002±0	0.001±0	0.2225	0.3600
Myxococcota	0.021±0.01	0.028±0.02	0.032±0.03	0.021±0.01	0.006±0.01	0.0044	0.0100	0.04±0.02	0.034±0.03	0.03±0.02	0.027±0.02	0.009±0.01	0.0100	0.0240
Patescibacteria	1.318±0.35	2.467±0.84	1.88±0.68	2.242±0.8	3.199±1.63	0.0002	0.0009	1.255±0.34	1.365±0.46	1.401±0.44	1.965±0.79	2.491±1.08	0.0011	0.0045
Planctomycetota	0.124±0.08	0.08±0.05	0.28±0.22	0.2±0.13	0.191±0.21	0.0409	0.0670	0.133±0.1	0.166±0.22	0.288±0.22	0.284±0.21	0.357±0.21	0.0040	0.0120
Proteobacteria	5.138±3.22	8.752±3.78	10.868±6.3	6.754±3.05	5.456±2.04	0.0019	0.0052	8.862±4.45	12.758±9.73	11.627±6.91	5.062±2.07	3.966±2.28	0.0003	0.0020
Riflebacteria	0.154±0.11	0.222±0.09	0.214±0.08	0.278±0.08	0.188±0.06	0.0325	0.0570	0.086±0.04	0.124±0.09	0.108±0.05	0.186±0.09	0.088±0.05	0.0101	0.0240
Spirochaetota	1.272±0.51	1.931±1.42	2.042±0.97	2.036±0.76	1.964±1.02	0.1851	0.2300	1.942±1.12	1.589±1.04	2.179±0.67	1.46±0.51	1.477±1.04	0.0443	0.0760
Synergistota	0.035±0.05	0.063±0.11	0.142±0.1	0.092±0.05	0.047±0.02	0.0003	0.0013	0.326±0.12	0.442±0.33	0.318±0.11	0.452±0.22	0.228±0.17	0.0267	0.0520
Unclassified Phylum	0.224±0.09	0.29±0.12	0.392±0.13	0.416±0.12	0.299±0.18	0.0023	0.0058	0.268±0.09	0.371±0.14	0.507±0.22	0.452±0.13	0.679±0.25	0.0001	0.0015
Verrucomicrobiota	5.246±1.9	5.01±1.9	4.735±1.53	3.774±1.02	4.493±1.81	0.2932	0.3300	1.954±0.91	1.527±0.63	1.807±0.65	1.616±0.46	2.81±2.79	0.3252	0.4300
**Genus**														
32-67-11	0±0	0±0	0.003±0.01	0.009±0.01	0.003±0.01	0.0241	0.0650	0±0	0±0	0±0	0.002±0.01	0.003±0.01	0.2383	0.3300
4C28d-15_X_X	0.036±0.05	0.076±0.1	0.073±0.06	0.113±0.1	0.031±0.03	0.0115	0.0360	0.027±0.08	0.019±0.02	0.011±0.01	0.019±0.02	0.017±0.02	0.6130	0.6600
992a	0.004±0.01	0.008±0.01	0.007±0.01	0.02±0.02	0.01±0.01	0.1097	0.2000	0.006±0.01	0.004±0.01	0.01±0.01	0.016±0.02	0.008±0.02	0.1635	0.2500
Acetivibrionaceae_X	0.012±0.01	0.02±0.03	0.036±0.03	0.096±0.12	0.157±0.23	0.0077	0.0270	0.095±0.09	0.081±0.06	0.114±0.1	0.103±0.07	0.138±0.14	0.7717	0.7900
Acetoanaerobium	0±0	0±0	0±0	0±0	0.002±0	0.0867	0.1700	0±0	0±0	0±0	0±0	0.006±0.01	0.0021	0.0094
Acetobacteraceae_X	0±0	0.011±0.02	0.005±0.01	0.008±0.01	0.007±0.02	0.3649	0.4800	0±0	0±0	0.007±0.02	0.001±0	0.006±0.01	0.1168	0.2000
Acholeplasma_C	0.43±0.15	0.228±0.23	0.504±0.29	0.646±0.25	0.751±0.32	0.0000	0.0004	0.226±0.09	0.235±0.19	0.339±0.15	0.308±0.13	0.256±0.09	0.1308	0.2200

Acholeplasma_D	0.007±0.01	0.018±0.02	0.026±0.01	0.046±0.02	0.033±0.02	0.0000	0.0005	0±0	0.007±0.01	0.01±0.01	0.009±0.01	0.003±0.01	0.0456	0.0900
Acholeplasmatales_X_X	0.055±0.05	0.012±0.02	0.027±0.02	0.054±0.03	0.192±0.17	0.0000	0.0004	0.025±0.03	0.009±0.01	0.011±0.01	0.041±0.03	0.012±0.02	0.0034	0.0130
Achromobacter	0.003±0.01	0.015±0.04	0.022±0.04	0.029±0.04	0.009±0.03	0.0164	0.0470	0±0	0.001±0	0.006±0.01	0.002±0.01	0±0	0.4243	0.5000
Acidovorax_E	0±0	0.003±0.01	0.005±0.01	0.014±0.03	0.003±0.01	0.0924	0.1800	0±0	0±0	0±0	0±0	0±0	NA	NA
Acinetobacter	0.01±0.03	0.044±0.09	0.038±0.05	0.074±0.11	0.05±0.09	0.1264	0.2200	0.11±0.29	0.018±0.03	0.031±0.05	0.022±0.03	0.023±0.03	0.9216	0.9300
Actinobacillus	0.086±0.06	0.103±0.19	0.17±0.18	0.141±0.09	0.14±0.16	0.1136	0.2100	1.628±1.27	1.856±2.3	0.849±1.01	0.585±0.39	0.119±0.15	0.0004	0.0032
Actinobacillus_A	0.002±0	0.005±0.01	0.005±0.01	0.008±0.01	0.012±0.02	0.8850	0.9100	0.012±0.03	0.027±0.03	0.005±0.01	0.012±0.02	0.008±0.02	0.2071	0.3000
Actinomyces	0±0	0±0	0.002±0	0±0	0.005±0.01	0.0790	0.1700	0.048±0.08	0.045±0.06	0.01±0.02	0.007±0.01	0±0	0.0022	0.0097
Actinomycetaceae_X	0.001±0	0.001±0	0.002±0	0.002±0.01	0.006±0.01	0.1839	0.2900	0.001±0	0.006±0.01	0.016±0.02	0.005±0.01	0.036±0.04	0.0197	0.0470
Actinomycetales_X_X	0±0	0.004±0.01	0.005±0.01	0±0	0.006±0.02	0.4754	0.5700	0±0	0±0	0.002±0	0±0	0±0	0.2281	0.3300
Acutalibacteraceae_X	0.028±0.03	0.039±0.04	0.054±0.05	0.061±0.03	0.057±0.05	0.1475	0.2500	0.032±0.03	0.016±0.02	0.023±0.03	0.035±0.03	0.039±0.05	0.4691	0.5300
Aerococcaceae_X	0.007±0.02	0.011±0.04	0.009±0.02	0.005±0.01	0.012±0.03	0.5128	0.6100	0.101±0.14	0.145±0.15	0.263±0.17	0.073±0.11	0.168±0.26	0.0185	0.0460
Aerococcus	0±0	0±0	0±0	0±0	0.005±0.01	0.0867	0.1700	0±0	0±0	0.015±0.02	0.014±0.03	0.003±0.01	0.0042	0.0160
Agarilytica	0±0	0±0	0.003±0.01	0.03±0.04	0.002±0	0.0007	0.0042	0±0	0±0	0±0	0.006±0.01	0.005±0.02	0.2482	0.3400
Agitococcus	0.153±0.4	0.25±0.32	0.235±0.24	0.108±0.16	0.134±0.15	0.2906	0.4100	0.041±0.04	0.203±0.23	0.18±0.22	0.059±0.06	0.083±0.16	0.1016	0.1700
Akkermansia	0±0	0.005±0.01	0.019±0.04	0.003±0.01	0±0	0.0637	0.1400	0.001±0	0.003±0.01	0.068±0.14	0±0	0.005±0.01	0.0003	0.0025
Alcanivorax_A	0.003±0.01	0.166±0.18	0.055±0.05	0.014±0.02	0.01±0.02	0.0000	0.0000	0±0	0.009±0.01	0.009±0.01	0.004±0.01	0.015±0.03	0.0082	0.0240
Algoriphagus	0±0	0±0	0±0	0.007±0.01	0±0	0.0000	0.0004	0±0	0±0	0±0	0±0	0±0	0.4060	0.4800
Aliarcobacter	0±0	0±0	0.084±0.19	0±0	0±0	0.0000	0.0003	0±0	0±0	0±0	0±0	0±0	NA	NA
Alishewanella	0±0	0.144±0.18	0.994±1.32	0.318±0.43	0.15±0.26	0.0000	0.0000	0.001±0	0.04±0.04	0.388±0.41	0.137±0.22	0.048±0.08	0.0000	0.0001
Alphaproteobacteria_X_X_X	0.247±0.22	0.689±0.36	0.336±0.16	0.442±0.29	0.385±0.23	0.0014	0.0071	0.066±0.06	0.038±0.03	0.057±0.05	0.059±0.02	0.139±0.26	0.2993	0.4000
Anaerofilum	0.002±0	0.006±0.01	0.022±0.02	0.019±0.01	0.008±0.01	0.0019	0.0087	0.048±0.03	0.048±0.03	0.09±0.04	0.076±0.03	0.091±0.07	0.0361	0.0750
Anaerofustis	0.001±0	0±0	0±0	0±0	0±0	0.4243	0.5200	0.004±0.01	0.008±0.01	0.01±0.01	0.006±0.01	0.001±0	0.0443	0.0880
Anaerorhabdus	0±0	0±0	0.001±0	0±0	0.001±0	0.6138	0.6800	0.011±0.01	0.001±0	0.002±0	0.003±0.01	0.001±0	0.1582	0.2500
Anaerosporobacter	0±0	0.001±0	0.001±0	0±0	0±0	0.5491	0.6200	0±0	0.014±0.04	0.01±0.02	0±0	0±0	0.0602	0.1100
Anaerotignum	0±0	0±0	0.001±0	0±0	0.002±0	0.0731	0.1600	0.005±0.01	0.005±0.01	0.012±0.01	0.006±0	0.007±0.01	0.0694	0.1300
Anaerovibrio	0.001±0	0.004±0.01	0.011±0.01	0.015±0.01	0.009±0.01	0.0118	0.0360	0.001±0	0.001±0.01	0.005±0.01	0.006±0.01	0.012±0.02	0.0409	0.0820
Anaerovoracaceae_X	0.009±0.01	0.003±0.01	0.011±0.01	0.005±0.01	0.005±0.01	0.1466	0.2500	0.054±0.05	0.02±0.02	0.018±0.01	0.018±0.03	0±0	0.0006	0.0041
Aquabacterium	0±0	0.029±0.05	0.042±0.05	0.022±0.03	0.001±0	0.0002	0.0013	0±0	0.014±0.02	0.054±0.04	0.01±0.01	0.009±0.03	0.0000	0.0001
Arabia	0±0	0±0	0±0	0±0	0±0	0.4060	0.5100	0±0	0.002±0	0.006±0.01	0.002±0	0.002±0	0.0271	0.0610
Arenimonas	0±0	0.2±0.26	0.026±0.03	0.005±0.01	0±0	0.0000	0.0000	0±0	0.004±0.01	0.001±0	0±0	0.004±0.01	0.6947	0.7300
Aromatoleum	0±0	0.008±0.03	0.002±0.01	0.001±0	0.03±0.04	0.0074	0.0260	0±0	0±0	0.001±0	0.001±0	0.009±0.02	0.0321	0.0690
Asticcacaulis	0±0	0±0	0±0	0.002±0	0±0	0.0145	0.0420	0±0	0±0	0±0	0.001±0	0.001±0	0.0695	0.1300
Bacilli_X_X_X	0.067±0.04	0.049±0.04	0.119±0.09	0.141±0.08	0.086±0.05	0.0024	0.0100	0.022±0.02	0.018±0.02	0.048±0.04	0.037±0.02	0.019±0.02	0.0699	0.1300
Bacillus	0.011±0.04	0±0	0±0	0±0	0±0	0.4060	0.5100	0.064±0.16	0±0	0.002±0.01	0±0	0±0	0.0016	0.0081
Bacillus_AD	0.001±0	0±0	0.001±0	0±0	0±0	0.5491	0.6200	0±0	0±0	0.044±0.08	0±0	0±0	0.0014	0.0076
Bacillus_W	0±0	0±0	0.09±0.16	0±0	0±0	0.0003	0.0018	0.009±0.03	0±0	0±0	0±0	0±0	0.4060	0.4800
Bact-08	0.059±0.05	0.088±0.09	0.061±0.04	0.1±0.05	0.041±0.03	0.0534	0.1200	0.091±0.1	0.078±0.1	0.069±0.05	0.097±0.07	0.015±0.04	0.0063	0.0200
Bacteria_X_X_X_X_X	0.224±0.09	0.29±0.12	0.392±0.13	0.416±0.12	0.299±0.18	0.0023	0.0100	0.268±0.09	0.371±0.14	0.507±0.22	0.452±0.13	0.679±0.25	0.0001	0.0014
Bacteroidaceae_X	5.377±1.76	4.038±1.03	3.938±0.91	3.932±0.83	2.485±0.67	0.0000	0.0004	5.72±2.94	6.177±2.36	4.844±1.49	5.934±1.17	4.591±0.95	0.1037	0.1800
Bacteroidales_X_X	11.604±2.44	10.481±2.21	10.767±2.44	11.511±2.28	9.81±2.96	0.0951	0.1800	10.623±2.08	11.364±2.6	10.852±2.32	13.851±2.07	13.042±2.16	0.0018	0.0088
Bacteroides	0.032±0.03	0.039±0.04	0.048±0.06	0.033±0.02	0.045±0.06	0.9959	1.0000	0.38±0.37	0.329±0.24	0.17±0.21	0.256±0.37	0.016±0.02	0.0005	0.0037
Bacteroidia_X_X_X	0.339±0.27	0.426±0.18	0.494±0.24	0.423±0.14	0.475±0.17	0.1000	0.1900	0.352±0.13	0.519±0.24	0.564±0.21	0.675±0.26	0.563±0.16	0.0063	0.0200
Bdellovibrio	0±0	0.002±0.01	0.001±0	0.005±0.01	0.001±0	0.1973	0.3000	0±0	0±0	0.001±0	0.003±0.01	0.001±0	0.1988	0.2900

Beijerinckiaceae_X	0.02±0.07	0±0	0.006±0.02	0±0	0±0	0.5491	0.6200	0.113±0.28	0±0	0±0	0±0	0±0	0.0003	0.0025
Bibersteinia	0.105±0.12	0.272±0.19	0.336±0.27	0.167±0.15	0.123±0.14	0.0146	0.0420	0.044±0.02	0.32±0.24	0.241±0.15	0.114±0.09	0.067±0.1	0.0000	0.0007
Blastomonas	0±0	0.007±0.02	0.005±0.01	0.009±0.01	0.006±0.01	0.3107	0.4300	0±0	0.001±0	0±0	0±0	0±0	0.4060	0.4800
Blautia_A	0±0	0.004±0.02	0.002±0.01	0.003±0.01	0±0	0.3961	0.5100	0.009±0.02	0.03±0.06	0.07±0.08	0.051±0.1	0.405±0.33	0.0004	0.0032
Bosea	0±0	0.001±0	0.007±0.01	0.001±0	0±0	0.0015	0.0073	0±0	0±0	0±0	0±0	0±0	NA	NA
Brachymonas	0.001±0	0.003±0.01	0.002±0	0.004±0.01	0.001±0	0.4164	0.5200	0.007±0.01	0.016±0.03	0.005±0.01	0.003±0	0±0	0.3347	0.4400
Brevundimonas	0.006±0.01	0.162±0.16	0.059±0.05	0.022±0.02	0.008±0.02	0.0000	0.0005	0.012±0.03	0.086±0.11	0.043±0.04	0.013±0.02	0.022±0.05	0.0007	0.0048
BRH-c57	0±0	0.004±0.01	0.005±0.01	0±0	0.014±0.03	0.0886	0.1700	0±0	0.001±0	0±0	0±0	0.002±0.01	0.2383	0.3300
Burkholderiaceae_X	0.123±0.15	0.135±0.1	0.06±0.05	0.061±0.05	0.04±0.03	0.1089	0.2000	0.028±0.07	0.026±0.06	0.013±0.02	0.021±0.01	0.015±0.02	0.2589	0.3500
Butyrivibrio	0.069±0.08	0.238±0.26	0.585±0.41	0.829±0.52	0.812±0.59	0.0000	0.0000	0.307±0.29	0.279±0.34	0.785±0.35	0.731±0.46	0.487±0.29	0.0019	0.0088
C941	0.035±0.05	0.149±0.25	0.256±0.15	0.199±0.1	0.11±0.06	0.0000	0.0002	1.044±0.54	1.05±0.8	1.007±0.34	0.88±0.44	0.489±0.2	0.0230	0.0530
CAG-180	0±0	0.003±0.01	0.007±0.01	0.002±0	0.005±0.01	0.1855	0.2900	0.004±0.01	0.006±0.01	0.009±0.01	0.011±0.01	0.005±0.01	0.0868	0.1500
CAG-312	0±0	0.002±0.01	0.026±0.04	0.01±0.02	0.009±0.02	0.0047	0.0180	0±0	0±0	0.009±0.02	0.001±0	0.001±0.01	0.0152	0.0390
CAG-313	0.045±0.04	0.047±0.03	0.075±0.03	0.069±0.04	0.058±0.03	0.1703	0.2700	0.004±0.01	0.015±0.02	0.037±0.02	0.024±0.02	0.01±0.01	0.0001	0.0015
CAG-354	0.241±0.15	0.565±0.49	0.588±0.31	0.703±0.34	0.773±0.55	0.0105	0.0330	0.598±0.36	0.458±0.45	0.726±0.31	0.631±0.3	1.005±0.37	0.0061	0.0200
CAG-462	3.729±0.84	2.669±0.66	3.359±1.29	2.925±0.74	2.579±0.47	0.0052	0.0200	5.496±1.59	4.887±2.14	3.886±0.63	4.974±1.25	3.192±1.2	0.0036	0.0140
CAG-465	0.004±0.01	0.012±0.03	0±0	0.007±0.01	0.023±0.04	0.1670	0.2700	0.001±0	0.01±0.03	0±0	0.002±0.01	0.002±0	0.9093	0.9200
CAG-475	0.003±0	0.01±0.01	0.008±0.01	0.012±0.01	0.008±0.01	0.1275	0.2200	0±0	0.002±0	0.001±0	0.003±0	0.002±0	0.2340	0.3300
CAG-495	0.356±0.25	0.839±0.66	0.587±0.17	0.59±0.24	0.564±0.35	0.0286	0.0740	0.193±0.07	0.26±0.18	0.2±0.12	0.21±0.13	0.25±0.19	0.8798	0.9000
CAG-826_X	0.213±0.14	0.277±0.27	0.468±0.29	0.377±0.17	0.395±0.3	0.0830	0.1700	0.06±0.06	0.095±0.09	0.212±0.13	0.17±0.15	0.116±0.08	0.0061	0.0200
CAG-873	0±0	0±0	0.005±0.02	0.004±0.01	0.003±0.01	0.2083	0.3100	0.011±0.03	0.037±0.09	0.041±0.07	0.017±0.03	0±0	0.1544	0.2500
CAG-878	0.053±0.07	0.15±0.11	0.119±0.09	0.141±0.07	0.105±0.1	0.0383	0.0930	0.034±0.03	0.03±0.04	0.087±0.04	0.062±0.02	0.085±0.08	0.0081	0.0240
Campylobacter	0.01±0.02	0.004±0.01	0.007±0.01	0.02±0.02	0.032±0.03	0.0242	0.0650	0.007±0.02	0.001±0	0.026±0.03	0.017±0.02	0.038±0.03	0.0069	0.0210
Campylobacter_A	0.015±0.01	0.034±0.03	0.054±0.05	0.037±0.03	0.026±0.05	0.1369	0.2400	0.041±0.04	0.08±0.08	0.033±0.06	0.036±0.05	0.009±0.02	0.0206	0.0490
Campylobacter_B	0±0	0.001±0	0±0	0.003±0.01	0.001±0	0.6679	0.7200	0.006±0.01	0.005±0.01	0.002±0.01	0.001±0	0±0	0.1650	0.2600
Campylobacteraceae_X	0.001±0	0.002±0.01	0.007±0.02	0.008±0.01	0.005±0.02	0.3210	0.4400	0.01±0.02	0.009±0.02	0.018±0.03	0.007±0.02	0.001±0.01	0.3802	0.4700
Caviibacter	0.007±0.02	0±0	0.008±0.01	0.01±0.02	0.001±0	0.1011	0.1900	0.015±0.02	0.016±0.04	0.026±0.05	0.006±0.01	0.01±0.03	0.2784	0.3800
Cellvibrio	0±0	0.031±0.04	0.056±0.05	0.074±0.08	0.005±0.01	0.0000	0.0000	0.002±0.01	0.011±0.01	0.024±0.05	0.021±0.04	0.004±0.01	0.1555	0.2500
Cellvibrionaceae_X	0±0	0±0	0.002±0	0.018±0.03	0±0	0.0025	0.0110	0±0	0±0	0±0	0.008±0.02	0.001±0	0.0121	0.0330
Chishuiella	0±0	0.01±0.02	0.008±0.01	0.01±0.02	0.009±0.02	0.0994	0.1900	0±0	0.014±0.03	0.005±0.02	0.01±0.02	0.015±0.02	0.0977	0.1700
CHKCI001	0±0	0.001±0	0.004±0.01	0.002±0.01	0±0	0.4754	0.5700	0±0	0±0	0±0	0.003±0.01	0.001±0	0.2281	0.3300
Christensenellales_X_X	0.001±0	0.001±0	0±0	0.004±0	0±0	0.0413	0.0980	0.008±0.01	0.032±0.04	0.027±0.02	0.022±0.02	0.026±0.03	0.2157	0.3100
Chryseobacterium	0±0	0.031±0.05	0.029±0.05	0.066±0.08	0.032±0.08	0.0177	0.0490	0.011±0.04	0.001±0	0.004±0.01	0.014±0.03	0.016±0.02	0.4850	0.5500
Chryseobacterium_D	0.005±0.02	0±0	0.001±0	0.011±0.03	0.004±0.01	0.4004	0.5100	0±0	0.001±0	0.001±0	0.003±0.01	0.002±0	0.6120	0.6600
Cloacibacterium	0.016±0.03	0.011±0.03	0.043±0.03	0.019±0.02	0.008±0.01	0.0105	0.0330	0.072±0.06	0.165±0.27	0.152±0.11	0.046±0.02	0.024±0.05	0.0006	0.0040
Clostridia_X_X_X	1.027±0.42	1.513±0.89	1.681±0.28	1.971±0.36	2.359±1.55	0.0011	0.0057	2.071±0.68	2.234±1.01	3.119±1	3.165±1.23	5.273±2.73	0.0001	0.0014
Clostridium	0.006±0.01	0.004±0.01	0.008±0.01	0.016±0.05	0.031±0.06	0.7380	0.7700	0.021±0.03	0.045±0.09	0.031±0.06	0.024±0.05	0.004±0.01	0.1321	0.2200
Clostridium_A	0±0	0±0	0±0	0±0	0.001±0	0.5491	0.6200	0.011±0.01	0.011±0.02	0.019±0.02	0.003±0.01	0.004±0.01	0.0490	0.0950
Clostridium_L	0.001±0	0.005±0.01	0.003±0.01	0±0	0±0	0.1859	0.2900	0±0	0±0	0.002±0.01	0±0	0±0	0.4060	0.4800
Clostridium_M	0.001±0	0±0	0±0	0±0	0.022±0.02	0.0002	0.0013	0±0	0±0	0±0	0±0	0±0	NA	NA
Clostridium_N	0.013±0.01	0.011±0.03	0.014±0.03	0.018±0.03	0.057±0.07	0.0487	0.1100	0.004±0.01	0.003±0.01	0.006±0.01	0.02±0.03	0±0	0.0999	0.1700
Comamonas	0.004±0.01	0.023±0.05	0.021±0.03	0.022±0.03	0.018±0.03	0.7814	0.8100	0.01±0.02	0.003±0.01	0±0	0.002±0.01	0.003±0.01	0.3682	0.4600
Corynebacterium	0±0	0±0	0±0	0±0	0±0	0.4060	0.5100	0±0	0.017±0.03	0.013±0.04	0±0	0±0	0.0053	0.0190
CP2B	0±0	0±0	0±0	0±0	0±0	0.5491	0.6200	0.001±0	0±0	0.007±0.01	0.001±0	0±0	0.0018	0.0086

Cytophagales_X_X	0.001±0	0.001±0	0.006±0.01	0.001±0	0.018±0.04	0.0835	0.1700	0±0	0.001±0	0.008±0.02	0.005±0.01	0±0	0.0020	0.0093
Denitrobacterium	0±0	0±0	0.001±0	0±0	0±0	0.5491	0.6200	0.001±0	0.003±0	0.002±0.01	0.001±0	0.001±0	0.7873	0.8100
Dermatophilaceae_X	0.003±0.01	0.008±0.03	0.007±0.02	0.007±0.02	0.022±0.05	0.8780	0.9000	0.04±0.05	0.028±0.05	0.044±0.04	0.01±0.02	0.034±0.08	0.1051	0.1800
Desulfobacterota_X_X_X_X	0.005±0.01	0.01±0.01	0.013±0.01	0.009±0.01	0.004±0.01	0.1176	0.2100	0.004±0.01	0.005±0.01	0.006±0.01	0.006±0.01	0.001±0	0.3620	0.4500
Desulfovibrio	0±0	0.001±0	0±0	0±0	0.001±0	0.5491	0.6200	0.011±0.03	0.007±0.02	0.002±0.01	0.007±0.01	0±0	0.1742	0.2700
Desulfovibrionaceae_X	0.002±0	0.001±0	0.011±0.01	0.012±0.01	0.001±0	0.0006	0.0036	0.036±0.04	0.044±0.03	0.058±0.04	0.035±0.02	0.055±0.02	0.1432	0.2300
Devosia	0±0	0.016±0.04	0.007±0.01	0.004±0.01	0.006±0.01	0.1682	0.2700	0±0	0.011±0.02	0.023±0.03	0.01±0.01	0.008±0.02	0.0321	0.0690
Dialister_A	0±0	0±0	0.001±0	0±0	0±0	0.3824	0.5000	0.001±0	0.004±0.01	0.012±0.01	0.005±0.01	0.001±0	0.0238	0.0540
Dichelobacter	0.045±0.06	0.044±0.04	0.07±0.04	0.055±0.06	0.029±0.04	0.1417	0.2400	0.041±0.05	0.079±0.06	0.103±0.07	0.046±0.04	0.03±0.05	0.0052	0.0190
Dietzia	0±0	0.003±0.01	0±0	0±0	0±0	0.0145	0.0420	0.002±0.01	0.01±0.02	0.013±0.02	0.001±0	0.001±0	0.0557	0.1100
Dongia	0±0	0.015±0.02	0.018±0.03	0.033±0.05	0.02±0.02	0.0035	0.0140	0±0	0.001±0	0.003±0.01	0.003±0.01	0.006±0.01	0.0539	0.1000
Dorea	0±0	0±0	0±0	0±0	0±0	NA	NA	0±0	0±0	0.006±0.01	0.002±0	0±0	0.0006	0.0040
DTU053	0.003±0.01	0.005±0.01	0.025±0.02	0.024±0.02	0.01±0.01	0.0063	0.0230	0.086±0.09	0.03±0.03	0.101±0.04	0.102±0.08	0.143±0.09	0.0103	0.0290
Duncaniella	0±0	0.005±0.01	0.001±0	0±0	0±0	0.1654	0.2700	0.002±0.01	0.023±0.05	0.001±0	0.004±0.01	0.001±0	0.3944	0.4800
Dysgonomonadaceae_X	0±0	0±0	0±0	0.008±0.01	0.023±0.06	0.0160	0.0460	0.003±0.01	0.013±0.04	0±0	0.001±0	0±0	0.9007	0.9100
Echinicola	0±0	0.02±0.02	0.023±0.02	0.013±0.02	0.006±0.01	0.0001	0.0009	0.002±0.01	0.004±0.01	0.019±0.02	0.015±0.02	0.008±0.01	0.0065	0.0210
Eggerthellaceae_X	0.001±0	0.001±0	0±0	0.003±0.01	0.001±0	0.0939	0.1800	0.007±0.01	0.01±0.02	0.037±0.03	0.017±0.01	0.008±0.02	0.0027	0.0110
Elizabethkingia	0±0	0.003±0.01	0.001±0	0.005±0.01	0.005±0.01	0.2328	0.3400	0±0	0.001±0	0±0	0.001±0	0.002±0	0.3763	0.4600
Emergencia	0±0	0±0	0±0	0.001±0	0.001±0	0.7224	0.7600	0.006±0.01	0.003±0.01	0.008±0.01	0.002±0.01	0±0	0.3077	0.4100
Empedobacter	0±0	0.011±0.02	0.01±0.01	0.026±0.04	0.013±0.02	0.0120	0.0370	0±0	0.017±0.03	0.015±0.02	0.018±0.03	0.038±0.04	0.0409	0.0820
Endomicrobium	0.009±0.01	0.024±0.04	0.008±0.01	0.005±0.01	0.008±0.02	0.9430	0.9500	0.003±0.01	0.006±0.02	0.006±0.01	0.005±0.01	0.021±0.05	0.6462	0.6900
Endomicrobium_A	0.181±0.11	0.119±0.11	0.062±0.05	0.073±0.03	0.085±0.06	0.0104	0.0330	0.335±0.07	0.141±0.08	0.101±0.06	0.12±0.07	0.096±0.07	0.0000	0.0006
Enterobacterales_X_X	0.329±0.41	0.064±0.05	0.175±0.13	0.138±0.1	0.157±0.09	0.0252	0.0670	0.094±0.13	0.036±0.03	0.04±0.02	0.058±0.05	0.124±0.11	0.2585	0.3500
Enterobacteriaceae_X	0.819±0.49	0.293±0.24	0.433±0.26	0.326±0.32	0.219±0.25	0.0017	0.0077	0.5±0.31	0.187±0.11	0.213±0.09	0.223±0.2	0.429±0.21	0.0028	0.0110
Enterococcaceae_X	0±0	0.013±0.04	0.006±0.01	0.009±0.02	0±0	0.1220	0.2200	0.014±0.02	0.039±0.04	0.038±0.04	0.014±0.01	0.003±0.01	0.0127	0.0340
Enterococcus	0±0	0.011±0.02	0.011±0.02	0.015±0.02	0.039±0.07	0.0174	0.0490	0±0	0.008±0.01	0.02±0.03	0.008±0.02	0.072±0.08	0.0404	0.0820
Erysipelothrix	0.008±0.01	0.021±0.02	0.031±0.02	0.051±0.04	0.033±0.04	0.0057	0.0210	0.062±0.06	0.061±0.04	0.129±0.1	0.056±0.08	0.081±0.12	0.0435	0.0870
Erysipelotrichaceae_X	0.003±0	0.004±0.02	0.008±0.01	0.007±0.01	0.002±0	0.1170	0.2100	0.024±0.02	0.014±0.02	0.022±0.02	0.019±0.01	0.009±0.01	0.0889	0.1600
Eubacterium_C	0.004±0.01	0.004±0.01	0±0	0.007±0.01	0.014±0.02	0.1222	0.2200	0.001±0	0±0	0±0	0±0	0.001±0	0.5491	0.6000
Eubacterium_E	0.011±0.02	0.08±0.06	0.096±0.05	0.07±0.03	0.07±0.06	0.0002	0.0017	0.03±0.02	0.065±0.07	0.124±0.08	0.077±0.06	0.094±0.07	0.0086	0.0250
Eubacterium_F	0.123±0.15	0.085±0.08	0.136±0.05	0.236±0.12	0.148±0.13	0.0036	0.0140	0.361±0.15	0.105±0.06	0.224±0.08	0.214±0.07	0.344±0.19	0.0000	0.0007
Eubacterium_Q	0.033±0.04	0.088±0.04	0.076±0.06	0.084±0.04	0.177±0.13	0.0072	0.0250	0.136±0.08	0.113±0.14	0.149±0.14	0.126±0.13	0.206±0.12	0.2286	0.3300
Eubacterium_S	0.006±0.01	0.024±0.01	0.031±0.03	0.016±0.02	0.031±0.02	0.0069	0.0250	0.042±0.03	0.044±0.03	0.079±0.05	0.044±0.04	0.077±0.06	0.0837	0.1500
F0040	0.395±0.27	0.304±0.23	0.294±0.28	0.133±0.1	0.265±0.33	0.1285	0.2200	4.337±4.06	3.809±2.93	1.379±1.72	1.108±1.2	0.23±0.44	0.0000	0.0011
F0058	0.001±0	0.004±0.01	0.003±0.01	0.002±0	0.002±0	0.7575	0.7900	0.033±0.03	0.04±0.05	0.029±0.04	0.018±0.02	0.003±0	0.0183	0.0460
F0422	0.012±0.02	0.012±0.02	0.023±0.03	0.008±0.01	0.07±0.15	0.4709	0.5700	1.006±1.28	0.503±0.69	0.192±0.29	0.092±0.12	0.237±0.63	0.0014	0.0076
F0428	0.006±0.01	0.025±0.02	0.029±0.03	0.04±0.02	0.028±0.03	0.0067	0.0250	0.086±0.06	0.105±0.09	0.124±0.05	0.094±0.05	0.167±0.09	0.1446	0.2300
F082	2.144±1.09	2.36±1.67	1.884±0.68	1.218±0.65	2.254±1.09	0.0832	0.1700	0.387±0.38	0.112±0.06	0.162±0.07	0.144±0.07	0.509±0.85	0.0002	0.0019
FD2005	0.031±0.04	0.165±0.1	0.31±0.28	0.323±0.2	0.216±0.11	0.0000	0.0002	0.181±0.13	0.13±0.11	0.27±0.14	0.181±0.13	0.212±0.13	0.0301	0.0650
Fermentimonas	0.003±0.01	0.002±0	0.006±0.01	0.001±0	0.003±0.01	0.9355	0.9400	0.073±0.1	0.044±0.07	0.018±0.02	0.017±0.03	0±0	0.0164	0.0420
Fibrobacter	3.059±1.31	4.19±1.9	3.686±0.72	4.367±1.78	2.463±1.04	0.0071	0.0250	3.689±1.18	3.925±1.82	4.416±2.25	4.199±1.68	3.37±1.29	0.6959	0.7300
Fibrobacter_A	0.05±0.03	0.088±0.07	0.059±0.06	0.078±0.06	0.13±0.09	0.0304	0.0780	0.007±0.01	0.018±0.03	0.013±0.02	0.018±0.01	0.012±0.03	0.3244	0.4300
Firm-04	0.006±0.01	0.001±0	0.016±0.01	0.009±0.01	0.008±0.02	0.0445	0.1000	0.127±0.08	0.059±0.06	0.086±0.07	0.071±0.04	0.113±0.05	0.0075	0.0220
Firm-16	0.006±0.01	0.006±0.01	0.001±0	0.004±0	0.002±0	0.4148	0.5200	0.047±0.07	0.013±0.02	0.017±0.03	0.021±0.04	0.005±0.01	0.3780	0.4600

Firmicutes_A_X_X_X_X	0.007±0.01	0.002±0.01	0.002±0	0.003±0.01	0.005±0.01	0.6183	0.6800	0.009±0.01	0.012±0.02	0.02±0.01	0.016±0.01	0.013±0.01	0.1300	0.2200
Firmicutes_B_X_X_X_X	0.006±0.01	0.003±0.01	0.001±0	0.001±0	0.003±0.01	0.1210	0.2200	0.095±0.1	0.017±0.02	0.03±0.03	0.018±0.02	0.006±0.01	0.0001	0.0016
Flavobacteriaceae_X	0.284±0.34	0.269±0.21	0.347±0.36	0.307±0.25	0.26±0.2	0.9206	0.9300	0.207±0.13	0.206±0.2	0.174±0.11	0.131±0.11	0.088±0.11	0.0479	0.0940
Flavobacteriales_X_X	0.032±0.03	0.04±0.03	0.033±0.01	0.051±0.05	0.063±0.08	0.4627	0.5600	0.033±0.02	0.084±0.03	0.088±0.12	0.094±0.06	0.049±0.03	0.0003	0.0025
Flavobacterium	0±0	0.11±0.17	0.177±0.26	0.198±0.37	0.094±0.19	0.0000	0.0005	0.001±0	0.003±0.01	0.017±0.03	0.057±0.08	0.036±0.06	0.0011	0.0062
Flavobacterium_A	0±0	0.054±0.09	0.151±0.2	0.124±0.22	0.02±0.03	0.0001	0.0007	0±0	0.006±0.01	0.018±0.02	0.025±0.02	0.002±0.01	0.0000	0.0006
Flexilinea	0.024±0.03	0.057±0.06	0.073±0.06	0.058±0.03	0.056±0.05	0.0601	0.1300	0.049±0.04	0.072±0.05	0.153±0.09	0.077±0.03	0.137±0.1	0.0148	0.0390
Fodinicurvata	0.005±0.01	0.008±0.01	0.001±0	0.001±0	0±0	0.0217	0.0590	0.001±0	0.001±0	0.001±0	0±0	0±0	0.7224	0.7500
Fusicatenibacter	0.006±0.01	0.031±0.05	0.051±0.05	0.045±0.03	0.05±0.04	0.0027	0.0110	0.021±0.03	0.003±0.01	0.033±0.02	0.015±0.02	0.03±0.03	0.0060	0.0200
Fusobacterium	0±0	0±0	0.001±0	0.002±0.01	0±0	0.2281	0.3300	0±0	0.037±0.06	0.003±0.01	0.002±0.01	0±0	0.1514	0.2400
Fusobacterium_A	0.054±0.05	0.109±0.13	0.111±0.13	0.055±0.05	0.045±0.04	0.6154	0.6800	0.699±0.73	0.747±0.57	0.368±0.32	0.436±0.6	0.046±0.06	0.0006	0.0040
Fusobacterium_C	0.044±0.05	0.119±0.12	0.189±0.2	0.14±0.09	0.12±0.11	0.0352	0.0880	0.544±0.54	0.858±0.71	0.84±0.81	1.152±1.66	0.128±0.13	0.0066	0.0210
Gammaproteobacteria_X_X_X	0.622±0.49	0.195±0.14	0.429±0.23	0.346±0.41	0.227±0.16	0.0023	0.0100	0.192±0.09	0.061±0.04	0.15±0.06	0.141±0.14	0.228±0.15	0.0020	0.0093
Gastranaerophilaceae_X	0.011±0.02	0.032±0.06	0.009±0.02	0.025±0.02	0.015±0.02	0.3208	0.4400	0±0	0.002±0.01	0.006±0.01	0.006±0.01	0.006±0.01	0.1747	0.2700
GCA-2733575	0±0	0±0	0.002±0	0.001±0	0±0	0.0648	0.1400	0±0	0±0	0.002±0.01	0±0	0±0	0.0145	0.0380
GCA-900066135	0±0	0±0	0±0	0±0	0±0	NA	NA	0±0	0.002±0.01	0.007±0.01	0±0	0±0	0.0406	0.0820
GCA-900066495	0±0	0.001±0	0.002±0	0.002±0	0±0	0.6177	0.6800	0±0	0.003±0.01	0.018±0.02	0.002±0	0.002±0.01	0.0001	0.0016
GCA-900066905	0±0	0.007±0.01	0.003±0	0.008±0.01	0.003±0.01	0.0181	0.0500	0.002±0	0.001±0	0.02±0.02	0.013±0.01	0.03±0.04	0.0001	0.0017
GCA-900066995	0.028±0.03	0.122±0.33	0.031±0.03	0.032±0.01	0.039±0.1	0.3180	0.4400	0.073±0.05	0.058±0.12	0.114±0.13	0.057±0.08	0.258±0.37	0.1629	0.2500
Gemella_A	0±0	0±0	0.004±0.01	0.006±0.01	0.001±0	0.0009	0.0050	0.002±0.01	0.005±0.01	0.009±0.02	0.005±0.01	0.004±0.01	0.2152	0.3100
Gemmatimonadaceae_X	0±0	0.001±0	0.001±0	0±0	0±0	0.5491	0.6200	0.001±0	0.005±0.01	0±0	0±0	0±0	0.0022	0.0097
Gemmobacter_A	0±0	0.023±0.08	0.005±0.01	0.001±0.01	0.04±0.06	0.0019	0.0086	0±0	0±0	0.001±0	0±0	0.03±0.04	0.0002	0.0020
Geothermobacter	0.001±0	0±0	0.004±0.01	0.002±0	0.001±0	0.0315	0.0800	0.013±0.02	0.013±0.02	0.012±0.01	0.019±0.02	0.006±0.01	0.3245	0.4300
Gluconobacter	0±0	0.003±0.01	0.004±0.01	0.008±0.01	0.005±0.02	0.1705	0.2700	0±0	0.004±0.01	0.002±0.01	0.004±0.01	0.004±0.01	0.4661	0.5300
Glutamicibacter	0±0	0±0	0.003±0.01	0.002±0.01	0.003±0.01	0.3864	0.5000	0±0	0.003±0.01	0±0	0.006±0.01	0.016±0.03	0.1205	0.2000
GN02-873	0.006±0.02	0.014±0.03	0.023±0.04	0.008±0.01	0.006±0.01	0.1958	0.3000	0.006±0.01	0.017±0.02	0.039±0.05	0.004±0.01	0.001±0	0.0014	0.0076
Gottschalkiaceae_X	0.002±0.01	0±0	0±0	0±0	0.001±0	0.6374	0.7000	0.003±0.01	0±0	0±0	0±0	0±0	0.0100	0.0280
GWE2-31-10	0.013±0.02	0.01±0.01	0.011±0.01	0.014±0.02	0.008±0.01	0.8940	0.9100	0.005±0.01	0.009±0.01	0.009±0.01	0.004±0.01	0.005±0.01	0.2197	0.3200
GWF2-44-16	0±0	0.004±0.01	0.014±0.02	0.001±0	0.005±0.01	0.0307	0.0780	0±0	0±0	0±0	0±0	0±0	NA	NA
Halomonas_A	0±0	0±0	0.01±0.02	0.002±0.01	0±0	0.0018	0.0082	0±0	0±0	0.001±0	0±0	0±0	0.5491	0.6000
Helcococcaceae_X	0±0	0±0	0±0	0±0	0±0	0.4060	0.5100	0.027±0.03	0.014±0.02	0.003±0.01	0.001±0	0±0	0.0001	0.0018
Helcococcus	0.001±0	0.003±0.01	0.013±0.01	0.006±0.01	0.001±0	0.0143	0.0420	0.002±0	0.02±0.02	0.03±0.02	0.012±0.01	0.009±0.02	0.0008	0.0049
Hepatobacter	0±0	0.007±0.01	0.065±0.12	0.052±0.09	0.044±0.08	0.0004	0.0025	0±0	0±0	0.005±0.01	0.003±0.01	0.006±0.02	0.1705	0.2600
Humitalea	0.002±0.01	0±0	0.002±0	0±0	0±0	0.0695	0.1500	0.046±0.14	0±0	0±0	0±0	0±0	0.0145	0.0380
Hungatella_A	0.057±0.04	0.375±0.41	0.596±0.25	0.809±0.35	0.601±0.46	0.0000	0.0001	0.153±0.16	0.237±0.29	0.597±0.32	0.577±0.4	0.59±0.27	0.0001	0.0016
Hyphomonas	0±0	0±0	0.002±0	0.004±0.01	0.004±0.01	0.0860	0.1700	0±0	0±0	0±0	0.001±0	0.002±0	0.1768	0.2700
Inquilinus	0.027±0.09	0±0	0±0	0±0	0±0	0.5491	0.6200	0.5±1.3	0±0	0±0	0±0	0±0	0.0003	0.0025
Kapabacteriaceae_X	0.004±0.01	0.001±0	0.003±0.01	0±0	0.002±0.01	0.7541	0.7900	0.001±0	0.009±0.01	0.008±0.01	0.014±0.02	0.003±0.01	0.0583	0.1100
Kingella	0.014±0.02	0.061±0.09	0.121±0.09	0.054±0.04	0.092±0.12	0.0029	0.0120	0.389±0.31	1.306±1.31	0.77±1.32	0.344±0.38	0.112±0.21	0.0003	0.0025
Kiritimatiellae_X_X_X	0.471±0.2	0.509±0.17	0.515±0.22	0.393±0.16	0.491±0.29	0.5056	0.6000	0.094±0.14	0.152±0.06	0.187±0.08	0.137±0.11	0.208±0.25	0.0207	0.0490
KLE1796	0±0	0±0	0±0	0±0	0±0	0.5491	0.6200	0±0	0.008±0.01	0.009±0.01	0.004±0.01	0±0	0.0066	0.0210
Kocuria	0.025±0.09	0±0	0±0	0±0	0±0	0.4060	0.5100	0.378±0.94	0±0	0±0	0±0	0±0	0.0003	0.0025
Lachnoanaerobaculum	0±0	0.004±0.01	0.03±0.04	0.017±0.01	0.007±0.01	0.0002	0.0017	0.034±0.05	0.061±0.08	0.106±0.07	0.051±0.03	0.111±0.07	0.0027	0.0110
Lachnospira	0±0	0.004±0.01	0.012±0.02	0.016±0.01	0.034±0.06	0.0009	0.0050	0.006±0.02	0.01±0.03	0.002±0	0.024±0.01	0.011±0.02	0.0000	0.0006

Lachnospiraceae_X	0.963±0.37	2.966±2.27	3.839±1.18	4.902±2.08	3.697±1.35	0.0000	0.0001	3.264±1.85	3.133±1.87	5.755±1.24	4.273±1.05	4.887±1.47	0.0008	0.0049
Lachnospirales_X_X	0.045±0.04	0.043±0.05	0.07±0.05	0.061±0.04	0.078±0.04	0.1069	0.2000	0.097±0.07	0.082±0.06	0.111±0.06	0.093±0.03	0.104±0.04	0.6550	0.6900
Lactobacillales_X_X	0±0	0±0	0.002±0.01	0.002±0	0.001±0	0.3809	0.5000	0.021±0.03	0.013±0.02	0.03±0.04	0.009±0.01	0.005±0.02	0.3633	0.4500
Lactobacillus_F	0.004±0.01	0±0	0±0	0±0	0±0	0.5491	0.6200	0±0	0±0	0.001±0	0.001±0	0.004±0.01	0.4243	0.5000
Lactococcus	0±0	0.01±0.02	0.006±0.01	0.007±0.01	0.01±0.02	0.2007	0.3000	0±0	0.012±0.02	0.016±0.02	0.017±0.02	0.059±0.08	0.0125	0.0340
Lactonifactor	0±0	0±0	0.007±0.02	0.008±0.02	0.005±0.02	0.3864	0.5000	0.005±0.02	0.01±0.02	0.012±0.02	0.012±0.02	0.009±0.02	0.4047	0.4800
Lancefieldella	0±0	0±0	0.001±0.01	0±0	0±0	0.4060	0.5100	0.002±0.01	0.007±0.01	0.014±0.02	0.009±0.01	0.001±0	0.0138	0.0370
Lawsonibacter	0.007±0.02	0.003±0.01	0.005±0.01	0±0	0.001±0	0.4542	0.5500	0±0	0±0	0±0	0±0	0±0	NA	NA
Leaf454	0.019±0.02	0.076±0.25	0.111±0.13	0.166±0.1	0.132±0.08	0.0000	0.0001	0.011±0.01	0.15±0.35	0.187±0.29	0.188±0.07	0.106±0.08	0.0000	0.0002
Lenti-01	0.042±0.03	0.143±0.19	0.185±0.11	0.115±0.05	0.1±0.09	0.0013	0.0067	0.043±0.03	0.071±0.07	0.158±0.11	0.107±0.06	0.098±0.11	0.0092	0.0260
Lentimicrobium	0.764±0.36	0.708±0.22	0.661±0.27	0.71±0.25	0.47±0.27	0.1659	0.2700	0.533±0.19	0.418±0.18	0.366±0.16	0.541±0.17	0.339±0.13	0.0183	0.0460
Leptotrichiaceae_X	0.002±0.01	0±0	0.001±0	0.002±0	0±0	0.1984	0.3000	0.003±0.01	0.006±0.01	0.008±0.02	0.011±0.02	0±0	0.2551	0.3500
Leuconostoc	0±0	0.004±0.01	0.016±0.02	0.014±0.02	0.019±0.03	0.0247	0.0660	0±0	0.014±0.03	0.038±0.06	0.024±0.03	0.127±0.16	0.0088	0.0260
Lysobacter	0.005±0.02	0±0	0.006±0.02	0±0	0±0	0.5491	0.6200	0.075±0.18	0±0	0±0	0±0	0±0	0.0003	0.0025
Mailhella	0.013±0.01	0.004±0.01	0.031±0.03	0.019±0.01	0.004±0.01	0.0003	0.0021	0.026±0.03	0.056±0.03	0.056±0.03	0.054±0.04	0.032±0.02	0.0353	0.0730
Mannheimia	0.011±0.02	0.021±0.02	0.031±0.06	0.015±0.02	0.03±0.05	0.8739	0.9000	0.145±0.13	0.626±0.61	0.293±0.49	0.17±0.17	0.013±0.04	0.0000	0.0007
Marinilabiliaceae_X	0.002±0.01	0±0	0.004±0.01	0.002±0	0±0	0.0825	0.1700	0.004±0.01	0.004±0.01	0.007±0.02	0.007±0.02	0±0	0.4552	0.5200
Massilia_B	0±0	0±0	0.334±0.35	0±0	0±0	0.0000	0.0000	0±0	0±0	0.029±0.06	0±0	0±0	0.0000	0.0008
Massilibacteroides	0.141±0.08	0.156±0.1	0.129±0.1	0.061±0.04	0.081±0.07	0.0254	0.0670	0.171±0.1	0.33±0.28	0.175±0.12	0.114±0.09	0.034±0.05	0.0004	0.0032
Metamycoplasma	0.001±0	0.003±0.01	0.005±0.01	0±0	0.002±0.01	0.8900	0.9100	0.004±0.01	0.009±0.01	0.007±0.01	0.001±0	0±0	0.0332	0.0700
Methylobacterium	0±0	0.004±0.01	0.001±0	0.001±0	0±0	0.7224	0.7600	0.015±0.05	0±0	0.002±0	0.002±0	0.001±0	0.6646	0.7000
Micavibrionaceae_X	0±0	0.002±0.01	0±0	0±0	0.004±0.01	0.0094	0.0310	0±0	0±0	0±0	0±0	0.001±0	0.0867	0.1500
Micrococcaceae_X	0±0	0.001±0	0.001±0	0.003±0.01	0±0	0.7224	0.7600	0±0	0±0	0.003±0.01	0±0	0.014±0.05	0.7224	0.7500
Monoglobales_X_X	0±0	0.003±0.01	0.002±0	0.006±0.01	0.013±0.03	0.5931	0.6700	0±0	0±0	0±0	0±0	0±0	NA	NA
Monoglobus	0.047±0.06	0.074±0.04	0.107±0.09	0.074±0.04	0.048±0.04	0.0495	0.1100	0.046±0.02	0.041±0.03	0.072±0.04	0.045±0.02	0.055±0.04	0.3601	0.4500
Moraxella	0.681±1.64	0.411±0.49	0.621±0.41	0.278±0.17	0.791±1.03	0.3272	0.4500	1.083±0.85	1.658±1.41	2.124±2.9	0.636±0.4	0.414±0.55	0.0026	0.0110
Moraxella_A	0.002±0	0±0	0±0	0±0	0.004±0.01	0.3497	0.4700	0.005±0.02	0.004±0.01	0.01±0.01	0.002±0	0±0	0.0323	0.0690
Moraxella_C	0±0	0±0	0±0	0±0	0±0	NA	NA	0±0	0.047±0.09	0.173±0.26	0±0	0.013±0.03	0.0015	0.0079
Moraxellaceae_X	0.099±0.08	0.124±0.08	0.216±0.22	0.079±0.05	0.03±0.04	0.0077	0.0270	0.012±0.03	0.096±0.09	0.089±0.08	0.039±0.04	0.038±0.05	0.0056	0.0190
Mucilaginibacter	0±0	0.003±0	0.002±0	0.004±0.01	0.007±0.01	0.2847	0.4000	0±0	0±0	0±0	0±0	0±0	0.4060	0.4800
Muribaculaceae_X	0.062±0.09	0.161±0.35	0.308±0.12	0.297±0.14	0.177±0.09	0.0000	0.0003	1.902±1.18	2.443±1.5	2.037±0.78	2.183±1.75	0.873±0.42	0.0046	0.0170
Ndongobacter	0±0	0±0	0±0	0.002±0.01	0±0	0.2383	0.3400	0.004±0.01	0.01±0.02	0.009±0.01	0.001±0	0±0	0.0089	0.0260
Negativicutes_X_X_X	0.086±0.05	0.101±0.06	0.139±0.05	0.154±0.06	0.054±0.04	0.0006	0.0036	0.051±0.06	0.025±0.02	0.052±0.01	0.046±0.01	0.022±0.04	0.0187	0.0460
Neisseria_B	0.001±0	0.009±0.03	0.005±0.01	0.003±0.01	0.001±0	0.4753	0.5700	0.009±0.02	0.021±0.03	0.011±0.02	0.006±0.01	0.003±0.01	0.0925	0.1600
Neisseriaceae_X	0.032±0.04	0.023±0.02	0.043±0.03	0.045±0.03	0.037±0.05	0.2628	0.3700	0.144±0.09	0.106±0.08	0.145±0.11	0.083±0.04	0.078±0.08	0.2787	0.3800
Neorhizobium	0.034±0.12	0.019±0.04	0.017±0.02	0.011±0.02	0.009±0.02	0.3768	0.5000	0.416±1.05	0.005±0.01	0.013±0.02	0.006±0.01	0.008±0.02	0.2924	0.3900
Niveispirillum	0±0	0.003±0.01	0±0	0.004±0.01	0.027±0.04	0.0013	0.0065	0±0	0.002±0	0±0	0.001±0	0±0	0.0603	0.1100
NS-102	0±0	0.017±0.04	0.001±0	0.002±0	0.001±0	0.1641	0.2700	0±0	0.006±0.01	0.009±0.02	0.01±0.02	0.002±0	0.0883	0.1600
OEMR01	0.001±0	0.001±0	0.001±0.01	0.001±0	0.001±0	0.7931	0.8200	0.019±0.02	0.043±0.06	0.075±0.13	0.014±0.04	0.018±0.03	0.0297	0.0650
OLB17	0±0	0±0	0±0	0±0	0±0	NA	NA	0.009±0.03	0.005±0.01	0.004±0.01	0.002±0.01	0±0	0.6469	0.6900
Olegusella	0±0	0±0	0±0	0.001±0	0±0	0.4060	0.5100	0.002±0.01	0.005±0.02	0.015±0.02	0.005±0.02	0.009±0.03	0.3659	0.4600
Olsenella	0.004±0.01	0.02±0.02	0.011±0.02	0.015±0.02	0.006±0.02	0.1074	0.2000	0.021±0.04	0.065±0.06	0.095±0.07	0.039±0.05	0.073±0.11	0.0194	0.0470
Opitutaceae_X	1.479±0.87	1.893±1.39	1.051±0.63	0.822±0.5	1.228±0.56	0.1316	0.2300	0.884±0.45	0.706±0.52	0.652±0.48	0.636±0.36	1.257±1.34	0.4519	0.5200
Opitutus	0±0	0±0	0.016±0.03	0.015±0.03	0±0	0.0015	0.0073	0±0	0±0	0±0	0±0	0±0	NA	NA

Oribacterium	0.001±0	0.008±0.01	0.022±0.02	0.045±0.03	0.03±0.03	0.0001	0.0010	0±0	0±0	0.005±0.01	0.013±0.02	0.003±0.01	0.0230	0.0530
Orrella	0.01±0.03	0.04±0.04	0.006±0.01	0.002±0.01	0.002±0.01	0.0009	0.0049	0.007±0.02	0.003±0.01	0.001±0	0±0	0±0	0.3497	0.4500
Oscillibacter	0.007±0.01	0.017±0.02	0.015±0.02	0.02±0.01	0.015±0.03	0.2568	0.3600	0.013±0.02	0.013±0.02	0.042±0.06	0.03±0.03	0.046±0.05	0.0903	0.1600
Oscillospiraceae_X	0.361±0.22	0.283±0.22	0.449±0.28	0.791±0.69	0.964±0.85	0.0014	0.0068	0.485±0.26	0.54±0.31	0.713±0.32	0.875±0.39	1.34±0.57	0.0002	0.0025
Oscillospirales_X_X	0.137±0.06	0.16±0.07	0.285±0.09	0.413±0.13	0.291±0.17	0.0000	0.0003	0.165±0.06	0.125±0.09	0.257±0.15	0.26±0.07	0.247±0.12	0.0014	0.0075
Paenibacillus_J	0±0	0.001±0	0±0	0.003±0.01	0.007±0.01	0.1616	0.2700	0±0	0.003±0.01	0.008±0.02	0±0	0.004±0.01	0.2039	0.3000
Paeniclostridium	0±0	0±0	0±0	0±0	0±0	NA	NA	0±0	0.002±0	0.011±0.01	0.002±0	0±0	0.0067	0.0210
PALSA-1355	0.004±0.01	0.009±0.01	0.011±0.02	0.005±0.01	0.01±0.02	0.9333	0.9400	0.008±0.01	0.016±0.04	0.01±0.01	0.01±0.01	0.005±0.01	0.5333	0.6000
Paludibacteraceae_X	0.328±0.33	0.295±0.26	0.329±0.18	0.204±0.13	0.143±0.14	0.0742	0.1600	0.051±0.07	0.064±0.05	0.07±0.05	0.101±0.08	0.089±0.22	0.0772	0.1400
Paramesorhizobium	0±0	0.01±0.02	0.004±0.01	0.005±0.01	0.003±0.01	0.0709	0.1500	0.091±0.22	0.002±0.01	0±0	0.003±0.01	0.005±0.01	0.0467	0.0920
Paramuribaculum	0.002±0	0.008±0.02	0.002±0	0.004±0.01	0.002±0	0.9798	0.9800	0.003±0.01	0.023±0.02	0.016±0.02	0.017±0.02	0.004±0.01	0.0123	0.0330
Parapedobacter	0±0	0.002±0.01	0.005±0.01	0.006±0.01	0.003±0.01	0.1267	0.2200	0.006±0.02	0±0	0.001±0	0±0	0±0	0.5491	0.6000
Paraprevotella	0.237±0.25	0.151±0.14	0.225±0.11	0.281±0.27	0.151±0.09	0.1761	0.2800	0.129±0.12	0.327±0.15	0.325±0.23	0.452±0.22	0.119±0.14	0.0002	0.0019
Parvibaculum	0.005±0.01	0.095±0.08	0.092±0.08	0.235±0.36	0.054±0.07	0.0001	0.0007	0.008±0.03	0.07±0.06	0.08±0.09	0.131±0.16	0.044±0.07	0.0004	0.0033
Parvimonas	0.002±0	0.013±0.02	0.004±0.01	0.009±0.01	0.001±0	0.0804	0.1700	0.036±0.03	0.073±0.07	0.05±0.05	0.02±0.02	0.005±0.01	0.0024	0.0100
Pasteurellaceae_X	0.356±0.45	0.629±1.12	1.054±0.8	0.524±0.31	0.43±0.48	0.0082	0.0280	1.644±1.16	4.806±5.11	4.308±2.64	1.116±0.46	0.637±0.57	0.0000	0.0007
Pauljensenia	0±0	0±0	0±0	0±0	0±0	NA	NA	0.034±0.06	0.014±0.03	0.009±0.01	0.002±0.01	0±0	0.1154	0.1900
Pedosphaeraceae_X	0.401±0.23	0.123±0.1	0.147±0.12	0.12±0.09	0.141±0.12	0.0133	0.0400	0.283±0.17	0.088±0.07	0.08±0.07	0.095±0.07	0.11±0.05	0.0018	0.0088
PeH17	0.06±0.04	0.136±0.13	0.115±0.05	0.166±0.1	0.079±0.07	0.0057	0.0210	0.342±0.19	0.361±0.17	0.457±0.23	0.388±0.14	0.374±0.2	0.5319	0.6000
Peptostreptococcaceae_X	0±0	0.002±0	0.001±0	0.005±0.01	0.001±0	0.0511	0.1200	0.093±0.08	0.079±0.07	0.025±0.03	0.014±0.01	0.002±0.01	0.0001	0.0016
Peptostreptococcales_X_X	0.026±0.04	0.069±0.11	0.055±0.04	0.028±0.02	0.016±0.02	0.0680	0.1500	0.204±0.13	0.294±0.21	0.196±0.16	0.131±0.17	0.026±0.04	0.0008	0.0049
Pigmentiphaga	0.052±0.12	0.174±0.28	0.007±0.01	0.006±0.01	0±0	0.0000	0.0003	0±0	0±0	0.001±0	0±0	0.009±0.03	0.5491	0.6000
Pirellulaceae_X	0±0	0±0	0.002±0	0.001±0	0±0	0.2383	0.3400	0±0	0±0	0.007±0.01	0.002±0	0±0	0.0275	0.0610
Pirellulales_X_X	0.039±0.03	0.064±0.05	0.241±0.22	0.164±0.12	0.158±0.17	0.0016	0.0077	0.05±0.07	0.116±0.18	0.232±0.24	0.239±0.19	0.295±0.21	0.0005	0.0038
Planctomycetes_X_X_X	0.006±0.01	0.004±0.01	0.012±0.03	0.011±0.01	0.015±0.03	0.6144	0.6800	0.001±0	0.007±0.02	0.013±0.02	0.014±0.02	0.022±0.03	0.1534	0.2400
Planctomycetota_X_X_X_X	0.075±0.07	0.002±0	0.013±0.02	0.019±0.03	0.009±0.01	0.0100	0.0330	0.067±0.06	0.015±0.02	0.024±0.03	0.019±0.02	0.035±0.03	0.2172	0.3200
Porphyromonas	0.261±0.22	0.456±0.51	0.481±0.49	0.23±0.15	0.281±0.36	0.5905	0.6700	1.298±1.09	2.135±1.44	0.982±0.74	1.014±0.96	0.25±0.45	0.0009	0.0051
Prevotella	37.736±7.82	23.263±5.92	20.763±3.73	22.903±6.74	31.913±13.77	0.0000	0.0004	19.158±4.62	14.973±3.84	14.021±3.47	17.068±3.03	21.326±5.14	0.0013	0.0075
Prevotellamassilia	0.264±0.11	0.199±0.11	0.18±0.09	0.166±0.09	0.18±0.04	0.2188	0.3200	0.391±0.13	0.258±0.14	0.256±0.11	0.224±0.09	0.189±0.13	0.0055	0.0190
Prolixibacteraceae_X	0.452±0.24	0.322±0.2	0.559±0.28	0.741±0.69	0.459±0.3	0.1686	0.2700	1.134±0.72	1.156±0.52	1.555±0.45	2.253±1.01	1.845±1.47	0.0056	0.0190
Prosthecobacter	0±0	0.036±0.12	0±0	0.013±0.02	0.04±0.07	0.0005	0.0031	0±0	0±0	0.002±0	0.014±0.02	0.054±0.07	0.0001	0.0018
Proteiniclasticum	0±0	0.007±0.02	0.031±0.03	0.016±0.02	0.051±0.09	0.0001	0.0007	0.004±0.01	0.006±0.01	0.088±0.1	0.051±0.07	0.039±0.08	0.0000	0.0003
Proteobacteria_X_X_X_X	0.167±0.08	0.528±0.35	0.21±0.06	0.174±0.1	0.096±0.12	0.0001	0.0007	0.207±0.3	0.113±0.1	0.125±0.11	0.085±0.08	0.065±0.08	0.0731	0.1300
Pseudaminobacter	0±0	0.008±0.03	0.008±0.02	0.003±0.01	0.001±0	0.3961	0.5100	0±0	0.012±0.03	0.045±0.04	0.006±0.01	0.012±0.03	0.0000	0.0003
Pseudomonadaceae_X	0.013±0.05	0.069±0.08	0.313±0.59	0.056±0.09	0.01±0.02	0.0000	0.0004	0.221±0.56	0.003±0.01	0.035±0.05	0.01±0.02	0.006±0.02	0.0404	0.0820
Pseudomonadales_X_X	0.002±0.01	0.004±0.01	0.008±0.01	0.017±0.02	0.002±0	0.0102	0.0330	0.001±0	0.002±0.01	0.002±0	0.008±0.01	0.002±0.01	0.2524	0.3500
Pseudomonas_A	0.071±0.17	0.296±0.31	0.425±0.49	0.123±0.22	0.1±0.16	0.0001	0.0006	0.052±0.08	0.09±0.08	0.16±0.21	0.058±0.07	0.049±0.07	0.0213	0.0500
Pseudomonas_B	0.006±0.02	0.005±0.01	0.006±0.01	0.005±0.01	0±0	0.2091	0.3100	0.001±0	0.005±0.01	0.002±0	0.001±0	0.002±0	0.8761	0.8900
Pseudomonas_D	0±0	0.073±0.11	0.165±0.27	0.011±0.03	0.008±0.02	0.0000	0.0001	0±0	0.001±0	0.002±0.01	0.001±0	0.002±0.01	0.9007	0.9100
Pseudomonas_E	0.008±0.01	0.044±0.09	0.055±0.07	0.061±0.08	0.028±0.06	0.2931	0.4100	0±0	0±0	0±0	0.001±0	0.002±0.01	0.5491	0.6000
Pseudomonas_F	0±0	0.003±0.01	0±0	0±0	0±0	0.0003	0.0018	0±0	0.001±0	0±0	0±0	0±0	0.0867	0.1500
Pseudomonas_M	0.002±0.01	0.128±0.26	0.316±0.34	0.147±0.18	0.092±0.15	0.0009	0.0050	0.061±0.15	0±0	0.013±0.03	0.01±0.02	0.018±0.04	0.2513	0.3500
Pygmaiobacter	0.002±0	0.003±0.01	0.016±0.01	0.009±0.01	0.004±0.01	0.0038	0.0150	0.027±0.03	0.105±0.14	0.071±0.13	0.038±0.04	0.001±0	0.0003	0.0025
Pyramidobacter	0.033±0.05	0.062±0.11	0.128±0.09	0.073±0.04	0.04±0.01	0.0003	0.0023	0.314±0.11	0.432±0.34	0.288±0.11	0.394±0.16	0.225±0.17	0.0351	0.0730

RC9	3.426±0.97	7.646±2.86	4.927±0.94	4.426±1.4	2.416±1.05	0.0000	0.0000	2.946±0.69	3.211±1.5	2.946±0.52	3.424±0.6	2.369±0.66	0.0270	0.0610
RF16	4.644±1.72	4.853±1.57	3.833±0.76	4.045±2.75	4.728±1.63	0.1412	0.2400	1.233±1.07	0.875±0.43	0.706±0.22	1.27±0.82	1.777±1.28	0.0123	0.0330
Rhizobiaceae_X	0.021±0.07	0.009±0.03	0.005±0.01	0.007±0.01	0.003±0.01	0.4772	0.5700	0.274±0.69	0.005±0.01	0±0	0.001±0	0.015±0.03	0.0336	0.0710
Rhizobiales_X_X	0.004±0.01	0.012±0.03	0.014±0.01	0.016±0.01	0.006±0.01	0.0391	0.0950	0.005±0.01	0.003±0.01	0.004±0.01	0.003±0.01	0±0	0.3595	0.4500
Rhodospirillaceae_X	0±0	0.021±0.03	0.023±0.03	0.006±0.01	0.007±0.01	0.0031	0.0130	0±0	0.001±0	0.001±0	0.002±0	0±0	0.1984	0.2900
Riemerella	0±0	0±0	0.008±0.02	0.004±0.01	0±0	0.0172	0.0490	0.002±0.01	0.005±0.01	0.012±0.01	0.007±0.01	0±0	0.0213	0.0500
Roseomonas	0±0	0.01±0.02	0.01±0.01	0.014±0.02	0.006±0.01	0.1108	0.2000	0±0	0±0	0±0	0±0	0±0	0.4060	0.4800
Rothia	0±0	0.009±0.02	0.007±0.01	0.003±0.01	0±0	0.0859	0.1700	0±0	0.014±0.02	0.001±0	0±0	0±0	0.0560	0.1100
RUG131	0.196±0.07	0.169±0.15	0.237±0.22	0.254±0.12	0.27±0.17	0.2026	0.3000	0.086±0.04	0.095±0.07	0.109±0.06	0.101±0.04	0.057±0.05	0.2483	0.3400
RUG163	0.277±0.13	0.443±0.52	0.457±0.41	0.828±0.51	0.257±0.15	0.0140	0.0420	0.456±0.25	0.458±0.31	0.662±0.28	0.694±0.27	0.147±0.13	0.0000	0.0008
RUG350	0.169±0.08	0.092±0.07	0.146±0.1	0.17±0.07	0.198±0.11	0.0383	0.0930	0.064±0.06	0.078±0.09	0.238±0.43	0.112±0.12	0.03±0.03	0.0173	0.0440
Ruminiclostridium	0.031±0.03	0.101±0.14	0.267±0.11	0.306±0.14	0.258±0.2	0.0000	0.0001	0.041±0.02	0.078±0.07	0.287±0.12	0.181±0.07	0.128±0.05	0.0000	0.0000
Ruminiclostridium_C	0.117±0.09	0.195±0.15	0.398±0.22	0.42±0.15	0.692±1.36	0.0000	0.0005	0.558±0.23	0.578±0.24	0.722±0.28	0.639±0.23	1.211±0.82	0.0232	0.0530
Ruminiclostridium_D	0.012±0.01	0.02±0.02	0.026±0.01	0.02±0.01	0.009±0.01	0.0169	0.0480	0.003±0.01	0.002±0.01	0.005±0.01	0.008±0.01	0.006±0.02	0.3422	0.4400
Ruminiclostridium_F	0.003±0.01	0.028±0.03	0.007±0.02	0.007±0.01	0.014±0.03	0.1402	0.2400	0±0	0.005±0.02	0.002±0.01	0±0	0.018±0.04	0.1697	0.2600
Ruminococcaceae_X	0.039±0.04	0.066±0.04	0.082±0.09	0.16±0.2	0.162±0.12	0.0083	0.0280	0.437±0.21	0.212±0.2	0.222±0.12	0.303±0.2	0.389±0.26	0.0241	0.0550
Ruminococcus	0.036±0.04	0.053±0.07	0.081±0.07	0.1±0.05	0.117±0.12	0.0409	0.0980	0.113±0.08	0.092±0.13	0.225±0.39	0.189±0.16	0.339±0.2	0.0071	0.0210
Ruminococcus_C	0.007±0.01	0.034±0.03	0.04±0.04	0.064±0.04	0.091±0.05	0.0001	0.0007	0.038±0.06	0.053±0.08	0.08±0.04	0.098±0.08	0.12±0.09	0.0058	0.0200
Ruminococcus_D	0.111±0.09	0.264±0.24	0.404±0.29	0.522±0.28	0.496±0.35	0.0001	0.0009	0.446±0.4	0.276±0.28	0.595±0.35	0.338±0.23	0.758±0.29	0.0014	0.0076
Ruminococcus_F	0.014±0.02	0.07±0.07	0.066±0.06	0.123±0.06	0.104±0.08	0.0003	0.0019	0.188±0.12	0.169±0.16	0.223±0.22	0.201±0.12	0.314±0.26	0.5818	0.6300
Saccharibacillus	0±0	0.001±0	0.002±0	0.006±0.01	0.005±0.01	0.4190	0.5200	0±0	0±0	0±0	0.002±0.01	0.002±0.01	0.3509	0.4500
Saccharicrinis	0±0	0.005±0.02	0.007±0.01	0.014±0.02	0.019±0.03	0.0381	0.0930	0±0	0±0	0.016±0.03	0.017±0.03	0.017±0.04	0.0003	0.0029
Saccharimonadaceae_X	0.112±0.08	0.157±0.14	0.212±0.1	0.171±0.13	0.192±0.18	0.3474	0.4700	0.049±0.06	0.055±0.05	0.157±0.05	0.115±0.05	0.07±0.06	0.0001	0.0017
Saccharimonadales_X_X	0.027±0.04	0.054±0.05	0.076±0.08	0.063±0.06	0.042±0.06	0.2272	0.3300	0.002±0.01	0.001±0	0.013±0.03	0.014±0.02	0.006±0.01	0.1761	0.2700
Saccharofermentans	0.047±0.03	0.158±0.12	0.228±0.15	0.217±0.09	0.138±0.07	0.0000	0.0004	0.209±0.1	0.306±0.39	0.349±0.11	0.33±0.16	0.386±0.14	0.0017	0.0086
Schwartzia	0.416±0.28	0.22±0.09	0.412±0.14	0.439±0.17	0.309±0.19	0.0070	0.0250	0.243±0.16	0.093±0.07	0.167±0.06	0.14±0.05	0.16±0.13	0.0515	0.1000
Selenomonadaceae_X	0.062±0.07	0.115±0.13	0.205±0.13	0.169±0.14	0.094±0.08	0.0091	0.0300	0.025±0.04	0.024±0.04	0.026±0.03	0.021±0.02	0.061±0.06	0.3307	0.4300
Selenomonas_A	0±0	0±0	0.001±0	0.005±0.01	0.001±0	0.0015	0.0073	0±0	0±0	0±0	0.002±0	0±0	0.2281	0.3300
Selenomonas_B	0±0	0.007±0.01	0.031±0.02	0.021±0.02	0.021±0.03	0.0003	0.0018	0±0	0.017±0.03	0.007±0.01	0.002±0	0.004±0.01	0.0197	0.0470
Serratia	0±0	0.01±0.02	0.008±0.01	0.011±0.01	0.011±0.02	0.2173	0.3200	0.001±0	0.004±0.02	0±0	0.004±0.01	0.001±0	0.6543	0.6900
Shinella	0±0	0.047±0.16	0.003±0.01	0.002±0.01	0.005±0.02	0.6646	0.7200	0±0	0.006±0.01	0.02±0.02	0.014±0.02	0.015±0.04	0.0081	0.0240
Simonsiella	0.014±0.02	0.002±0.01	0.038±0.05	0.029±0.04	0.044±0.09	0.2325	0.3400	0.086±0.07	0.074±0.09	0.087±0.11	0.064±0.05	0.077±0.22	0.2309	0.3300
Sneathia	0±0	0.028±0.07	0.01±0.02	0.048±0.11	0±0	0.0987	0.1900	0±0	0.014±0.03	0.003±0.01	0.01±0.03	0.002±0.01	0.4700	0.5300
Soleaferrea	0.009±0.01	0.044±0.05	0.108±0.13	0.078±0.05	0.058±0.05	0.0007	0.0042	0.046±0.09	0.05±0.04	0.136±0.08	0.066±0.06	0.239±0.17	0.0005	0.0035
Sphaerochaeta	0.005±0.01	0.004±0.01	0.068±0.04	0.044±0.04	0.029±0.02	0.0000	0.0001	0±0	0±0	0.009±0.01	0.009±0.01	0.002±0	0.0002	0.0024
Sphaerochaeta_A	0.004±0.01	0.012±0.01	0.036±0.02	0.034±0.02	0.017±0.01	0.0000	0.0002	0.013±0.02	0.007±0.01	0.014±0.02	0.02±0.01	0.016±0.02	0.1849	0.2800
Sphaerochaetaceae_X	0.054±0.03	0.121±0.12	0.169±0.08	0.186±0.11	0.243±0.22	0.0004	0.0027	0.106±0.08	0.118±0.13	0.137±0.05	0.13±0.04	0.148±0.13	0.3010	0.4000
Sphingobacterium	0±0	0.085±0.14	0.143±0.12	0.177±0.17	0.097±0.15	0.0001	0.0009	0.001±0	0.009±0.02	0.01±0.02	0.02±0.03	0.016±0.02	0.1349	0.2200
Sphingomonas	0±0	0.049±0.06	0.03±0.04	0.055±0.05	0.033±0.05	0.0030	0.0120	0±0	0.023±0.02	0.007±0.01	0.011±0.02	0.007±0.02	0.0024	0.0100
Sphingopyxis_A	0.011±0.03	0.008±0.02	0.01±0.02	0.001±0	0±0	0.1527	0.2500	0.088±0.16	0.001±0	0.004±0.01	0.001±0	0±0	0.0004	0.0032
Spirochaetia_X_X_X	0.953±0.38	1.275±1.09	1.203±0.75	1.245±0.63	1.202±0.68	0.8944	0.9100	1.283±0.74	0.964±0.69	1.357±0.48	0.808±0.32	0.937±0.75	0.0340	0.0710
Spirochaetota_X_X_X_X	0.012±0.01	0.025±0.03	0.016±0.02	0.023±0.02	0.017±0.02	0.6086	0.6800	0.047±0.03	0.034±0.05	0.035±0.03	0.034±0.03	0.039±0.03	0.7644	0.7900
Stenotrophomonas	0.004±0.01	1.431±1.28	1.669±2.27	0.759±0.69	0.308±0.46	0.0000	0.0001	0.015±0.04	0.095±0.08	0.119±0.11	0.09±0.08	0.147±0.24	0.0053	0.0190
Streptococcus	0.049±0.12	0.058±0.08	0.073±0.07	0.06±0.06	0.219±0.48	0.2749	0.3900	0.984±0.92	1.051±1.25	0.545±0.53	0.173±0.12	0.101±0.13	0.0001	0.0016

Succiniclasticum	1.879±0.91	0.836±1.11	1.531±0.7	1.195±0.48	0.712±0.29	0.0000	0.0003	3.267±1.11	3.575±1.63	4.186±1.65	4.565±1.79	5.868±3.67	0.1440	0.2300
Succinimonas	0.386±0.18	0.104±0.06	0.225±0.18	0.265±0.21	0.168±0.09	0.0023	0.0100	0.098±0.08	0.04±0.05	0.063±0.06	0.094±0.07	0.286±0.21	0.0007	0.0048
Succinivibrio	0.12±0.07	0.241±0.22	0.292±0.13	0.338±0.23	0.384±0.39	0.0009	0.0050	0.05±0.04	0.034±0.05	0.029±0.02	0.064±0.05	0.053±0.05	0.2372	0.3300
Sutterella	0.001±0	0.002±0.01	0±0	0±0	0.004±0.01	0.7224	0.7600	0.004±0.01	0.003±0.01	0.001±0	0.001±0	0±0	0.6817	0.7200
Syner-01	0±0	0±0	0.001±0	0.004±0.01	0±0	0.2281	0.3300	0.001±0	0.002±0.01	0±0	0.038±0.12	0±0	0.1783	0.2700
Synergistales_X_X	0.002±0	0.001±0	0.014±0.02	0.015±0.02	0.007±0.01	0.0841	0.1700	0.005±0.01	0.006±0.01	0.021±0.02	0.014±0.01	0.002±0	0.0036	0.0140
Synergistes	0.001±0	0±0	0±0	0±0	0±0	0.4060	0.5100	0.006±0.01	0.002±0.01	0.01±0.01	0.005±0.01	0.001±0	0.0020	0.0093
SZUA-359	0.177±0.13	0.515±0.35	0.378±0.22	0.523±0.38	0.464±0.52	0.0250	0.0670	0.097±0.08	0.198±0.15	0.249±0.15	0.436±0.52	0.219±0.22	0.0158	0.0400
Tannerella	0.001±0	0.001±0	0.003±0.01	0±0	0±0	0.3584	0.4800	0.001±0	0.01±0.02	0.007±0.01	0.006±0.01	0.001±0	0.3466	0.4400
Tannerellaceae_X	0.031±0.04	0.023±0.02	0.022±0.02	0.017±0.02	0.011±0.02	0.3373	0.4600	0.034±0.03	0.085±0.06	0.025±0.03	0.038±0.03	0.008±0.02	0.0007	0.0048
TF01-11	0.006±0.02	0.016±0.02	0.019±0.02	0.012±0.02	0.007±0.01	0.0960	0.1800	0.031±0.03	0.132±0.13	0.045±0.03	0.027±0.02	0.027±0.05	0.0056	0.0190
Tissierellaceae_X	0±0	0±0	0.002±0.01	0.001±0	0±0	0.2482	0.3500	0.004±0.02	0.001±0	0.002±0.01	0.001±0	0.001±0	0.9543	0.9500
Tissierellales_X_X	0.032±0.03	0.042±0.06	0.075±0.04	0.074±0.05	0.054±0.04	0.0207	0.0570	0.178±0.15	0.163±0.08	0.203±0.08	0.168±0.07	0.118±0.06	0.1396	0.2300
Treponema_A	0.001±0	0.001±0	0.003±0.01	0.002±0	0±0	0.1635	0.2700	0.002±0.01	0.004±0.01	0.02±0.05	0.02±0.07	0±0	0.1811	0.2700
Treponema_B	0.003±0.01	0.004±0.01	0.006±0.01	0.007±0.01	0.007±0.01	0.4526	0.5500	0.049±0.07	0.022±0.03	0.029±0.04	0.059±0.11	0.004±0.01	0.0250	0.0570
Treponema_C	0.006±0.01	0.012±0.02	0.005±0.01	0.007±0.01	0.02±0.02	0.1347	0.2300	0.015±0.02	0.01±0.02	0.018±0.01	0.009±0.01	0.004±0.01	0.0383	0.0790
Treponema_D	0.074±0.04	0.111±0.07	0.088±0.05	0.072±0.03	0.121±0.07	0.1948	0.3000	0.103±0.06	0.076±0.06	0.092±0.05	0.038±0.02	0.044±0.03	0.0048	0.0170
Treponemataceae_X	0.096±0.06	0.223±0.14	0.245±0.15	0.224±0.12	0.192±0.12	0.0109	0.0340	0.246±0.22	0.195±0.15	0.265±0.08	0.19±0.11	0.184±0.13	0.1760	0.2700
Treponematales_X_X	0.028±0.03	0.097±0.13	0.086±0.04	0.088±0.04	0.058±0.05	0.0014	0.0069	0.07±0.06	0.15±0.18	0.194±0.08	0.128±0.07	0.091±0.08	0.0038	0.0140
Tumebacillus	0.001±0	0±0	0.006±0.01	0.001±0	0.001±0	0.1236	0.2200	0.001±0	0±0	0.001±0.01	0±0	0±0	0.7224	0.7500
UBA1020	0.001±0	0.017±0.04	0±0	0.006±0.01	0±0	0.0670	0.1500	0±0	0.002±0.01	0±0	0±0	0.001±0	0.5491	0.6000
UBA1033	0.001±0	0.013±0.02	0.019±0.03	0.136±0.27	0.016±0.03	0.0007	0.0042	0.009±0.01	0.01±0.02	0.033±0.03	0.085±0.17	0.128±0.15	0.0042	0.0160
UBA1067	2.503±1.15	1.785±0.51	1.898±0.55	1.637±0.51	2.231±1.03	0.1211	0.2200	0.542±0.34	0.393±0.23	0.447±0.1	0.463±0.17	0.851±0.86	0.0564	0.1100
UBA1174	0.07±0.1	0.138±0.18	0.075±0.05	0.039±0.03	0.062±0.03	0.2253	0.3300	0.044±0.12	0.018±0.02	0.022±0.02	0.015±0.01	0.084±0.25	0.9302	0.9300
UBA1191	0±0	0.008±0.02	0.004±0.01	0.007±0.01	0.003±0.01	0.3167	0.4400	0.027±0.04	0.024±0.02	0.046±0.03	0.016±0.01	0.005±0.01	0.0065	0.0210
UBA1258	0.033±0.03	0.185±0.21	0.225±0.09	0.297±0.18	0.206±0.13	0.0000	0.0004	0.228±0.16	0.198±0.13	0.401±0.13	0.297±0.14	0.205±0.11	0.0039	0.0150
UBA1361	0.057±0.04	0.067±0.06	0.086±0.08	0.116±0.05	0.123±0.07	0.0414	0.0980	0.07±0.04	0.066±0.03	0.114±0.05	0.094±0.02	0.047±0.05	0.0062	0.0200
UBA1394	0.001±0	0.001±0	0.006±0.01	0.006±0.01	0.007±0.01	0.0930	0.1800	0.01±0.01	0.012±0.01	0.039±0.04	0.019±0.02	0.05±0.04	0.0016	0.0083
UBA1436	0.133±0.14	0.534±0.27	0.256±0.14	0.325±0.2	0.27±0.15	0.0004	0.0025	0.094±0.13	0.181±0.1	0.089±0.04	0.128±0.1	0.226±0.16	0.0003	0.0027
UBA1532	0.019±0.02	0.028±0.02	0.027±0.03	0.019±0.01	0.005±0.01	0.0102	0.0330	0.039±0.02	0.033±0.03	0.026±0.02	0.02±0.01	0.009±0.01	0.0104	0.0290
UBA1547	0±0	0.012±0.02	0.005±0.01	0.001±0	0±0	0.0429	0.1000	0±0	0.001±0	0±0	0±0	0±0	0.4060	0.4800
UBA1711	0.42±0.23	0.318±0.11	0.357±0.2	0.367±0.17	0.279±0.35	0.0643	0.1400	0.278±0.18	0.132±0.1	0.173±0.07	0.273±0.14	0.183±0.15	0.0518	0.1000
UBA1829	0.273±0.14	0.441±0.25	0.746±0.79	0.544±0.26	0.167±0.13	0.0001	0.0009	0.089±0.08	0.099±0.1	0.178±0.07	0.134±0.04	0.193±0.35	0.0023	0.0100
UBA2450	0.128±0.31	0.037±0.03	0.045±0.04	0.052±0.03	0.08±0.06	0.3286	0.4500	0.036±0.08	0.02±0.02	0.027±0.03	0.042±0.04	0.021±0.03	0.3376	0.4400
UBA3206	0.076±0.05	0.124±0.13	0.095±0.07	0.183±0.15	0.188±0.12	0.0270	0.0700	0.021±0.03	0.025±0.04	0.024±0.02	0.026±0.02	0.031±0.02	0.4418	0.5100
UBA4179	0.018±0.03	0.061±0.2	0.054±0.08	0.055±0.05	0.092±0.11	0.0600	0.1300	0.031±0.06	0.025±0.09	0.02±0.02	0.072±0.05	0.042±0.06	0.0274	0.0610
UBA4658	0±0	0.002±0.01	0.001±0	0±0	0±0	0.2383	0.3400	0.006±0.02	0.012±0.03	0±0	0±0	0±0	0.4373	0.5100
UBA5124	0.98±0.21	1.673±0.54	1.167±0.43	1.32±0.49	2.431±1.02	0.0000	0.0004	1.097±0.26	1.082±0.4	0.922±0.32	1.378±0.47	2.183±0.96	0.0001	0.0017
UBA5194	0.008±0.02	0.004±0.01	0.007±0.02	0.006±0.01	0±0	0.0120	0.0370	0.005±0.01	0.009±0.01	0.02±0.02	0.003±0.01	0.005±0.01	0.3263	0.4300
UBA5946	0.005±0.02	0.013±0.02	0.009±0.02	0.005±0.01	0.005±0.02	0.7826	0.8100	0±0	0±0	0±0	0±0	0±0	NA	NA
UBA636	0.001±0	0±0	0.005±0.01	0.002±0	0.001±0	0.1605	0.2600	0.003±0.01	0.009±0.01	0.014±0.02	0.015±0.05	0±0	0.0070	0.0210
UBA6984	0.012±0.02	0.02±0.02	0.018±0.02	0.027±0.02	0.029±0.03	0.3467	0.4700	0±0	0±0	0±0	0±0	0±0	NA	NA
UBA7182	0±0	0±0	0.004±0.01	0.005±0.01	0.203±0.32	0.0001	0.0010	0.006±0.02	0.018±0.05	0.005±0.01	0.005±0.01	0.008±0.01	0.6926	0.7300
UBA733	0.091±0.06	0.082±0.05	0.083±0.07	0.063±0.06	0.085±0.08	0.7284	0.7700	0.009±0.02	0.006±0.01	0.011±0.02	0.009±0.01	0.012±0.02	0.7292	0.7500

UBA7862	0.05±0.03	0.085±0.05	0.088±0.05	0.118±0.06	0.063±0.07	0.0280	0.0730	0.014±0.01	0.012±0.02	0.021±0.01	0.021±0.02	0.025±0.03	0.4600	0.5300
UBA8416	0.049±0.05	0.033±0.02	0.064±0.04	0.053±0.03	0.044±0.03	0.2304	0.3400	0.01±0.02	0.008±0.01	0.015±0.01	0.017±0.02	0.018±0.02	0.3385	0.4400
UBA8525	0.023±0.03	0.034±0.04	0.105±0.11	0.09±0.07	0.052±0.04	0.0545	0.1200	0.001±0	0±0	0±0	0.011±0.03	0.004±0.01	0.3370	0.4400
UBA8953	0.154±0.11	0.222±0.09	0.214±0.08	0.278±0.08	0.188±0.06	0.0325	0.0820	0.086±0.04	0.124±0.09	0.108±0.05	0.186±0.09	0.088±0.05	0.0101	0.0280
UBA932_X	0.062±0.04	0.062±0.1	0.108±0.06	0.103±0.06	0.02±0.04	0.0011	0.0058	0.079±0.1	0.096±0.1	0.167±0.1	0.224±0.09	0.003±0.01	0.0000	0.0006
UBA9983_A_X_X	0±0	0.006±0.02	0±0	0.136±0.21	0.059±0.11	0.0000	0.0000	0±0	0±0	0±0	0.015±0.02	0.006±0.01	0.0010	0.0058
V5-8f	0±0	0±0	0±0	0±0	0.002±0	0.0867	0.1700	0.002±0.01	0.003±0.01	0.053±0.09	0.002±0	0.002±0.01	0.0299	0.0650
Vampirovibrionia_X_X_X	0.002±0.01	0.004±0.01	0±0	0.003±0.01	0±0	0.7224	0.7600	0.003±0.01	0±0	0.001±0	0.001±0	0.006±0.02	0.9007	0.9100
Varibaculum_A	0±0	0±0	0.002±0	0±0	0.001±0	0.0603	0.1300	0.007±0.01	0.003±0.01	0.005±0.01	0.004±0.01	0.002±0.01	0.3102	0.4100
Veillonella	0.009±0.01	0.001±0	0.007±0.01	0.007±0.01	0.059±0.13	0.3621	0.4800	0.221±0.27	0.057±0.08	0.024±0.03	0.042±0.07	0.133±0.35	0.0154	0.0400
Veillonella_A	0.024±0.08	0.017±0.02	0.013±0.02	0.01±0.01	0.001±0	0.0383	0.0930	0.604±0.74	0.238±0.22	0.04±0.04	0.178±0.46	0.015±0.04	0.0001	0.0017
Victivallis	0.026±0.03	0.031±0.03	0.049±0.05	0.046±0.02	0.033±0.03	0.1994	0.3000	0.007±0.01	0.006±0.01	0.009±0.01	0.011±0.01	0.013±0.03	0.6423	0.6900
Vitiosangium	0.002±0	0±0	0.006±0.01	0.002±0	0.001±0	0.0395	0.0950	0.001±0	0.001±0	0.004±0.01	0.007±0.01	0.001±0	0.0027	0.0110
Weeksellaceae_X	0±0	0±0	0.002±0	0.001±0	0.001±0	0.6218	0.6800	0.001±0	0.005±0.01	0.003±0.01	0.007±0.02	0.002±0.01	0.6820	0.7200
Weissella	0±0	0±0	0.001±0	0.001±0	0.002±0	0.1788	0.2800	0±0	0±0	0.003±0.01	0.002±0	0.014±0.03	0.1605	0.2500
Williamwhitmania	0.005±0.01	0.023±0.03	0.043±0.04	0.045±0.03	0.022±0.02	0.0077	0.0270	0.144±0.17	0.188±0.24	0.259±0.3	0.242±0.21	0.123±0.13	0.4377	0.5100
Xanthomonadaceae_X	0.001±0	0.018±0.05	0.001±0.01	0.005±0.01	0.007±0.02	0.6111	0.6800	0±0	0±0	0±0	0.005±0.01	0.008±0.02	0.0189	0.0460
Xanthomonadales_X_X	0±0	0±0	0±0	0.004±0.01	0.003±0.01	0.0031	0.0130	0±0	0±0	0±0	0.009±0.01	0.006±0.02	0.0009	0.0051
Xanthomonas_A	0±0	0.007±0.02	0.004±0.01	0.014±0.02	0.007±0.02	0.1566	0.2600	0±0	0±0	0±0	0±0	0±0	NA	NA
Xanthomonas_B	0.015±0.03	0.105±0.09	0.051±0.05	0.031±0.05	0.003±0.01	0.0001	0.0011	0.003±0.01	0.026±0.04	0.019±0.03	0.01±0.02	0.005±0.01	0.0946	0.1600
XBB1006	0±0	0±0	0.001±0	0.002±0	0.003±0.01	0.3562	0.4800	0.001±0	0±0	0.001±0	0.002±0	0.003±0.01	0.4633	0.5300
XYC2-FULL-35-21	0.002±0.01	0±0	0.003±0	0.003±0	0±0	0.0913	0.1800	0.005±0.02	0.001±0	0.006±0.01	0.025±0.08	0.001±0	0.0963	0.1700
YD12-FULL-39-22	0±0	0.008±0.01	0.003±0.01	0.001±0	0.001±0	0.4437	0.5400	0±0	0.002±0	0±0	0.001±0	0±0	0.3497	0.4500
Zag1	1.307±0.39	3.558±1.82	2.49±0.84	2.259±0.56	2.175±1.03	0.0002	0.0012	0.378±0.28	0.437±0.23	0.55±0.12	0.664±0.18	1.05±1.1	0.0025	0.0110
Zag111	0.061±0.05	0.104±0.07	0.108±0.07	0.096±0.05	0.062±0.06	0.1409	0.2400	0.08±0.14	0.078±0.05	0.146±0.1	0.132±0.1	0.213±0.12	0.0013	0.0075

Table S5: Pairwise-comparison of taxa in Collection-1 with other Collections (2 to 5) from samples of liquid fraction. Suffix "_X" in genus name suggests that taxa was assigned at higher taxonomic levels than genus. Multiple suffixes are kept identifying exact level of assignment.

	Collection-2		Collection-3		Collection-4		Collection-5	
Taxa	Wilcoxon test p-value	Wilcoxon test, BH adjusted p-value	Wilcoxon test p-value	Wilcoxon test, BH adjusted p-value	Wilcoxon test p-value	Wilcoxon test, BH adjusted p-value	Wilcoxon test p-value	Wilcoxon test, BH adjusted p-value
**Phylum**								
**Actinobacteriota**	0.0380	0.1300	0.0124	0.0630	0.1761	0.3600	0.1521	0.3300
**Bacteroidota**	0.0003	0.0055	0.0000	0.0007	0.0000	0.0007	0.0045	0.0310
**Bdellovibrionota**	0.1129	0.2700	0.1129	0.2700	0.0485	0.1500	0.2478	0.4600
**Campylobacterota**	0.5015	0.6600	0.0032	0.0250	0.0297	0.1100	0.0429	0.1400
**Chloroflexota**	0.0957	0.2400	0.0145	0.0680	0.0137	0.0680	0.0417	0.1400
**Cyanobacteria**	0.0002	0.0051	0.0001	0.0036	0.0001	0.0029	0.0387	0.1300
**Desulfobacterota**	0.3615	0.5400	0.0470	0.1500	0.1504	0.3300	0.8163	0.9000
**Desulfobacterota_A**	0.0552	0.1700	0.0256	0.1100	0.0687	0.2000	0.1123	0.2700
**Desulfuromonadota**	0.1662	0.3400	0.1217	0.2800	0.5189	0.6700	0.6513	0.7600
**Elusimicrobiota**	0.0002	0.0051	0.1782	0.3600	0.3474	0.5400	0.1135	0.2700
**Fibrobacterota**	0.1432	0.3200	0.1432	0.3200	0.0284	0.1100	0.5899	0.7200
**Firmicutes**	0.3186	0.5200	0.0597	0.1800	0.0018	0.0200	0.0045	0.0310
**Firmicutes_A**	0.0100	0.0540	0.0000	0.0001	0.0000	0.0001	0.0000	0.0001
**Firmicutes_B**	0.2207	0.4200	0.0193	0.0860	0.0531	0.1600	0.2198	0.4200
**Firmicutes_C**	0.0007	0.0100	0.8874	0.9500	0.4776	0.6500	0.0018	0.0200
**Firmicutes_I**	0.1662	0.3400	0.1662	0.3400	0.0367	0.1300	0.0788	0.2100
**Firmicutes_K**	0.0788	0.2100	0.4085	0.5800	0.3740	0.5500	0.3740	0.5500
**Fusobacteriota**	0.4353	0.6000	0.0782	0.2100	0.0885	0.2300	0.3122	0.5200
**Gemmatimonadota**	0.3593	0.5400	0.3593	0.5400	0.0788	0.2100	0.3593	0.5400
**Myxococcota**	0.3397	0.5400	0.4356	0.6000	0.8851	0.9500	0.0124	0.0630
**Patescibacteria**	0.0002	0.0051	0.0100	0.0540	0.0029	0.0240	0.0000	0.0007
**Planctomycetota**	0.1432	0.3200	0.1277	0.2900	0.1782	0.3600	0.7553	0.8600
**Proteobacteria**	0.0068	0.0410	0.0011	0.0140	0.1005	0.2500	0.4095	0.5800
**Riflebacteria**	0.1135	0.2700	0.1432	0.3200	0.0100	0.0540	0.2657	0.4700

**Spirochaetota**	0.5137	0.6600	0.0387	0.1300	0.0284	0.1100	0.0887	0.2300
**Synergistota**	1.0000	1.0000	0.0009	0.0130	0.0020	0.0210	0.0166	0.0760
**Unclassified Phylum**	0.1600	0.3400	0.0005	0.0087	0.0003	0.0055	0.3777	0.5500
**Verrucomicrobiota**	0.8874	0.9500	0.6297	0.7400	0.0684	0.2000	0.3474	0.5400
**Genus**								
**32-67-11**	NA	NA	0.1662	0.3900	0.0165	0.1100	0.1662	0.3900
**4C28d-15_X_X**	0.3226	0.5600	0.0826	0.2700	0.0013	0.0210	1.0000	1.0000
**992a**	0.3355	0.5600	0.4091	0.6100	0.0216	0.1300	0.1955	0.4400
**ABY1_X_X_X**	0.8389	0.9500	0.8389	0.9500	0.8389	0.9500	0.1662	0.3900
**Acetivibrionaceae_X**	0.8257	0.9400	0.0200	0.1200	0.0342	0.1700	0.0046	0.0500
**Acetoanaerobium**	NA	NA	NA	NA	NA	NA	0.1662	0.3900
**Acetobacteraceae_X**	0.0788	0.2600	0.1662	0.3900	0.0367	0.1700	0.1662	0.3900
**Acholeplasma_C**	0.0009	0.0170	0.8428	0.9500	0.0205	0.1200	0.0011	0.0190
**Acholeplasma_D**	0.2593	0.5300	0.0033	0.0390	0.0001	0.0054	0.0005	0.0110
**Acholeplasmatales_X_X**	0.0091	0.0770	0.2345	0.5000	0.9770	1.0000	0.0102	0.0820
**Achromobacter**	0.5807	0.7600	0.0809	0.2700	0.0087	0.0750	0.5807	0.7600
**Acidovorax_E**	0.3593	0.5600	0.0788	0.2600	0.0165	0.1100	0.1662	0.3900
**Acinetobacter**	0.8199	0.9300	0.1535	0.3900	0.0080	0.0690	0.3021	0.5600
**Actinobacillus**	0.2854	0.5600	0.2189	0.4800	0.0684	0.2600	0.5899	0.7600
**Actinobacillus_A**	0.5189	0.7000	0.8097	0.9200	0.7052	0.8400	0.7569	0.8800
**Actinomyces**	NA	NA	0.0788	0.2600	NA	NA	0.1662	0.3900
**Actinomycetaceae_X**	0.8939	0.9800	0.8939	0.9800	0.5420	0.7300	0.0585	0.2300
**Actinomycetales_X_X**	0.3593	0.5600	0.1662	0.3900	NA	NA	0.3593	0.5600
**Acutalibacteraceae_X**	0.2243	0.4800	0.2446	0.5100	0.0101	0.0820	0.1920	0.4400
**Aerococcaceae_X**	0.6513	0.8000	0.2652	0.5400	0.4709	0.6700	0.6548	0.8000
**Aerococcus**	NA	NA	NA	NA	NA	NA	0.1662	0.3900
**Agarilytica**	NA	NA	0.1662	0.3900	0.0029	0.0360	0.1662	0.3900
**Agitococcus**	0.0802	0.2700	0.1265	0.3600	0.9248	1.0000	0.1336	0.3800
**Akkermansia**	0.1662	0.3900	0.0165	0.1100	0.1662	0.3900	0.3593	0.5600
**Alcanivorax_A**	0.0000	0.0039	0.0001	0.0048	0.1071	0.3200	0.3712	0.5700

**Algoriphagus**	NA	NA	NA	NA	0.0071	0.0620	NA	NA
**Aliarcobacter**	NA	NA	0.0029	0.0360	NA	NA	0.3593	0.5600
**Alishewanella**	0.0000	0.0039	0.0000	0.0018	0.0000	0.0018	0.0004	0.0096
**Alphaproteobacteria_X_X_X**	0.0007	0.0140	0.0780	0.2600	0.0068	0.0620	0.0684	0.2600
**Anaerofilum**	0.4453	0.6400	0.0039	0.0450	0.0011	0.0190	0.2087	0.4600
**Anaerofustis**	0.1662	0.3900	0.5139	0.7000	0.5139	0.7000	0.1662	0.3900
**Anaerorhabdus**	0.3593	0.5600	0.1662	0.3900	0.3593	0.5600	0.1662	0.3900
**Anaerosporobacter**	0.3593	0.5600	0.3593	0.5600	NA	NA	NA	NA
**Anaerotignum**	NA	NA	0.1662	0.3900	NA	NA	0.0788	0.2600
**Anaerovibrio**	0.8939	0.9800	0.0154	0.1100	0.0015	0.0220	0.2394	0.5100
**Anaerovoracaceae_X**	0.1391	0.3900	0.5292	0.7100	0.3956	0.5900	0.3035	0.5600
**Aquabacterium**	0.0011	0.0190	0.0004	0.0096	0.0029	0.0360	0.1662	0.3900
**Arabia**	NA	NA	NA	NA	NA	NA	0.3593	0.5600
**Arenimonas**	0.0002	0.0074	0.0002	0.0074	0.0195	0.1200	0.3593	0.5600
**Aromatoleum**	0.3593	0.5600	0.3593	0.5600	0.1662	0.3900	0.0071	0.0620
**Asticcacaulis**	NA	NA	NA	NA	0.0788	0.2600	NA	NA
**Bacilli_X_X_X**	0.1135	0.3300	0.1277	0.3600	0.0014	0.0220	0.2986	0.5600
**Bacillus**	0.3593	0.5600	0.3593	0.5600	0.3593	0.5600	0.3593	0.5600
**Bacillus_AD**	0.3593	0.5600	1.0000	1.0000	0.3593	0.5600	0.3593	0.5600
**Bacillus_W**	NA	NA	0.0165	0.1100	NA	NA	NA	NA
**Bact-08**	0.5058	0.7000	0.9310	1.0000	0.0463	0.2000	0.4701	0.6700
**Bacteria_X_X_X_X_X**	0.1600	0.3900	0.0005	0.0120	0.0003	0.0081	0.3777	0.5700
**Bacteroidaceae_X**	0.0242	0.1400	0.0332	0.1700	0.0173	0.1100	0.0000	0.0018
**Bacteroidales_X_X**	0.4095	0.6100	0.3777	0.5700	0.8874	0.9800	0.0449	0.1900
**Bacteroides**	0.7506	0.8800	0.9079	0.9900	0.8397	0.9500	0.8392	0.9500
**Bacteroidia_X_X_X**	0.1135	0.3300	0.0332	0.1700	0.0449	0.1900	0.0121	0.0920
**Bdellovibrio**	0.3593	0.5600	0.3593	0.5600	0.0367	0.1700	0.1662	0.3900
**Beijerinckiaceae_X**	0.3593	0.5600	1.0000	1.0000	0.3593	0.5600	0.3593	0.5600
**Bibersteinia**	0.0193	0.1200	0.0102	0.0820	0.2854	0.5600	0.6233	0.7900
**Blastomonas**	0.1662	0.3900	0.0367	0.1700	0.0367	0.1700	0.0788	0.2600

**Blautia_A**	0.3593	0.5600	0.1662	0.3900	0.0788	0.2600	0.3593	0.5600
**Bosea**	0.3593	0.5600	0.0071	0.0620	0.3593	0.5600	NA	NA
**Brachymonas**	0.3281	0.5600	0.6513	0.8000	0.1798	0.4100	1.0000	1.0000
**Brevundimonas**	0.0027	0.0360	0.0005	0.0120	0.0163	0.1100	0.4915	0.6900
**BRH-c57**	0.1662	0.3900	0.1662	0.3900	NA	NA	0.0367	0.1700
**Burkholderiaceae_X**	0.3404	0.5600	0.5440	0.7300	0.7074	0.8400	0.2834	0.5600
**Butyrivibrio**	0.0729	0.2600	0.0000	0.0042	0.0000	0.0042	0.0000	0.0039
**C941**	0.0261	0.1400	0.0001	0.0054	0.0002	0.0068	0.0038	0.0440
**CAG-180**	0.0788	0.2600	0.0165	0.1100	0.0788	0.2600	0.0788	0.2600
**CAG-312**	0.1662	0.3900	0.0011	0.0190	0.0165	0.1100	0.0367	0.1700
**CAG-313**	0.7506	0.8800	0.0687	0.2600	0.1745	0.4100	0.3854	0.5800
**CAG-354**	0.0597	0.2300	0.0036	0.0420	0.0007	0.0140	0.0100	0.0810
**CAG-462**	0.0029	0.0360	0.1005	0.3100	0.0242	0.1400	0.0011	0.0190
**CAG-465**	0.7569	0.8800	0.0367	0.1700	0.8097	0.9200	0.4453	0.6400
**CAG-475**	0.1951	0.4400	0.0836	0.2700	0.0099	0.0810	0.4783	0.6800
**CAG-495**	0.0145	0.1000	0.0284	0.1500	0.0242	0.1400	0.0887	0.2800
**CAG-508_X**	0.3593	0.5600	NA	NA	NA	NA	0.3593	0.5600
**CAG-826_X**	0.7553	0.8800	0.0242	0.1400	0.0145	0.1000	0.2415	0.5100
**CAG-873**	NA	NA	0.3593	0.5600	0.0788	0.2600	0.1662	0.3900
**CAG-878**	0.0225	0.1300	0.0462	0.2000	0.0043	0.0480	0.1257	0.3600
**Campylobacter**	0.4709	0.6700	0.8014	0.9200	0.2198	0.4800	0.0449	0.1900
**Campylobacter_A**	0.2089	0.4600	0.0313	0.1600	0.0871	0.2800	0.4953	0.6900
**Campylobacter_B**	0.3593	0.5600	0.3593	0.5600	0.1662	0.3900	0.3593	0.5600
**Campylobacteraceae_X**	1.0000	1.0000	0.2860	0.5600	0.1129	0.3300	1.0000	1.0000
**Capnocytophaga**	NA	NA	NA	NA	NA	NA	0.3593	0.5600
**Caryophanon**	0.3593	0.5600	0.3593	0.5600	0.3593	0.5600	0.1662	0.3900
**Caviibacter**	0.0788	0.2600	0.2734	0.5500	0.9395	1.0000	0.6548	0.8000
**Cellvibrio**	0.0001	0.0054	0.0000	0.0018	0.0001	0.0054	0.0788	0.2600
**Cellvibrionaceae_X**	0.3593	0.5600	0.0071	0.0620	0.0071	0.0620	0.3593	0.5600
**Chishuiella**	0.0367	0.1700	0.0029	0.0360	0.0367	0.1700	0.0788	0.2600

**CHKCI001**	0.3593	0.5600	0.3593	0.5600	0.1662	0.3900	NA	NA
**Christensenellales_X_X**	0.3740	0.5700	0.2860	0.5600	0.1912	0.4400	0.0788	0.2600
**Chryseobacterium**	0.0367	0.1700	0.0071	0.0620	0.0011	0.0190	0.0788	0.2600
**Chryseobacterium_B**	NA	NA	NA	NA	0.1662	0.3900	0.1662	0.3900
**Chryseobacterium_D**	0.3593	0.5600	1.0000	1.0000	0.5807	0.7600	0.3740	0.5700
**Cloacibacterium**	0.4591	0.6600	0.0437	0.1900	0.2994	0.5600	0.4973	0.7000
**Clostridia_X_X_X**	0.1432	0.3900	0.0007	0.0140	0.0000	0.0042	0.0121	0.0920
**Clostridium**	0.6548	0.8000	0.5358	0.7200	0.9395	1.0000	0.4504	0.6500
**Clostridium_A**	0.3593	0.5600	0.3593	0.5600	0.3593	0.5600	1.0000	1.0000
**Clostridium_L**	0.5139	0.7000	0.2860	0.5600	0.3593	0.5600	0.3593	0.5600
**Clostridium_M**	0.3593	0.5600	0.3593	0.5600	0.3593	0.5600	0.0195	0.1200
**Clostridium_N**	0.1955	0.4400	0.2734	0.5500	0.9263	1.0000	0.0910	0.2900
**Comamonas**	1.0000	1.0000	0.1298	0.3700	0.6059	0.7700	1.0000	1.0000
**Corynebacterium**	NA	NA	NA	NA	0.3593	0.5600	NA	NA
**CP2B**	0.3593	0.5600	NA	NA	0.3593	0.5600	NA	NA
**Cytophagales_X_X**	1.0000	1.0000	0.0481	0.2000	0.6513	0.8000	0.1548	0.3900
**Denitrobacterium**	NA	NA	0.3593	0.5600	0.3593	0.5600	NA	NA
**Dermatophilaceae_X**	0.6513	0.8000	0.9645	1.0000	0.9645	1.0000	0.5420	0.7300
**Desulfobacterota_X_X_X_X**	0.3615	0.5700	0.0470	0.2000	0.1504	0.3900	0.8163	0.9300
**Desulfovibrio**	0.3593	0.5600	NA	NA	NA	NA	0.3593	0.5600
**Desulfovibrionaceae_X**	0.5972	0.7600	0.1039	0.3200	0.0014	0.0220	0.5972	0.7600
**Devosia**	0.0165	0.1100	0.0367	0.1700	0.0788	0.2600	0.0367	0.1700
**Dialister_A**	0.3593	0.5600	0.0788	0.2600	0.3593	0.5600	0.3593	0.5600
**Dichelobacter**	0.7062	0.8400	0.0604	0.2300	0.4179	0.6200	0.7711	0.8900
**Dietzia**	0.0788	0.2600	NA	NA	NA	NA	NA	NA
**Dongia**	0.0011	0.0190	0.0029	0.0360	0.0001	0.0054	0.0029	0.0360
**DTU053**	0.9395	1.0000	0.0090	0.0770	0.0055	0.0570	0.2422	0.5100
**Duncaniella**	0.0788	0.2600	0.1662	0.3900	0.3593	0.5600	NA	NA
**Dysgonomonadaceae_X**	NA	NA	0.3593	0.5600	0.0165	0.1100	0.0788	0.2600
**Echinicola**	0.0001	0.0054	0.0000	0.0039	0.0029	0.0360	0.0165	0.1100

**Eggerthellaceae_X**	1.0000	1.0000	0.3593	0.5600	0.1129	0.3300	1.0000	1.0000
**Elizabethkingia**	0.1662	0.3900	0.3593	0.5600	0.0367	0.1700	0.0788	0.2600
**Emergencia**	0.3593	0.5600	NA	NA	0.3593	0.5600	0.3593	0.5600
**Empedobacter**	0.0367	0.1700	0.0071	0.0620	0.0004	0.0096	0.0788	0.2600
**Endomicrobium**	0.7832	0.9000	0.7150	0.8400	0.5283	0.7100	0.4091	0.6100
**Endomicrobium_A**	0.1005	0.3100	0.0009	0.0170	0.0009	0.0170	0.0166	0.1100
**Enterobacterales_X_X**	0.0261	0.1400	0.7125	0.8400	0.3186	0.5600	0.6297	0.7900
**Enterobacteriaceae_X**	0.0045	0.0500	0.0449	0.1900	0.0068	0.0620	0.0014	0.0220
**Enterococcaceae_X**	0.2478	0.5100	0.1129	0.3300	0.1129	0.3300	0.3593	0.5600
**Enterococcus**	0.0788	0.2600	0.0367	0.1700	0.0004	0.0096	0.0165	0.1100
**Erysipelatoclostridiaceae_X**	NA	NA	0.3593	0.5600	0.3593	0.5600	NA	NA
**Erysipelothrix**	0.1123	0.3300	0.0153	0.1100	0.0002	0.0066	0.0598	0.2300
**Erysipelotrichaceae_X**	0.0568	0.2300	0.3467	0.5600	0.7813	0.9000	0.3702	0.5700
**Erysipelotrichales_X_X**	1.0000	1.0000	1.0000	1.0000	0.3740	0.5700	0.6513	0.8000
**Eubacterium_C**	0.7145	0.8400	0.0788	0.2600	0.6404	0.8000	0.2710	0.5500
**Eubacterium_E**	0.0012	0.0200	0.0001	0.0054	0.0001	0.0054	0.0015	0.0220
**Eubacterium_F**	0.6033	0.7700	0.1277	0.3600	0.0068	0.0620	0.3864	0.5800
**Eubacterium_Q**	0.0059	0.0600	0.0776	0.2600	0.0117	0.0910	0.0066	0.0620
**Eubacterium_S**	0.0033	0.0390	0.0243	0.1400	0.0733	0.2600	0.0012	0.0200
**F0040**	0.3777	0.5700	0.4095	0.6100	0.0145	0.1000	0.1135	0.3300
**F0058**	0.3428	0.5600	0.8939	0.9800	0.3828	0.5800	0.9645	1.0000
**F0422**	1.0000	1.0000	0.1504	0.3900	0.9749	1.0000	0.8467	0.9500
**F0428**	0.0139	0.1000	0.0228	0.1300	0.0002	0.0069	0.0420	0.1900
**F082**	0.9774	1.0000	0.5899	0.7600	0.0284	0.1500	0.8874	0.9800
**FD2005**	0.0002	0.0058	0.0001	0.0047	0.0001	0.0047	0.0001	0.0054
**Fermentimonas**	0.8389	0.9500	0.5420	0.7300	0.8939	0.9800	0.7145	0.8400
**Fibrobacter**	0.1600	0.3900	0.1277	0.3600	0.0284	0.1500	0.4428	0.6400
**Fibrobacter_A**	0.3777	0.5700	0.7290	0.8600	0.2726	0.5500	0.0009	0.0170
**Firm-04**	0.0685	0.2600	0.0641	0.2500	0.8467	0.9500	0.7832	0.9000
**Firm-16**	0.4067	0.6100	0.5420	0.7300	0.6798	0.8200	0.8794	0.9800

**Firmicutes_A_X_X_X_X**	0.1548	0.3900	0.4085	0.6100	0.6548	0.8000	0.5898	0.7600
**Firmicutes_B_X_X_X_X**	0.2207	0.4800	0.0193	0.1200	0.0531	0.2200	0.2198	0.4800
**Flavobacteriaceae_X**	0.4776	0.6800	0.3474	0.5600	0.6707	0.8100	0.9323	1.0000
**Flavobacteriales_X_X**	0.7072	0.8400	0.7506	0.8800	0.2851	0.5600	0.1649	0.3900
**Flavobacterium**	0.0001	0.0054	0.0000	0.0018	0.0000	0.0039	0.0004	0.0096
**Flavobacterium_A**	0.0031	0.0380	0.0001	0.0047	0.0002	0.0074	0.0073	0.0630
**Flexilinea**	0.0957	0.3000	0.0145	0.1000	0.0137	0.1000	0.0417	0.1900
**Fodinicurvata**	0.1798	0.4100	0.8939	0.9800	0.5139	0.7000	0.1662	0.3900
**Frateuria**	0.3593	0.5600	0.1662	0.3900	0.1662	0.3900	0.0788	0.2600
**Fusicatenibacter**	0.1761	0.4100	0.0069	0.0620	0.0003	0.0081	0.0020	0.0290
**Fusobacterium**	NA	NA	0.3593	0.5600	0.1662	0.3900	NA	NA
**Fusobacterium_A**	0.2582	0.5300	0.2834	0.5600	0.9309	1.0000	0.8160	0.9300
**Fusobacterium_C**	0.2582	0.5300	0.0151	0.1100	0.0029	0.0360	0.0193	0.1200
**Gammaproteobacteria_X_X_X**	0.0009	0.0170	0.4776	0.6800	0.0242	0.1400	0.0056	0.0570
**Gastranaerophilaceae_X**	0.3358	0.5600	0.8014	0.9200	0.1086	0.3300	0.7464	0.8800
**GCA-2733575**	NA	NA	0.0788	0.2600	0.3593	0.5600	NA	NA
**GCA-900066495**	1.0000	1.0000	0.5807	0.7600	0.2478	0.5100	1.0000	1.0000
**GCA-900066905**	0.0485	0.2000	0.1326	0.3700	0.0008	0.0160	0.2478	0.5100
**GCA-900066995**	0.9291	1.0000	0.6611	0.8000	0.2844	0.5600	0.3849	0.5800
**Gemella_A**	0.3593	0.5600	0.0788	0.2600	0.0011	0.0190	0.1662	0.3900
**Gemmatimonadaceae_X**	0.3593	0.5600	0.3593	0.5600	NA	NA	NA	NA
**Gemmatimonas**	NA	NA	NA	NA	0.0788	0.2600	0.3593	0.5600
**Gemmobacter_A**	0.3593	0.5600	0.0367	0.1700	0.3593	0.5600	0.0029	0.0360
**Geothermobacter**	0.1662	0.3900	0.1217	0.3500	0.5189	0.7000	0.6513	0.8000
**Gluconobacter**	0.3593	0.5600	0.0788	0.2600	0.0367	0.1700	0.3593	0.5600
**Glutamicibacter**	NA	NA	0.1662	0.3900	0.3593	0.5600	0.1662	0.3900
**GN02-873**	0.2194	0.4800	0.0255	0.1400	0.5898	0.7600	0.5821	0.7600
**Gottschalkiaceae_X**	0.1662	0.3900	0.5807	0.7600	0.5807	0.7600	1.0000	1.0000
**GWE2-31-10**	0.7577	0.8800	0.5372	0.7200	0.6370	0.8000	0.9749	1.0000
**GWF2-44-16**	0.0685	0.2600	0.0073	0.0630	0.5807	0.7600	0.2478	0.5100

**Halomonas_A**	NA	NA	0.0165	0.1100	0.3593	0.5600	NA	NA
**Helcococcaceae_X**	NA	NA	NA	NA	NA	NA	0.3593	0.5600
**Helcococcus**	0.5807	0.7600	0.0133	0.1000	0.0809	0.2700	1.0000	1.0000
**Hepatobacter**	0.0788	0.2600	0.0004	0.0096	0.0001	0.0054	0.0029	0.0360
**Humitalea**	0.3593	0.5600	0.3740	0.5700	0.3593	0.5600	0.3593	0.5600
**Hungatella_A**	0.0029	0.0360	0.0000	0.0005	0.0000	0.0005	0.0000	0.0008
**Hyphomonas**	NA	NA	0.1662	0.3900	0.0367	0.1700	0.0788	0.2600
**Inquilinus**	0.3593	0.5600	0.3593	0.5600	1.0000	1.0000	0.3593	0.5600
**Kapabacteriaceae_X**	0.5972	0.7600	0.7145	0.8400	0.2478	0.5100	0.3281	0.5600
**Kingella**	0.1262	0.3600	0.0005	0.0120	0.0066	0.0620	0.0049	0.0530
**Kiritimatiellae_X_X_X**	0.5512	0.7300	0.7125	0.8400	0.3474	0.5600	0.7553	0.8800
**KLE1796**	NA	NA	0.3593	0.5600	0.3593	0.5600	NA	NA
**Kocuria**	0.3593	0.5600	0.3593	0.5600	0.3593	0.5600	0.3593	0.5600
**Lachnoanaerobaculum**	0.2478	0.5100	0.0008	0.0160	0.0008	0.0160	0.0485	0.2000
**Lachnospira**	1.0000	1.0000	0.2478	0.5100	0.0002	0.0074	0.0073	0.0630
**Lachnospiraceae_X**	0.0009	0.0170	0.0000	0.0005	0.0000	0.0005	0.0000	0.0005
**Lachnospirales_X_X**	0.7072	0.8400	0.1841	0.4200	0.1978	0.4400	0.0684	0.2600
**Lactobacillales_X_X**	0.3593	0.5600	1.0000	1.0000	0.2478	0.5100	1.0000	1.0000
**Lactobacillus_F**	0.3593	0.5600	0.3593	0.5600	0.3593	0.5600	1.0000	1.0000
**Lactococcus**	0.0367	0.1700	0.0165	0.1100	0.0165	0.1100	0.0788	0.2600
**Lactonifactor**	NA	NA	0.1662	0.3900	0.1662	0.3900	0.3593	0.5600
**Lancefieldella**	NA	NA	0.3593	0.5600	NA	NA	NA	NA
**Lawsonibacter**	0.8939	0.9800	0.8389	0.9500	0.1662	0.3900	0.5139	0.7000
**Leaf454**	0.0483	0.2000	0.0016	0.0240	0.0001	0.0054	0.0008	0.0160
**Lenti-01**	0.0349	0.1700	0.0007	0.0150	0.0007	0.0150	0.0530	0.2200
**Lentimicrobium**	0.8874	0.9800	0.4776	0.6800	1.0000	1.0000	0.1005	0.3100
**Lentisphaeria_X_X_X**	0.0788	0.2600	0.3593	0.5600	0.1662	0.3900	0.3593	0.5600
**Leptotrichiaceae_X**	0.3593	0.5600	1.0000	1.0000	0.3740	0.5700	0.3593	0.5600
**Leuconostoc**	0.1662	0.3900	0.0071	0.0620	0.0029	0.0360	0.0367	0.1700
**Lysobacter**	0.3593	0.5600	1.0000	1.0000	0.3593	0.5600	0.3593	0.5600

**Mailhella**	0.0267	0.1500	0.0521	0.2100	0.2345	0.5000	0.0362	0.1700
**Mannheimia**	0.2668	0.5400	0.4693	0.6700	0.6370	0.8000	0.6061	0.7700
**Marinilabiliaceae_X**	0.3593	0.5600	0.5807	0.7600	0.2076	0.4600	0.3593	0.5600
**Marseille-P3160**	0.3593	0.5600	1.0000	1.0000	0.3593	0.5600	0.5139	0.7000
**Massilia_B**	NA	NA	0.0004	0.0096	NA	NA	NA	NA
**Massilibacteroides**	0.6707	0.8100	0.4776	0.6800	0.0068	0.0620	0.0597	0.2300
**Metamycoplasma**	0.5139	0.7000	0.5139	0.7000	1.0000	1.0000	1.0000	1.0000
**Methylobacterium**	0.3593	0.5600	0.3593	0.5600	0.3593	0.5600	NA	NA
**Micavibrionaceae_X**	0.3593	0.5600	NA	NA	0.3593	0.5600	0.0165	0.1100
**Micrococcaceae_X**	0.3593	0.5600	0.3593	0.5600	0.3593	0.5600	NA	NA
**Monoglobales_X_X**	1.0000	1.0000	0.5139	0.7000	0.2478	0.5100	0.2478	0.5100
**Monoglobus**	0.0526	0.2200	0.0400	0.1800	0.0600	0.2300	0.4671	0.6700
**Moraxella**	0.2913	0.5600	0.0684	0.2600	0.2189	0.4800	0.6297	0.7900
**Moraxella_A**	0.1662	0.3900	0.5139	0.7000	0.1662	0.3900	0.8939	0.9800
**Moraxellaceae_X**	0.4021	0.6000	0.1649	0.3900	0.5245	0.7100	0.0228	0.1300
**Mucilaginibacter**	0.0788	0.2600	0.1662	0.3900	0.1662	0.3900	0.0367	0.1700
**Muribaculaceae_X**	0.3086	0.5600	0.0003	0.0086	0.0009	0.0170	0.0117	0.0910
**Myroides**	0.3593	0.5600	0.3593	0.5600	0.3593	0.5600	0.1662	0.3900
**Ndongobacter**	NA	NA	0.3593	0.5600	0.1662	0.3900	NA	NA
**Negativicutes_X_X_X**	0.4528	0.6500	0.0242	0.1400	0.0121	0.0920	0.1935	0.4400
**Neisseria_B**	0.3281	0.5600	0.0809	0.2700	0.1326	0.3700	0.1798	0.4100
**Neisseria_G**	0.3593	0.5600	0.3593	0.5600	1.0000	1.0000	1.0000	1.0000
**Neisseriaceae_X**	0.5426	0.7300	0.2592	0.5300	0.1257	0.3600	0.8851	0.9800
**Neorhizobium**	0.3740	0.5700	0.0568	0.2300	0.2076	0.4600	0.3740	0.5700
**Niveispirillum**	0.1662	0.3900	NA	NA	0.0367	0.1700	0.0029	0.0360
**NS-102**	0.0367	0.1700	0.3593	0.5600	0.0788	0.2600	0.1662	0.3900
**OEMR01**	0.6513	0.8000	1.0000	1.0000	0.3740	0.5700	1.0000	1.0000
**Olegusella**	NA	NA	NA	NA	0.3593	0.5600	NA	NA
**Olsenella**	0.0216	0.1300	0.2710	0.5500	0.1353	0.3800	0.9395	1.0000
**Opitutaceae_X**	0.5512	0.7300	0.3474	0.5600	0.0205	0.1200	0.9323	1.0000

**Opitutales_X_X**	0.6513	0.8000	0.6513	0.8000	1.0000	1.0000	0.3593	0.5600
**Opitutus**	NA	NA	0.0165	0.1100	0.0165	0.1100	NA	NA
**Oribacterium**	0.1406	0.3900	0.0018	0.0270	0.0001	0.0053	0.0050	0.0540
**Orrella**	0.0108	0.0860	0.7760	0.9000	0.8939	0.9800	0.8939	0.9800
**Oscillibacter**	0.3355	0.5600	0.1955	0.4400	0.0228	0.1300	0.5898	0.7600
**Oscillospiraceae_X**	0.3777	0.5700	0.3777	0.5700	0.0145	0.1000	0.0056	0.0570
**Oscillospirales_X_X**	0.3186	0.5600	0.0001	0.0047	0.0000	0.0018	0.0284	0.1500
**Paenibacillus_J**	0.3593	0.5600	NA	NA	0.1662	0.3900	0.0788	0.2600
**PALSA-1355**	0.5412	0.7300	0.5412	0.7300	1.0000	1.0000	0.6404	0.8000
**Paludibacteraceae_X**	0.7553	0.8800	0.2913	0.5600	0.4095	0.6100	0.0462	0.2000
**Paramesorhizobium**	0.0071	0.0620	0.1662	0.3900	0.0367	0.1700	0.1662	0.3900
**Paramuribaculum**	0.8389	0.9500	1.0000	1.0000	0.8199	0.9300	0.6548	0.8000
**Parapedobacter**	0.1662	0.3900	0.0367	0.1700	0.0165	0.1100	0.1662	0.3900
**Paraprevotella**	0.5443	0.7300	0.4704	0.6700	0.6235	0.7900	0.5443	0.7300
**Parvibaculum**	0.0002	0.0075	0.0001	0.0047	0.0001	0.0057	0.0051	0.0540
**Parvimonas**	0.2422	0.5100	0.3712	0.5700	0.0711	0.2600	0.2860	0.5600
**Pasteurellaceae_X**	0.4095	0.6100	0.0029	0.0360	0.0387	0.1700	0.4776	0.6800
**Pedosphaeraceae_X**	0.0045	0.0500	0.0068	0.0620	0.0023	0.0320	0.0068	0.0620
**PeH17**	0.0887	0.2800	0.0100	0.0810	0.0002	0.0069	0.5830	0.7600
**Peptostreptococcaceae_X**	0.1662	0.3900	0.3593	0.5600	0.0165	0.1100	0.3593	0.5600
**Peptostreptococcales_X_X**	0.2089	0.4600	0.0395	0.1800	0.1379	0.3900	1.0000	1.0000
**Phycorickettsia**	1.0000	1.0000	1.0000	1.0000	0.2478	0.5100	0.1326	0.3700
**Pigmentiphaga**	0.0343	0.1700	0.0549	0.2200	0.0474	0.2000	0.0031	0.0380
**Pirellulaceae_X**	NA	NA	0.1662	0.3900	0.3593	0.5600	NA	NA
**Pirellulales_X_X**	0.2913	0.5600	0.0003	0.0081	0.0011	0.0190	0.0332	0.1700
**Planctomycetes_X_X_X**	0.9735	1.0000	0.8574	0.9600	0.2198	0.4800	0.9178	1.0000
**Planctomycetota_X_X_X_X**	0.0031	0.0370	0.0295	0.1600	0.0360	0.1700	0.0140	0.1000
**Porphyromonas**	0.4095	0.6100	0.2657	0.5400	0.9323	1.0000	0.6707	0.8100
**Prevotella**	0.0000	0.0022	0.0000	0.0005	0.0000	0.0042	0.1005	0.3100
**Prevotellamassilia**	0.1432	0.3900	0.1005	0.3100	0.0387	0.1700	0.0597	0.2300

**Prolixibacteraceae_X**	0.2913	0.5600	0.4428	0.6400	0.3777	0.5700	0.8874	0.9800
**Prosthecobacter**	0.3593	0.5600	0.3593	0.5600	0.0011	0.0190	0.0071	0.0620
**Proteiniclasticum**	0.1662	0.3900	0.0001	0.0054	0.0004	0.0096	0.0011	0.0190
**Proteobacteria_X_X_X_X**	0.0011	0.0190	0.1782	0.4100	1.0000	1.0000	0.0121	0.0920
**Pseudaminobacter**	0.3593	0.5600	0.0788	0.2600	0.1662	0.3900	0.3593	0.5600
**Pseudomonadaceae_X**	0.0018	0.0270	0.0002	0.0067	0.0046	0.0500	0.6513	0.8000
**Pseudomonadales_X_X**	0.3281	0.5600	0.0405	0.1800	0.0048	0.0520	0.6513	0.8000
**Pseudomonas_A**	0.0033	0.0390	0.0004	0.0100	0.0244	0.1400	0.2365	0.5000
**Pseudomonas_B**	0.8635	0.9600	0.5283	0.7100	0.8574	0.9600	0.0367	0.1700
**Pseudomonas_D**	0.0011	0.0190	0.0000	0.0018	0.0367	0.1700	0.1662	0.3900
**Pseudomonas_E**	0.7832	0.9000	0.0841	0.2700	0.1087	0.3300	1.0000	1.0000
**Pseudomonas_F**	0.0165	0.1100	NA	NA	NA	NA	NA	NA
**Pseudomonas_M**	0.2394	0.5100	0.0001	0.0053	0.0023	0.0320	0.0154	0.1100
**Pygmaiobacter**	0.8389	0.9500	0.0031	0.0370	0.0350	0.1700	0.5412	0.7300
**Pyramidobacter**	1.0000	1.0000	0.0007	0.0150	0.0042	0.0480	0.0141	0.1000
**RC9**	0.0001	0.0057	0.0007	0.0140	0.0887	0.2800	0.0242	0.1400
**RF16**	0.4776	0.6800	0.1782	0.4100	0.1432	0.3900	0.4776	0.6800
**Rhizobiaceae_X**	1.0000	1.0000	0.2076	0.4600	0.3740	0.5700	1.0000	1.0000
**Rhizobiales_X_X**	0.9713	1.0000	0.0449	0.1900	0.0139	0.1000	0.9141	0.9900
**Rhodospirillaceae_X**	0.0011	0.0190	0.0011	0.0190	0.0165	0.1100	0.0367	0.1700
**Riemerella**	NA	NA	0.0367	0.1700	0.0788	0.2600	NA	NA
**Roseomonas**	0.0788	0.2600	0.0071	0.0620	0.0165	0.1100	0.0788	0.2600
**Rothia**	0.0367	0.1700	0.0788	0.2600	0.1662	0.3900	NA	NA
**RUG131**	0.2189	0.4800	0.7553	0.8800	0.2913	0.5600	0.4776	0.6800
**RUG163**	0.9774	1.0000	0.4428	0.6400	0.0007	0.0140	0.7987	0.9200
**RUG350**	0.0173	0.1100	0.3474	0.5600	0.9323	1.0000	0.5899	0.7600
**Ruminiclostridium**	0.0991	0.3100	0.0000	0.0042	0.0000	0.0039	0.0002	0.0059
**Ruminiclostridium_C**	0.0887	0.2800	0.0001	0.0057	0.0000	0.0018	0.0009	0.0170
**Ruminiclostridium_D**	0.3226	0.5600	0.0119	0.0920	0.0529	0.2200	0.4102	0.6100
**Ruminiclostridium_F**	0.0499	0.2100	0.6513	0.8000	0.4896	0.6900	0.4896	0.6900

**Ruminococcaceae_X**	0.1257	0.3600	0.2834	0.5600	0.0051	0.0540	0.0046	0.0500
**Ruminococcus**	1.0000	1.0000	0.1262	0.3600	0.0034	0.0400	0.0362	0.1700
**Ruminococcus_C**	0.0066	0.0620	0.0216	0.1300	0.0002	0.0069	0.0001	0.0054
**Ruminococcus_D**	0.2415	0.5100	0.0000	0.0039	0.0000	0.0018	0.0000	0.0042
**Ruminococcus_F**	0.0311	0.1600	0.0070	0.0620	0.0001	0.0048	0.0005	0.0120
**Saccharibacillus**	0.3593	0.5600	0.1662	0.3900	0.0788	0.2600	0.1662	0.3900
**Saccharicrinis**	0.1662	0.3900	0.0071	0.0620	0.0071	0.0620	0.0165	0.1100
**Saccharimonadaceae_X**	0.7068	0.8400	0.0262	0.1400	0.3407	0.5600	0.2844	0.5600
**Saccharimonadales_X_X**	0.1719	0.4000	0.0539	0.2200	0.0618	0.2400	0.6370	0.8000
**Saccharofermentans**	0.0005	0.0120	0.0000	0.0039	0.0000	0.0018	0.0003	0.0081
**Schwartzia**	0.0597	0.2300	0.4095	0.6100	0.4095	0.6100	0.3474	0.5600
**Selenomonadaceae_X**	0.1809	0.4100	0.0023	0.0320	0.0161	0.1100	0.2446	0.5100
**Selenomonas_A**	NA	NA	0.3593	0.5600	0.0071	0.0620	0.3593	0.5600
**Selenomonas_B**	0.1129	0.3300	0.0001	0.0047	0.0002	0.0074	0.1129	0.3300
**Serratia**	0.1662	0.3900	0.0367	0.1700	0.0165	0.1100	0.0788	0.2600
**Shinella**	0.3593	0.5600	0.1662	0.3900	0.1662	0.3900	0.1662	0.3900
**Simonsiella**	0.0585	0.2300	0.4406	0.6400	0.4780	0.6800	0.5726	0.7500
**Sneathia**	0.1662	0.3900	0.0367	0.1700	0.0788	0.2600	NA	NA
**Soleaferrea**	0.0539	0.2200	0.0006	0.0140	0.0001	0.0054	0.0052	0.0550
**Sphaerochaeta**	1.0000	1.0000	0.0002	0.0059	0.0010	0.0180	0.0096	0.0800
**Sphaerochaeta_A**	0.1863	0.4300	0.0001	0.0047	0.0001	0.0047	0.0064	0.0620
**Sphaerochaetaceae_X**	0.2039	0.4600	0.0000	0.0018	0.0000	0.0018	0.0023	0.0320
**Sphingobacterium**	0.0025	0.0340	0.0000	0.0022	0.0001	0.0047	0.0073	0.0630
**Sphingomonas**	0.0011	0.0190	0.0071	0.0620	0.0001	0.0054	0.0071	0.0620
**Sphingopyxis_A**	0.4709	0.6700	0.8635	0.9600	0.1798	0.4100	0.0367	0.1700
**Spirochaetia_X_X_X**	0.9774	1.0000	0.6297	0.7900	0.3186	0.5600	0.4776	0.6800
**Spirochaetota_X_X_X_X**	0.3580	0.5600	0.8532	0.9600	0.1761	0.4100	0.5897	0.7600
**Stenotrophomonas**	0.0000	0.0037	0.0000	0.0037	0.0002	0.0060	0.0148	0.1100
**Streptococcus**	0.6142	0.7800	0.0669	0.2600	0.0678	0.2600	0.5049	0.7000
**Succiniclasticum**	0.0001	0.0057	0.4428	0.6400	0.0519	0.2100	0.0000	0.0022

**Succinimonas**	0.0001	0.0054	0.0205	0.1200	0.1277	0.3600	0.0056	0.0570
**Succinivibrio**	0.0145	0.1000	0.0002	0.0069	0.0000	0.0042	0.0173	0.1100
**Sutterella**	1.0000	1.0000	0.3593	0.5600	0.3593	0.5600	1.0000	1.0000
**Syner-01**	NA	NA	0.3593	0.5600	0.1662	0.3900	NA	NA
**Synergistales_X_X**	0.6513	0.8000	0.1058	0.3200	0.0499	0.2100	0.3428	0.5600
**Synergistes**	0.3593	0.5600	0.3593	0.5600	0.3593	0.5600	0.3593	0.5600
**SZUA-359**	0.0079	0.0680	0.0332	0.1700	0.0009	0.0170	0.2189	0.4800
**Tannerella**	0.5807	0.7600	0.5139	0.7000	0.3593	0.5600	0.3593	0.5600
**Tannerellaceae_X**	0.6636	0.8000	0.6025	0.7700	0.2598	0.5300	0.0916	0.2900
**TF01-11**	0.3021	0.5600	0.0114	0.0900	0.1535	0.3900	1.0000	1.0000
**Thermotalea**	0.3593	0.5600	0.3593	0.5600	1.0000	1.0000	0.3593	0.5600
**Tissierellaceae_X**	NA	NA	0.3593	0.5600	0.1662	0.3900	NA	NA
**Tissierellales_X_X**	1.0000	1.0000	0.0072	0.0630	0.0225	0.1300	0.0885	0.2800
**Treponema_A**	1.0000	1.0000	1.0000	1.0000	0.1798	0.4100	0.3593	0.5600
**Treponema_B**	0.7760	0.9000	1.0000	1.0000	0.2207	0.4800	0.5898	0.7600
**Treponema_C**	0.8467	0.9500	0.7150	0.8400	0.8467	0.9500	0.0480	0.2000
**Treponema_D**	0.1841	0.4200	0.5899	0.7600	0.5137	0.7000	0.1135	0.3300
**Treponemataceae_X**	0.0121	0.0920	0.0011	0.0190	0.0023	0.0320	0.0205	0.1200
**Treponematales_X_X**	0.0092	0.0770	0.0007	0.0150	0.0013	0.0210	0.0934	0.2900
**Tumebacillus**	0.0788	0.2600	0.4085	0.6100	0.3740	0.5700	0.3740	0.5700
**UBA1020**	0.1548	0.3900	1.0000	1.0000	0.1326	0.3700	0.3593	0.5600
**UBA1033**	0.1129	0.3300	0.0236	0.1400	0.0003	0.0086	0.2478	0.5100
**UBA1067**	0.0887	0.2800	0.1277	0.3600	0.0205	0.1200	0.5512	0.7300
**UBA1174**	0.2125	0.4700	0.2976	0.5600	0.8847	0.9800	0.4353	0.6400
**UBA1191**	0.5139	0.7000	0.1326	0.3700	0.0485	0.2000	0.5139	0.7000
**UBA1258**	0.0024	0.0330	0.0001	0.0047	0.0000	0.0039	0.0008	0.0160
**UBA1361**	0.9081	0.9900	0.4428	0.6400	0.0100	0.0810	0.0282	0.1500
**UBA1394**	0.8939	0.9800	0.2394	0.5100	0.0185	0.1200	0.0683	0.2600
**UBA1436**	0.0001	0.0047	0.0205	0.1200	0.0121	0.0920	0.0068	0.0620
**UBA1487**	0.3593	0.5600	0.3593	0.5600	1.0000	1.0000	1.0000	1.0000

**UBA1532**	0.2345	0.5000	0.8392	0.9500	0.9539	1.0000	0.0194	0.1200
**UBA1547**	0.0367	0.1700	0.0788	0.2600	0.3593	0.5600	NA	NA
**UBA1711**	0.3777	0.5700	0.7553	0.8800	0.5512	0.7300	0.0205	0.1200
**UBA1829**	0.0449	0.1900	0.0011	0.0190	0.0056	0.0570	0.0242	0.1400
**UBA2022**	1.0000	1.0000	1.0000	1.0000	1.0000	1.0000	0.3593	0.5600
**UBA2450**	0.7478	0.8800	0.8838	0.9800	0.6636	0.8000	0.2566	0.5300
**UBA2705**	0.3593	0.5600	NA	NA	0.3593	0.5600	0.0788	0.2600
**UBA3206**	0.4428	0.6400	0.6707	0.8100	0.0332	0.1700	0.0045	0.0500
**UBA4179**	0.2652	0.5400	0.4693	0.6700	0.0836	0.2700	0.0558	0.2300
**UBA4658**	0.1662	0.3900	0.3593	0.5600	NA	NA	NA	NA
**UBA5124**	0.0005	0.0120	0.1432	0.3900	0.0519	0.2100	0.0000	0.0014
**UBA5194**	0.4915	0.6900	0.7145	0.8400	0.0417	0.1900	0.2860	0.5600
**UBA5946**	0.3281	0.5600	0.5807	0.7600	0.6513	0.8000	1.0000	1.0000
**UBA636**	0.3593	0.5600	0.1129	0.3300	0.6513	0.8000	0.6513	0.8000
**UBA6984**	0.2880	0.5600	0.5171	0.7000	0.0210	0.1200	0.3165	0.5600
**UBA7182**	NA	NA	0.0788	0.2600	0.0788	0.2600	0.0011	0.0190
**UBA733**	0.7506	0.8800	0.8170	0.9300	0.1745	0.4100	0.6033	0.7700
**UBA7862**	0.0734	0.2600	0.0519	0.2100	0.0011	0.0190	1.0000	1.0000
**UBA8416**	0.6233	0.7900	0.2598	0.5300	0.4704	0.6700	0.9770	1.0000
**UBA8525**	0.4495	0.6500	0.0713	0.2600	0.0119	0.0920	0.0871	0.2800
**UBA8953**	0.1135	0.3300	0.1432	0.3900	0.0100	0.0810	0.2657	0.5400
**UBA932_X**	0.2795	0.5600	0.0347	0.1700	0.0604	0.2300	0.0228	0.1300
**UBA9983_A_X_X**	0.3593	0.5600	NA	NA	0.0001	0.0054	0.0004	0.0096
**V5-8f**	NA	NA	NA	NA	NA	NA	0.1662	0.3900
**Vallitalea_A**	0.3593	0.5600	1.0000	1.0000	0.3593	0.5600	1.0000	1.0000
**Vampirovibrionia_X_X_X**	1.0000	1.0000	0.3593	0.5600	1.0000	1.0000	0.3593	0.5600
**Varibaculum_A**	NA	NA	0.0788	0.2600	NA	NA	0.3593	0.5600
**Veillonella**	0.0340	0.1700	0.4067	0.6100	0.5403	0.7300	0.4453	0.6400
**Veillonella_A**	0.0585	0.2300	0.1060	0.3200	0.1039	0.3200	0.8939	0.9800
**Verrucomicrobiota_X_X_X_X**	1.0000	1.0000	0.2478	0.5100	0.5139	0.7000	1.0000	1.0000

**Victivallis**	0.6434	0.8000	0.1569	0.3900	0.0145	0.1000	0.7290	0.8600
**Vitiosangium**	0.1662	0.3900	0.1060	0.3200	0.7760	0.9000	0.8939	0.9800
**Weeksellaceae_X**	0.3593	0.5600	0.1662	0.3900	0.3593	0.5600	0.1662	0.3900
**Weissella**	NA	NA	0.3593	0.5600	0.3593	0.5600	0.0788	0.2600
**Williamwhitmania**	0.3177	0.5600	0.0044	0.0500	0.0013	0.0210	0.0449	0.1900
**Xanthomonadaceae_X**	0.2860	0.5600	1.0000	1.0000	0.2860	0.5600	0.3281	0.5600
**Xanthomonadales_X_X**	NA	NA	NA	NA	0.0165	0.1100	0.0367	0.1700
**Xanthomonas_A**	0.1662	0.3900	0.0788	0.2600	0.0165	0.1100	0.1662	0.3900
**Xanthomonas_B**	0.0012	0.0200	0.0424	0.1900	0.8257	0.9400	0.1217	0.3500
**XBB1006**	NA	NA	0.1662	0.3900	0.1662	0.3900	0.1662	0.3900
**XYC2-FULL-35-21**	0.1662	0.3900	0.3828	0.5800	0.6548	0.8000	0.1662	0.3900
**YD12-FULL-39-22**	0.2478	0.5100	0.2478	0.5100	1.0000	1.0000	1.0000	1.0000
**Zag1**	0.0002	0.0069	0.0001	0.0047	0.0001	0.0057	0.0205	0.1200
**Zag111**	0.1325	0.3700	0.0687	0.2600	0.1745	0.4100	1.0000	1.0000

Table S6: Pairwise-comparison of taxa in Collection-1 with other Collections (2 to 5) from samples of solid fractions. Suffix "_X" in genus name suggests that taxa was assigned at higher taxonomic levels than genus. Multiple suffixes are kept identifying exact level of assignment.

	Collection-2		Collection-3		Collection-4		Collection-5	
**Taxa**	Wilcoxon test p-value	Wilcoxon test, BH adjusted p-value	Wilcoxon test p-value	Wilcoxon test, BH adjusted p-value	Wilcoxon test p-value	Wilcoxon test, BH adjusted p-value	Wilcoxon test p-value	Wilcoxon test, BH adjusted p-value
**Phylum**								
**Acidobacteriota**	0.3440	0.5500	0.3440	0.5500	0.6059	0.7500	1.0000	1.0000
**Actinobacteriota**	0.7566	0.8500	0.4451	0.6500	0.5353	0.7200	0.7642	0.8500
**Bacteroidota**	0.0153	0.0680	0.0000	0.0002	0.0029	0.0270	0.0019	0.0190
**Bdellovibrionota**	0.0358	0.1300	0.0358	0.1300	0.0037	0.0270	0.0728	0.2000
**Campylobacterota**	0.3058	0.5400	0.0034	0.0270	0.1731	0.3900	0.3266	0.5500
**Chloroflexota**	0.0427	0.1500	0.0004	0.0054	0.0032	0.0270	0.0050	0.0350
**Cyanobacteria**	0.1415	0.3400	0.0153	0.0680	0.0088	0.0510	0.0077	0.0480
**Desulfobacterota**	0.3250	0.5500	0.0284	0.1100	0.0601	0.1800	0.5462	0.7200
**Desulfobacterota_A**	0.8756	0.9200	0.0079	0.0480	0.0265	0.1100	0.9586	0.9800
**Desulfuromonadota**	0.7619	0.8500	0.0574	0.1800	0.0545	0.1700	0.9265	0.9600
**Elusimicrobiota**	0.1472	0.3500	0.0677	0.1900	0.1923	0.4000	0.9919	1.0000
**Fibrobacterota**	0.3305	0.5500	0.1359	0.3300	0.0319	0.1200	0.2638	0.5100
**Firmicutes**	0.2727	0.5100	0.0183	0.0770	0.4429	0.6500	0.5601	0.7300
**Firmicutes_A**	0.1472	0.3500	0.0000	0.0005	0.0000	0.0002	0.0000	0.0004
**Firmicutes_B**	0.0040	0.0290	0.0608	0.1800	0.0097	0.0530	0.0006	0.0080
**Firmicutes_C**	0.0646	0.1900	0.7360	0.8500	0.6022	0.7500	0.2068	0.4200
**Firmicutes_I**	0.0811	0.2200	0.0207	0.0840	0.0103	0.0540	0.0103	0.0540
**Firmicutes_K**	0.0410	0.1500	0.5549	0.7300	0.4404	0.6500	0.1797	0.3900
**Fusobacteriota**	0.3588	0.5700	0.1518	0.3600	0.3173	0.5400	0.1835	0.4000
**Gemmatimonadota**	0.0535	0.1700	0.6059	0.7500	0.0988	0.2600	0.3440	0.5500
**Myxococcota**	0.7726	0.8600	0.6798	0.8000	0.2313	0.4500	0.0001	0.0016
**Patescibacteria**	0.0094	0.0520	0.0153	0.0680	0.0001	0.0016	0.0000	0.0000
**Planctomycetota**	0.1904	0.4000	0.0014	0.0150	0.0091	0.0520	0.0182	0.0770

**Proteobacteria**	0.0271	0.1100	0.0120	0.0590	0.5464	0.7200	0.0437	0.1500
**Riflebacteria**	0.0489	0.1600	0.0814	0.2200	0.0001	0.0022	0.2977	0.5400
**Spirochaetota**	0.8622	0.9100	0.0172	0.0760	0.4308	0.6400	0.9919	1.0000
**Synergistota**	0.8933	0.9300	0.2047	0.4200	0.0851	0.2300	0.9918	1.0000
**Unclassified Phylum**	0.0153	0.0680	0.0000	0.0002	0.0000	0.0001	0.0027	0.0260
**Verrucomicrobiota**	0.5062	0.7000	0.7670	0.8500	0.1994	0.4100	0.9106	0.9400
**Genus**								
**32-67-11**	NA	NA	NA	NA	0.3593	0.5400	0.1662	0.3700
**4C28d-15_X_X**	0.1918	0.4100	0.4315	0.6200	0.1918	0.4100	0.6518	0.7800
**992a**	0.6404	0.7800	0.4315	0.6200	0.1471	0.3700	0.6926	0.8200
**Acetivibrionaceae_X**	0.8173	0.9100	0.5512	0.7100	0.5137	0.6800	0.5137	0.6800
**Acetoanaerobium**	NA	NA	NA	NA	NA	NA	0.0367	0.1600
**Acetobacteraceae_X**	NA	NA	0.0788	0.2500	0.3593	0.5400	0.0788	0.2500
**Acholeplasma_C**	0.4428	0.6300	0.0597	0.2200	0.1978	0.4100	0.4095	0.5900
**Acholeplasma_D**	0.0165	0.0970	0.0165	0.0970	0.0029	0.0390	0.0788	0.2500
**Acholeplasmatales_X_X**	0.0705	0.2500	0.1304	0.3500	0.1182	0.3300	0.1057	0.3000
**Achromobacter**	0.3593	0.5400	0.1662	0.3700	0.3593	0.5400	NA	NA
**Acinetobacter**	0.8097	0.9000	0.7229	0.8300	0.6370	0.7800	0.9735	1.0000
**Actinobacillus**	0.7553	0.8600	0.1600	0.3700	0.0332	0.1600	0.0005	0.0190
**Actinobacillus_A**	0.2614	0.5000	0.2734	0.5100	0.9754	1.0000	0.3358	0.5400
**Actinomyces**	0.4953	0.6800	0.2194	0.4400	0.1535	0.3700	0.0071	0.0610
**Actinomycetaceae_X**	0.2394	0.4700	0.0585	0.2200	0.1223	0.3300	0.0050	0.0540
**Actinomycetales_X_X**	0.3593	0.5400	0.1662	0.3700	NA	NA	NA	NA
**Acutalibacteraceae_X**	0.2168	0.4400	0.3173	0.5400	0.4004	0.5900	1.0000	1.0000
**Aerococcaceae_X**	0.4021	0.5900	0.0120	0.0870	0.9310	0.9900	0.7935	0.9000
**Aerococcus**	NA	NA	0.0071	0.0610	0.0165	0.0970	0.1662	0.3700
**Agarilytica**	NA	NA	NA	NA	0.1662	0.3700	0.3593	0.5400
**Agitococcus**	0.0598	0.2200	0.0984	0.2900	0.6565	0.7800	0.7577	0.8600
**Akkermansia**	1.0000	1.0000	0.0039	0.0460	0.3593	0.5400	0.2860	0.5300
**Alcanivorax_A**	0.0029	0.0390	0.0011	0.0240	0.1662	0.3700	0.0367	0.1600

**Algoriphagus**	NA	NA	NA	NA	0.3593	0.5400	NA	NA
**Alishewanella**	0.0008	0.0230	0.0000	0.0052	0.0000	0.0058	0.0195	0.1100
**Alphaproteobacteria_X_X_X**	0.3404	0.5400	0.8851	0.9600	0.5137	0.6800	0.4883	0.6700
**Anaerofilum**	0.9770	1.0000	0.0173	0.0990	0.0887	0.2700	0.0687	0.2400
**Anaerofustis**	0.5283	0.7000	0.0733	0.2500	0.3615	0.5400	0.3056	0.5400
**Anaerorhabdus**	0.0485	0.1900	0.1058	0.3000	0.3035	0.5400	0.1058	0.3000
**Anaerosporobacter**	0.1662	0.3700	0.0367	0.1600	0.3593	0.5400	NA	NA
**Anaerotignum**	0.8014	0.9000	0.0311	0.1500	0.1392	0.3600	0.5358	0.7000
**Anaerovibrio**	1.0000	1.0000	0.1129	0.3200	0.0133	0.0940	0.0485	0.1900
**Anaerovoracaceae_X**	0.0847	0.2600	0.0678	0.2400	0.0502	0.2000	0.0001	0.0120
**Aquabacterium**	0.0165	0.0970	0.0000	0.0052	0.0029	0.0390	0.3593	0.5400
**Arabia**	0.5139	0.6800	0.0073	0.0620	0.5139	0.6800	0.5139	0.6800
**Arenimonas**	0.1662	0.3700	0.3593	0.5400	0.3593	0.5400	0.3593	0.5400
**Aromatoleum**	NA	NA	0.3593	0.5400	0.3593	0.5400	0.0367	0.1600
**Asticcacaulis**	NA	NA	NA	NA	0.0788	0.2500	0.3593	0.5400
**Bacilli_X_X_X**	0.7270	0.8400	0.1935	0.4100	0.1569	0.3700	0.6416	0.7800
**Bacillus**	0.0165	0.0970	0.0578	0.2200	0.0165	0.0970	0.0165	0.0970
**Bacillus_AD**	NA	NA	0.0165	0.0970	NA	NA	0.3593	0.5400
**Bacillus_W**	0.3593	0.5400	0.3593	0.5400	0.3593	0.5400	0.3593	0.5400
**Bact-08**	0.8614	0.9500	0.8392	0.9300	0.5064	0.6800	0.0090	0.0720
**Bacteria_X_X_X_X_X**	0.0519	0.2000	0.0014	0.0280	0.0007	0.0200	0.0002	0.0140
**Bacteroidaceae_X**	0.3777	0.5600	0.5512	0.7100	0.2189	0.4400	0.5137	0.6800
**Bacteroidales_X_X**	0.3474	0.5400	0.8874	0.9600	0.0011	0.0240	0.0100	0.0770
**Bacteroides**	0.7950	0.9000	0.1935	0.4100	0.4353	0.6200	0.0020	0.0340
**Bacteroidia_X_X_X**	0.0597	0.2200	0.0100	0.0770	0.0004	0.0170	0.0023	0.0370
**Bdellovibrio**	NA	NA	0.1662	0.3700	0.0788	0.2500	0.1662	0.3700
**Beijerinckiaceae_X**	0.0165	0.0970	0.0165	0.0970	0.0165	0.0970	0.0165	0.0970
**Bibersteinia**	0.0002	0.0130	0.0000	0.0075	0.0459	0.1900	0.2994	0.5400
**Blastomonas**	0.3593	0.5400	NA	NA	NA	NA	NA	NA
**Blautia_A**	0.5420	0.7100	0.0012	0.0250	0.0423	0.1800	0.0018	0.0320

**Brachymonas**	0.3712	0.5600	0.8794	0.9600	0.7614	0.8700	0.2478	0.4800
**Brevundimonas**	0.0034	0.0430	0.0018	0.0320	0.1798	0.3900	0.2652	0.5000
**BRH-c57**	0.3593	0.5400	NA	NA	NA	NA	0.1662	0.3700
**Burkholderiaceae_X**	0.5403	0.7100	0.8339	0.9300	0.1214	0.3300	0.8750	0.9600
**Butyrivibrio**	0.4021	0.5900	0.0018	0.0320	0.0068	0.0610	0.1005	0.3000
**C941**	0.7987	0.9000	0.9323	0.9900	0.5137	0.6800	0.0018	0.0320
**CAG-180**	0.4255	0.6100	0.1039	0.3000	0.0255	0.1300	0.7145	0.8300
**CAG-312**	NA	NA	0.0165	0.0970	0.1662	0.3700	0.3593	0.5400
**CAG-313**	0.0711	0.2500	0.0001	0.0110	0.0014	0.0280	0.0616	0.2300
**CAG-354**	0.2415	0.4700	0.2657	0.5000	0.5512	0.7100	0.0205	0.1100
**CAG-462**	0.3186	0.5400	0.0100	0.0770	0.4095	0.5900	0.0009	0.0230
**CAG-465**	0.5807	0.7300	1.0000	1.0000	0.5807	0.7300	0.5807	0.7300
**CAG-475**	0.5139	0.6800	1.0000	1.0000	0.0578	0.2200	0.2478	0.4800
**CAG-495**	0.5512	0.7100	0.5137	0.6800	0.9774	1.0000	0.4095	0.5900
**CAG-508_X**	0.3593	0.5400	NA	NA	0.1662	0.3700	0.3593	0.5400
**CAG-826_X**	0.4004	0.5900	0.0024	0.0380	0.0085	0.0700	0.1080	0.3100
**CAG-873**	0.8939	0.9700	0.2085	0.4300	0.4709	0.6600	0.1662	0.3700
**CAG-878**	0.8356	0.9300	0.0050	0.0540	0.0459	0.1900	0.1857	0.4000
**Campylobacter**	0.5139	0.6800	0.0794	0.2500	0.1217	0.3300	0.0131	0.0930
**Campylobacter_A**	0.2834	0.5300	0.1029	0.3000	0.3318	0.5400	0.0057	0.0590
**Campylobacter_B**	0.6926	0.8200	0.2711	0.5100	0.1129	0.3200	0.0367	0.1600
**Campylobacteraceae_X**	0.9395	1.0000	0.3355	0.5400	0.9395	1.0000	0.2478	0.4800
**Capnocytophaga**	0.5807	0.7300	0.5807	0.7300	1.0000	1.0000	0.3593	0.5400
**Caryophanon**	NA	NA	0.1662	0.3700	NA	NA	NA	NA
**Caviibacter**	0.2286	0.4600	0.8317	0.9200	0.4406	0.6300	0.1955	0.4100
**Cellvibrio**	0.0809	0.2500	0.0405	0.1700	0.0685	0.2400	0.5807	0.7300
**Cellvibrionaceae_X**	NA	NA	NA	NA	0.0367	0.1600	0.3593	0.5400
**Chishuiella**	0.0367	0.1600	0.1662	0.3700	0.0165	0.0970	0.0165	0.0970
**CHKCI001**	NA	NA	NA	NA	0.1662	0.3700	0.3593	0.5400
**Christensenellales_X_X**	0.0787	0.2500	0.0287	0.1400	0.0558	0.2100	0.1568	0.3700

**Chryseobacterium**	1.0000	1.0000	0.6513	0.7800	0.6513	0.7800	0.2076	0.4300
**Chryseobacterium_B**	0.3593	0.5400	NA	NA	0.3593	0.5400	NA	NA
**Chryseobacterium_D**	0.3593	0.5400	0.3593	0.5400	0.1662	0.3700	0.1662	0.3700
**Cloacibacterium**	0.3555	0.5400	0.0887	0.2700	0.4188	0.6000	0.0057	0.0590
**Clostridia_X_X_X**	0.7553	0.8600	0.0068	0.0610	0.0036	0.0440	0.0003	0.0170
**Clostridium**	0.6142	0.7600	0.5500	0.7100	0.9291	0.9900	0.0616	0.2300
**Clostridium_A**	0.6436	0.7800	0.4185	0.6000	0.1188	0.3300	0.1217	0.3300
**Clostridium_L**	NA	NA	0.3593	0.5400	NA	NA	NA	NA
**Clostridium_N**	1.0000	1.0000	0.5972	0.7400	0.1406	0.3600	0.1662	0.3700
**Comamonas**	0.2860	0.5300	0.0788	0.2500	0.2860	0.5300	0.5420	0.7100
**Corynebacterium**	0.0165	0.0970	0.1662	0.3700	NA	NA	NA	NA
**CP2B**	0.3593	0.5400	0.0161	0.0970	0.5807	0.7300	1.0000	1.0000
**Cytophagales_X_X**	0.3593	0.5400	0.0165	0.0970	0.0071	0.0610	NA	NA
**Denitrobacterium**	0.3828	0.5600	0.6548	0.7800	0.5189	0.6900	0.9645	1.0000
**Dermatophilaceae_X**	0.4225	0.6100	0.6380	0.7800	0.1387	0.3600	0.1758	0.3800
**Desulfobacterota_X_X_X_X**	0.5898	0.7300	0.3355	0.5400	0.1736	0.3800	0.5972	0.7400
**Desulfovibrio**	0.9645	1.0000	0.8939	0.9700	0.3689	0.5500	0.1662	0.3700
**Desulfovibrionaceae_X**	0.4154	0.6000	0.1244	0.3400	0.6647	0.7900	0.0826	0.2600
**Devosia**	0.0367	0.1600	0.0029	0.0390	0.0165	0.0970	0.1662	0.3700
**Dialister_A**	0.1798	0.3900	0.0161	0.0970	0.0950	0.2800	1.0000	1.0000
**Dichelobacter**	0.0604	0.2200	0.0177	0.1000	0.3688	0.5500	0.5564	0.7200
**Dietzia**	0.0950	0.2800	0.0809	0.2500	1.0000	1.0000	1.0000	1.0000
**Dongia**	0.3593	0.5400	0.0788	0.2500	0.0165	0.0970	0.0165	0.0970
**Dorea**	NA	NA	0.0071	0.0610	0.1662	0.3700	NA	NA
**DTU053**	0.1905	0.4000	0.2144	0.4400	0.4704	0.6600	0.2592	0.5000
**Duncaniella**	0.1326	0.3500	0.3740	0.5600	0.5139	0.6800	1.0000	1.0000
**Dysgonomonadaceae_X**	1.0000	1.0000	0.3593	0.5400	1.0000	1.0000	1.0000	1.0000
**Echinicola**	0.1798	0.3900	0.0015	0.0280	0.0037	0.0450	0.1798	0.3900
**Eggerthellaceae_X**	0.8097	0.9000	0.0025	0.0390	0.0485	0.1900	0.7464	0.8600
**Elizabethkingia**	0.3593	0.5400	NA	NA	0.1662	0.3700	0.1662	0.3700

**Emergencia**	0.4255	0.6100	0.8163	0.9100	0.4947	0.6800	0.1129	0.3200
**Empedobacter**	0.0367	0.1600	0.0071	0.0610	0.0029	0.0390	0.0071	0.0610
**Endomicrobium**	0.6513	0.7800	0.3828	0.5600	0.4255	0.6100	0.5420	0.7100
**Endomicrobium_A**	0.0000	0.0067	0.0000	0.0047	0.0000	0.0052	0.0001	0.0085
**Enterobacterales_X_X**	0.4695	0.6600	0.6707	0.7900	0.8428	0.9300	0.2657	0.5000
**Enterobacteriaceae_X**	0.0045	0.0510	0.0068	0.0610	0.0205	0.1100	0.7987	0.9000
**Enterococcaceae_X**	0.1951	0.4100	0.0705	0.2500	0.4851	0.6700	0.1610	0.3700
**Enterococcus**	0.0367	0.1600	0.0165	0.0970	0.0367	0.1600	0.0071	0.0610
**Erysipelatoclostridiaceae_X**	NA	NA	0.1662	0.3700	0.3593	0.5400	0.3593	0.5400
**Erysipelothrix**	0.6647	0.7900	0.0402	0.1700	0.8170	0.9100	0.8838	0.9600
**Erysipelotrichaceae_X**	0.2218	0.4400	0.9307	0.9900	0.9079	0.9800	0.1262	0.3400
**Erysipelotrichales_X_X**	0.3593	0.5400	NA	NA	NA	NA	NA	NA
**Eubacterium_C**	0.3593	0.5400	0.3593	0.5400	0.3593	0.5400	1.0000	1.0000
**Eubacterium_E**	0.4685	0.6600	0.0007	0.0220	0.0043	0.0490	0.0259	0.1300
**Eubacterium_F**	0.0000	0.0058	0.0121	0.0870	0.0121	0.0870	0.7553	0.8600
**Eubacterium_Q**	0.3253	0.5400	0.6297	0.7700	0.2913	0.5300	0.0567	0.2200
**Eubacterium_S**	0.8851	0.9600	0.0225	0.1200	0.9769	1.0000	0.1563	0.3700
**F0040**	0.9323	0.9900	0.0780	0.2500	0.0284	0.1400	0.0016	0.0310
**F0058**	0.8386	0.9300	0.5049	0.6800	0.1905	0.4000	0.0020	0.0340
**F0422**	0.1432	0.3700	0.0068	0.0610	0.0009	0.0230	0.0055	0.0580
**F0428**	0.8397	0.9300	0.1571	0.3700	0.8398	0.9300	0.0463	0.1900
**F082**	0.0022	0.0370	0.0121	0.0870	0.0056	0.0580	0.6297	0.7700
**FD2005**	0.2039	0.4200	0.0780	0.2500	0.9774	1.0000	0.5899	0.7300
**Fermentimonas**	0.6931	0.8200	0.8821	0.9600	0.4406	0.6300	0.0071	0.0610
**Fibrobacter**	0.7987	0.9000	0.4776	0.6600	0.5899	0.7300	0.3777	0.5600
**Fibrobacter_A**	0.3956	0.5800	0.5927	0.7400	0.0552	0.2100	0.7150	0.8300
**Firm-04**	0.0220	0.1200	0.0449	0.1900	0.0242	0.1300	0.8173	0.9100
**Firm-16**	0.2880	0.5300	0.3580	0.5400	0.4953	0.6800	0.1087	0.3100
**Firmicutes_A_X_X_X_X**	0.7112	0.8300	0.0656	0.2400	0.0395	0.1700	0.5897	0.7300
**Firmicutes_B_X_X_X_X**	0.0004	0.0180	0.0153	0.0970	0.0011	0.0240	0.0002	0.0140

**Flavobacteriaceae_X**	0.3777	0.5600	0.6297	0.7700	0.1432	0.3700	0.0141	0.0970
**Flavobacteriales_X_X**	0.0001	0.0110	0.2144	0.4400	0.0006	0.0200	0.2356	0.4700
**Flavobacterium**	0.5807	0.7300	0.0089	0.0720	0.0010	0.0240	0.0073	0.0620
**Flavobacterium_A**	0.0788	0.2500	0.0001	0.0120	0.0001	0.0120	0.1662	0.3700
**Flexilinea**	0.1920	0.4100	0.0051	0.0540	0.1406	0.3600	0.0206	0.1100
**Fodinicurvata**	1.0000	1.0000	1.0000	1.0000	0.3593	0.5400	0.3593	0.5400
**Fusicatenibacter**	0.0405	0.1700	0.0847	0.2600	0.9274	0.9900	0.4479	0.6300
**Fusobacterium**	0.0367	0.1600	0.1662	0.3700	0.1662	0.3700	0.3593	0.5400
**Fusobacterium_A**	0.6649	0.7900	0.5443	0.7100	0.7950	0.9000	0.0040	0.0470
**Fusobacterium_C**	0.4428	0.6300	0.1782	0.3900	0.6707	0.7900	0.0145	0.0970
**Gammaproteobacteria_X_X_X**	0.0007	0.0200	0.1978	0.4100	0.1432	0.3700	0.7125	0.8300
**Gastranaerophilaceae_X**	0.3593	0.5400	0.0367	0.1600	0.0367	0.1600	0.0788	0.2500
**GCA-2733575**	NA	NA	0.0788	0.2500	NA	NA	NA	NA
**GCA-900066135**	0.3593	0.5400	0.0367	0.1600	NA	NA	0.3593	0.5400
**GCA-900066495**	0.1662	0.3700	0.0004	0.0170	0.1662	0.3700	0.1662	0.3700
**GCA-900066905**	0.3740	0.5600	0.0025	0.0390	0.0018	0.0320	0.0090	0.0720
**GCA-900066995**	0.0910	0.2800	0.6228	0.7700	0.1739	0.3800	0.4322	0.6200
**Gemella_A**	0.3828	0.5600	0.0679	0.2400	0.0905	0.2700	0.9645	1.0000
**Gemmatimonadaceae_X**	0.0950	0.2800	0.3593	0.5400	0.3593	0.5400	0.3593	0.5400
**Gemmatimonas**	NA	NA	0.3593	0.5400	0.1662	0.3700	0.1662	0.3700
**Gemmobacter_A**	NA	NA	0.3593	0.5400	NA	NA	0.0071	0.0610
**Geothermobacter**	0.5088	0.6800	0.3710	0.5600	0.1123	0.3200	0.9230	0.9900
**Gluconobacter**	0.3593	0.5400	0.3593	0.5400	0.1662	0.3700	0.0788	0.2500
**Glutamicibacter**	0.3593	0.5400	NA	NA	0.0788	0.2500	0.0788	0.2500
**GN02-873**	0.1156	0.3200	0.0139	0.0970	0.8097	0.9000	0.2394	0.4700
**Gottschalkiaceae_X**	0.0367	0.1600	0.0367	0.1600	0.0367	0.1600	0.1129	0.3200
**GWE2-31-10**	0.3236	0.5400	0.4693	0.6600	0.5821	0.7300	0.2973	0.5400
**Halomonas_A**	NA	NA	0.3593	0.5400	NA	NA	0.3593	0.5400
**Helcococcaceae_X**	0.1634	0.3700	0.0051	0.0550	0.0016	0.0300	0.0004	0.0170
**Helcococcus**	0.0129	0.0930	0.0003	0.0170	0.0114	0.0860	0.9395	1.0000

**Hepatobacter**	NA	NA	0.0788	0.2500	0.0788	0.2500	0.1662	0.3700
**Humitalea**	0.0788	0.2500	0.0788	0.2500	0.0788	0.2500	0.0788	0.2500
**Hungatella_A**	0.8173	0.9100	0.0001	0.0090	0.0005	0.0190	0.0002	0.0140
**Hyphomonas**	NA	NA	NA	NA	0.1662	0.3700	0.1662	0.3700
**Inquilinus**	0.0165	0.0970	0.0165	0.0970	0.0165	0.0970	0.0165	0.0970
**Kapabacteriaceae_X**	0.0424	0.1800	0.1058	0.3000	0.0154	0.0970	0.8939	0.9700
**Kingella**	0.0173	0.0990	0.8428	0.9300	0.6707	0.7900	0.0092	0.0730
**Kiritimatiellae_X_X_X**	0.0051	0.0540	0.0029	0.0390	0.1257	0.3400	0.1182	0.3300
**KLE1796**	0.0165	0.0970	0.0029	0.0390	0.0367	0.1600	0.3593	0.5400
**Kocuria**	0.0165	0.0970	0.0165	0.0970	0.0165	0.0970	0.0165	0.0970
**Lachnoanaerobaculum**	0.7192	0.8300	0.0034	0.0430	0.0809	0.2500	0.0036	0.0440
**Lachnospira**	1.0000	1.0000	0.6513	0.7800	0.0003	0.0170	0.2076	0.4300
**Lachnospiraceae_X**	0.6297	0.7700	0.0011	0.0240	0.0684	0.2400	0.0205	0.1100
**Lachnospirales_X_X**	0.7125	0.8300	0.5137	0.6800	0.6707	0.7900	0.4776	0.6600
**Lactobacillales_X_X**	0.5283	0.7000	0.7293	0.8400	0.6825	0.8100	0.1610	0.3700
**Lactobacillus_F**	NA	NA	0.3593	0.5400	0.3593	0.5400	0.1662	0.3700
**Lactococcus**	0.0367	0.1600	0.0011	0.0240	0.0011	0.0240	0.0071	0.0610
**Lactonifactor**	0.3740	0.5600	0.0568	0.2200	0.1798	0.3900	0.5807	0.7300
**Lancefieldella**	0.3281	0.5400	0.0087	0.0710	0.1548	0.3700	1.0000	1.0000
**Leaf454**	0.0066	0.0610	0.0001	0.0090	0.0000	0.0067	0.0005	0.0190
**Lenti-01**	0.4528	0.6400	0.0007	0.0200	0.0173	0.0990	0.0734	0.2500
**Lentimicrobium**	0.1978	0.4100	0.0449	0.1900	0.7987	0.9000	0.0068	0.0610
**Lentisphaeria_X_X_X**	0.1662	0.3700	0.1662	0.3700	NA	NA	0.1662	0.3700
**Leptotrichiaceae_X**	0.5898	0.7300	0.6404	0.7800	0.5412	0.7100	0.0788	0.2500
**Leuconostoc**	0.0788	0.2500	0.0029	0.0390	0.0071	0.0610	0.0029	0.0390
**Lysobacter**	0.0165	0.0970	0.0165	0.0970	0.0165	0.0970	0.0165	0.0970
**Mailhella**	0.0417	0.1800	0.0256	0.1300	0.0521	0.2000	0.2795	0.5200
**Mannheimia**	0.0166	0.0970	0.8852	0.9600	0.7072	0.8300	0.0010	0.0240
**Marinilabiliaceae_X**	0.3740	0.5600	0.1798	0.3900	0.5807	0.7300	1.0000	1.0000
**Marseille-P3160**	0.3593	0.5400	NA	NA	0.3593	0.5400	NA	NA

**Massilia_B**	NA	NA	0.0071	0.0610	NA	NA	NA	NA
**Massilibacteroides**	0.2657	0.5000	0.9774	1.0000	0.1600	0.3700	0.0007	0.0200
**Metamycoplasma**	0.2330	0.4600	0.4985	0.6800	0.4947	0.6800	0.0367	0.1600
**Methylobacterium**	0.3593	0.5400	0.6513	0.7800	0.6513	0.7800	0.6513	0.7800
**Micavibrionaceae_X**	NA	NA	NA	NA	NA	NA	0.1662	0.3700
**Micrococcaceae_X**	0.3593	0.5400	0.3593	0.5400	NA	NA	0.3593	0.5400
**Monoglobus**	0.4188	0.6000	0.1432	0.3700	0.9323	0.9900	0.7948	0.9000
**Moraxella**	0.4095	0.5900	0.3186	0.5400	0.3186	0.5400	0.0332	0.1600
**Moraxella_A**	0.3740	0.5600	0.0568	0.2200	0.3740	0.5600	0.3593	0.5400
**Moraxella_C**	0.0788	0.2500	0.0029	0.0390	NA	NA	0.1662	0.3700
**Moraxellaceae_X**	0.0042	0.0490	0.0023	0.0370	0.0308	0.1500	0.1060	0.3000
**Mucilaginibacter**	NA	NA	NA	NA	NA	NA	0.3593	0.5400
**Muribaculaceae_X**	0.2913	0.5300	0.6707	0.7900	0.8428	0.9300	0.0145	0.0970
**Myroides**	NA	NA	NA	NA	NA	NA	0.3593	0.5400
**Ndongobacter**	0.2194	0.4400	0.2194	0.4400	0.2860	0.5300	0.0788	0.2500
**Negativicutes_X_X_X**	0.2726	0.5100	0.4347	0.6200	0.5825	0.7300	0.1651	0.3700
**Neisseria_B**	0.1918	0.4100	0.9486	1.0000	0.4360	0.6200	0.2085	0.4300
**Neisseria_G**	0.1662	0.3700	0.6513	0.7800	0.1662	0.3700	0.1662	0.3700
**Neisseriaceae_X**	0.4353	0.6200	0.9081	0.9800	0.1432	0.3700	0.0492	0.2000
**Neorhizobium**	0.1058	0.3000	0.5927	0.7400	0.1912	0.4100	0.1912	0.4100
**Niveispirillum**	0.0788	0.2500	NA	NA	0.3593	0.5400	NA	NA
**NS-102**	0.0367	0.1600	0.0165	0.0970	0.0165	0.0970	0.1662	0.3700
**OEMR01**	0.7478	0.8600	0.1569	0.3700	0.0641	0.2300	0.4015	0.5900
**OLB17**	0.3740	0.5600	0.3740	0.5600	0.6513	0.7800	1.0000	1.0000
**Olegusella**	1.0000	1.0000	0.1129	0.3200	1.0000	1.0000	0.5139	0.6800
**Olsenella**	0.0179	0.1000	0.0023	0.0370	0.2708	0.5100	0.2593	0.5000
**Opitutaceae_X**	0.3777	0.5600	0.1005	0.3000	0.1600	0.3700	0.8428	0.9300
**Opitutales_X_X**	0.3593	0.5400	NA	NA	NA	NA	NA	NA
**Oribacterium**	NA	NA	0.0788	0.2500	0.0165	0.0970	0.1662	0.3700
**Orrella**	0.8939	0.9700	0.5139	0.6800	0.1662	0.3700	0.1662	0.3700

**Oscillibacter**	0.9735	1.0000	0.0539	0.2100	0.0910	0.2800	0.1087	0.3100
**Oscillospiraceae_X**	0.6297	0.7700	0.0145	0.0970	0.0083	0.0690	0.0002	0.0140
**Oscillospirales_X_X**	0.0597	0.2200	0.0887	0.2700	0.0018	0.0320	0.0597	0.2200
**Paenibacillus_J**	0.3593	0.5400	0.1662	0.3700	NA	NA	0.0788	0.2500
**Paeniclostridium**	0.5139	0.6800	0.0073	0.0620	0.2860	0.5300	1.0000	1.0000
**PALSA-1355**	0.5358	0.7000	0.7770	0.8800	0.7770	0.8800	0.2652	0.5000
**Paludibacteraceae_X**	0.2089	0.4300	0.2308	0.4600	0.0389	0.1700	1.0000	1.0000
**Paramesorhizobium**	0.0485	0.1900	0.0165	0.0970	0.1058	0.3000	0.1912	0.4100
**Paramuribaculum**	0.0090	0.0720	0.0262	0.1300	0.0109	0.0830	0.7145	0.8300
**Parapedobacter**	0.3593	0.5400	1.0000	1.0000	0.3593	0.5400	0.3593	0.5400
**Paraprevotella**	0.0018	0.0320	0.0173	0.0990	0.0004	0.0170	0.5817	0.7300
**Parvibaculum**	0.0006	0.0200	0.0002	0.0140	0.0003	0.0170	0.0481	0.1900
**Parvimonas**	0.3226	0.5400	0.5830	0.7300	0.1696	0.3700	0.0099	0.0770
**Pasteurellaceae_X**	0.0597	0.2200	0.0083	0.0690	0.1978	0.4100	0.0045	0.0510
**Pauljensenia**	0.3712	0.5600	0.4453	0.6300	0.1223	0.3300	0.0165	0.0970
**Pedosphaeraceae_X**	0.0007	0.0200	0.0005	0.0190	0.0029	0.0390	0.0056	0.0580
**PeH17**	0.7125	0.8300	0.0780	0.2500	0.3777	0.5600	0.4428	0.6300
**Peptostreptococcaceae_X**	0.5426	0.7100	0.0313	0.1500	0.0153	0.0970	0.0015	0.0280
**Peptostreptococcales_X_X**	0.3864	0.5700	0.7987	0.9000	0.1005	0.3000	0.0012	0.0260
**Phycorickettsia**	NA	NA	NA	NA	0.3593	0.5400	0.3593	0.5400
**Pigmentiphaga**	NA	NA	0.3593	0.5400	NA	NA	0.3593	0.5400
**Pirellulaceae_X**	0.3593	0.5400	0.0165	0.0970	0.0788	0.2500	0.3593	0.5400
**Pirellulales_X_X**	0.2818	0.5300	0.0013	0.0280	0.0011	0.0240	0.0028	0.0390
**Planctomycetes_X_X_X**	0.5139	0.6800	0.0284	0.1400	0.1129	0.3200	0.0578	0.2200
**Planctomycetota_X_X_X_X**	0.0639	0.2300	0.1361	0.3600	0.1080	0.3100	0.2308	0.4600
**Porphyromonas**	0.1978	0.4100	0.6707	0.7900	0.6297	0.7700	0.0042	0.0490
**Prevotella**	0.0449	0.1900	0.0068	0.0610	0.3474	0.5400	0.4095	0.5900
**Prevotellamassilia**	0.0242	0.1300	0.0145	0.0970	0.0023	0.0370	0.0018	0.0320
**Prolixibacteraceae_X**	0.5137	0.6800	0.0083	0.0690	0.0011	0.0240	0.2415	0.4700
**Prosthecobacter**	NA	NA	0.0367	0.1600	0.0071	0.0610	0.0011	0.0240

**Proteiniclasticum**	0.3828	0.5600	0.0000	0.0070	0.0002	0.0140	0.1223	0.3300
**Proteobacteria_X_X_X_X**	0.3777	0.5600	0.2189	0.4400	0.0332	0.1600	0.0086	0.0700
**Pseudaminobacter**	0.1662	0.3700	0.0000	0.0052	0.0788	0.2500	0.0788	0.2500
**Pseudomonadaceae_X**	0.1058	0.3000	1.0000	1.0000	0.3035	0.5400	0.1058	0.3000
**Pseudomonadales_X_X**	0.5807	0.7300	0.3740	0.5600	0.0485	0.1900	0.5807	0.7300
**Pseudomonas_A**	0.0656	0.2400	0.0284	0.1400	0.2023	0.4200	0.8257	0.9200
**Pseudomonas_B**	0.5139	0.6800	0.3281	0.5400	0.5807	0.7300	0.5807	0.7300
**Pseudomonas_D**	0.3593	0.5400	0.3593	0.5400	0.3593	0.5400	0.3593	0.5400
**Pseudomonas_E**	NA	NA	NA	NA	0.3593	0.5400	0.3593	0.5400
**Pseudomonas_F**	0.1662	0.3700	NA	NA	NA	NA	NA	NA
**Pseudomonas_M**	0.0367	0.1600	0.7569	0.8600	0.7052	0.8300	0.4255	0.6100
**Pygmaiobacter**	0.1244	0.3400	0.2582	0.5000	0.6647	0.7900	0.0015	0.0280
**Pyramidobacter**	0.8874	0.9600	0.4095	0.5900	0.2415	0.4700	0.0242	0.1300
**RC9**	0.8874	0.9600	0.9774	1.0000	0.1005	0.3000	0.0284	0.1400
**RF16**	0.7125	0.8300	0.1600	0.3700	0.1782	0.3900	0.1432	0.3700
**Rhizobiaceae_X**	0.1058	0.3000	0.0165	0.0970	0.0485	0.1900	0.3035	0.5400
**Rhizobiales_X_X**	0.5972	0.7400	0.8574	0.9400	0.7614	0.8700	0.0788	0.2500
**Rhodospirillaceae_X**	0.3593	0.5400	0.3593	0.5400	0.0788	0.2500	NA	NA
**Riemerella**	0.5139	0.6800	0.0340	0.1600	0.0950	0.2800	0.3593	0.5400
**Roseomonas**	NA	NA	0.3593	0.5400	NA	NA	NA	NA
**Rothia**	0.0788	0.2500	0.3593	0.5400	NA	NA	NA	NA
**RUG131**	0.9323	0.9900	0.4776	0.6600	0.3777	0.5600	0.1569	0.3700
**RUG163**	0.7987	0.9000	0.1432	0.3700	0.0597	0.2200	0.0011	0.0240
**RUG350**	0.8874	0.9600	0.0887	0.2700	0.1600	0.3700	0.2135	0.4300
**Ruminiclostridium**	0.2415	0.4700	0.0000	0.0013	0.0000	0.0013	0.0001	0.0090
**Ruminiclostridium_C**	0.9323	0.9900	0.1978	0.4100	0.5512	0.7100	0.0068	0.0610
**Ruminiclostridium_D**	0.7145	0.8300	0.2454	0.4800	0.2194	0.4400	0.9395	1.0000
**Ruminiclostridium_F**	0.3593	0.5400	0.3593	0.5400	NA	NA	0.0788	0.2500
**Ruminococcaceae_X**	0.0121	0.0870	0.0029	0.0390	0.1135	0.3200	0.4776	0.6600
**Ruminococcus**	0.1626	0.3700	0.5830	0.7300	0.2913	0.5300	0.0027	0.0390

**Ruminococcus_C**	0.8794	0.9600	0.0088	0.0720	0.0124	0.0890	0.0036	0.0440
**Ruminococcus_D**	0.1600	0.3700	0.1782	0.3900	0.7125	0.8300	0.0145	0.0970
**Ruminococcus_F**	0.6233	0.7700	0.6707	0.7900	0.8874	0.9600	0.2726	0.5100
**Saccharibacillus**	NA	NA	0.3593	0.5400	0.1662	0.3700	0.1662	0.3700
**Saccharicrinis**	NA	NA	0.0011	0.0240	0.0011	0.0240	0.0367	0.1600
**Saccharimonadaceae_X**	0.6565	0.7800	0.0005	0.0200	0.0130	0.0930	0.4124	0.5900
**Saccharimonadales_X_X**	1.0000	1.0000	0.2860	0.5300	0.0485	0.1900	0.5139	0.6800
**Saccharofermentans**	0.9323	0.9900	0.0007	0.0200	0.0780	0.2500	0.0011	0.0240
**Schwartzia**	0.0166	0.0970	0.3777	0.5600	0.1432	0.3700	0.1277	0.3400
**Selenomonadaceae_X**	0.7443	0.8600	0.4803	0.6600	0.4851	0.6700	0.1080	0.3100
**Selenomonas_A**	NA	NA	0.3593	0.5400	0.1662	0.3700	NA	NA
**Selenomonas_B**	0.0071	0.0610	0.0029	0.0390	0.0071	0.0610	0.0788	0.2500
**Serratia**	0.6513	0.7800	0.1662	0.3700	0.8939	0.9700	0.6513	0.7800
**Shinella**	0.1662	0.3700	0.0011	0.0240	0.0071	0.0610	0.1662	0.3700
**Simonsiella**	0.5692	0.7300	0.7062	0.8300	0.4301	0.6200	0.0898	0.2700
**Sneathia**	0.1662	0.3700	0.1662	0.3700	0.0788	0.2500	0.3593	0.5400
**Soleaferrea**	0.1336	0.3500	0.0040	0.0470	0.0447	0.1900	0.0032	0.0410
**Sphaerochaeta**	NA	NA	0.0011	0.0240	0.0071	0.0610	0.1662	0.3700
**Sphaerochaeta_A**	0.4091	0.5900	0.9486	1.0000	0.0996	0.3000	0.8257	0.9200
**Sphaerochaetaceae_X**	1.0000	1.0000	0.2415	0.4700	0.1277	0.3400	0.3864	0.5700
**Sphingobacterium**	0.2394	0.4700	0.2394	0.4700	0.0075	0.0630	0.1058	0.3000
**Sphingomonas**	0.0004	0.0170	0.0788	0.2500	0.0165	0.0970	0.1662	0.3700
**Sphingopyxis_A**	0.0031	0.0410	0.0255	0.1300	0.0062	0.0610	0.0011	0.0240
**Spirochaetia_X_X_X**	0.1978	0.4100	0.4428	0.6300	0.0519	0.2000	0.1135	0.3200
**Spirochaetota_X_X_X_X**	0.2006	0.4100	0.4685	0.6600	0.5830	0.7300	0.5629	0.7300
**Stenotrophomonas**	0.0027	0.0390	0.0002	0.0140	0.0048	0.0530	0.0114	0.0860
**Streptococcus**	0.7553	0.8600	0.1135	0.3200	0.0007	0.0200	0.0008	0.0230
**Succiniclasticum**	0.8428	0.9300	0.1978	0.4100	0.0597	0.2200	0.0449	0.1900
**Succinimonas**	0.0395	0.1700	0.2141	0.4400	0.7508	0.8600	0.0166	0.0970
**Succinivibrio**	0.2089	0.4300	0.1833	0.3900	0.5137	0.6800	1.0000	1.0000

**Sutterella**	0.5807	0.7300	0.5139	0.6800	0.5139	0.6800	0.1662	0.3700
**Syner-01**	0.5807	0.7300	0.3593	0.5400	0.2860	0.5300	0.3593	0.5400
**Synergistales_X_X**	1.0000	1.0000	0.0136	0.0960	0.0549	0.2100	0.5420	0.7100
**Synergistes**	0.0481	0.1900	0.1496	0.3700	0.7813	0.8800	0.0683	0.2400
**SZUA-359**	0.0871	0.2700	0.0118	0.0870	0.0005	0.0190	0.1669	0.3700
**Tannerella**	0.2478	0.4800	0.0809	0.2500	0.1548	0.3700	0.6513	0.7800
**Tannerellaceae_X**	0.0188	0.1100	0.4337	0.6200	1.0000	1.0000	0.0558	0.2100
**TF01-11**	0.0048	0.0530	0.2566	0.4900	0.6209	0.7600	0.2726	0.5100
**Thermotalea**	0.0788	0.2500	0.2860	0.5300	0.0788	0.2500	0.0788	0.2500
**Tissierellaceae_X**	1.0000	1.0000	0.6513	0.7800	1.0000	1.0000	1.0000	1.0000
**Tissierellales_X_X**	0.7987	0.9000	0.0780	0.2500	0.5137	0.6800	0.4776	0.6600
**Treponema_A**	1.0000	1.0000	0.1548	0.3700	0.5807	0.7300	0.3593	0.5400
**Treponema_B**	0.5897	0.7300	1.0000	1.0000	0.6193	0.7600	0.0220	0.1200
**Treponema_C**	0.1973	0.4100	0.4650	0.6500	0.4124	0.5900	0.0585	0.2200
**Treponema_D**	0.2415	0.4700	0.7987	0.9000	0.0018	0.0320	0.0165	0.0970
**Treponemataceae_X**	0.4776	0.6600	0.1600	0.3700	0.8874	0.9600	0.7125	0.8300
**Treponematales_X_X**	0.1935	0.4100	0.0005	0.0190	0.0530	0.2100	0.6643	0.7900
**Tumebacillus**	0.3593	0.5400	1.0000	1.0000	1.0000	1.0000	0.3593	0.5400
**UBA1020**	0.3593	0.5400	NA	NA	NA	NA	0.3593	0.5400
**UBA1033**	1.0000	1.0000	0.0262	0.1300	0.0005	0.0190	0.1970	0.4100
**UBA1067**	0.1600	0.3700	0.9323	0.9900	0.9774	1.0000	0.1432	0.3700
**UBA1174**	0.6216	0.7700	0.4591	0.6400	0.8051	0.9000	0.8257	0.9200
**UBA1191**	0.6931	0.8200	0.1305	0.3500	0.7645	0.8700	0.2156	0.4400
**UBA1258**	0.7125	0.8300	0.0083	0.0690	0.2657	0.5000	0.9539	1.0000
**UBA1361**	1.0000	1.0000	0.0387	0.1700	0.0519	0.2000	0.3226	0.5400
**UBA1394**	0.6058	0.7500	0.0040	0.0470	0.0728	0.2500	0.0043	0.0500
**UBA1436**	0.0018	0.0320	0.1600	0.3700	0.0687	0.2400	0.0004	0.0170
**UBA1487**	0.3593	0.5400	0.3593	0.5400	0.5807	0.7300	1.0000	1.0000
**UBA1532**	0.5435	0.7100	0.1392	0.3600	0.0259	0.1300	0.0040	0.0470
**UBA1547**	0.3593	0.5400	NA	NA	NA	NA	NA	NA

**UBA1711**	0.0529	0.2100	0.0887	0.2700	0.7553	0.8600	0.1745	0.3800
**UBA1829**	0.6707	0.7900	0.0009	0.0230	0.0145	0.0970	0.4428	0.6300
**UBA2022**	0.3593	0.5400	0.3593	0.5400	NA	NA	NA	NA
**UBA2450**	0.6105	0.7500	0.4907	0.6700	0.1191	0.3300	0.8750	0.9600
**UBA2705**	NA	NA	NA	NA	0.3593	0.5400	0.3593	0.5400
**UBA3206**	0.9763	1.0000	0.2345	0.4700	0.2592	0.5000	0.1716	0.3800
**UBA4179**	0.3740	0.5600	0.5726	0.7300	0.0308	0.1500	0.5898	0.7300
**UBA4658**	0.5807	0.7300	1.0000	1.0000	0.3593	0.5400	0.3593	0.5400
**UBA5124**	0.6297	0.7700	0.1135	0.3200	0.1277	0.3400	0.0000	0.0075
**UBA5194**	0.2734	0.5100	0.1039	0.3000	0.8574	0.9400	0.7464	0.8600
**UBA636**	0.1798	0.3900	0.0308	0.1500	1.0000	1.0000	0.5139	0.6800
**UBA7182**	0.5972	0.7400	0.6548	0.7800	1.0000	1.0000	0.2356	0.4700
**UBA733**	0.8635	0.9500	0.7150	0.8300	0.3389	0.5400	0.6661	0.7900
**UBA7862**	0.6531	0.7800	0.1392	0.3600	0.2110	0.4300	0.5368	0.7100
**UBA8416**	0.9486	1.0000	0.1341	0.3600	0.1369	0.3600	0.3956	0.5800
**UBA8525**	0.3593	0.5400	1.0000	1.0000	0.2860	0.5300	1.0000	1.0000
**UBA8953**	0.4188	0.6000	0.3474	0.5400	0.0007	0.0200	0.9081	0.9800
**UBA932_X**	0.8106	0.9100	0.0417	0.1800	0.0028	0.0390	0.0089	0.0720
**UBA9983_A_X_X**	NA	NA	NA	NA	0.0071	0.0610	0.0788	0.2500
**V5-8f**	1.0000	1.0000	0.0195	0.1100	0.6513	0.7800	0.5807	0.7300
**Vallitalea_A**	0.3593	0.5400	NA	NA	0.1662	0.3700	NA	NA
**Vampirovibrionia_X_X_X**	0.3593	0.5400	1.0000	1.0000	1.0000	1.0000	1.0000	1.0000
**Varibaculum_A**	0.1836	0.3900	0.7472	0.8600	0.7965	0.9000	0.1110	0.3200
**Veillonella**	0.0114	0.0860	0.0027	0.0390	0.0130	0.0930	0.0246	0.1300
**Veillonella_A**	0.9309	0.9900	0.0770	0.2500	0.1730	0.3800	0.0037	0.0450
**Victivallis**	0.8215	0.9100	0.2537	0.4900	0.2991	0.5400	0.9726	1.0000
**Vitiosangium**	0.5807	0.7300	0.1610	0.3700	0.0090	0.0720	0.5807	0.7300
**Weeksellaceae_X**	0.5139	0.6800	0.2860	0.5300	0.2478	0.4800	1.0000	1.0000
**Weissella**	NA	NA	0.1662	0.3700	0.0788	0.2500	0.0788	0.2500
**Williamwhitmania**	0.6380	0.7800	0.1392	0.3600	0.3064	0.5400	0.7242	0.8400

**Xanthomonadaceae_X**	NA	NA	NA	NA	0.0367	0.1600	0.0788	0.2500
**Xanthomonadales_X_X**	NA	NA	NA	NA	0.0071	0.0610	0.1662	0.3700
**Xanthomonas_B**	0.0585	0.2200	0.0502	0.2000	0.2085	0.4300	1.0000	1.0000
**XBB1006**	0.3593	0.5400	0.6513	0.7800	0.6513	0.7800	0.3281	0.5400
**XYC2-FULL-35-21**	0.8939	0.9700	0.1585	0.3700	0.4709	0.6600	0.5139	0.6800
**YD12-FULL-39-22**	0.1662	0.3700	NA	NA	0.1662	0.3700	0.3593	0.5400
**Zag1**	0.4428	0.6300	0.0121	0.0870	0.0045	0.0510	0.0068	0.0610
**Zag111**	0.1730	0.3800	0.0139	0.0970	0.0223	0.1200	0.0024	0.0380

Table S7: Pairwise-comparison of taxa in Collection-1 with other Collections (2 to 5) of all samples. Suffix "_X" in genus name suggests that taxa was assigned at higher taxonomic levels than genus. Multiple suffixes are kept identifying exact level of assignment.

	Collection-2		Collection-3		Collection-4		Collection-5	
**Taxa**	Wilcoxon test p-value	Wilcoxon test, BH adjusted p-value	Wilcoxon test p-value	Wilcoxon test, BH adjusted p-value	Wilcoxon test p-value	Wilcoxon test, BH adjusted p-value	Wilcoxon test p-value	Wilcoxon test, BH adjusted p-value
**Phylum**								
**Acidobacteriota**	0.3440	0.5500	0.3440	0.5500	0.6059	0.7500	1.0000	1.0000
**Actinobacteriota**	0.7566	0.8500	0.4451	0.6500	0.5353	0.7200	0.7642	0.8500
**Bacteroidota**	0.0153	0.0680	0.0000	0.0002	0.0029	0.0270	0.0019	0.0190
**Bdellovibrionota**	0.0358	0.1300	0.0358	0.1300	0.0037	0.0270	0.0728	0.2000
**Campylobacterota**	0.3058	0.5400	0.0034	0.0270	0.1731	0.3900	0.3266	0.5500
**Chloroflexota**	0.0427	0.1500	0.0004	0.0054	0.0032	0.0270	0.0050	0.0350
**Cyanobacteria**	0.1415	0.3400	0.0153	0.0680	0.0088	0.0510	0.0077	0.0480
**Desulfobacterota**	0.3250	0.5500	0.0284	0.1100	0.0601	0.1800	0.5462	0.7200
**Desulfobacterota_A**	0.8756	0.9200	0.0079	0.0480	0.0265	0.1100	0.9586	0.9800
**Desulfuromonadota**	0.7619	0.8500	0.0574	0.1800	0.0545	0.1700	0.9265	0.9600
**Elusimicrobiota**	0.1472	0.3500	0.0677	0.1900	0.1923	0.4000	0.9919	1.0000
**Fibrobacterota**	0.3305	0.5500	0.1359	0.3300	0.0319	0.1200	0.2638	0.5100
**Firmicutes**	0.2727	0.5100	0.0183	0.0770	0.4429	0.6500	0.5601	0.7300
**Firmicutes_A**	0.1472	0.3500	0.0000	0.0005	0.0000	0.0002	0.0000	0.0004
**Firmicutes_B**	0.0040	0.0290	0.0608	0.1800	0.0097	0.0530	0.0006	0.0080
**Firmicutes_C**	0.0646	0.1900	0.7360	0.8500	0.6022	0.7500	0.2068	0.4200
**Firmicutes_I**	0.0811	0.2200	0.0207	0.0840	0.0103	0.0540	0.0103	0.0540
**Firmicutes_K**	0.0410	0.1500	0.5549	0.7300	0.4404	0.6500	0.1797	0.3900
**Fusobacteriota**	0.3588	0.5700	0.1518	0.3600	0.3173	0.5400	0.1835	0.4000
**Gemmatimonadota**	0.0535	0.1700	0.6059	0.7500	0.0988	0.2600	0.3440	0.5500
**Myxococcota**	0.7726	0.8600	0.6798	0.8000	0.2313	0.4500	0.0001	0.0016
**Patescibacteria**	0.0094	0.0520	0.0153	0.0680	0.0001	0.0016	0.0000	0.0000
**Planctomycetota**	0.1904	0.4000	0.0014	0.0150	0.0091	0.0520	0.0182	0.0770
**Proteobacteria**	0.0271	0.1100	0.0120	0.0590	0.5464	0.7200	0.0437	0.1500

**Riflebacteria**	0.0489	0.1600	0.0814	0.2200	0.0001	0.0022	0.2977	0.5400
**Spirochaetota**	0.8622	0.9100	0.0172	0.0760	0.4308	0.6400	0.9919	1.0000
**Synergistota**	0.8933	0.9300	0.2047	0.4200	0.0851	0.2300	0.9918	1.0000
**Unclassified Phylum**	0.0153	0.0680	0.0000	0.0002	0.0000	0.0001	0.0027	0.0260
**Verrucomicrobiota**	0.5062	0.7000	0.7670	0.8500	0.1994	0.4100	0.9106	0.9400
**Genus**								
**32-67-11**	NA	NA	0.1617	0.3400	0.0103	0.0550	0.0410	0.1400
**4C28d-15_X_X**	0.2019	0.3900	0.1485	0.3300	0.0112	0.0590	0.6764	0.8100
**992a**	0.7160	0.8400	0.1647	0.3400	0.0032	0.0270	0.4760	0.6600
**ABY1_X_X_X**	0.7410	0.8600	0.7410	0.8600	0.7410	0.8600	0.1617	0.3400
**Acetivibrionaceae_X**	0.9916	1.0000	0.0463	0.1500	0.0323	0.1200	0.0062	0.0420
**Acetoanaerobium**	NA	NA	NA	NA	NA	NA	0.0103	0.0550
**Acetobacteraceae_X**	0.0811	0.2200	0.0207	0.0880	0.0207	0.0880	0.0207	0.0880
**Acholeplasma_C**	0.0052	0.0370	0.1994	0.3900	0.0587	0.1800	0.0507	0.1600
**Acholeplasma_D**	0.0264	0.1100	0.0004	0.0064	0.0000	0.0006	0.0008	0.0100
**Acholeplasmatales_X_X**	0.0017	0.0180	0.0909	0.2400	0.2115	0.4000	0.4798	0.6700
**Achromobacter**	0.3225	0.5300	0.0241	0.0990	0.0069	0.0450	0.5720	0.7400
**Acidovorax_E**	0.3379	0.5300	0.0811	0.2200	0.0207	0.0880	0.1617	0.3400
**Acinetobacter**	0.9590	1.0000	0.1647	0.3400	0.0253	0.1000	0.4331	0.6200
**Actinobacillus**	0.5567	0.7300	0.8944	0.9700	1.0000	1.0000	0.0252	0.1000
**Actinobacillus_A**	0.6321	0.7800	0.5227	0.7000	0.6869	0.8200	0.6381	0.7800
**Actinomyces**	0.4591	0.6400	0.7967	0.9000	0.2317	0.4300	0.1087	0.2700
**Actinomycetaceae_X**	0.3305	0.5300	0.1009	0.2500	0.1127	0.2700	0.0008	0.0100
**Actinomycetales_X_X**	0.1617	0.3400	0.0410	0.1400	NA	NA	0.3379	0.5300
**Acutalibacteraceae_X**	0.9751	1.0000	0.8102	0.9100	0.0180	0.0840	0.3213	0.5300
**Aerococcaceae_X**	0.6834	0.8200	0.0755	0.2200	0.7239	0.8500	0.8850	0.9600
**Aerococcus**	NA	NA	0.0103	0.0550	0.0207	0.0880	0.0410	0.1400
**Agarilytica**	NA	NA	0.1617	0.3400	0.0011	0.0130	0.0811	0.2200
**Agitococcus**	0.0104	0.0560	0.0147	0.0720	0.5877	0.7500	0.3801	0.5700
**Akkermansia**	0.3019	0.5200	0.0002	0.0033	0.5720	0.7400	0.1674	0.3500

**Alcanivorax_A**	0.0000	0.0002	0.0000	0.0002	0.0478	0.1500	0.0368	0.1400
**Algoriphagus**	NA	NA	NA	NA	0.0050	0.0360	NA	NA
**Aliarcobacter**	NA	NA	0.0050	0.0360	NA	NA	0.3379	0.5300
**Alishewanella**	0.0000	0.0000	0.0000	0.0000	0.0000	0.0000	0.0000	0.0006
**Alphaproteobacteria_X_X_X**	0.3481	0.5300	0.3588	0.5500	0.1592	0.3400	0.2011	0.3900
**Anaerofilum**	0.8488	0.9400	0.0078	0.0490	0.0110	0.0580	0.2503	0.4600
**Anaerofustis**	0.9219	0.9900	0.1969	0.3900	0.5986	0.7500	0.1159	0.2800
**Anaerorhabdus**	0.1737	0.3600	0.5150	0.7000	0.7274	0.8500	0.5150	0.7000
**Anaerosporobacter**	0.0811	0.2200	0.0207	0.0880	0.3379	0.5300	NA	NA
**Anaerotignum**	0.7497	0.8700	0.0117	0.0610	0.1341	0.3100	0.1269	0.3000
**Anaerovibrio**	0.8859	0.9600	0.0040	0.0320	0.0000	0.0011	0.0212	0.0890
**Anaerovoracaceae_X**	0.0505	0.1600	0.4082	0.5900	0.0672	0.2000	0.0001	0.0026
**Aquabacterium**	0.0000	0.0011	0.0000	0.0000	0.0000	0.0006	0.0811	0.2200
**Arabia**	0.5391	0.7100	0.0172	0.0810	0.5391	0.7100	0.3019	0.5200
**Arenimonas**	0.0003	0.0050	0.0007	0.0094	0.0187	0.0860	1.0000	1.0000
**Aromatoleum**	0.3379	0.5300	0.1617	0.3400	0.0811	0.2200	0.0005	0.0075
**Asticcacaulis**	NA	NA	NA	NA	0.0103	0.0550	0.3379	0.5300
**Bacilli_X_X_X**	0.2604	0.4700	0.0466	0.1500	0.0252	0.1000	0.9588	1.0000
**Bacillus**	0.0103	0.0550	0.0389	0.1400	0.0103	0.0550	0.0103	0.0550
**Bacillus_AD**	0.3379	0.5300	0.0358	0.1300	0.3379	0.5300	1.0000	1.0000
**Bacillus_W**	0.3379	0.5300	0.0787	0.2200	0.3379	0.5300	0.3379	0.5300
**Bact-08**	0.8198	0.9100	0.9753	1.0000	0.0591	0.1800	0.0186	0.0860
**Bacteria_X_X_X_X_X**	0.0153	0.0740	0.0000	0.0002	0.0000	0.0001	0.0027	0.0240
**Bacteroidaceae_X**	0.5194	0.7000	0.0507	0.1600	0.5062	0.6900	0.0004	0.0059
**Bacteroidales_X_X**	0.9593	1.0000	0.6753	0.8100	0.0355	0.1300	0.7827	0.8900
**Bacteroides**	0.7491	0.8700	0.5154	0.7000	0.7726	0.8800	0.0062	0.0420
**Bacteroidia_X_X_X**	0.0120	0.0620	0.0012	0.0130	0.0003	0.0045	0.0001	0.0018
**Bdellovibrio**	0.3379	0.5300	0.0811	0.2200	0.0050	0.0360	0.0410	0.1400
**Beijerinckiaceae_X**	0.0103	0.0550	0.0358	0.1300	0.0103	0.0550	0.0103	0.0550
**Bibersteinia**	0.0000	0.0003	0.0000	0.0003	0.0181	0.0840	0.3036	0.5200

**Blastomonas**	0.0811	0.2200	0.0410	0.1400	0.0410	0.1400	0.0811	0.2200
**Blautia_A**	0.3602	0.5500	0.0012	0.0130	0.0081	0.0510	0.0054	0.0380
**Bosea**	0.3379	0.5300	0.0103	0.0550	0.3379	0.5300	NA	NA
**Brachymonas**	0.2074	0.4000	0.8916	0.9700	0.5286	0.7100	0.3237	0.5300
**Brevundimonas**	0.0000	0.0005	0.0000	0.0002	0.0116	0.0600	0.6788	0.8200
**BRH-c57**	0.0811	0.2200	0.1617	0.3400	NA	NA	0.0103	0.0550
**Burkholderiaceae_X**	0.7597	0.8700	0.9419	1.0000	0.5555	0.7300	0.4520	0.6400
**Butyrivibrio**	0.7333	0.8600	0.0000	0.0002	0.0000	0.0001	0.0000	0.0006
**C941**	0.3808	0.5700	0.1011	0.2500	0.2047	0.4000	0.8770	0.9600
**CAG-180**	0.0929	0.2400	0.0036	0.0290	0.0045	0.0350	0.1484	0.3300
**CAG-312**	0.1617	0.3400	0.0000	0.0011	0.0050	0.0360	0.0207	0.0880
**CAG-313**	0.1161	0.2800	0.0005	0.0078	0.0073	0.0470	0.1051	0.2600
**CAG-354**	0.7827	0.8900	0.0036	0.0300	0.0036	0.0300	0.0006	0.0080
**CAG-462**	0.0136	0.0680	0.0136	0.0680	0.1060	0.2600	0.0000	0.0005
**CAG-465**	0.5850	0.7500	0.0728	0.2100	0.6044	0.7500	0.4006	0.5900
**CAG-475**	0.1877	0.3700	0.2051	0.4000	0.0035	0.0290	0.3104	0.5300
**CAG-495**	0.0483	0.1600	0.1994	0.3900	0.0814	0.2200	0.0507	0.1600
**CAG-508_X**	0.1617	0.3400	NA	NA	0.1617	0.3400	0.1617	0.3400
**CAG-826_X**	0.5090	0.6900	0.0017	0.0180	0.0071	0.0460	0.1211	0.2900
**CAG-873**	0.9486	1.0000	0.1314	0.3100	0.0989	0.2500	0.9486	1.0000
**CAG-878**	0.1546	0.3400	0.0006	0.0088	0.0003	0.0053	0.0453	0.1500
**Campylobacter**	0.2562	0.4700	0.2641	0.4800	0.0512	0.1600	0.0016	0.0170
**Campylobacter_A**	0.1293	0.3000	0.9083	0.9800	0.9088	0.9800	0.0096	0.0550
**Campylobacter_B**	0.9620	1.0000	0.5795	0.7400	0.6026	0.7500	0.1558	0.3400
**Campylobacteraceae_X**	1.0000	1.0000	0.1626	0.3400	0.3482	0.5300	0.4194	0.6100
**Capnocytophaga**	0.5720	0.7400	0.5720	0.7400	1.0000	1.0000	1.0000	1.0000
**Caryophanon**	0.3379	0.5300	0.0811	0.2200	0.3379	0.5300	0.1617	0.3400
**Caviibacter**	0.0345	0.1300	0.4183	0.6100	0.8869	0.9600	0.1578	0.3400
**Cellvibrio**	0.0000	0.0011	0.0000	0.0001	0.0000	0.0010	0.0917	0.2400
**Cellvibrionaceae_X**	0.3379	0.5300	0.0103	0.0550	0.0005	0.0075	0.1617	0.3400

**Chishuiella**	0.0024	0.0220	0.0011	0.0130	0.0011	0.0130	0.0024	0.0220
**CHKCI001**	0.3379	0.5300	0.3379	0.5300	0.0410	0.1400	0.3379	0.5300
**Christensenellales_X_X**	0.3136	0.5300	0.1480	0.3300	0.0282	0.1100	0.7001	0.8300
**Chryseobacterium**	0.0988	0.2500	0.0148	0.0720	0.0031	0.0270	0.0284	0.1100
**Chryseobacterium_B**	0.3379	0.5300	NA	NA	0.0811	0.2200	0.1617	0.3400
**Chryseobacterium_D**	1.0000	1.0000	0.6059	0.7500	0.1797	0.3600	0.1063	0.2600
**Cloacibacterium**	0.9917	1.0000	0.0180	0.0840	1.0000	1.0000	0.0087	0.0540
**Clostridia_X_X_X**	0.2912	0.5100	0.0017	0.0180	0.0001	0.0021	0.0001	0.0026
**Clostridium**	0.9175	0.9800	0.8244	0.9200	0.9185	0.9800	0.4619	0.6500
**Clostridium_A**	0.6638	0.8000	0.6606	0.8000	0.1309	0.3100	0.1633	0.3400
**Clostridium_L**	0.5391	0.7100	0.1558	0.3400	0.3379	0.5300	0.3379	0.5300
**Clostridium_M**	0.3379	0.5300	0.3379	0.5300	0.3379	0.5300	0.0358	0.1300
**Clostridium_N**	0.2742	0.4900	0.6165	0.7700	0.3325	0.5300	0.4918	0.6800
**Comamonas**	0.4751	0.6600	0.9895	1.0000	0.7863	0.8900	0.6644	0.8000
**Corynebacterium**	0.0207	0.0880	0.1617	0.3400	0.3379	0.5300	NA	NA
**CP2B**	1.0000	1.0000	0.0241	0.0990	0.3225	0.5300	1.0000	1.0000
**Cytophagales_X_X**	0.6059	0.7500	0.0017	0.0180	0.0148	0.0720	0.1674	0.3500
**Denitrobacterium**	0.3991	0.5900	0.3793	0.5700	0.2901	0.5100	0.9828	1.0000
**Dermatophilaceae_X**	0.4441	0.6300	0.5805	0.7400	0.3503	0.5400	0.4901	0.6800
**Desulfobacterota_X_X_X_X**	0.3250	0.5300	0.0284	0.1100	0.0601	0.1800	0.5462	0.7200
**Desulfovibrio**	0.7118	0.8400	0.9486	1.0000	0.3056	0.5200	0.5391	0.7100
**Desulfovibrionaceae_X**	0.9026	0.9700	0.1108	0.2700	0.0397	0.1400	0.4250	0.6100
**Devosia**	0.0011	0.0130	0.0002	0.0042	0.0024	0.0220	0.0103	0.0550
**Dialister_A**	0.0988	0.2500	0.0031	0.0270	0.0535	0.1700	0.6059	0.7500
**Dichelobacter**	0.1236	0.2900	0.0037	0.0300	0.2107	0.4000	0.4336	0.6200
**Dietzia**	0.0148	0.0720	0.0917	0.2400	1.0000	1.0000	1.0000	1.0000
**Dongia**	0.0011	0.0130	0.0005	0.0075	0.0000	0.0003	0.0001	0.0023
**Dorea**	NA	NA	0.0103	0.0550	0.1617	0.3400	NA	NA
**DTU053**	0.4536	0.6400	0.0272	0.1100	0.0350	0.1300	0.2942	0.5200
**Duncaniella**	0.0222	0.0930	0.1063	0.2600	0.3019	0.5200	1.0000	1.0000

**Dysgonomonadaceae_X**	1.0000	1.0000	1.0000	1.0000	0.0577	0.1800	0.1674	0.3500
**Echinicola**	0.0001	0.0027	0.0000	0.0000	0.0000	0.0007	0.0075	0.0480
**Eggerthellaceae_X**	0.9075	0.9800	0.0460	0.1500	0.0214	0.0900	0.7505	0.8700
**Elizabethkingia**	0.0811	0.2200	0.3379	0.5300	0.0103	0.0550	0.0207	0.0880
**Emergencia**	0.6746	0.8100	0.7736	0.8800	0.8613	0.9500	0.3602	0.5500
**Empedobacter**	0.0024	0.0220	0.0001	0.0023	0.0000	0.0002	0.0011	0.0130
**Endomicrobium**	0.5471	0.7200	0.7538	0.8700	0.9101	0.9800	0.8848	0.9600
**Endomicrobium_A**	0.0002	0.0037	0.0000	0.0000	0.0000	0.0001	0.0000	0.0003
**Enterobacterales_X_X**	0.0309	0.1200	0.6456	0.7900	0.3956	0.5800	0.6456	0.7900
**Enterobacteriaceae_X**	0.0000	0.0011	0.0029	0.0250	0.0003	0.0050	0.0055	0.0380
**Enterococcaceae_X**	0.1370	0.3200	0.0247	0.1000	0.0946	0.2400	0.1234	0.2900
**Enterococcus**	0.0050	0.0360	0.0011	0.0130	0.0000	0.0011	0.0002	0.0042
**Erysipelatoclostridiaceae_X**	NA	NA	0.0811	0.2200	0.1617	0.3400	0.3379	0.5300
**Erysipelothrix**	0.1173	0.2800	0.0053	0.0380	0.0160	0.0770	0.3848	0.5700
**Erysipelotrichaceae_X**	0.2089	0.4000	0.4246	0.6100	0.5849	0.7500	0.1861	0.3700
**Erysipelotrichales_X_X**	0.6059	0.7500	1.0000	1.0000	0.3440	0.5300	0.6059	0.7500
**Eubacterium_C**	0.4404	0.6300	0.0410	0.1400	0.9367	1.0000	0.3757	0.5600
**Eubacterium_E**	0.0030	0.0260	0.0000	0.0000	0.0000	0.0001	0.0003	0.0045
**Eubacterium_F**	0.0078	0.0490	0.5062	0.6900	0.7827	0.8900	0.9589	1.0000
**Eubacterium_Q**	0.6269	0.7700	0.4021	0.5900	0.4389	0.6200	0.0030	0.0260
**Eubacterium_S**	0.0756	0.2200	0.0069	0.0450	0.3848	0.5700	0.0107	0.0570
**F0040**	0.9268	0.9900	0.1153	0.2800	0.0194	0.0880	0.0003	0.0050
**F0058**	1.0000	1.0000	0.8153	0.9100	0.5892	0.7500	0.0283	0.1100
**F0422**	0.6830	0.8200	0.6556	0.8000	0.2141	0.4100	0.1653	0.3400
**F0428**	0.1835	0.3700	0.0540	0.1700	0.0358	0.1300	0.0423	0.1400
**F082**	0.2011	0.3900	0.2550	0.4700	0.0459	0.1500	0.9919	1.0000
**FD2005**	0.0524	0.1700	0.0001	0.0019	0.0007	0.0093	0.0010	0.0120
**Fermentimonas**	0.9716	1.0000	0.6534	0.8000	0.6493	0.7900	0.0551	0.1700
**Fibrobacter**	0.3842	0.5700	0.1359	0.3100	0.0374	0.1400	0.2068	0.4000
**Fibrobacter_A**	0.2465	0.4500	0.7935	0.9000	0.2403	0.4500	0.1442	0.3300

**Firm-04**	0.0455	0.1500	0.9090	0.9800	0.5789	0.7400	0.9915	1.0000
**Firm-16**	0.7615	0.8800	0.4542	0.6400	0.9026	0.9700	0.1803	0.3600
**Firmicutes_A_X_X_X_X**	0.2232	0.4200	0.5518	0.7200	0.3949	0.5800	0.9185	0.9800
**Firmicutes_B_X_X_X_X**	0.0040	0.0320	0.0608	0.1800	0.0097	0.0550	0.0006	0.0088
**Flavobacteriaceae_X**	0.9919	1.0000	0.6753	0.8100	0.5328	0.7100	0.0969	0.2500
**Flavobacteriales_X_X**	0.0075	0.0480	0.3374	0.5300	0.0018	0.0180	0.0690	0.2000
**Flavobacterium**	0.0003	0.0053	0.0000	0.0000	0.0000	0.0000	0.0000	0.0003
**Flavobacterium_A**	0.0008	0.0100	0.0000	0.0000	0.0000	0.0000	0.0037	0.0300
**Flexilinea**	0.0427	0.1400	0.0004	0.0062	0.0032	0.0270	0.0050	0.0360
**Fodinicurvata**	0.2012	0.3900	0.8859	0.9600	0.3019	0.5200	0.0811	0.2200
**Frateuria**	0.3379	0.5300	0.1617	0.3400	0.1617	0.3400	0.0811	0.2200
**Fusicatenibacter**	0.6956	0.8300	0.0010	0.0120	0.0072	0.0470	0.0056	0.0390
**Fusobacterium**	0.0410	0.1400	0.0811	0.2200	0.0410	0.1400	0.3379	0.5300
**Fusobacterium_A**	0.3581	0.5500	0.5842	0.7500	0.9589	1.0000	0.0199	0.0880
**Fusobacterium_C**	0.2522	0.4600	0.0303	0.1200	0.0649	0.1900	0.9507	1.0000
**Gammaproteobacteria_X_X_X**	0.0002	0.0032	0.3104	0.5300	0.0106	0.0570	0.0972	0.2500
**Gastranaerophilaceae_X**	0.2117	0.4000	0.1492	0.3300	0.0144	0.0710	0.2225	0.4200
**GCA-2733575**	NA	NA	0.0103	0.0550	0.3379	0.5300	NA	NA
**GCA-900066135**	0.3379	0.5300	0.0410	0.1400	NA	NA	0.3379	0.5300
**GCA-900066495**	0.2822	0.5000	0.0008	0.0100	0.0728	0.2100	0.3019	0.5200
**GCA-900066905**	0.3025	0.5200	0.0021	0.0210	0.0000	0.0003	0.0094	0.0550
**GCA-900066995**	0.1589	0.3400	0.7636	0.8800	0.5982	0.7500	0.7767	0.8900
**Gemella_A**	0.2466	0.4500	0.0112	0.0590	0.0004	0.0060	0.4194	0.6100
**Gemmatimonadaceae_X**	0.0535	0.1700	1.0000	1.0000	0.3379	0.5300	0.3379	0.5300
**Gemmatimonas**	NA	NA	0.3379	0.5300	0.0207	0.0880	0.0811	0.2200
**Gemmobacter_A**	0.3379	0.5300	0.0207	0.0880	0.3379	0.5300	0.0000	0.0011
**Geothermobacter**	0.7619	0.8800	0.0574	0.1800	0.0545	0.1700	0.9265	0.9900
**Gluconobacter**	0.1617	0.3400	0.0410	0.1400	0.0103	0.0550	0.0410	0.1400
**Glutamicibacter**	0.3379	0.5300	0.1617	0.3400	0.0410	0.1400	0.0207	0.0880
**GN02-873**	0.0396	0.1400	0.0017	0.0180	0.7538	0.8700	0.7580	0.8700

**Gottschalkiaceae_X**	0.0103	0.0550	0.0422	0.1400	0.0389	0.1400	0.2201	0.4200
**GWE2-31-10**	0.3603	0.5500	0.3331	0.5300	0.9534	1.0000	0.5454	0.7200
**GWF2-44-16**	0.0850	0.2200	0.0172	0.0810	0.5720	0.7400	0.2822	0.5000
**Halomonas_A**	NA	NA	0.0103	0.0550	0.3379	0.5300	0.3379	0.5300
**Helcococcaceae_X**	0.2977	0.5200	0.0154	0.0750	0.0048	0.0360	0.0037	0.0300
**Helcococcus**	0.0281	0.1100	0.0000	0.0007	0.0029	0.0250	0.9620	1.0000
**Hepatobacter**	0.0811	0.2200	0.0001	0.0023	0.0000	0.0011	0.0011	0.0130
**Humitalea**	0.0410	0.1400	0.5567	0.7300	0.0410	0.1400	0.0410	0.1400
**Hungatella_A**	0.0444	0.1500	0.0000	0.0000	0.0000	0.0000	0.0000	0.0000
**Hyphomonas**	NA	NA	0.1617	0.3400	0.0103	0.0550	0.0207	0.0880
**Inquilinus**	0.0103	0.0550	0.0103	0.0550	0.0358	0.1300	0.0103	0.0550
**Kapabacteriaceae_X**	0.2742	0.4900	0.3566	0.5400	0.2410	0.4500	0.5462	0.7200
**Kingella**	0.0735	0.2100	0.0463	0.1500	0.2966	0.5200	0.8442	0.9300
**Kiritimatiellae_X_X_X**	0.2047	0.4000	0.1904	0.3800	0.8771	0.9600	0.4831	0.6700
**KLE1796**	0.0207	0.0880	0.0024	0.0220	0.0207	0.0880	0.3379	0.5300
**Kocuria**	0.0103	0.0550	0.0103	0.0550	0.0103	0.0550	0.0103	0.0550
**Lachnoanaerobaculum**	0.4991	0.6800	0.0001	0.0030	0.0021	0.0210	0.0090	0.0550
**Lachnospira**	0.9828	1.0000	0.2331	0.4300	0.0000	0.0000	0.0039	0.0320
**Lachnospiraceae_X**	0.0483	0.1600	0.0000	0.0001	0.0000	0.0002	0.0000	0.0005
**Lachnospirales_X_X**	0.6575	0.8000	0.2011	0.3900	0.3305	0.5300	0.0852	0.2200
**Lactobacillales_X_X**	0.4589	0.6400	0.7419	0.8600	0.6246	0.7700	0.2437	0.4500
**Lactobacillus_F**	0.3379	0.5300	1.0000	1.0000	1.0000	1.0000	0.3440	0.5300
**Lactococcus**	0.0024	0.0220	0.0000	0.0011	0.0000	0.0011	0.0011	0.0130
**Lactonifactor**	0.3440	0.5300	0.0161	0.0780	0.0535	0.1700	0.3225	0.5300
**Lancefieldella**	0.3225	0.5300	0.0069	0.0450	0.1674	0.3500	1.0000	1.0000
**Lawsonibacter**	0.9486	1.0000	0.7410	0.8600	0.1617	0.3400	0.5391	0.7100
**Leaf454**	0.5161	0.7000	0.0000	0.0000	0.0000	0.0000	0.0000	0.0001
**Lenti-01**	0.0443	0.1500	0.0000	0.0001	0.0000	0.0010	0.0078	0.0490
**Lentimicrobium**	0.4932	0.6800	0.1654	0.3400	0.8622	0.9500	0.0029	0.0250
**Lentisphaeria_X_X_X**	0.0207	0.0880	0.0811	0.2200	0.1617	0.3400	0.0811	0.2200

**Leptotrichiaceae_X**	0.9620	1.0000	0.7505	0.8700	0.3067	0.5200	0.0410	0.1400
**Leuconostoc**	0.0207	0.0880	0.0000	0.0011	0.0000	0.0011	0.0002	0.0042
**Lysobacter**	0.0103	0.0550	0.0494	0.1600	0.0103	0.0550	0.0103	0.0550
**Mailhella**	0.6053	0.7500	0.0028	0.0240	0.0198	0.0880	0.8571	0.9400
**Mannheimia**	0.0693	0.2000	0.4323	0.6200	0.6590	0.8000	0.0182	0.0850
**Marinilabiliaceae_X**	0.7118	0.8400	0.1575	0.3400	0.1575	0.3400	0.5391	0.7100
**Marseille-P3160**	1.0000	1.0000	1.0000	1.0000	1.0000	1.0000	0.5391	0.7100
**Massilia_B**	NA	NA	0.0000	0.0003	NA	NA	NA	NA
**Massilibacteroides**	0.3409	0.5300	0.5880	0.7500	0.0046	0.0350	0.0001	0.0020
**Metamycoplasma**	0.1833	0.3700	0.3231	0.5300	0.5754	0.7400	0.1063	0.2600
**Methylobacterium**	1.0000	1.0000	0.3440	0.5300	0.3440	0.5300	0.6059	0.7500
**Micavibrionaceae_X**	0.3379	0.5300	NA	NA	0.3379	0.5300	0.0050	0.0360
**Micrococcaceae_X**	0.1617	0.3400	0.1617	0.3400	0.3379	0.5300	0.3379	0.5300
**Monoglobales_X_X**	1.0000	1.0000	0.5391	0.7100	0.2822	0.5000	0.2822	0.5000
**Monoglobus**	0.1797	0.3600	0.0102	0.0550	0.1010	0.2500	0.5281	0.7100
**Moraxella**	0.2638	0.4800	0.0814	0.2200	0.9756	1.0000	0.4803	0.6700
**Moraxella_A**	1.0000	1.0000	0.1633	0.3400	0.9714	1.0000	0.6830	0.8200
**Moraxella_C**	0.0811	0.2200	0.0050	0.0360	NA	NA	0.1617	0.3400
**Moraxellaceae_X**	0.0110	0.0580	0.0098	0.0550	0.3194	0.5300	0.5144	0.7000
**Mucilaginibacter**	0.0811	0.2200	0.1617	0.3400	0.1617	0.3400	0.0207	0.0880
**Muribaculaceae_X**	0.5424	0.7200	0.0928	0.2400	0.1403	0.3200	0.9096	0.9800
**Myroides**	0.3379	0.5300	0.3379	0.5300	0.3379	0.5300	0.0811	0.2200
**Ndongobacter**	0.2691	0.4900	0.1722	0.3500	0.9714	1.0000	0.0811	0.2200
**Negativicutes_X_X_X**	0.7636	0.8800	0.0887	0.2300	0.1010	0.2500	0.0960	0.2500
**Neisseria_B**	0.1141	0.2800	0.2635	0.4800	0.0904	0.2400	0.8601	0.9500
**Neisseria_G**	0.0811	0.2200	0.3225	0.5300	0.2822	0.5000	0.3225	0.5300
**Neisseriaceae_X**	0.3742	0.5600	0.6798	0.8200	0.9671	1.0000	0.1697	0.3500
**Neorhizobium**	0.4580	0.6400	0.3999	0.5900	0.8228	0.9200	0.6351	0.7800
**Niveispirillum**	0.0207	0.0880	NA	NA	0.0207	0.0880	0.0050	0.0360
**NS-102**	0.0024	0.0220	0.0103	0.0550	0.0024	0.0220	0.0410	0.1400

**OEMR01**	0.8992	0.9700	0.3221	0.5300	0.2325	0.4300	0.3440	0.5300
**OLB17**	0.3440	0.5300	0.3440	0.5300	0.6059	0.7500	1.0000	1.0000
**Olegusella**	1.0000	1.0000	0.1448	0.3300	0.5720	0.7400	0.5391	0.7100
**Olsenella**	0.0008	0.0100	0.0032	0.0270	0.0689	0.2000	0.3763	0.5600
**Opitutaceae_X**	0.8143	0.9100	0.1106	0.2700	0.0113	0.0590	0.8302	0.9200
**Opitutales_X_X**	0.3440	0.5300	0.6059	0.7500	1.0000	1.0000	0.3379	0.5300
**Opitutus**	NA	NA	0.0207	0.0880	0.0207	0.0880	NA	NA
**Oribacterium**	0.1960	0.3900	0.0008	0.0100	0.0000	0.0006	0.0046	0.0350
**Orrella**	0.0434	0.1500	0.8863	0.9600	0.3237	0.5300	0.3237	0.5300
**Oscillibacter**	0.5227	0.7000	0.0201	0.0880	0.0043	0.0330	0.1320	0.3100
**Oscillospiraceae_X**	0.7054	0.8300	0.0437	0.1500	0.0003	0.0048	0.0000	0.0003
**Oscillospirales_X_X**	0.5328	0.7100	0.0000	0.0010	0.0000	0.0000	0.0045	0.0350
**Paenibacillus_J**	0.1617	0.3400	0.1617	0.3400	0.1617	0.3400	0.0103	0.0550
**Paeniclostridium**	0.5391	0.7100	0.0172	0.0810	0.3019	0.5200	1.0000	1.0000
**PALSA-1355**	0.9900	1.0000	0.4991	0.6800	0.7630	0.8800	0.6906	0.8200
**Paludibacteraceae_X**	0.8769	0.9600	0.4029	0.5900	0.6424	0.7900	0.1781	0.3600
**Paramesorhizobium**	0.8072	0.9100	0.1737	0.3600	0.9442	1.0000	0.7274	0.8500
**Paramuribaculum**	0.0408	0.1400	0.0728	0.2100	0.0270	0.1100	0.9768	1.0000
**Parapedobacter**	0.6059	0.7500	0.1063	0.2600	0.1063	0.2600	0.6059	0.7500
**Paraprevotella**	0.0928	0.2400	0.0215	0.0900	0.0035	0.0290	0.5701	0.7400
**Parvibaculum**	0.0000	0.0000	0.0000	0.0000	0.0000	0.0000	0.0005	0.0074
**Parvimonas**	0.2549	0.4700	0.3222	0.5300	0.8717	0.9500	0.0187	0.0860
**Pasteurellaceae_X**	0.1923	0.3800	0.0039	0.0320	0.7360	0.8600	0.1719	0.3500
**Pauljensenia**	0.4271	0.6100	0.5962	0.7500	0.1846	0.3700	0.0207	0.0880
**Pedosphaeraceae_X**	0.0000	0.0007	0.0000	0.0006	0.0000	0.0004	0.0001	0.0021
**PeH17**	0.2638	0.4800	0.0814	0.2200	0.0437	0.1500	0.7570	0.8700
**Peptostreptococcaceae_X**	0.8290	0.9200	0.4909	0.6800	0.8221	0.9200	0.0212	0.0890
**Peptostreptococcales_X_X**	0.3258	0.5300	0.3638	0.5500	0.8932	0.9700	0.0071	0.0460
**Phycorickettsia**	1.0000	1.0000	1.0000	1.0000	0.1558	0.3400	0.0850	0.2200
**Pigmentiphaga**	0.2031	0.4000	0.1626	0.3400	0.0850	0.2200	0.0378	0.1400

**Pirellulaceae_X**	0.3379	0.5300	0.0050	0.0360	0.0410	0.1400	0.3379	0.5300
**Pirellulales_X_X**	0.1398	0.3200	0.0000	0.0002	0.0000	0.0002	0.0002	0.0037
**Planctomycetes_X_X_X**	0.5691	0.7400	0.1578	0.3400	0.0439	0.1500	0.1298	0.3000
**Planctomycetota_X_X_X_X**	0.0008	0.0100	0.0097	0.0550	0.0076	0.0480	0.0098	0.0550
**Porphyromonas**	0.1768	0.3600	0.5430	0.7200	0.6427	0.7900	0.0129	0.0660
**Prevotella**	0.0055	0.0380	0.0003	0.0045	0.0072	0.0470	0.5740	0.7400
**Prevotellamassilia**	0.0072	0.0470	0.0048	0.0360	0.0004	0.0068	0.0001	0.0027
**Prolixibacteraceae_X**	0.7054	0.8300	0.0972	0.2500	0.0415	0.1400	0.6604	0.8000
**Prosthecobacter**	0.3379	0.5300	0.0207	0.0880	0.0000	0.0006	0.0000	0.0006
**Proteiniclasticum**	0.1314	0.3100	0.0000	0.0000	0.0000	0.0000	0.0005	0.0075
**Proteobacteria_X_X_X_X**	0.2300	0.4300	0.7984	0.9000	0.1719	0.3500	0.0002	0.0036
**Pseudaminobacter**	0.0811	0.2200	0.0000	0.0003	0.0207	0.0880	0.0410	0.1400
**Pseudomonadaceae_X**	0.3023	0.5200	0.0074	0.0480	0.2291	0.4300	0.3025	0.5200
**Pseudomonadales_X_X**	0.2466	0.4500	0.0260	0.1000	0.0007	0.0098	0.4194	0.6100
**Pseudomonas_A**	0.0003	0.0048	0.0000	0.0008	0.0191	0.0880	0.3377	0.5300
**Pseudomonas_B**	0.6044	0.7500	0.2980	0.5200	0.9306	0.9900	0.2331	0.4300
**Pseudomonas_D**	0.0011	0.0130	0.0000	0.0011	0.0207	0.0880	0.0811	0.2200
**Pseudomonas_E**	0.6113	0.7600	0.2781	0.5000	0.2013	0.3900	0.8389	0.9300
**Pseudomonas_F**	0.0050	0.0360	NA	NA	NA	NA	NA	NA
**Pseudomonas_M**	0.6526	0.8000	0.0050	0.0360	0.0366	0.1300	0.2641	0.4800
**Pygmaiobacter**	0.4470	0.6300	0.0117	0.0610	0.0660	0.2000	0.0380	0.1400
**Pyramidobacter**	0.8365	0.9300	0.2277	0.4300	0.1147	0.2800	1.0000	1.0000
**RC9**	0.0172	0.0810	0.0459	0.1500	0.0230	0.0950	0.0025	0.0230
**RF16**	0.9919	1.0000	0.2465	0.4500	0.8302	0.9200	0.3622	0.5500
**Rhizobiaceae_X**	0.1878	0.3700	0.3025	0.5200	0.3025	0.5200	0.4580	0.6400
**Rhizobiales_X_X**	0.6842	0.8200	0.1261	0.3000	0.1046	0.2600	0.2117	0.4000
**Rhodospirillaceae_X**	0.0011	0.0130	0.0011	0.0130	0.0024	0.0220	0.0410	0.1400
**Riemerella**	0.5391	0.7100	0.0029	0.0250	0.0148	0.0720	0.3379	0.5300
**Roseomonas**	0.0811	0.2200	0.0050	0.0360	0.0207	0.0880	0.0811	0.2200
**Rothia**	0.0050	0.0360	0.0410	0.1400	0.1617	0.3400	NA	NA

**RUG131**	0.4308	0.6200	0.7670	0.8800	0.3104	0.5300	0.7807	0.8900
**RUG163**	0.9919	1.0000	0.0646	0.1900	0.0000	0.0012	0.0048	0.0360
**RUG350**	0.1531	0.3400	0.4308	0.6200	0.4308	0.6200	0.5847	0.7500
**Ruminiclostridium**	0.0363	0.1300	0.0000	0.0000	0.0000	0.0000	0.0000	0.0000
**Ruminiclostridium_C**	0.3622	0.5500	0.0163	0.0780	0.0113	0.0590	0.0128	0.0650
**Ruminiclostridium_D**	0.7809	0.8900	0.0350	0.1300	0.0389	0.1400	0.5818	0.7400
**Ruminiclostridium_F**	0.0473	0.1500	0.9486	1.0000	0.5728	0.7400	0.0954	0.2500
**Ruminococcaceae_X**	0.4641	0.6500	0.5292	0.7100	0.5430	0.7200	0.4034	0.5900
**Ruminococcus**	0.3351	0.5300	0.4615	0.6500	0.0115	0.0600	0.0035	0.0290
**Ruminococcus_C**	0.0587	0.1800	0.0007	0.0098	0.0000	0.0006	0.0000	0.0002
**Ruminococcus_D**	0.7207	0.8500	0.0005	0.0072	0.0032	0.0270	0.0000	0.0009
**Ruminococcus_F**	0.4215	0.6100	0.2027	0.3900	0.0212	0.0900	0.0233	0.0970
**Saccharibacillus**	0.3379	0.5300	0.0811	0.2200	0.0207	0.0880	0.0410	0.1400
**Saccharicrinis**	0.1617	0.3400	0.0000	0.0006	0.0000	0.0006	0.0011	0.0130
**Saccharimonadaceae_X**	0.7468	0.8700	0.0001	0.0023	0.0248	0.1000	0.2808	0.5000
**Saccharimonadales_X_X**	0.2977	0.5200	0.0366	0.1300	0.0112	0.0590	0.4962	0.6800
**Saccharofermentans**	0.0374	0.1400	0.0000	0.0009	0.0002	0.0035	0.0013	0.0140
**Schwartzia**	0.0043	0.0330	0.7827	0.8900	0.5464	0.7200	0.1202	0.2900
**Selenomonadaceae_X**	0.1638	0.3400	0.0101	0.0550	0.0257	0.1000	0.0382	0.1400
**Selenomonas_A**	NA	NA	0.1617	0.3400	0.0024	0.0220	0.3379	0.5300
**Selenomonas_B**	0.0018	0.0180	0.0000	0.0001	0.0000	0.0003	0.0187	0.0860
**Serratia**	0.5728	0.7400	0.3237	0.5300	0.0441	0.1500	0.3237	0.5300
**Shinella**	0.0811	0.2200	0.0005	0.0075	0.0024	0.0220	0.0410	0.1400
**Simonsiella**	0.1534	0.3400	0.5478	0.7200	0.8899	0.9700	0.1089	0.2700
**Sneathia**	0.0410	0.1400	0.0103	0.0550	0.0103	0.0550	0.3379	0.5300
**Soleaferrea**	0.0098	0.0550	0.0000	0.0002	0.0000	0.0006	0.0001	0.0018
**Sphaerochaeta**	1.0000	1.0000	0.0000	0.0003	0.0001	0.0017	0.0095	0.0550
**Sphaerochaeta_A**	0.7942	0.9000	0.0063	0.0430	0.0000	0.0009	0.0479	0.1500
**Sphaerochaetaceae_X**	0.2882	0.5100	0.0001	0.0021	0.0000	0.0011	0.0040	0.0320
**Sphingobacterium**	0.0019	0.0200	0.0000	0.0009	0.0000	0.0001	0.0019	0.0200

**Sphingomonas**	0.0000	0.0001	0.0011	0.0130	0.0000	0.0003	0.0024	0.0220
**Sphingopyxis_A**	0.0056	0.0390	0.1320	0.3100	0.0032	0.0270	0.0001	0.0023
**Spirochaetia_X_X_X**	0.3104	0.5300	0.3956	0.5800	0.4803	0.6700	0.4552	0.6400
**Spirochaetota_X_X_X_X**	0.6985	0.8300	0.5064	0.6900	0.7478	0.8700	0.9417	1.0000
**Stenotrophomonas**	0.0000	0.0000	0.0000	0.0000	0.0000	0.0001	0.0003	0.0048
**Streptococcus**	0.8684	0.9500	0.8932	0.9700	0.4958	0.6800	0.1890	0.3800
**Succiniclasticum**	0.1785	0.3600	0.8461	0.9300	0.8461	0.9300	0.3409	0.5300
**Succinimonas**	0.0007	0.0093	0.0620	0.1900	0.2699	0.4900	0.9261	0.9900
**Succinivibrio**	0.5154	0.7000	0.4332	0.6200	0.0355	0.1300	0.2083	0.4000
**Sutterella**	0.6830	0.8200	0.2822	0.5000	0.2822	0.5000	0.3440	0.5300
**Syner-01**	0.5720	0.7400	1.0000	1.0000	0.0787	0.2200	0.3379	0.5300
**Synergistales_X_X**	0.7736	0.8800	0.0025	0.0230	0.0045	0.0350	0.7687	0.8800
**Synergistes**	0.0284	0.1100	0.3286	0.5300	0.6830	0.8200	0.0584	0.1800
**SZUA-359**	0.0040	0.0320	0.0012	0.0130	0.0000	0.0002	0.0805	0.2200
**Tannerella**	0.2078	0.4000	0.0584	0.1800	0.3991	0.5900	0.9828	1.0000
**Tannerellaceae_X**	0.2378	0.4400	0.3573	0.5400	0.6199	0.7700	0.0059	0.0410
**TF01-11**	0.0373	0.1400	0.0513	0.1600	0.4839	0.6700	0.5317	0.7100
**Thermotalea**	0.0410	0.1400	0.1558	0.3400	0.1448	0.3300	0.0410	0.1400
**Tissierellaceae_X**	1.0000	1.0000	0.3440	0.5300	0.3440	0.5300	1.0000	1.0000
**Tissierellales_X_X**	0.7965	0.9000	0.0422	0.1400	0.1296	0.3000	0.7493	0.8700
**Treponema_A**	0.9828	1.0000	0.2078	0.4000	0.1575	0.3400	0.1617	0.3400
**Treponema_B**	0.7349	0.8600	0.8309	0.9200	0.2155	0.4100	0.1505	0.3400
**Treponema_C**	0.4006	0.5900	0.6103	0.7600	0.6483	0.7900	0.9734	1.0000
**Treponema_D**	0.8609	0.9500	0.8622	0.9500	0.0136	0.0680	0.4454	0.6300
**Treponemataceae_X**	0.1835	0.3700	0.0006	0.0084	0.0513	0.1600	0.2977	0.5200
**Treponematales_X_X**	0.0121	0.0620	0.0000	0.0007	0.0005	0.0072	0.2074	0.4000
**Tumebacillus**	0.0410	0.1400	0.5549	0.7300	0.4404	0.6300	0.1797	0.3600
**UBA1020**	0.0850	0.2200	1.0000	1.0000	0.1558	0.3400	1.0000	1.0000
**UBA1033**	0.2977	0.5200	0.0025	0.0230	0.0000	0.0001	0.1168	0.2800
**UBA1067**	0.2382	0.4400	0.5062	0.6900	0.3622	0.5500	0.6753	0.8100

**UBA1174**	0.3063	0.5200	0.2112	0.4000	0.8083	0.9100	0.2887	0.5100
**UBA1191**	0.3195	0.5300	0.0439	0.1500	0.0996	0.2500	0.5659	0.7400
**UBA1258**	0.0538	0.1700	0.0001	0.0017	0.0003	0.0045	0.0096	0.0550
**UBA1361**	0.8934	0.9700	0.0489	0.1600	0.0012	0.0130	0.3577	0.5500
**UBA1394**	0.6729	0.8100	0.0057	0.0390	0.0090	0.0550	0.0040	0.0320
**UBA1436**	0.0000	0.0002	0.0337	0.1300	0.0043	0.0330	0.0000	0.0006
**UBA1487**	0.1617	0.3400	0.1617	0.3400	0.5996	0.7500	0.9486	1.0000
**UBA1532**	0.6715	0.8100	0.4559	0.6400	0.1025	0.2600	0.0002	0.0037
**UBA1547**	0.0207	0.0880	0.0811	0.2200	0.3379	0.5300	NA	NA
**UBA1711**	0.0422	0.1400	0.1202	0.2900	0.5062	0.6900	0.0081	0.0500
**UBA1829**	0.4188	0.6100	0.0024	0.0220	0.0459	0.1500	0.3514	0.5400
**UBA2022**	0.5720	0.7400	0.6059	0.7500	1.0000	1.0000	0.3379	0.5300
**UBA2450**	0.9916	1.0000	0.7920	0.9000	0.1420	0.3200	0.5025	0.6900
**UBA2705**	0.3379	0.5300	NA	NA	0.1617	0.3400	0.0410	0.1400
**UBA3206**	0.5344	0.7100	0.5223	0.7000	0.1294	0.3000	0.0441	0.1500
**UBA4179**	0.1341	0.3100	0.3277	0.5300	0.0041	0.0320	0.0996	0.2500
**UBA4658**	0.1797	0.3600	0.6059	0.7500	0.3379	0.5300	0.3379	0.5300
**UBA5124**	0.0459	0.1500	0.9593	1.0000	0.0082	0.0510	0.0000	0.0000
**UBA5194**	0.1887	0.3800	0.3486	0.5400	0.1046	0.2600	0.6847	0.8200
**UBA5946**	0.3225	0.5300	0.5720	0.7400	0.6059	0.7500	1.0000	1.0000
**UBA636**	0.3251	0.5300	0.0070	0.0460	0.7753	0.8800	0.8859	0.9600
**UBA6984**	0.5358	0.7100	0.7759	0.8800	0.0937	0.2400	0.5533	0.7300
**UBA7182**	0.6269	0.7700	0.1314	0.3100	0.2331	0.4300	0.0006	0.0080
**UBA733**	0.7738	0.8800	0.7902	0.8900	0.7614	0.8800	0.9244	0.9900
**UBA7862**	0.5330	0.7100	0.0830	0.2200	0.0326	0.1200	0.9175	0.9800
**UBA8416**	0.8412	0.9300	0.1725	0.3500	0.1661	0.3400	0.3848	0.5700
**UBA8525**	0.9163	0.9800	0.4682	0.6500	0.0432	0.1400	0.3453	0.5300
**UBA8953**	0.0489	0.1600	0.0814	0.2200	0.0001	0.0026	0.2977	0.5200
**UBA932_X**	0.7920	0.9000	0.0033	0.0270	0.0004	0.0069	0.0002	0.0044
**UBA9983_A_X_X**	0.3379	0.5300	NA	NA	0.0000	0.0002	0.0001	0.0023

**V5-8f**	1.0000	1.0000	0.0358	0.1300	0.6059	0.7500	0.1797	0.3600
**Vallitalea_A**	1.0000	1.0000	1.0000	1.0000	0.5391	0.7100	1.0000	1.0000
**Vampirovibrionia_X_X_X**	0.6059	0.7500	0.5391	0.7100	1.0000	1.0000	0.6059	0.7500
**Varibaculum_A**	0.2202	0.4200	0.4850	0.6700	0.9075	0.9800	0.2605	0.4700
**Veillonella**	0.0297	0.1200	0.0288	0.1100	0.0539	0.1700	0.0489	0.1600
**Veillonella_A**	0.2993	0.5200	0.9228	0.9900	0.9148	0.9800	0.0209	0.0890
**Verrucomicrobiota_X_X_X_X**	1.0000	1.0000	0.2822	0.5000	0.5391	0.7100	1.0000	1.0000
**Victivallis**	0.8821	0.9600	0.2052	0.4000	0.0522	0.1600	0.8157	0.9100
**Vitiosangium**	0.1558	0.3400	0.0264	0.1100	0.0300	0.1200	0.6263	0.7700
**Weeksellaceae_X**	0.3019	0.5200	0.0787	0.2200	0.1448	0.3300	0.3225	0.5300
**Weissella**	NA	NA	0.0811	0.2200	0.0410	0.1400	0.0103	0.0550
**Williamwhitmania**	0.2594	0.4700	0.0030	0.0260	0.0067	0.0450	0.1038	0.2600
**Xanthomonadaceae_X**	0.3019	0.5200	1.0000	1.0000	0.0222	0.0930	0.0422	0.1400
**Xanthomonadales_X_X**	NA	NA	NA	NA	0.0002	0.0042	0.0103	0.0550
**Xanthomonas_A**	0.1617	0.3400	0.0811	0.2200	0.0207	0.0880	0.1617	0.3400
**Xanthomonas_B**	0.0006	0.0087	0.0057	0.0390	0.3612	0.5500	0.2074	0.4000
**XBB1006**	0.3379	0.5300	0.1927	0.3800	0.1927	0.3800	0.0850	0.2200
**XYC2-FULL-35-21**	0.3602	0.5500	0.0977	0.2500	0.3627	0.5500	0.1558	0.3400
**YD12-FULL-39-22**	0.0728	0.2100	0.2822	0.5000	0.2822	0.5000	0.5391	0.7100
**Zag1**	0.1305	0.3100	0.0287	0.1100	0.0153	0.0740	0.0163	0.0780
**Zag111**	0.0482	0.1600	0.0025	0.0230	0.0085	0.0520	0.0145	0.0720

Table S8: Sequence data statistics for shotgun metagenomic data.

Sample name	Breed	Feed	Type	Collection	Collection day	Paired reads	bases (GB)
**BB14L**	Bikaneri	Bajra	Liquid	Collection-4	42 days	1,039,171	3.4
**BB14S**	Bikaneri	Bajra	Solid	Collection-4	42 days	459,931	1.69
**BB15L**	Bikaneri	Bajra	Liquid	Collection-5	63 days	788,933	2.52
**BB15S**	Bikaneri	Bajra	Solid	Collection-5	63 days	1,071,653	3.49
**BB24L**	Bikaneri	Bajra	Liquid	Collection-4	42 days	544,409	2.03
**BB24S**	Bikaneri	Bajra	Solid	Collection-4	42 days	482,776	1.77
**BB25L**	Bikaneri	Bajra	Liquid	Collection-5	63 days	1,038,806	3.01
**BB25S**	Bikaneri	Bajra	Solid	Collection-5	63 days	700,368	2.54
**BJ14L**	Bikaneri	Jowar	Liquid	Collection-4	42 days	1,046,066	3.2
**BJ14S**	Bikaneri	Jowar	Solid	Collection-4	42 days	699,194	2.56
**BJ15L**	Bikaneri	Jowar	Liquid	Collection-5	63 days	670,354	2.25
**BJ15S**	Bikaneri	Jowar	Solid	Collection-5	63 days	982,794	3.27
**BJ24L**	Bikaneri	Jowar	Liquid	Collection-4	42 days	914,789	2.89
**BJ24S**	Bikaneri	Jowar	Solid	Collection-4	42 days	574,598	2.09
**BJ25L**	Bikaneri	Jowar	Liquid	Collection-5	63 days	541,639	1.7
**BJ25S**	Bikaneri	Jowar	Solid	Collection-5	63 days	831,210	2.92
**BM14L**	Bikaneri	Maize	Liquid	Collection-4	42 days	1,018,284	3.26
**BM14S**	Bikaneri	Maize	Solid	Collection-4	42 days	579,752	2.15
**BM15L**	Bikaneri	Maize	Liquid	Collection-5	63 days	661,435	1.95
**BM15S**	Bikaneri	Maize	Solid	Collection-5	63 days	702,782	2.2
**BM24L**	Bikaneri	Maize	Liquid	Collection-4	42 days	761,214	2.54
**BM24S**	Bikaneri	Maize	Solid	Collection-4	42 days	584,053	2.12
**BM25L**	Bikaneri	Maize	Liquid	Collection-5	63 days	607,263	1.79
**BM25S**	Bikaneri	Maize	Solid	Collection-5	63 days	1,067,565	3.44
**KB14L**	Kachchhi	Bajra	Liquid	Collection-4	42 days	598,951	2.22
**KB14S**	Kachchhi	Bajra	Solid	Collection-4	42 days	456,718	1.67
**KB15L**	Kachchhi	Bajra	Liquid	Collection-5	63 days	835,266	2.47
**KB15S**	Kachchhi	Bajra	Solid	Collection-5	63 days	880,925	2.5
**KB24L**	Kachchhi	Bajra	Liquid	Collection-4	42 days	986,267	3.02
**KB24S**	Kachchhi	Bajra	Solid	Collection-4	42 days	589,990	2.16
**KB25L**	Kachchhi	Bajra	Liquid	Collection-5	63 days	770,299	2.23
**KB25S**	Kachchhi	Bajra	Solid	Collection-5	63 days	632,694	1.77
**KJ14L**	Kachchhi	Jowar	Liquid	Collection-4	42 days	1,110,152	3.51
**KJ14S**	Kachchhi	Jowar	Solid	Collection-4	42 days	744,385	2.68
**KJ15L**	Kachchhi	Jowar	Liquid	Collection-5	63 days	561,268	1.66
**KJ15S**	Kachchhi	Jowar	Solid	Collection-5	63 days	611,033	2.19
**KJ24L**	Kachchhi	Jowar	Liquid	Collection-4	42 days	969,443	3.06
**KJ24S**	Kachchhi	Jowar	Solid	Collection-4	42 days	688,440	2.52
**KJ25L**	Kachchhi	Jowar	Liquid	Collection-5	63 days	696,680	2.22
**KJ25S**	Kachchhi	Jowar	Solid	Collection-5	63 days	677,869	2.46

**KM14L**	Kachchhi	Maize	Liquid	Collection-4	42 days	1,037,269	3.57
**KM14S**	Kachchhi	Maize	Solid	Collection-4	42 days	520,070	1.92
**KM15L**	Kachchhi	Maize	Liquid	Collection-5	63 days	669,326	2.45
**KM15S**	Kachchhi	Maize	Solid	Collection-5	63 days	516,747	1.51
**KM24L**	Kachchhi	Maize	Liquid	Collection-4	42 days	1,091,481	3.53
**KM24S**	Kachchhi	Maize	Solid	Collection-4	42 days	722,541	2.64
**KM25L**	Kachchhi	Maize	Liquid	Collection-5	63 days	495,555	1.82
**KM25S**	Kachchhi	Maize	Solid	Collection-5	63 days	626,663	1.8
